# α‐Amino Radical Halogen Atom Transfer Agents for Metallaphotoredox‐Catalyzed Cross‐Electrophile Couplings of Distinct Organic Halides

**DOI:** 10.1002/cssc.202200906

**Published:** 2022-06-13

**Authors:** Xianhai Tian, Jaspreet Kaur, Shahboz Yakubov, Joshua P. Barham

**Affiliations:** ^1^ Institute of Organic Chemistry University of Regensburg Universitätsstr. 31 93053 Regensburg Germany

**Keywords:** cross coupling, halogen atom transfer, late-stage functionalization, nickel catalysis, photocatalysis

## Abstract

α‐Amino radicals from simple tertiary amines were employed as halogen atom transfer (XAT) agents in metallaphotoredox catalysis for cross‐electrophile couplings of organic bromides with organic iodides. This XAT strategy proved to be efficient for the generation of carbon radicals from a range of partners (alkyl, aryl, alkenyl, and alkynyl iodides). The reactivities of these radical intermediates were captured by nickel catalysis with organobromides including aryl, heteroaryl, alkenyl, and alkyl bromides, enabling six diverse C−C bond formations. Classic named reactions including Negishi, Suzuki, Heck, and Sonogashira reactions were readily achieved in a net‐reductive fashion under mild conditions. More importantly, the cross coupling was viable with either organic bromide or iodide as limiting reactant based on the availability of substrates, which is beneficial to the late‐stage functionalization of complex molecules. The scalability of this method in batch and flow was investigated, further demonstrating its applicability.

## Graphical abstract

**Cross coupling**: A practical dual Ni/photoredox catalyzed cross‐electrophile coupling of organic halides by employing readily available α‐amino radicals as halogen atom transfer agents is reported. Both coupling partners can be flexibly chosen as limiting reactant to forge 6 different types of C−C bond for a broad scope of applications (>70 examples) including late‐stage functionalizations of pharmaceutical compounds. The reaction is scalable in batch and continuous flow.

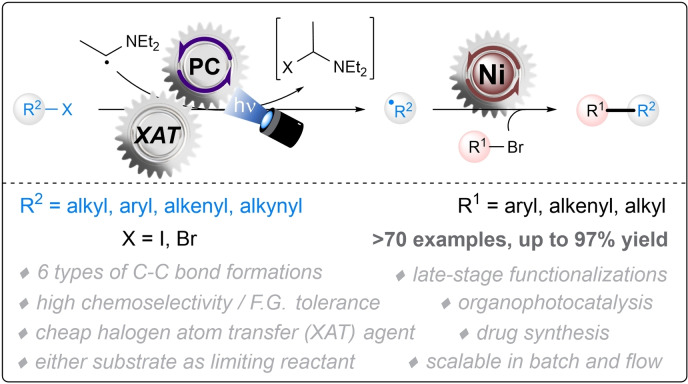

## Introduction

Cross couplings of organic halides with organometallic nucleophiles such as the Negishi reaction have become widely used methods for the constructions of C−C bonds.^[1]^ Metal‐catalyzed reductive cross couplings of two organic halides with superstoichiometric metal (Mg, Zn, Mn) reductants have been demonstrated as a comparatively more streamlined and convenient approach.^[2]^ However, the excess metals and their generated metal salt by‐products complicate reaction workup, generate waste, and encourage side reactions when applied to densely‐functionalized substrates bearing susceptible functional groups. Recently, contemporary redox platforms including photoredox catalysis^[3]^ and electrolysis^[4]^ offer alternate strategies for cross‐electrophile coupling under attractive conditions: at room temperature and without superstoichiometric metals as reductants. However, a key issue in engaging readily available or accessible unactivated organic halides (aryl, alkenyl, and alkyl) is that they generally require deep 1 e^−^ reduction potentials [*E* ^p^_red_<−2.0 V vs. saturated calomel electrode (SCE)]^[5]^ that encourage side reactions, or they require more complex technological advances (multiple photon‐harvesting paradigms,^[6]^ photoelectrochemistry^[7]^) to retain high redox chemoselectivity in couplings.

**Scheme 1:**
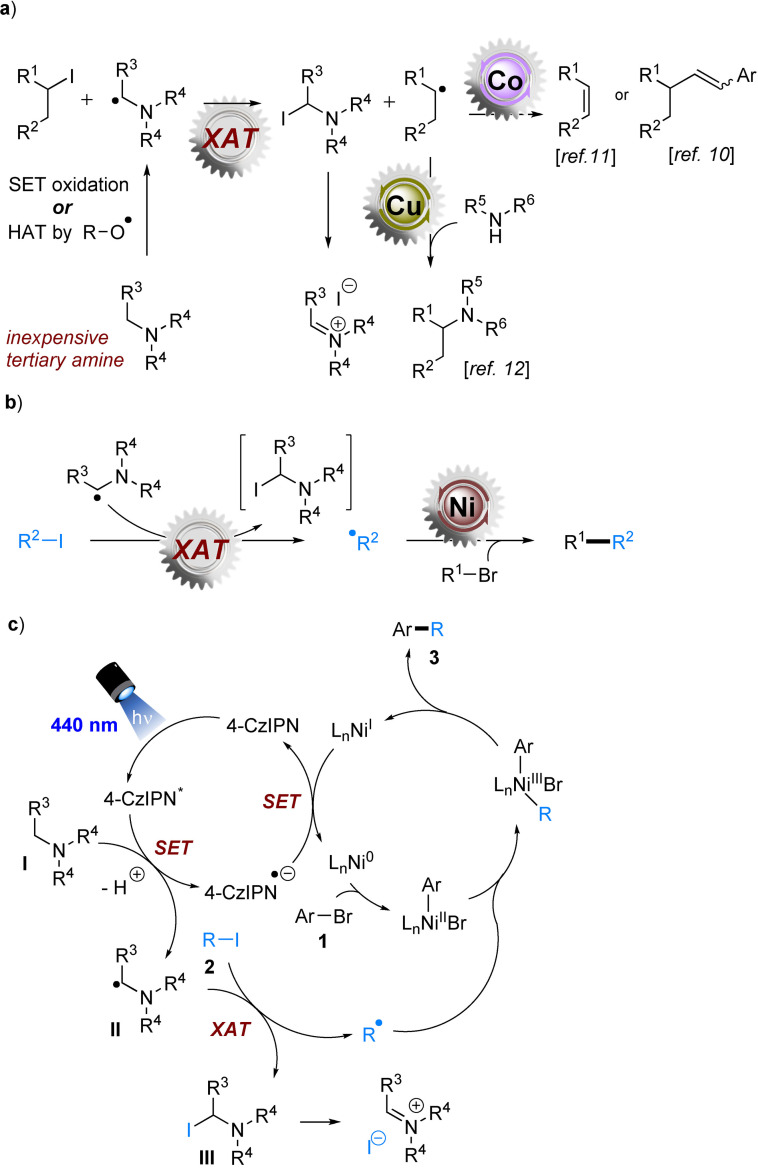
α‐Aminoalkyl radical as XAT agents. (a) State of the art: merging α‐aminoalkyl radical‐mediated XAT with transition metal (Co, Cu) catalysis. (b) This hypothesis: merging α‐aminoalkyl radical‐mediated XAT with Ni catalysis for cross‐electrophile coupling. (c) Proposed mechanism.

In this context, halogen atom transfer (XAT) represents a straightforward, benign strategy for the activation of organic halides.^[8]^ By merging nickel catalysis with photoredox catalysis, MacMillan and co‐workers reported cross coupling of two electrophiles in the presence of stoichiometric, bulky silanes or silanols.[^3a^, ^3b^, ^3c^, ^3d^, ^3e^, ^3g^] Silyl radical species generated from the silanol or silane function as XAT agents to cleave C−X (X=halogen) bonds to form alkyl or aryl radicals whose reactivity is captured by transition metal catalysis to enable C−C bond formations. Xie and co‐workers developed a related combination of metallaphotoredox catalysis with oxygen atom transfer (deoxygenation) for the synthesis of ketones.^[3f]^ Compared to the superstoichiometric metal reducing conditions, these strategies notably broaden the scope of engageable substrates, showcasing (i) the advantages of metallaphotoredox catalysis^[9]^ in late‐stage functionalizations of complex molecules and (ii) how photoredox catalysis can impact the future assembly of pharmaceutically relevant compounds. Despite the indisputable advances, the production costs in large‐scale reactions would be raised by (i) the use of these superstoichiometric silane or silanol XAT agent precursors and (ii) the use of iridium‐based photocatalysts. Comparatively less expensive XAT agents (which, like their by‐products, are volatile for facile removal) and organophotocatalysts would therefore be highly interesting to pursue. α‐Aminoalkyl radicals generated from cheap tertiary amines by either (i) photocatalytic single electron transfer (SET) oxidations or (ii) oxygen radical‐mediated hydrogen atom transfer (HAT) have been employed for XAT by Leonori and co‐workers.^[10]^ This strategy was remarkably effective for transformations of unactivated alkyl iodides to alkyl radicals, whose reactivity was captured with Co[^10^, ^11^] and Cu^[12]^ catalysis for olefinations and aminations (Scheme 1a). In line with our interest in net‐reductive transformations,^[13]^ we questioned whether this XAT strategy could be merged with Ni catalysis to effect a practical, cheap, and broadly applicable cross‐electrophile coupling (Scheme 1b).^[14]^

Taking the coupling of aryl bromide and alkyl iodide as an example, our mechanistic rationale is proposed in Scheme 1c. The reductive quenching of the photoexcited 4‐CzIPN (excited state redox half‐wave potential *E_1/2_=+1.35 V vs. SCE) photocatalyst by tertiary amine **I** affords 4‐CzIPN^.−^ and α‐aminoalkyl radical **II**. Meanwhile, the Ni^0^ catalyst undergoes oxidative addition to ArBr **1** to deliver a Ni^II^ species. α‐Aminoalkyl radical **II** could easily abstract the iodine atom from alkyl iodide **2** to give a carbon radical R⋅ and α‐iodo amine by‐product **III**. The addition of R⋅ to the Ni^II^ center gives rise to the Ni^III^ species which would undergo reductive elimination to provide final product **3** and the Ni^I^ species. 4‐CzIPN^.−^ (*E*_1/2_=−1.21 V vs. SCE) is not able to reduce alkyl iodide **2** (generally *E* ^p^_red_<−2.0 V vs. SCE) directly, but is able to reduce Ni^I^ catalyst back to the Ni^0^ form, simulteneously regenerating the ground‐state 4‐CzIPN catalyst and completing both catalytic cycles.

## Results and Discussion

We assessed the feasibility of our hypothesis by benchmarking the reaction of readily available aryl bromide **1 j** and alkyl iodide **2 a** (Table 1). With **1 j** as limiting reactant (0.2 mmol), the optimal conditions employed 4‐CzIPN (5 mol %), dtbbpyNiCl_2_ (5 mol %), Et_3_N (2 equiv.), and K_3_PO_4_ (1 equiv.) in MeCN (0.1 m) under irradiation of 440 nm light at room temperature, under which the desired C(sp^2^)−C(sp^3^) coupling product **3 ja** was obtained in 85 % yield. A 3 mmol reaction was also conducted, affording **3 ja** in a 75 % isolated yield (entry 1). Control experiments showed that the dtbbpy ligand, NiCl_2_⋅glyme, 4‐CzIPN photocatalyst, light, and K_3_PO_4_ were all critical for this efficient transformation (entries 2–6). Light of 400 nm was less effective (entry 7). Another inorganic base Na_2_CO_3_ delivered a slightly lower product yield (entry 8). The replacement of K_3_PO_4_ with Et_3_N dramatically decreased the yield (entry 9). Interestingly, 2,6‐lutidine proved to be a suitable organic base in this transformation (entry 10), which could be potentially employed for homogeneous flow chemistry. The reaction also proceeded with other electron donors; while *N*,*N*‐diisopropylethylamine (DIPEA) afforded a clean transformation (entry 11), pentamethylpiperidine (PMP) gave a low yield of the desired product (entry 12). No aryl‐amino alkyl coupling product was formed despite the commonly observed reactivity of α‐amino radicals in dual Ni/photoredox catalysis,^[15]^ implying that the XAT process predominates over other pathways like interception of such radicals by Ni^II/I^.[^15b^, ^15c^] Recent studies have shown that the radical anions of 4‐CzIPN and its derivatives can be photoexcited to afford potent reductants that can reduce substrates with redox potentials more negative than −2.0 V.^[16]^ To understand whether the C−I bond cleavage of **2 a** in this reaction was driven by direct XAT or SET, a tertiary amine that is not able to generate an α‐amino radical was tested. No reaction was observed, a result evidencing against a direct SET reduction mechanism (entry 13). Other polar aprotic solvents were tested but were less effective (entries 14 and 15).

**Table 1:** Reaction optimization.^[a]^

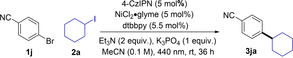		
Entry	Deviation from standard conditions	**3 ja** ^[b]^ [%]
1	none	85 (75)^[c]^
2	no NiCl_2_⋅glyme	0
3	no dtbby	0
4	no 4‐CzIPN	0
5	in the dark	0
6	no K_3_PO_4_	44
7	400 instead of 440 nm light	75
8	Na_2_CO_3_ (1 equiv.) instead K_3_PO_4_	78
9	Et_3_N (1 equiv.) instead of K_3_PO_4_	45
10	2,6‐lutidine (1 equiv.) instead of K_3_PO_4_	78
11	DIPEA (2 equiv.) instead of Et_3_N	83
12	PMP (2 equiv.) instead of Et_3_N	25
13	DABCO (2 equiv.) instead of Et_3_N	0
14	1,4‐dioxane as solvent	58
15	DMA as solvent	60
16	CyBr instead of CyI	trace
17	ArCl instead of ArBr	0
18	**2 a** as the limiting reactant^[c]^	93
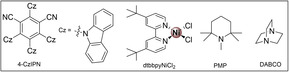		

[a] Reaction conditions A: **1 j** (0.20 mmol), **2 a** (0.30 mmol), 4‐CzIPN (0.01 mmol), NiCl_2_⋅glyme (0.01 mmol), dtbbpy (0.011 mmol), Et_3_N (0.6 mmol), K_3_PO_4_ (0.2 mmol), MeCN (2.0 mL), RT, 440 nm LED. [b] Yields of **1 ja** were determined by ^1^H NMR spectroscopy with 1,3,5‐trimethoxybenzene as an internal standard. [c] isolated yield of the reaction on a 3.0 mmol scale. [d] Reaction conditions B: **1 j** (0.3 mmol), **2 a** (0.2 mmol), 4‐CzIPN (0.01 mmol), NiCl_2_⋅glyme (0.01 mmol), dtbbpy (0.011 mmol), Et_3_N (0.4 mmol), K_3_PO_4_ (0.2 mmol), MeCN (2.0 mL), RT, 440 nm LED.

Other electrophiles were also evaluated. Bromocyclohexane failed to deliver the desired product (entry 16), consistent with previous reports where XAT on an unactivated secondary alkyl bromides was ineffective.[^10^, ^17^] 4‐Cyanophenyl chloride was unreactive under the standard conditions (entry 17), showcasing the excellent selectivity of C−Br over C−Cl bond activation and ruling out activity of *4‐CzIPN^.−^ known to reduce aryl chlorides,^[16a]^ presumably due to rapid turnover of 4‐CzIPN^.−^ by the in situ‐generated Ni^I^ complex. A general issue in dual Ni/photoredox catalysis is the use of excess radical precursors,^[18]^ which is detrimental in the late‐stage functionalizations of less available, complex molecule radical precursors. We questioned whether the alkyl iodide could be employed as the limiting reactant. Pleasingly, **2 a** as the limiting reactant provided **3 aa** in even higher yield (93 %, entry 18), which allows users of this method to flexibly choose either the aryl bromide or the alkyl iodide as the limiting reactant based on their complexities and production costs.

**Scheme 2:**
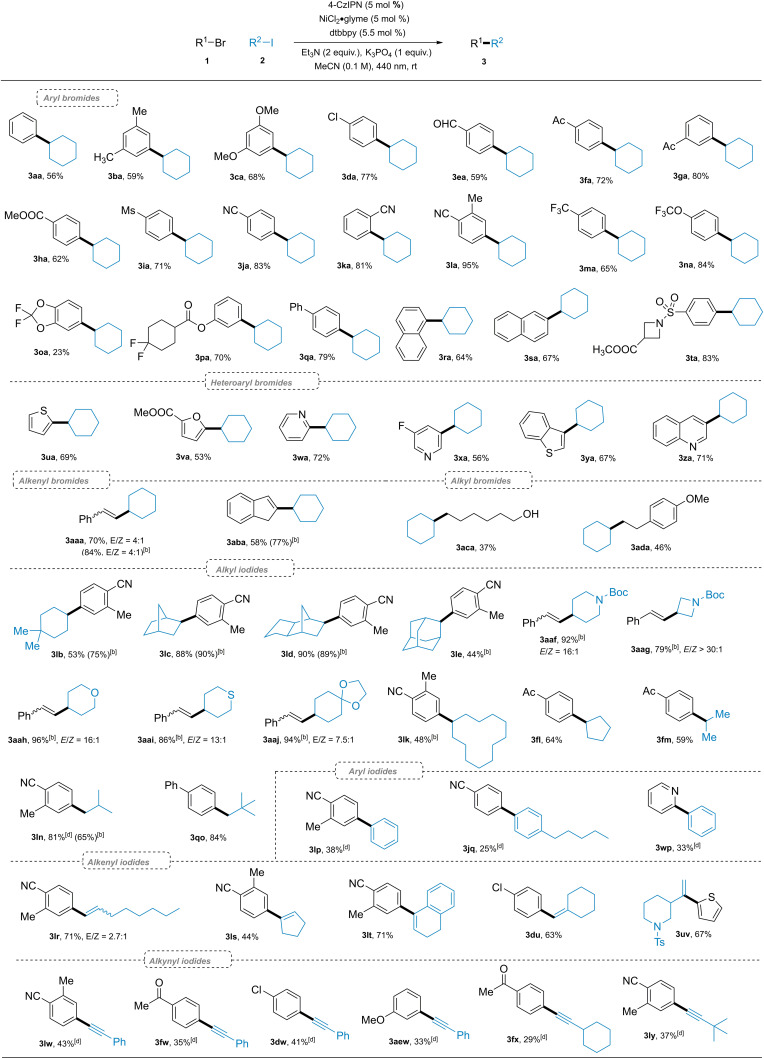
Reaction scope^[a,c]^. [a] Reaction conditions A: **1** (0.20 mmol), **2** (0.30 mmol), 4‐CzIPN (0.01 mmol), NiCl_2_⋅glyme (0.01 mmol), dtbbpy (0.011 mmol), Et_3_N (0.6 mmol), K_3_PO_4_ (0.2 mmol), MeCN (2.0 mL), RT, 440 nm LED. [b] Reaction conditions B: **1** (0.3 mmol), **2** (0.2 mmol), 4‐CzIPN (0.01 mmol), NiCl_2_⋅glyme (0.01 mmol), dtbbpy (0.011 mmol), Et_3_N (0.4 mmol), K_3_PO_4_ (0.2 mmol), MeCN (2.0 mL), RT, 440 nm LED. [c] Isolated yields were given. [d] 3 equiv. of organic iodide **2** was employed.

With the optimized conditions (Table 1, entry 1 or 18) in hand, we turned our attention to evaluate the reaction scope by employing either substrate **1** or **2** as the limiting reactant (Scheme 2). Beginning the investigation into the substrate scope with respect to aryl bromides, we found that this reaction was compatible with bromoarenes **1 a**–**1 d** bearing electron‐donating groups, providing **3 aa**–**3 da** all in good (59–77 %) yields, where 4‐chlorophenyl bromide underwent a selective C−Br alkylation leaving the chlorine atom untouched. A variety of electron‐withdrawing groups (CHO, Ac, COOCH_3_, Ms, CN, CF_3_) at different positions of the arene rings were well tolerated, the corresponding products **3 ea**–**3 ma** were obtained in good to excellent (59–95 %) yields. C−F bonds are widely prevalent in pharmaceutically‐relevant compounds,^[19]^ to our delight, bromoarenes possessing divergent C−F bonds were well tolerated by our system (**3 ma**–**3 pa**). Cyclohexylations of brominated larger π‐systems **3 q**–**3 s** also proceeded smoothly by this protocol. A complex azetidine‐based substrate gave the desired product **3 ta** in 83 % yield. We also applied this catalytic system to the cross couplings of cyclohexyl iodide with substrates containing other Csp^2^−Br bonds. Heteroaryl bromides **1 u**–**1 z** were suitable substrates for this reaction with thiophene, benzothiophene, furan, pyridine, and quinolone rings intact. Alkenyl bromides were efficiently coupled to deliver respective products (**3 aaa**, **3 aba**) in good to high (58–70 %) yields. Iodocyclohexane as limiting reactant afforded **3 aaa** in an even higher yield (84 %). Csp^2^−Csp^3^ bond formations were also successful, exemplified by the formations of **3 aca** and **3 ada** from unactivated alkyl bromides **1 ac** and **1 ad**.

Encouraged by the excellent performance of organic bromides, we attempted to further examine the scope with organic iodides. Hindered cyclohexyl iodides such as 4,4‐dimethyl‐1‐iodocyclohexane and 2‐iodonorbornane did not retard the reaction (products **3 lb**, **3 lc**). Notably, a tricyclic alkyl iodide gave rise to the expected product **3 ld** in an excellent (90 %) yield. 2‐Iodoadamantane substrate proved effective despite its high steric hindrance (**3 le**). Piperidine, azetidine, tetrahedropyran, tetrahedrothiopyran, and dioxolane containing iodides were efficiently converted into desired products **3 aaf**–**3 aaj** (79–96 % yields). Alkyl iodides derived from either a larger or a smaller aliphatic carbocycle provided products **3 lk** and **3 fl**, respectively, in good (48–64 %) yields. Open‐chain secondary and primary alkyl iodides were also competent substrates, affording **3 fm** and **3 ln**. 1‐Iodo‐2,2‐dimethylpropane **2 o** was surprisingly well‐tolerated (**3 qo**) in spite of its high steric hinderance, further highlighting the broad scope of alkyl iodides. We also attempted to utilize other organic iodides to achieve more C−C bond formations. Without further optimizations of the reaction conditions, the replacement of alkyl iodides with aryl iodides^[20]^ formed biaryl products **3 lp**, **3 jq**, and **3 wp** in low to moderate (25–38 %) yields, representing a novel reductive Suzuki‐like aryl‐aryl coupling reaction under mild conditions. Analogously, reductive Heck‐like couplings of aryl bromides and vinyl iodides were also realized. A diverse set of alkenyl iodides [including 1‐iodo‐1‐octene, 1‐iodocyclopentene, 4‐iodo‐1,2‐dihydronaphthalene, and (iodomethylene)cyclohexane] were efficiently arylated affording **3 lr**–**3 lt** and **3 du** in good yields (48–71 %). Biheterocyclic product **3 uv**, the core structure of a glucocorticoid receptor antagonist with a decreased hERG inhibition,^[21]^ was readily prepared by the coupling of thienyl bromide with the vinyl iodide. The substrate scope was successfully expanded to alkynyl iodides **2 w**–**2 y**, affording products in low to moderate yields (29–43 %) representing a new, net‐reductive Sonogashira‐like C(sp^2^)−C(sp) coupling reaction without a dual metallic catalyst system.

**Scheme 3:**
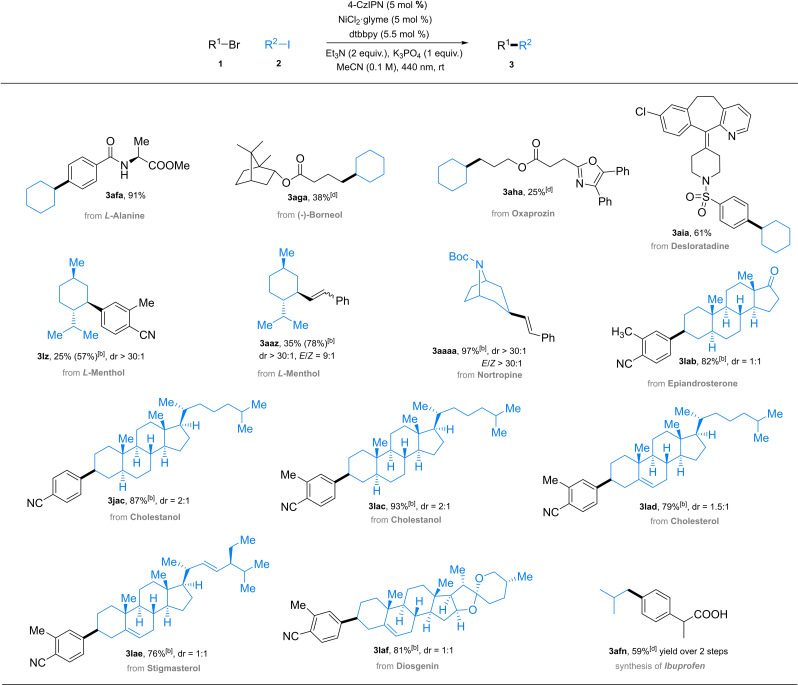
Late‐stage functionalizations of pharmaceutically relevant molecules and drug molecule synthesis^[a,c]^. [a] Reaction conditions A: **1** (0.20 mmol), **2** (0.30 mmol), 4‐CzIPN (0.01 mmol), NiCl_2_⋅glyme (0.01 mmol), dtbbpy (0.011 mmol), Et_3_N (0.6 mmol), K_3_PO_4_ (0.2 mmol), MeCN (2.0 mL), RT, 440 nm LED. [b] reaction conditions B: **1** (0.3 mmol), **2** (0.2 mmol), 4‐CzIPN (0.01 mmol), NiCl_2_⋅glyme (0.01 mmol), dtbbpy (0.011 mmol), Et_3_N (0.4 mmol), K_3_PO_4_ (0.2 mmol), MeCN (2.0 mL), RT, 440 nm LED. [c] Isolated yields were given. [d] 3 equiv. of organic iodide **2** was employed.

**Scheme 4:**
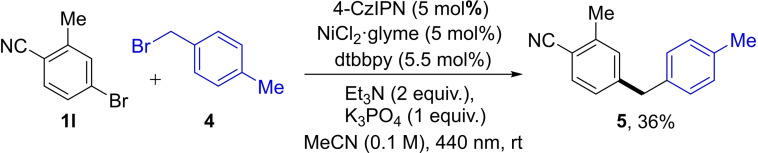
Coupling of two distinct organic bromides^[a,b]^. [a] Reaction conditions: **1 l** (0.20 mmol), **4** (0.60 mmol), 4‐CzIPN (0.01 mmol), NiCl_2_⋅glyme (0.01 mmol), dtbbpy (0.011 mmol), Et_3_N (1.2 mmol), K_3_PO_4_ (0.2 mmol), MeCN (2.0 mL), RT, 440 nm LED. [b] Isolated yield.

The applicability of this catalytic system to pharmaceutically‐relevant substrates and its ability to tolerate various functional groups is highlighted in Scheme 3. An exceptional advantage of our catalytic system compared to the state of art is that both organic bromide and iodide can be flexibly employed as the limiting reactant (Table 1, entries 1 and 18). Thus, an excess of cyclohexyl iodide can be employed for the functionalization of organic bromides that bear biologically important units. l‐Alanine was well tolerated in the Csp^2^−Csp^3^ coupling reaction, affording the desired product **3 afa** in 91 % yield. Borneol‐ and oxaprozin‐based alkyl bromides were also able to generate the Csp^2^−Csp^3^ coupling products. The successful functionalization of desloratadine is potentially beneficial to the discovery of antiallergic drugs. To further demonstrate the breath of this approach in the complementary direction, a series of alkyl iodides derived from biologically‐important compounds were tested. Highly sterically hindered alkyl iodide **2 z** from l‐menthol coupled with aryl bromide **1 l** and styryl bromide **1 aa**, delivering products **3 lz** and **3 aaz** in 57 and 78 % yields, respectively, with exclusive diastereoselectivity (>30 : 1). A nortropine derivative successfully afforded product **3 aaaa** in 97 % yield exclusively as the *E*‐isomer. Notably, a number of complex polycyclic alkyl iodides prepared from the corresponding steroidal natural products bearing olefins and spirocyclic ethers were smoothly tolerated affording arylated products **3 jac** and **3 lab**–**3 laf** in high (76–93 %) yields. As a common non‐steroidal anti‐inflammatory drug, ibuprofen was readily prepared through sequential metallaphotoredox‐catalyzed cross coupling and ester hydrolysis (59 % yield over two steps). Although the coupling of an unactivated alkyl bromide with aryl bromide **1 j** was unsuccessful (Table 1, entry 16), benzyl bromide **4** as radical precursor provided the aryl‐benzyl coupling product **5** in a satisfactory yield (Scheme 4). The results were consistent with a previous report in which benzyl bromide was successfully coupled in 40 % yield,^[10]^ where an unactivated secondary alkyl bromide was not included.

Finally, the potential to scale the reaction with continuous flow was confirmed (Table 2). K_3_PO_4_ was replaced with an organic base, 2,6‐lutidine, to achieve homogenous conditions. With iodocyclohexane (**2 a**) as the limiting reactant, full conversion of a 0.07 m solution of **2 a** was achieved in a single pass after 100 min, giving a 51 % yield of **3 ja**. Recirculating the reaction was found not to be beneficial (see the Supporting Information). Maintaining the same residence time and decreasing temperature to 25 °C marginally improved yield at the cost of conversion (entry 2). Increasing the flow rate maintained the yield, affording **3 ja** in 54 % and 0.8 g d^−1^ productivity (entry 3). Surprised that conversion was maintained in a shorter residence time, we postulated that photodecomposition of **2 a** under the high‐power LED irradiation (60 W input, 24 W radiant power) may explain this, arising lower yields compared to the batch reaction. Gratifyingly, when **1 j** was set as the limiting reactant the product yield increased (78 %, entry 4), showcasing the power of our method's ability to employ either partner as the limiting reagent. Doubling the flow rate afforded a slightly lower yield but almost doubled productivity (2.61 g d^−1^, entry 5). Compared to the batch reactions (0.28 g d^−1^), continuous flow accessed considerably higher (≈10×) productivities.

**Table 2:** Scale‐up using continuous flow.^[a]^

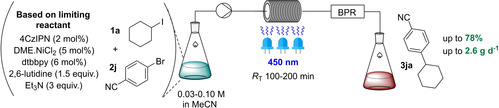							
Entry	Limiting reactant (mmol)	Concentration [m]	Flow rate [mL min^−1^]	*R*_T_ [min]	Conversion [%]	Yield of **3ja** [%]	Productivity^[b]^ [g d^−1^]
1^[c]^	**2 a** (0.7)	0.07	0.05	100	100	51	0.48
2	**2 a** (0.8)	0.07	0.05	100	89	54	0.50
3	**2 a** (1.2)	0.07	0.08	100	92	54	0.81
4	**1 j** (1.0)	0.07	0.10	100	100	78	1.45
5	**1 j** (1.0)	0.07	0.20	50	72	70	2.61

[a] Unless otherwise stated, reactions were conducted at a controlled 25 °C. [b] Productivity calculated assuming a single pass. [c] 30 °C.

## Conclusions

We report a metallaphotoredox catalyzed cross‐electrophile coupling of distinct organic halides leveraging the merger of nickel catalysis and an organophotocatalysis for a halogen atom transfer (XAT) strategy. Key to success was the use of cheap triethylamine as a precursor to α‐aminoalkyl radical halogen atom transfer agents. The reaction avoids stoichiometric transition metal salts and takes place under mild conditions, allowing the method to transform a broad scope of coupling partners into products with excellent chemo‐, regio‐, and diastereoselectivity. The method offers net‐reductive alternatives to Heck‐type, Suzuki‐type and Sonogashira‐type couplings, which are attractive since they do not require (i) prefunctionalization of organic halides as boron‐derived partners^[14]^ or (ii) dual metallic catalyst systems. A particularly attractive feature of the method is it can deliver products in high yields when employing either coupling partner as the limiting reactant, allowing user‐flexibility to accommodate and limit the organic halide partner with greatest cost or pharmaceutical importance. As demonstrated herein by the broad scope of applications in late‐stage functionalization, this method represents an attractive tool for synthetic and medicinal chemists to rapidly build molecular complexity and contributes to a rapidly developing field of XAT dual first‐row transition metal catalysis.[^11^, ^12^, ^22^, ^23^] Future challenges include expanding the scope of organic halide partners to aryl/alkyl chlorides without bulky silane XAT agents.

## Experimental Section

### Preparation of Ni complex stock solution

To an oven‐dried crimp cap vial (50 mL) equipped with a magnetic stirring bar was added NiCl_2_⋅glyme powder (44 mg, 0.2 mmol) and dtbbpy (58 mg, 0.22 mmol). Then the vial was sealed, degassed, and backfilled with N_2_ (3×), followed by the addition of 40 mL anhydrous, degassed MeCN under N_2_ via a syringe. The resulting mixture was bubbled with N_2_ for 10 min, placed into an oil bath, and stirred at 50 °C. The vial was moved out of the oil bath after particles were completely dissolved. The resulting homogeneous solution was stored in the dark ready for use.

### General procedure for photochemical cross‐electrophile couplings in batch

**Conditions A**: To an oven‐dried crimp cap vial (5 mL) equipped with a magnetic stirring bar was added 0.2 mmol substrate **1**, 4‐CzIPN catalyst (0.01 mmol, 0.05 equiv.), K_3_PO_4_ (0.2 mmol, 1.0 equiv.), and substrate **2** (0.3 or 0.6 mmol) or **4**. The reaction vial was sealed, degassed, and backfilled with N_2_. 2 mL of dtbbpyNiCl_2_ solution (see the Supporting Information for preparation) was added under N_2_ via a syringe. The resulting mixture was bubbled with N_2_ for another 10 min and degassed Et_3_N (2 equiv. based on substrate **2**) was added. The vial was placed into a water‐cooled cooling block, stirred, and irradiated (through the bottom of the reaction vial) with a 440 nm LED for 36 h. Then, the reaction mixture was transferred into a round‐bottom flask and evaporated under reduced pressure. The residue was purified by column chromatography on silica gel using pentane or an EtOAc/pentane mixture as eluent to afford pure product **3** or **5**.

**Conditions B**: To an oven‐dried crimp cap vial equipped with a magnetic stirring bar was added 0.2 mmol substrate **2**, 4‐CzIPN catalyst (0.01 mmol, 0.05 equiv.), K_3_PO_4_ (0.2 mmol, 1.0 equiv.), and substrate **1** (0.3 mmol, 1.5 equiv.). Then the reaction vial was sealed, degassed, and backfilled with N_2_. 2 mL dtbbpy⋅NiCl_2_ (5 mol %) solution (see the Supporting Information for preparation) was added under N_2_ via a syringe. The resulting mixture was bubbled with N_2_ for another 10 min and degassed Et_3_N (0.4 mmol, 2.0 equiv.) was added. The vial was placed into a water‐cooled cooling block, stirred, and irradiated through the bottom of the reaction vial with a 440 nm LED for 36 h. Workup and purification followed the aforementioned procedure in “Conditions A”.

### General procedure for photochemical cross‐electrophile couplings in flow

To an oven‐dried crimp cap vial (50 mL) equipped with a magnetic stirrer bar was added substrate **2 a** and 4CzIPN (5 mol % based on limiting substrate). The reaction vial was sealed, degassed, and backfilled with N_2_. 2,6‐Lutidine, substrate **1 j**, and MeCN were added. The resulting mixture was bubbled with N_2_ for 10 min. Afterwards, dtbbpy⋅NiCl_2_ solution (5 mol % based on limiting substrate) was added under N_2_ via a syringe. The resulting mixture was bubbled with N_2_ for another 10 min and degassed Et_3_N (2.0 equiv. based on limiting substrate) was added. A Vapourtec UV‐150 Photochemical Reactor (R Series) was first primed with anhydrous MeCN (20 mL) under an N_2_ atmosphere, and then the reaction mixture was introduced and exposed to conditions described in Table S1 within the 10 mL tubular reactor under 450 nm irradiation (60 W input power, 24 W radiant power) and collected in another closed crimp cap vial. The temperature of the coil was controlled precisely (±1 °C) by a regulated stream of N_2_ cooled through a canister filled with dry ice) or heated by convection with an in‐built electronic heating gun. After collection of the whole reaction mixture, the reaction mixture was transferred into a round‐bottom flask and a known amount of 1,3,5‐trimethoxybenzene was added as an internal standard for ^1^H NMR yield calculation. Solvent was then evaporated under reduced pressure and the residue was passed through a silica gel plug using 25 % EtOAc in pentane (100 mL) as eluent, to give the crude product **3 ja**, which was quantified by ^1^H NMR spectroscopy.

## Conflict of interest

The authors declare no conflict of interest.

1

## Supporting information

As a service to our authors and readers, this journal provides supporting information supplied by the authors. Such materials are peer reviewed and may be re‐organized for online delivery, but are not copy‐edited or typeset. Technical support issues arising from supporting information (other than missing files) should be addressed to the authors.

Supporting Information

## Data Availability

The data that support the findings of this study are available in the supplementary material of this article.
