# Supplementary material for: α‐Amino Radical Halogen Atom Transfer Agents for Metallaphotoredox‐Catalyzed Cross‐Electrophile Couplings of Distinct Organic Halides
Source: ChemSusChem. 2022 Jun 13;15(15):e202200906. doi: 10.1002/cssc.202200906 (PMC9541218; doi:10.1002/cssc.202200906)
Supplement: Supplementary file 1 — Supporting Information [file CSSC-15-0-s001.pdf]

ChemSusChem

Supporting Information

α-Amino Radical Halogen Atom Transfer Agents for Metallaphotoredox-Catalyzed Cross-Electrophile Couplings of Distinct Organic Halides

Xianhai Tian, Jaspreet Kaur, Shahboz Yakubov, and Joshua P. Barham*© 2022 The Authors. ChemSusChem published by Wiley-VCH GmbH. This is an open access article under the terms of the Creative Commons Attribution License, which permits use, distribution and reproduction in any medium, provided the original work is properly cited.

SUPPORTING INFORMATION FILE

α-AMINO RADICAL HALOGEN ATOM TRANSFER AGENTS FOR METALLAPHOTOREDOX CATALYZED CROSS-ELECTROPHILE COUPLINGS OF DISTINCT ORGANIC HALIDES

Xianhai Tian, Jaspreet Kaur, Shahboz Yakubov, Joshua P. Barham*

Universität Regensburg, Institut für Organische Chemie, Fakultät für Chemie und Pharmazie, 93040 Regensburg, Germany

*Corresponding Author. E-mail: Joshua-Philip.Barham@chemie.uni-regensburg.de

TABLE OF CONTENTS

1. General Experimental Information 1
2. Materials 2
3. Synthetic Procedures 2
4. Characterization Data 5
5. Continuous Flow Experiments 36
6. ^{1}H NMR and ^{13}C NMR Spectra 38
7. References 125

1. GENERAL EXPERIMENTAL INFORMATION

Unless stated otherwise, reactions were carried out under an inert (N_2) atmosphere. Cryogenic conditions (-78 °C) were achieved using dry ice/acetone baths. Temperatures of 0 °C were obtained by means of an ice bath or ice/salt bath. 'Room temperature' (rt) indicates temperatures in the range of 20-25 °C. For purposes of thin layer chromatography (TLC), ALUGRAM® Xtra SIL G/UV_{254} silica plates were used, with UV light ($\lambda = 254$ nm), near-UV light ($\lambda = 366$ nm) and potassium permanganate used for visualisation. Purification was achieved by column chromatography using Macherey-Nagel silica gel 60 (0.063-0.2 mm) or Merck silica gel 60 (0.040-0.063 mm, 230-440 mesh). Removal of solvents (in vacuo) was achieved using Heidolph rotary evaporators or Vacuubrand high vacuum pumps.

All NMR data were collected using a Bruker Avance 400 Ultrashield instrument (400 MHz, 376 MHz, 162 MHz and 101 MHz for ^{1}H, ^{19}F and ^{13}C NMR), or a Bruker Avance 300 Ultrashield instrument (300 MHz, 282 MHz, 162 MHz and 75 MHz for ^{1}H, ^{19}F and ^{13}C NMR) was used. ^{13}C NMR was run in ^{1}H-decoupled mode. Data were manipulated using MestReNova version 12.0.0. Multiplicities for coupled signals were denoted as: s = singlet, d = doublet, t = triplet, q = quartet, quint = quintet, sext = sextet, hept = heptet, dd = doublet of doublets, ddd = doublet of doublets of doublets, td = triplet of doublets, qd = quartet of doublets, m = multiplet, br. = broad, apt. = apparent. Coupling constants (*J*) are given in Hz and are uncorrected. Where appropriate, COSY, DEPT, HSQC and HMBC experiments were carried out to aid assignment.

High Resolution Mass spectral analyses were carried out in EI or ESI mode on a Finnigan MAT 95, Thermo Quest Finnigan TSQ 7000, Finnigan MATSSQ 710 A or an Agilent Q - TOF 6540 UHD instrument, masses observed are accurate to within ±5 ppm. Melting points are uncorrected and were recorded using a Stuart melting point device up to 300 °C. All anhydrous solvents were purchased from Sigma Aldrich or Fisher Scientific, dried over 4Å molecular sieves. All other solvents and reagents were used as supplied or purified using standard techniques. Reactants and reagents were purchased at the highest commercial quality and used as received, from TCI, Sigma-Aldrich, Fischer Scientific, Fluorochem or ChemPur.

2. Materials

LED details: 440 nm: OSRAM Oslon (Oslon SSL 80 LDCQ70-2U3U LT1960). Input power per LED is 3.3 W, luminous flux is 1.5 W @ 1000 mA. Spectra were measured by a fibre-optic probe (BWTEK Inc.) at a distance of 30 cm directly above the LED and are shown below in Figure S1. Two batches of LEDs were used with similar emission profiles.

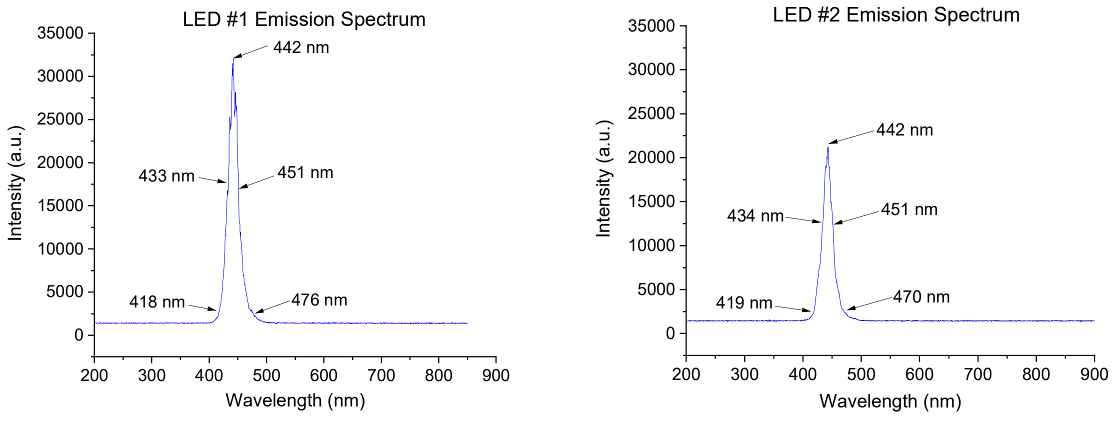

Figure S1. LED Emission Spectra.

3. Synthetic Procedures

Procedure 1: Synthesis of complex aryl bromides **1**

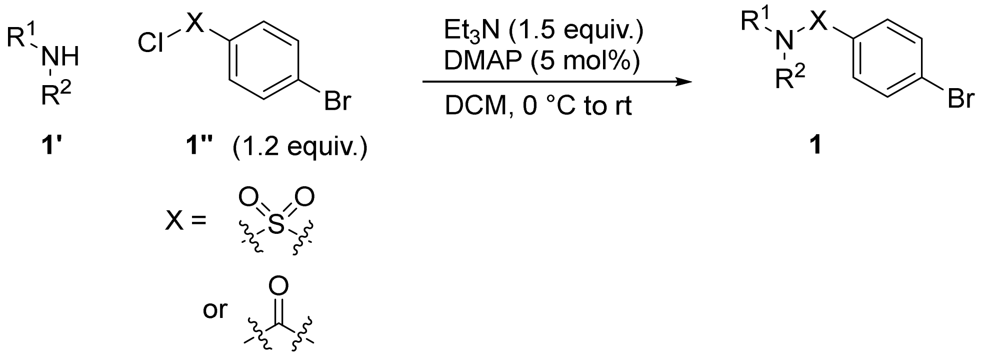

At 0 °C, to a mixture of **1'** (5.0 mmol, 1.0 equiv.) and Et_3N (7.5 mmol, 1.5 equiv.) in 30 mL anhydrous DCM was added **1''** (6.0 mmol, 1.2 equiv.) followed by the addition of DMAP (0.25 mmol, 0.05 equiv.). The reaction mixture was stirred at 0 °C for 1 h before allowing to warm to rt. After being stirred at rt overnight, 30 mL 5% $NaHCO_3$ aqueous solution was added and the resulting mixture was transferred into a separation funnel, separated. The organic phase was washed with 30 mL saturated brine, dried over Na_2SO_4 and evaporated. The residue was purified by flash column chromatography using a mixture of pentane and EtOAc to afford the desired product **1** in high yield.

Procedure 2: Synthesis of iodoalkanes **2** from alcohols **2'**

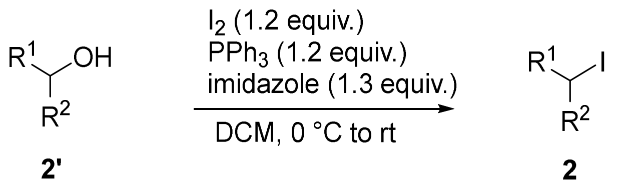

At 0 °C, to a mixture of alcohol **2'** (5.0 mmol, 1.0 equiv.), PPh_3 (6.0 mmol, 1.2 equiv.) and imidazole (6.5 mmol, 1.3 equiv.) in 40 mL anhydrous DCM was added I_2 (6.0 mmol, 1.2 equiv.) in three portions. The reaction mixture was stirred at 0 °C for 1 h before allowing to warm to rt. After being stirred at rt for 4 h, the reaction mixture was transferred into a separation funnel, washed with 40 mL 5% $Na_2S_2O_3$ aqueous solution and 40 mL saturated brine. The organic phase was dried over Na_2SO_4 and evaporated. The residue was purified by flash column chromatography using pentane or a mixture of pentane and EtOAc to give the desired alkyl iodide **2** in high yield.

Procedure 3: Synthesis of alkenyl iodides **2** from ketones **2''**

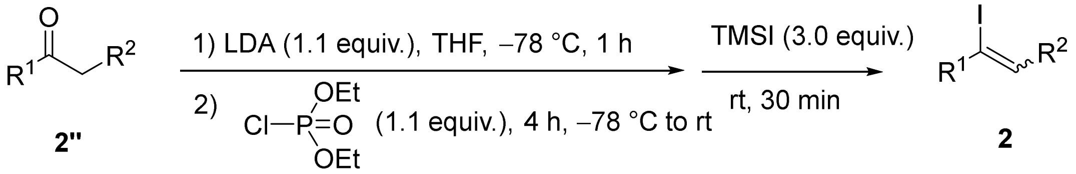

Prepared according to a literature procedure.[1] At -78 °C, to a solution of ketone **2''** (5.0 mmol, 1.0 equiv.) in 30 mL anhydrous THF was added LDA (1.0 M in THF/hexanes) (5.5 mmol, 1.1 equiv.) dropwise. The reaction mixture was stirred at -78 °C for 1 h followed by the addition of diethyl chlorophosphate (5.5 mmol, 1.1 equiv.). The resulting mixture was allowed to warm to rt and stirred for 4 h. Then TMSI (15.0 mmol, 3.0 equiv.) was added dropwise and the resulting mixture was stirred at rt for 30 min followed by additions of 20 mL saturated $NaHCO_3$ and 20 mL 5% $Na_2S_2O_3$ solutions. The organic layer was separated and the aqueous layer was extracted with Et_2O (40 mL × 2). The organic layers were combined, dried over Na_2SO_4 and evaporated. The residue was purified by flash column chromatography using pentane or a mixture of pentane and EtOAc to give the desired alkenyl iodide **2** in good yield.

Procedure 4: Preparation of the dtbbpy·$NiCl_2$ stock solution (0.005 M)

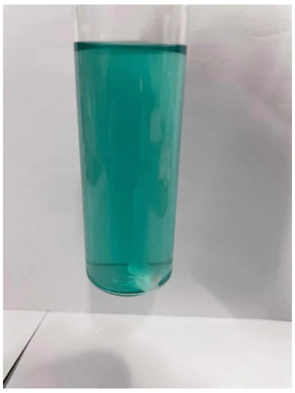

To an oven-dried crimp cap vial (50 mL in volume) equipped with a magnetic stirring bar was added with $NiCl_2$·glyme powder (44 mg, 0.2 mmol), dtbbpy (4,4'-di-*tert*-butyl-2,2'-dipyridyl) (58 mg, 0.22 mmol). Then the vial was sealed, degassed and backfilled with N_2 (3×), followed by the addition of 40 mL anhydrous degassed MeCN under N_2 via a syringe. The resulting mixture was bubbled with N_2 for 10 min, placed into an oil bath and heated to 50 °C under stirring. The vial was moved out of the oil bath until the powders were completely dissolved. The resulting homogenous solution was stored in the dark and ready for use.

Figure S2. Picture of the prepared Ni complex solution.

Procedure 5: Photochemical cross-electrophile couplings (organic bromide **1** as limiting reactant) 'Reaction Conditions A' (in main manuscript)

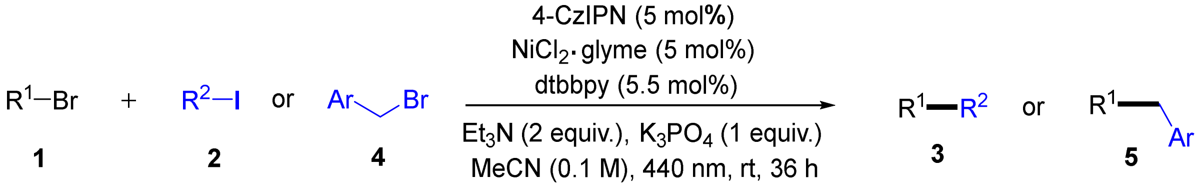

To an oven-dried crimp cap vial (5 mL in volume) equipped with a magnetic stirring bar was added 0.2 mmol substrate **1**, 4-CzIPN (0.01 mmol, 0.05 equiv.), K_3PO_4 (0.2 mmol, 1.0 equiv.) and either substrate **2** (0.3 mmol) or **4** (0.6 mmol). Then the reaction vial was sealed, degassed and backfilled with N_2. 2 mL of the dtbbpy·$NiCl_2$ solution was added under N_2 via a syringe. The resulting mixture was bubbled with N_2 for another 10 min and degassed Et_3N was added (2 equiv. based on substrate **2**). The vial was placed into a water-cooled cooling block, stirred and irradiated (through the bottom of the reaction vial) with a 440 nm LED for 36 h. Then the reaction mixture was transferred to a round-bottom flask and evaporated under reduced pressure. The residue was purified by column chromatography on silica gel using pentane or a mixture of pentane and EtOAc as eluent to afford pure product **3** or **5**.

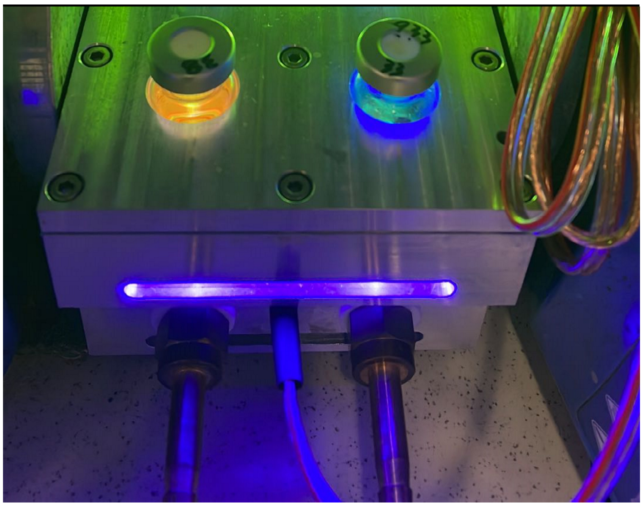

Figure S3. Picture of the photochemical reaction setup.

Procedure 6: Photochemical cross-electrophile couplings (organic iodide **2** as limiting reactant)

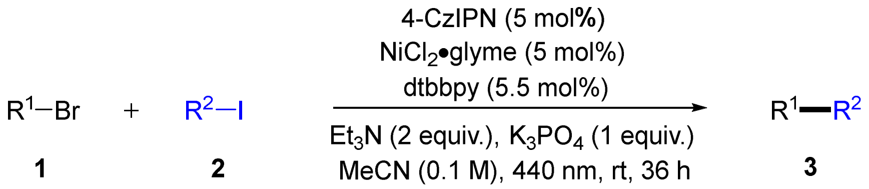

To an oven-dried crimp cap vial equipped with a magnetic stirring bar was added 0.2 mmol substrate **2**, 4-CzIPN (0.01 mmol, 0.05 equiv.), K_3PO_4 (0.2 mmol, 1.0 equiv.) and substrate **1** (0.3 mmol, 1.5 equiv.). Then the reaction vial was sealed, degassed and backfilled with N_2. 2 mL of the dtbbpy·$NiCl_2$ (5 mol%) solution was added under N_2 via a syringe. The resulting mixture was bubbled with N_2 for another 10 min and was then added degassed Et_3N (0.4 mmol, 2.0 equiv.). The vial was placed into a water-cooled cooling block, stirred and irradiated through the bottom of the reaction vial a 440 nm LED for 36 h. Then the reaction mixture was transferred into a round-bottom flask and evaporated under reduced pressure. The residue was purified by column chromatography on silica gel using pentane or a mixture of pentane and EtOAc as eluent to afford pure product **3**.

4. Characterization Data

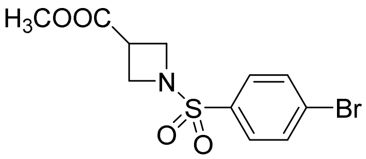

methyl 1-((4-bromophenyl)sulfonyl)azetidine-3-carboxylate (1t)

According to **Procedure 1**. Yield: 1.65 g, 99%; white solid, m.p. 78 °C; ^{1}H NMR (400 MHz, $CDCl_3$) δ 7.75-7.67 (m, 4H), 4.05-3.97 (m, 2H), 3.95-3.88 (m, 2H), 3.64 (s, 3H), 3.33-3.23 (m, 1H) ppm; ^{13}C NMR (100 MHz, $CDCl_3$) δ 171.6, 133.9, 132.7, 129.9, 128.7, 53.1, 52.6, 31.7 ppm; HRMS (ESI) (m/z) $[M+H]^+$ $C_{11}H_{13}{}^{81}BrNO_4S$ calcd. for 335.9723, found 335.9726.

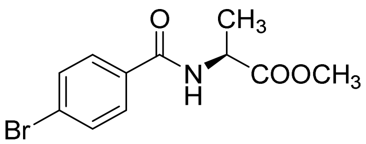

methyl (4-bromobenzoyl)-*L*-alaninate (1af)

According to **Procedure 1**. Yield: 53 mg, 82%; white solid, m.p. 105-106 °C; ^{1}H NMR (400 MHz, $CDCl_3$) δ 7.70-7.62 (m, 2H), 7.58-7.52 (m, 2H), 6.79 (d, J = 5.6 Hz, 1H), 4.83-4.73 (m, 1H), 3.78 (s, 3H), 1.51 (d, J = 7.2 Hz, 3H) ppm; ^{13}C NMR (100 MHz, $CDCl_3$) δ 173.6, 165.9, 132.7, 131.8, 128.7, 126.5, 52.7, 48.6, 18.6 ppm; HRMS (ESI) (m/z) $[M+H]^+$ $C_{11}H_{13}{}^{79}BrNO_3S$ calcd. for 286.0073, found 286.0075.

Data are consistent with the literature.[2]

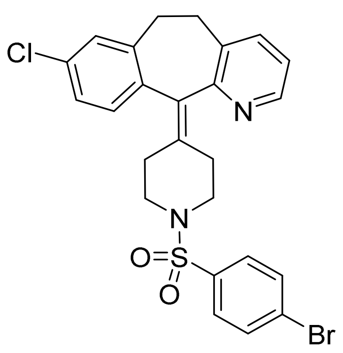

11-(1-((4-bromophenyl)sulfonyl)piperidin-4-ylidene)-8-chloro-6,11-dihydro-5*H*-benzo[5,6]cyclohepta[1,2-*b*]pyridine (1ai)

According to **Procedure 1**. Yield: 2.36 g, 89%; white solid, m.p. 202-203 °C; ^{1}H NMR (400 MHz, $CDCl_3$) δ 8.36 (d, *J* = 4.4 Hz, 1H), 7.68-7.63 (m, 2H), 7.62-7.58 (m, 2H), 7.40 (d, *J* = 8.0 Hz, 1H), 7.13 (d, *J* = 1.2 Hz, 1H), 7.12-7.05 (m, 2H), 7.04 (d, *J* = 8.0 Hz, 1H), 3.34-3.18 (m, 4H), 2.98-2.88 (m, 2H), 2.84-2.69 (m, 2H), 2.66-2.58 (m, 1H), 2.53-2.44 (m, 1H), 2.40-2.30 (m, 2H) ppm; ^{13}C NMR (100 MHz, $CDCl_3$) δ 156.6, 146.8, 139.7, 137.8, 137.4, 135.6, 135.5, 135.2, 133.4, 133.3, 132.5, 130.4, 129.2, 129.1, 128.0, 126.3, 122.5, 47.5 (one carbon was overlapped), 31.7, 31.6, 30.3, 30.0 ppm; HRMS (ESI) (m/z) $[M+H]^+$ $C_{25}H_{23}{}^{79}Br^{35}ClN_2O_2S$ calcd. for 529.0347, found 529.0350.

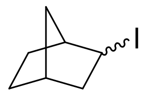

(1*S*,4*R*)-2-iodobicyclo[2.2.1]heptane (2c)

According to **Procedure 2**. Yield: 721 mg, 65%; colorless oil; as a mixture of isomers: *endo*/*exo* = 3.5/1; ^{1}H NMR (400 MHz, $CDCl_3$) δ 4.29-4.22 (m, 0.22H, the *exo* isomer), 4.01-3.95 (m, 0.78H, the *endo* isomer), 2.64-2.58 (m, 0.78H, the *endo* isomer), 2.43-2.38 (m, 0.22H, the *exo* isomer), 2.31-1.80 (m, 4H), 1.57-1.45 (m, 2H), 1.43-1.32 (m, 1H), 1.31-1.05 (m, 2H) ppm; ^{13}C NMR (100 MHz, $CDCl_3$) δ 48.2 (the *endo* isomer), 45.4 (the *endo* isomer), 44.9 (the *exo* isomer), 43.5 (the *exo* isomer), 38.2 (the *endo* isomer), 37.1 (the *exo* isomer), 36.5 (the *endo* isomer), 36.4 (the *exo* isomer), 32.6 (the *exo* isomer), 30.5 (the *endo* isomer), 29.7 (the *exo* isomer), 28.9 (the *endo* isomer), 28.64 (the *exo* isomer), 28.59 (the *endo* isomer) ppm; HRMS (EI) (m/z) [M] $C_7H_{11}I$ calcd. for 221.9905, found 221.9902.

Data are consistent with the literature.[3]

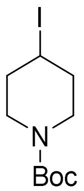

tert-butyl 4-iodopiperidine-1-carboxylate (2f)

According to **Procedure 2**. Yield: 1.50 g, 90%; colorless crystal, m.p. 47 °C; ^{1}H NMR (400 MHz, $CDCl_3$) δ 4.42 (quint, *J* = 6.0 Hz, 1H), 3.60-3.50 (m, 2H), 3.29-3.21 (m, 2H), 2.03-1.95 (m, 4H), 1.42 (s, 9H) ppm; ^{13}C NMR (100 MHz, $CDCl_3$) δ 154.7, 79.8, 43.7, 37.4, 28.5, 27.8 ppm; HRMS (ESI) (m/z) $[M+Na]^+$ $C_{10}H_{18}INaNO_2$ calcd. for 334.0274, found 334.0276.

Data are consistent with the literature.[4]

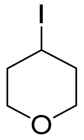

4-iodotetrahydro-2H-pyran (2h)

According to **Procedure 2**. Yield: 597 mg, 57%; colorless oil; ^{1}H NMR (400 MHz, CD_3CN) δ 4.50-4.39 (m, 1H), 3.85-3.75 (m, 2H), 3.57-3.47 (m, 2H), 2.21-2.08 (m, 4H) ppm; ^{13}C NMR (100 MHz, CD_3CN) δ 68.0, 38.9, 25.4 ppm; HRMS (EI) (m/z) [M] C_5H_9OI calcd. for 211.9698, found 211.9696.

Data are consistent with the literature.[5]

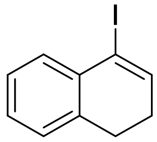

4-iodo-1,2-dihydronaphthalene (2t)

According to **Procedure 3**. Yield: 1.71 g, 66%; yellow oil; ^{1}H NMR (400 MHz, $CDCl_3$) δ 7.50-7.42 (m, 1H), 7.30-7.16 (m, 2H), 7.07-7.00 (m, 1H), 6.88-6.82 (m, 1H), 2.87 (d, *J* = 7.6 Hz, 2H), 2.42-2.33 (m, 2H) ppm; ^{13}C NMR (100 MHz, $CDCl_3$) δ 140.2, 135.9, 134.4, 130.8, 128.4, 127.3, 127.1, 98.1, 27.9, 27.2 ppm; HRMS (EI) (m/z) [M] $C_{10}H_9I$ calcd. for 255.9743, found 255.9745.

Data are consistent with the literature.[6]

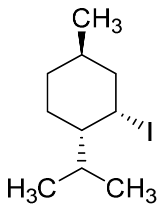

(1*S*,2*S*,4*R*)-2-iodo-1-isopropyl-4-methylcyclohexane (2z)

According to **Procedure 2**. Yield: 1.53 g, 91%; colorless oil; ^{1}H NMR (400 MHz, $CDCl_3$) δ 4.79-4.75 (m, 1H), 2.25-2.16 (m, 1H), 2.04-1.91 (m, 1H), 1.80-1.68 (m, 2H), 1.41-1.17 (m, 3H), 1.05-0.85 (m, 11H)

ppm; ^{13}C NMR (100 MHz, $CDCl_3$) δ 49.5, 46.8, 45.5, 35.0, 34.0, 28.8, 26.9, 21.8, 20.5, 20.1 ppm; HRMS (EI+) (m/z) $[M-I]^+$ $C_{10}H_{19}$ calcd. for 139.1481, found 139.1481.

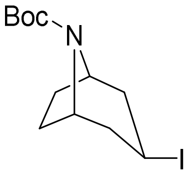

***tert*-butyl (1*R*,3*s*,5*S*)-3-iodo-8-azabicyclo[3.2.1]octane-8-carboxylate (2aa)**

According to **Procedure 2**. Yield: 1.49 g, 83%; colorless crystal, m.p. 99 °C; ^1H NMR (400 MHz, $CDCl_3$) δ 4.57-4.45 (m, 1H), 4.15-3.90 (m, 2H), 2.42-2.25 (m, 2H), 2.22-2.12 (m, 2H), 1.97-1.84 (m, 2H), 1.67-1.58 (m, 2H), 1.47 (s, 9H) ppm; ^{13}C NMR (100 MHz, $CDCl_3$) δ 153.3, 79.8, 56.3, 55.7, 45.5, 44.7, 28.6, 27.9, 27.2, 18.7 ppm; HRMS (ESI) (m/z) $[M+Na]^+$ $C_{12}H_{20}INaNO_2$ calcd. for 360.0431, found 360.0430.

Data are consistent with the literature.[7]

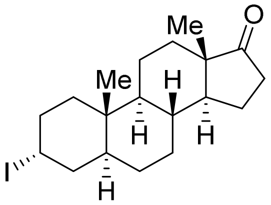

(3*R*,5*S*,8*R*,9*S*,10*S*,13*S*,14*S*)-3-iodo-10,13-dimethylhexadecahydro-17*H*-cyclopenta[*a*]phenanthren-17-one (2ab)

According to **Procedure 2**. Yield: 1.94 g, 97%; white solid, m.p. 124-125 °C; ^1H NMR (400 MHz, $CDCl_3$) δ 4.94-4.88 (m, 1H), 2.46-2.36 (m, 1H), 2.10-1.40 (m, 15H), 1.34-1.21 (m, 5H), 1.12-1.00 (m, 1H), 0.84 (s, 3H), 0.80 (s, 3H) ppm; ^{13}C NMR (100 MHz, $CDCl_3$) δ 221.3, 54.0, 51.5, 47.9, 42.1, 38.8, 37.7, 36.8, 35.9, 35.1, 34.4, 32.7, 31.6, 30.7, 27.5, 21.8, 20.2, 13.9, 13.5 ppm; HRMS (EI) (m/z) [M] $C_{19}H_{29}IO$ calcd. for 400.1263, found 400.1261.

Data are consistent with the literature.[8]

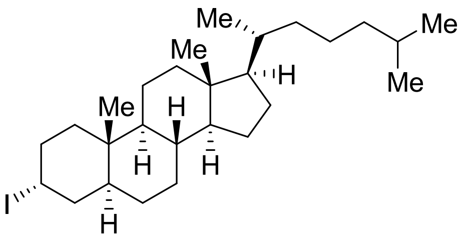

(3*R*,5*S*,8*R*,9*S*,10*S*,13*R*,14*S*,17*R*)-3-iodo-10,13-dimethyl-17-((*R*)-6-methylheptan-2-yl)hexadecahydro-1H-cyclopenta[*a*]phenanthrene (2ac)

According to **Procedure 2**. Yield: 2.46 g, 99%; white solid, m.p. 116-117 °C; ^1H NMR (400 MHz, $CDCl_3$) δ 4.97-4.90 (m, 1H), 2.00-1.00 (m, 31H), 0.91 (d, J = 6.8 Hz, 3H), 0.87 (dd, J = 6.4, 1.6 Hz, 6H), 0.79

(s, 3H), 0.65 (s, 3H) ppm; ^{13}C NMR (100 MHz, $CDCl_3$) δ 56.6, 56.4, 54.0, 42.7, 42.2, 40.1, 39.7, 38.9, 38.4, 36.7, 36.3, 36.0, 35.6, 34.5, 32.9, 31.9, 28.4, 28.2, 27.9, 24.3, 24.0, 23.0, 22.7, 21.0, 18.8, 13.5, 12.2 ppm; HRMS (ESI) (m/z) $[M-I]^+$ $C_{27}H_{47}$ calcd. for 371.3672, found 371.3678.

Data are consistent with the literature.[9]

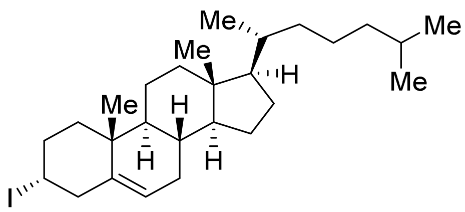

(3*R*,8*S*,9*S*,10*R*,13*R*,14*S*,17*R*)-3-iodo-10,13-dimethyl-17-((*R*)-6-methylheptan-2-yl)-2,3,4,7,8,9,10,11,12,13,14,15,16,17-tetradecahydro-1*H*-cyclopenta[*a*]phenanthrene (2ad)

According to **Procedure 2**. Yield: 2.28 g, 92%; white solid, m.p. 103 °C; ^{1}H NMR (400 MHz, $CDCl_3$) δ 5.35-5.31 (m, 1H), 4.09-3.99 (m, 1H), 2.98-2.86 (m, 1H), 2.72-2.63 (m, 1H), 2.34-2.14 (m, 2H), 2.04-1.91 (m, 2H), 1.88-1.77 (m, 1H), 1.76-1.69 (m, 1H), 1.62-1.06 (m, 17H), 1.04 (s, 3H), 1.02-0.93 (m, 3H), 0.91 (d, J = 6.8 Hz, 3H), 0.87 (d, J = 6.4 Hz, 6H), 0.86 (d, J = 6.4 Hz, 3H) ppm; ^{13}C NMR (100 MHz, $CDCl_3$) δ 142.9, 121.8, 56.9, 56.3, 50.5, 46.6, 42.5, 42.1, 39.8, 39.7, 36.8, 36.6, 36.3, 35.9, 31.9, 31.8, 30.7, 28.4, 28.2, 24.4, 24.0, 23.0, 22.7, 20.9, 19.4, 18.9, 12.0 ppm; HRMS (EI) (m/z) [M] $C_{27}H_{45}I$ calcd. for 496.2566, found 496.2565.

Data are consistent with the literature.[10]

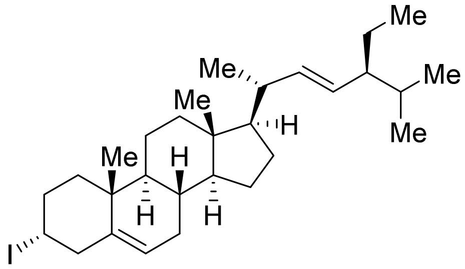

(3*R*,8*S*,9*S*,10*R*,13*R*,14*S*,17*R*)-17-((2*R*,5*S*,*E*)-5-ethyl-6-methylhept-3-en-2-yl)-3-iodo-10,13-dimethyl-2,3,4,7,8,9,10,11,12,13,14,15,16,17-tetradecahydro-1*H*-cyclopenta[*a*]phenanthrene (2ae)

According to **Procedure 2**. Yield: 2.42 g, 93%; white solid, m.p. 102-103 °C; ^{1}H NMR (400 MHz, $CDCl_3$) δ 5.36-5.30 (m, 1H), 5.15 (dd, J = 15.6, 8.8 Hz, 1H), 5.02 (dd, J = 15.2, 8.8 Hz, 1H), 4.09-3.99 (m, 1H), 2.98-2.87 (m, 1H), 2.72-2.63 (m, 1H), 2.33-2.13 (m, 2H), 2.08-1.91 (m, 3H), 1.77-1.65 (m, 2H), 1.60-1.51 (m, 3H), 1.50-1.35 (m, 5H), 1.32-0.91 (m, 14H), 0.87-0.77 (m, 9H), 0.69 (s, 3H) ppm; ^{13}C NMR (100 MHz, $CDCl_3$) δ 142.9, 138.4, 129.5, 121.8, 57.0, 56.1, 51.4, 50.5, 46.6, 42.4, 42.1, 40.6, 39.7, 36.8, 36.6, 32.0, 31.9, 31.8, 30.7, 29.0, 25.6, 24.5, 21.4, 21.3, 20.9, 19.4, 19.1, 12.4, 12.2 ppm; HRMS (ESI) (m/z) $[M+H]^+$ $C_{29}H_{47}I$ calcd. for 522.2722, found 522.2275.

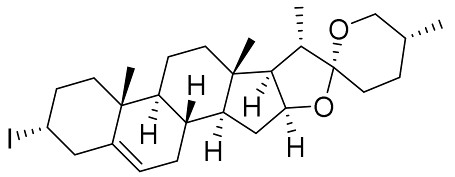

(4*R*,5'*R*,6*aR*,6*bS*,8*aS*,8*bR*,9*S*,10*R*,11*aS*,12*aS*,12*bS*)-4-iodo-5',6*a*,8*a*,9-tetramethyl-1,3,3',4,4',5,5',6,6*a*,6b,6',7,8,8*a*,8*b*,9,11*a*,12,12*a*,12*b*-icosahydrospiro[naphtho[2',1':4,5]indeno[2,1-*b*]furan-10,2'-pyran] (2af)

According to **Procedure 2**. Yield: 2.57 g, 98%; white solid, m.p. 161-162 °C; ^{1}H NMR (400 MHz, $CDCl_3$) δ 5.33 (s, 1H), 4.45-4.36 (m, 1H), 4.09-3.90 (m, 1H), 3.52-3.25 (m, 2H), 3.00-2.84 (m, 1H), 2.73-2.60 (m, 1H), 2.34-2.12 (m, 2H), 2.04-1.92 (m, 2H), 1.90-1.82 (m, 1H), 1.79-1.36 (m, 12H), 1.33-1.22 (m, 1H), 1.09-1.01 (m, 6H), 1.00-0.90 (m, 4H), 0.84-0.72 (m, 6H) ppm; ^{13}C NMR (100 MHz, $CDCl_3$) δ 142.9, 121.5, 109.4, 80.9, 67.0, 62.2, 56.6, 50.4, 46.5, 42.0, 41.7, 40.4, 39.8, 36.8, 36.7, 32.0, 32.0, 31.5, 31.3, 30.43, 30.41, 28.9, 20.7, 19.4, 17.3, 16.4, 14.7 ppm; HRMS (EI) (m/z) [M] $C_{27}H_{41}IO_2$ calcd. for 524.2151, found 524.2153.

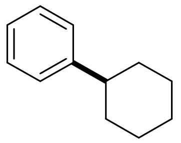

Cyclohexylbenzene (3aa)

According to **Procedure 5**. Yield: 18 mg, 56%; colorless liquid; ^{1}H NMR (400 MHz, $CDCl_3$) δ 7.33-7.27 (m, 2H), 7.24-7.15 (m, 3H), 2.58-2.47 (m, 1H), 1.94-1.82 (m, 4H), 1.78-1.71 (m, 1H), 1.50-1.33 (m, 4H), 1.31-1.22 (m, 1H) ppm; ^{13}C NMR (100 MHz, $CDCl_3$) δ 148.2, 128.4, 127.0, 125.9, 44.8, 34.6, 27.1, 26.3 ppm; HRMS (EI) (m/z) [M] $C_{12}H_{16}$ calcd. for 160.1252, found 160.1248.

Data are consistent with the literature.[11]

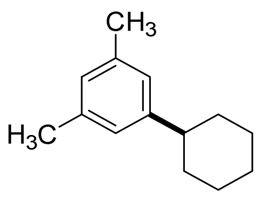

1-cyclohexyl-3,5-dimethylbenzene (3ba)

According to **Procedure 5**. Yield: 22 mg, 59%; colorless oil; ^{1}H NMR (400 MHz, $CDCl_3$) δ 6.87-6.82 (m, 3H), 2.49-2.37 (m, 1H), 2.31 (s, 6H),1.94-1.78 (m, 4H), 1.78-1.69 (m, 1H), 1.50-1.34 (m, 4H), 1.33-1.22 (m, 1H) ppm; ^{13}C NMR (100 MHz, $CDCl_3$) δ 148.3, 137.8, 127.6, 124.8, 44.7, 34.7, 27.1, 26.4, 21.5 ppm; HRMS (EI) (m/z) [M] $C_{14}H_{20}$ calcd. for 188.1565, found 188.1563.

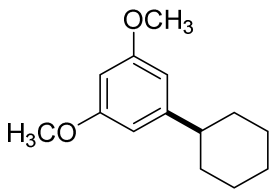

1-cyclohexyl-3,5-dimethoxybenzene (3ca)

According to **Procedure 5**. Yield: 30 mg, 68%; colorless oil; ^{1}H NMR (400 MHz, $CDCl_3$) δ 6.39 (d, *J* = 2.4 Hz, 2H), 6.31 (t, *J* = 2.4 Hz, 1H), 3.79 (s, 6H), 2.49-2.40 (m, 1H), 1.92-1.80 (m, 4H), 1.77-1.71 (m, 1H), 1.47-1.32 (m, 4H), 1.30-1.22 (m, 1H) ppm; ^{13}C NMR (100 MHz, $CDCl_3$) δ 160.8, 150.8, 105.2, 97.7, 55.4, 45.1, 34.5, 27.0, 26.3 ppm; HRMS (EI) (m/z) [M] $C_{14}H_{20}O_2$ calcd. for 220.1463, found 220.1462.

Data are consistent with the literature.[12]

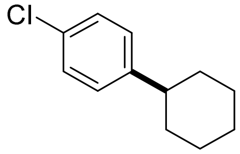

1-chloro-4-cyclohexylbenzene (3da)

According to **Procedure 5**. Yield: 30 mg, 77%; colorless oil; ^{1}H NMR (400 MHz, $CDCl_3$) δ 7.27-7.22 (m, 2H), 7.15-7.11 (m, 2H), 2.52-2.42 (m, 1H), 1.90-1.80 (m, 4H), 1.78-1.71 (m, 1H), 1.45-1.31 (m, 4H), 1.30-1.21 (m, 1H) ppm; ^{13}C NMR (100 MHz, $CDCl_3$) δ 146.7, 131.4, 128.5, 128.3, 44.1, 34.6, 27.0, 26.2 ppm; HRMS (EI) (m/z) [M] $C_{12}H_{15}{}^{35}Cl$ calcd. for 194.0862, found 194.0853.

Data are consistent of the literature.[13]

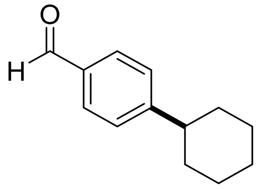

4-cyclohexylbenzaldehyde (3ea)

According to **Procedure 5**. Yield: 22 mg, 59%; colorless oil; ^{1}H NMR (400 MHz, $CDCl_3$) δ 9.97 (s, 1H), 7.82-7.77 (m, 2H), 7.39-7.34 (m, 2H), 2.62-2.52 (m, 1H), 1.93-1.82 (m, 4H), 1.80-1.73 (m, 1H), 1.50-1.31 (m, 5H) ppm; ^{13}C NMR (100 MHz, $CDCl_3$) δ 192.2, 155.5, 134.7, 130.1, 127.7, 45.1, 34.2, 26.8, 26.2 ppm; HRMS (ESI) (m/z) $[M+H]^+$ $C_{13}H_{16}O$ calcd. for 188.1201, found 188.1193.

Data are consistent of the literature.[14]

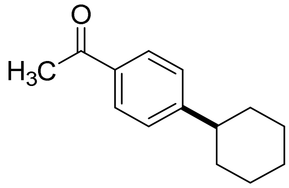

1-(4-cyclohexylphenyl)ethan-1-one (3fa)

According to **Procedure 5**. Yield: 29 mg, 72%; colorless oil; ^{1}H NMR (400 MHz, $CDCl_3$) δ 7.91-7.86 (m, 2H), 7.32-7.27 (m, 2H), 2.61-2.51 (m, 1H), 2.58 (s, 3H), 1.91-1.82 (m, 4H), 1.80-1.72 (m, 1H), 1.50-1.34 (m, 4H), 1.32-1.25 (m, 1H) ppm; ^{13}C NMR (100 MHz, $CDCl_3$) δ 198.0, 153.9, 135.2, 128.7, 127.2, 44.8, 34.3, 26.9, 26.7, 26.2 ppm; HRMS (EI) (m/z) [M] $C_{14}H_{18}O$ calcd. for 202.1358, found 202.1358.

Data are consistent with the literature.[11]

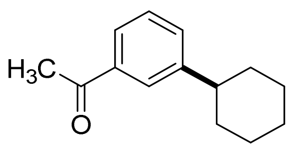

1-(3-cyclohexylphenyl)ethan-1-one (3ga)

According to **Procedure 5**. Yield: 32 mg, 80%; colorless oil; ^{1}H NMR (400 MHz, $CDCl_3$) δ 7.82-7.80 (m, 1H), 7.78-7.74 (m, 1H), 7.43-7.40 (m, 1H), 7.40-7.35 (m, 1H), 2.60 (s, 3H), 2.59-2.53 (m, 1H), 1.94-1.82 (m, 4H), 1.80-1.72 (m, 1H), 1.51-1.34 (m, 4H), 1.32-1.23 (m, 1H) ppm; ^{13}C NMR (100 MHz, $CDCl_3$) δ 198.6, 148.7, 137.4, 131.9, 128.6, 126.7, 126.2, 44.6, 34.5, 26.9, 26.8, 26.2 ppm; HRMS (EI) (m/z) [M] $C_{14}H_{18}O$ calcd. for 202.1358, found 202.1353.

Data are consistent with the literature.[15]

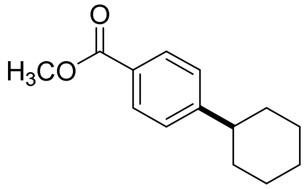

methyl 4-cyclohexylbenzoate (3ha)

According to **Procedure 5**. Yield: 27 mg, 62%; colorless oil; ^{1}H NMR (400 MHz, $CDCl_3$) δ 7.98-7.94 (m, 2H), 7.29-7.25 (m, 2H), 3.90 (s, 3H), 2.60-2.50 (m, 1H), 1.92-1.82 (m, 4H), 1.79-1.72 (m, 1H), 1.49-1.28 (m, 5H) ppm; ^{13}C NMR (100 MHz, $CDCl_3$) δ 167.3, 153.6, 129.8, 127.9, 127.0, 52.1, 44.8, 34.3, 26.9, 26.2 ppm; HRMS (EI) (m/z) [M] $C_{14}H_{18}O_2$ calcd. for 218.1307, found 218.1306.

Data are consistent of the literature.[13]

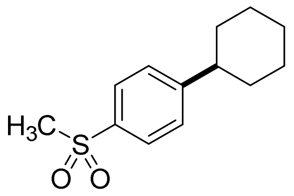

1-cyclohexyl-4-(methylsulfonyl)benzene (3ia)

According to **Procedure 5**. Yield: 35 mg, 71%; white solid, m.p. 101-102 °C; ^{1}H NMR (400 MHz, $CDCl_3$) δ 7.84 (d, *J* = 8.4 Hz, 2H), 7.39 (d, *J* = 8.4 Hz, 2H), 3.03 (s, 3H), 2.65-2.53 (m, 1H), 1.90-1.80 (m, 4H), 1.79-1.72 (m, 1H), 1.47-1.33 (m, 4H), 1.30-1.20 (m, 1H) ppm; ^{13}C NMR (100 MHz, $CDCl_3$) δ 154.6, 138.0, 128.0, 127.6, 44.8, 44.7, 34.2, 26.8, 26.1 ppm; HRMS (ESI) (m/z) $[M+H]^+$ $C_{13}H_{19}O_2S$ calcd. for 239.1100, found 239.1104.

Data are consistent of the literature.[13]

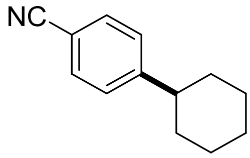

4-cyclohexylbenzonitrile (3ja)

According to **Procedure 5**, yield: 31 mg, 83%; according to **Procedure 6**, yield: 34 mg, 92%; colorless oil; ^{1}H NMR (400 MHz, $CDCl_3$) δ 7.60-7.53 (m, 2H), 7.32-7.27 (m, 2H), 2.60-2.49 (m, 1H), 1.92-1.80 (m, 4H), 1.79-1.71 (m, 1H), 1.47-1.33 (m, 4H), 1.30-1.21 (m, 1H) ppm; ^{13}C NMR (100 MHz, $CDCl_3$) δ 153.6, 132.3, 127.8, 119.3, 109.7, 44.9, 34.1, 26.7, 26.1 ppm; HRMS (EI) (m/z) [M] $C_{13}H_{15}N$ calcd. for 185.1204, found 185.1202.

Data are consistent of the literature.[13]

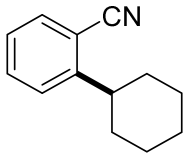

2-cyclohexylbenzonitrile (3ka)

According to **Procedure 5**. Yield: 30 mg, 81%; pale yellow oil; ^{1}H NMR (400 MHz, $CDCl_3$) δ 7.60 (dd, *J* = 7.6, 0.8 Hz, 1H), 7.55-7.50 (m, 1H), 7.36 (d, *J* = 8.0 Hz, 1H), 7.29-7.23 (m, 1H), 3.02-2.92 (m, 1H), 1.95-1.84 (m, 4H), 1.82-1.75 (m, 1H), 1.54-1.38 (m, 4H), 1.32-1.23 (m, 1H) ppm; ^{13}C NMR (100 MHz, $CDCl_3$) δ 151.6, 133.04, 133.00, 126.7, 126.4, 118.4, 112.0, 42.9, 33.8, 26.7, 26.1 ppm; HRMS (ESI) (m/z) $[M+H]^+$ $C_{13}H_{15}N$ calcd. for 185.1204, found 185.1196.

Data are consistent of the literature.[13]

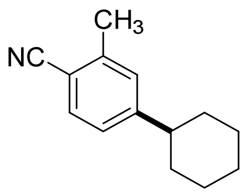

4-cyclohexyl-2-methylbenzonitrile (3la)

According to **Procedure 5**. Yield: 38 mg, 95%; colorless oil; ^{1}H NMR (400 MHz, $CDCl_3$) δ 7.50 (d, *J* = 7.6 Hz, 1H), 7.14 (s, 1H), 7.10 (d, *J* = 8.0 Hz, 1H), 2.55-2.46 (m, 1H), 2.51 (s, 3H), 1.89-1.80 (m, 4H), 1.79-1.72 (m, 1H), 1.45-1.32 (m, 4H), 1.30-1.20 (m, 1H) ppm; ^{13}C NMR (100 MHz, $CDCl_3$) δ 153.5, 142.0, 132.6, 129.0, 125.0, 118.6, 110.1, 44.8, 34.1, 26.8, 26.1, 20.6 ppm; HRMS (EI) (m/z) [M] $C_{14}H_{17}N$ calcd. for 199.1361, found 199.1357.

Data are consistent of the literature.[16]

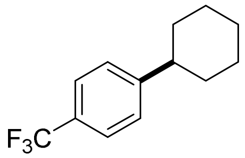

1-cyclohexyl-4-(trifluoromethyl)benzene (3ma)

According to **Procedure 5**. Yield: 30 mg, 65%; colorless oil; ^{1}H NMR (400 MHz, $CDCl_3$) δ 7.54 (d, *J* = 8.0 Hz, 2H), 7.32 (d, *J* = 8.0 Hz, 2H), 2.62-2.51 (m, 1H), 1.93-1.82 (m, 4H), 1.80-1.72 (m, 1H), 1.48-1.35 (m, 4H), 1.32-1.22 (m, 1H) ppm; ^{13}C NMR (100 MHz, $CDCl_3$) δ 152.2, 128.2 (q, $J_{C\text{-}F}$ = 32.0 Hz), 127.3, 125.4 (q, $J_{C\text{-}F}$ = 3.7 Hz), 124.6 (q, $J_{C\text{-}F}$ = 270.0 Hz), 44.7, 34.4, 26.9, 26.2 ppm; ^{19}F NMR (377 MHz, $CDCl_3$) δ -62.8 ppm; HRMS (EI) (m/z) [M] $C_{13}H_{15}F_3$ calcd. for 228.1126, found 228.1122.

Data are consistent with the literature.[11]

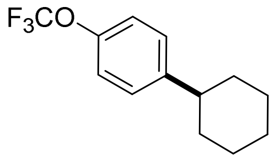

1-cyclohexyl-4-(trifluoromethoxy)benzene (3na)

According to **Procedure 5**. Yield: 41 mg, 84%; colorless oil; ^{1}H NMR (400 MHz, $CDCl_3$) δ 7.29-7.24 (m, 2H), 7.21-7.15 (m, 2H), 2.62-2.51 (m, 1H), 1.97-1.86 (m, 4H), 1.84-1.77 (m, 1H), 1.52-1.38 (m, 4H), 1.35-1.26 (m, 1H) ppm; ^{13}C NMR (100 MHz, $CDCl_3$) δ 147.4 (q, $J_{C\text{-}F}$ = 1.8 Hz), 146.9, 128.1, 120.9, 120.7 (q, $J_{C\text{-}F}$ = 254.7 Hz), 44.1, 34.6, 26.9, 26.2 ppm; ^{19}F NMR (377 MHz, $CDCl_3$) δ -58.4 ppm; HRMS (EI) (m/z) [M] $C_{13}H_{15}F_3O$ calcd. for 244.1075, found 244.1063.

Data are consistent with the literature.[11]

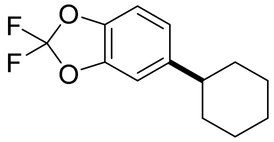

5-cyclohexyl-2,2-difluorobenzo[*d*][1,3]dioxole (3oa)

According to **Procedure 5**. Yield: 11 mg, 23%; colorless oil; ^{1}H NMR (400 MHz, $CDCl_3$) δ 6.96-6.86 (m, 3H), 2.55-2.45 (m, 1H), 1.92-1.80 (m, 4H), 1.78-1.71 (m, 1H), 1.42-1.32 (m, 4H), 1.27-1.20 (m, 1H) ppm; ^{13}C NMR (100 MHz, $CDCl_3$) δ 144.6, 143.9, 141.9, 131.8 ($J_{C\text{-}F}$ = 252.5 Hz), 121.8, 109.1, 108.1, 44.6, 34.8, 26.9, 26.1 ppm; ^{19}F NMR (377 MHz, $CDCl_3$) δ -50.6 ppm; HRMS (EI) (m/z) [M] $C_{13}H_{14}F_2O_2$ calcd. for 240.0962, found 240.0962.

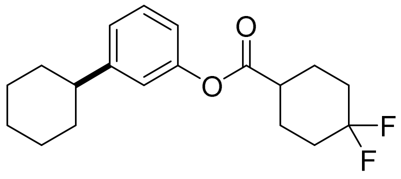

3-cyclohexylphenyl 4,4-difluorocyclohexane-1-carboxylate (3pa)

According to **Procedure 5**. Yield: 45 mg, 70%; colorless oil; ^{1}H NMR (400 MHz, $CDCl_3$) δ 7.29 (d, J = 8.0 Hz, 1H), 7.09 (d, J = 7.6 Hz, 1H), 6.92-6.85 (m, 2H), 2.72-2.62 (m, 1H), 2.56-2.46 (m, 1H), 2.24-2.10 (m, 4H), 2.08-1.96 (m, 2H), 1.94-1.79 (m, 6H), 1.78-1.72 (m, 1H), 1.46-1.32 (m, 4H), 1.30-1.22 (m, 1H) ppm; ^{13}C NMR (100 MHz, $CDCl_3$) δ 172.9, 150.7, 150.1, 129.3, 124.6, 122.7 (t, $J_{C\text{-}F}$ = 239.6 Hz), 119.8, 118.8, 44.5, 40.7, 34.4, 32.6 (t, $J_{C\text{-}F}$ = 24.5 Hz), 26.9, 26.2, 25.3-25.1 (m) ppm; ^{19}F NMR (377 MHz, $CDCl_3$) δ -95.1 (d, $J_{C\text{-}F}$ = 66.4 Hz), -99.9 (d, $J_{C\text{-}F}$ = 63.9 Hz) ppm; HRMS (EI) (m/z) [M] $C_{19}H_{24}F_2O_2$ calcd. for 322.1744, found 322.1738.

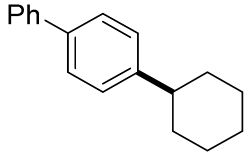

4-cyclohexyl-1,1'-biphenyl (3qa)

According to **Procedure 5**. Yield: 37 mg, 79%; m.p. 78-79 °C; ^{1}H NMR (400 MHz, $CDCl_3$) δ 7.62-7.56 (m, 2H), 7.55-7.50 (m, 2H), 7.46-7.39 (m, 2H), 7.35-7.27 (m, 3H), 2.60-2.49 (m, 1H), 1.96-1.84 (m, 4H), 1.81-1.72 (m, 1H), 1.52-1.36 (m, 4H), 1.34-1.28 (m, 1H) ppm; ^{13}C NMR (100 MHz, $CDCl_3$) δ 147.4, 141.3, 138.9, 128.8, 127.4, 127.18, 127.17, 127.1, 44.4, 34.6, 27.1, 26.3 ppm; HRMS (EI) (m/z) [M] $C_{18}H_{20}$ calcd. for 236.1565, found 236.1561.

Data are consistent with the literature.[11]

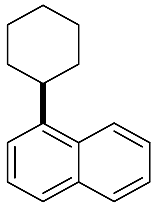

1-cyclohexylnaphthalene (3ra)

According to **Procedure 5**. Yield: 27 mg, 64%; colorless liquid; ^{1}H NMR (400 MHz, $CDCl_3$) δ 8.13 (d, *J* = 8.0 Hz, 1H), 7.86 (d, *J* = 8.0 Hz, 1H), 7.70 (d, *J* = 8.0 Hz, 1H), 7.54-7.38 (m, 4H), 3.41-3.28 (m, 1H), 2.12-1.81 (m, 5H), 1.64-1.50 (m, 4H), 1.42-1.30 (m, 1H) ppm; ^{13}C NMR (100 MHz, $CDCl_3$) δ 144.0, 134.1, 131.5, 129.1, 126.4, 125.8, 125.7, 125.4, 123.4, 122.4, 39.4, 34.4, 27.4, 26.7 ppm; HRMS (EI) (m/z) [M] $C_{16}H_{18}$ calcd. for 210.1409, found 210.1404.

Data are consistent with the literature.[11]

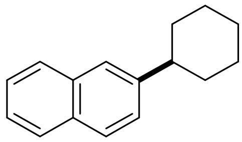

2-cyclohexylnaphthalene (3sa)

According to **Procedure 5**. Yield: 28 mg, 67%; colorless oil; ^{1}H NMR (400 MHz, $CDCl_3$) δ 7.83-7.75 (m, 3H), 7.64 (s, 1H), 7.47-7.36 (m, 3H), 2.72-2.62 (m, 1H), 2.02-1.94 (m, 2H), 1.93-1.85 (m, 2H), 1.83-1.76 (m, 1H), 1.62-1.38 (m, 4H), 1.37-1.25 (m, 1H) ppm; ^{13}C NMR (100 MHz, $CDCl_3$) δ 145.7, 133.8, 132.3, 127.9, 127.73, 127.69, 126.4, 125.9, 125.2, 124.7, 44.8, 34.6, 27.1, 26.4 ppm; HRMS (ESI) (m/z) $[M+H]^+$ $C_{16}H_{18}$ calcd. for 210.1409, found 210.1404.

Data are consistent with the literature.[11]

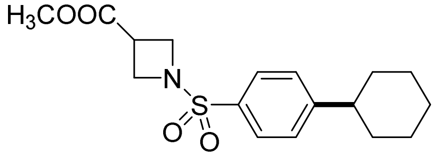

methyl 1-((4-cyclohexylphenyl)sulfonyl)azetidine-3-carboxylate (3ta)

According to **Procedure 5**. Yield: 56 mg, 83%; colorless oil; ^{1}H NMR (400 MHz, $CDCl_3$) δ 7.77-7.72 (m, 2H), 7.42-7.37 (m, 2H), 4.02-3.90 (m, 4H), 3.61 (s, 3H), 3.28-3.18 (m, 1H), 2.64-2.55 (m, 1H), 1.94-1.82 (m, 4H), 1.80-1.73 (m, 1H), 1.50-1.34 (m, 4H), 1.32-1.20 (m, 1H) ppm; ^{13}C NMR (100 MHz, $CDCl_3$) δ 171.7, 154.2, 131.8, 128.6, 127.8, 52.9, 52.5, 44.7, 34.2, 31.7, 26.8, 26.1 ppm; HRMS (ESI) (m/z) $[M+H]^+$ $C_{17}H_{24}NO_4S$ calcd. for 338.1421, found 338.1424.

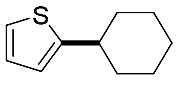

2-cyclohexylthiophene (3ua)

According to **Procedure 5**. Yield: 23 mg, 69%; colorless oil; ^{1}H NMR (400 MHz, $CDCl_3$) δ 7.11 (dd, *J* = 5.2, 0.8 Hz, 1H), 6.93 (dd, *J* = 5.2, 3.2 Hz, 1H), 6.81-6.78 (m, 1H), 2.85-2.78 (m, 1H), 2.10-2.02 (m, 2H), 1.85-1.78 (m, 2H), 1.75-1.67 (m, 1H), 1.50-1.28 (m, 5H) ppm; ^{13}C NMR (100 MHz, $CDCl_3$) δ 152.5, 126.6, 122.3, 121.9, 39.5, 35.7, 26.6, 26.1 ppm; HRMS (EI) (m/z) [M] $C_{10}H_{14}S$ calcd. for 166.0816, found 166.0808.

Data are consistent with the literature.[17]

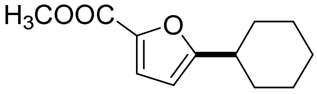

methyl 5-cyclohexylfuran-2-carboxylate (3va)

According to **Procedure 5**. Yield: 22 mg, 53%; colorless oil; ^{1}H NMR (400 MHz, $CDCl_3$) δ 7.09 (d, *J* = 3.6 Hz, 1H), 6.08 (d, *J* = 3.2, 0.8 Hz, 1H), 3.86 (s, 3H), 2.75-2.66 (m, 1H), 2.09-2.01 (m, 2H), 1.82-1.76 (m, 2H), 1.73-1.67 (m, 1H), 1.46-1.29 (m, 4H), 1.29-1.21 (m, 1H) ppm; ^{13}C NMR (100 MHz, $CDCl_3$) δ 166.0, 159.5, 142.7, 119.3, 105.7, 51.8, 37.6, 31.4, 26.0, 25.9 ppm; HRMS (EI) (m/z) [M] $C_{12}H_{13}O_3$ calcd. for 208.1094, found 208.1094.

Data are consistent with the literature.[18]

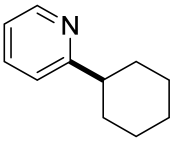

2-cyclohexylpyridine (3wa)

According to **Procedure 5**. Yield: 23 mg, 72%; pale yellow oil; ^{1}H NMR (400 MHz, $CDCl_3$) δ 8.55-8.50 (m, 1H), 7.62-7.56 (m, 1H), 7.14 (d, *J* = 8.0 Hz, 1H), 7.11-7.05 (m, 1H), 2.74-2.63 (m, 1H), 2.00-1.90 (m, 2H), 1.89-1.81 (m, 2H), 1.79-1.71 (m, 1H), 1.59-1.32 (m, 5H) ppm; ^{13}C NMR (100 MHz, $CDCl_3$) δ 166.7, 149.2, 136.5, 121.1, 46.8, 33.1, 26.8, 26.2 ppm; HRMS (ESI) (m/z) $[M+H]^+$ $C_{11}H_{16}N$ calcd. for 162.1277, found 162.1278.

Data are consistent of the literature.[13]

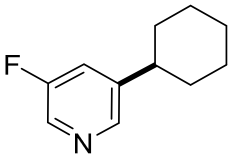

3-cyclohexyl-5-fluoropyridine (3xa)

According to **Procedure 5**. Yield: 20 mg, 56%; colorless oil; ^{1}H NMR (400 MHz, $CDCl_3$) δ 8.31-8.24 (m, 2H), 7.25-7.21 (m, 1H), 2.64-2.51 (m, 1H), 1.93-1.83 (m, 4H), 1.80-1.73 (m, 1H), 1.46-1.33 (m, 4H),

1.31-1.21 (m, 1H) ppm; ^{13}C NMR (100 MHz, $CDCl_3$) δ 159.9 ($J_{C\text{-}F}$ = 254.1 Hz), 145.1 ($J_{C\text{-}F}$ = 2.9 Hz), 145.0 ($J_{C\text{-}F}$ = 3.6 Hz), 135.6 ($J_{C\text{-}F}$ = 3.0 Hz), 120.9 ($J_{C\text{-}F}$ = 17.4 Hz), 41.7, 34.1, 26.7, 26.0 ppm; ^{19}F NMR (377 MHz, $CDCl_3$) δ -128.3 ppm; HRMS (EI) (m/z) [M] $C_{11}H_{14}FN$ calcd. for 179.1110, found 179.1107.

Data are consistent of the literature.[13]

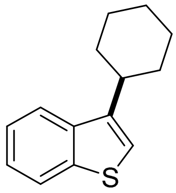

3-cyclohexylbenzo[*b*]thiophene (3ya)

According to **Procedure 5**. Yield: 29 mg, 67%; colorless oil; ^{1}H NMR (400 MHz, $CDCl_3$) δ 7.88-7.83 (m, 1H), 7.81-7.77 (m, 1H), 7.40-7.30 (m, 2H), 7.07 (s, 1H), 2.99-2.89 (m, 1H), 2.15-2.07 (m, 2H), 1.95-1.86 (m, 2H), 1.85-1.77 (m, 1H), 1.57-1.43 (m, 4H), 1.39-1.29 (m, 1H) ppm; ^{13}C NMR (100 MHz, $CDCl_3$) δ 143.0, 140.7, 138.7, 124.2, 123.8, 123.1, 121.9, 119.3, 38.1, 33.7, 27.0, 26.6 ppm; HRMS (EI) (m/z) [M] $C_{12}H_{13}O_3$ calcd. for 208.1094, found 208.1094.

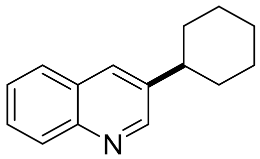

3-cyclohexylquinoline (3za)

According to **Procedure 5**. Yield: 30 mg, 71%; pale yellow oil; ^{1}H NMR (400 MHz, $CDCl_3$) δ 8.81 (d, J = 2.0 Hz, 1H), 8.07 (d, J = 8.4 Hz, 1H), 7.91 (d, J = 2.4 Hz, 1H), 7.77 (d, J = 8.0 Hz, 1H), 7.67-7.61 (m, 1H), 7.53-7.48 (m, 1H), 2.77-2.67 (m, 1H), 2.03-1.96 (m, 2H), 1.93-1.87 (m, 2H), 1.84-1.76 (m, 1H), 1.59-1.40 (m, 4H), 1.37-1.27 (m, 1H) ppm; ^{13}C NMR (100 MHz, $CDCl_3$) δ 151.6, 147.1, 140.5, 132.3, 129.3, 128.6, 128.4, 127.6, 126.6, 42.2, 34.3, 26.9, 26.1 ppm; HRMS (ESI) (m/z) $[M+H]^+$ $C_{15}H_{18}N$ calcd. for 212.1434, found 212.1435.

Data are consistent of the literature.[19]

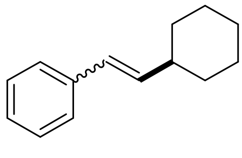

(2-cyclohexylvinyl)benzene (3aaa)

According to **Procedure 5**, yield: 26 mg, 70%; according to **Procedure 6**, yield: 31 mg, 84%; colorless oil, as a mixture of isomers, *E*/*Z* = 4:1; ^{1}H NMR (400 MHz, $CDCl_3$) δ 7.38-7.16 (m, 5H), 6.39-6.29 (m, 1H), 6.19 (dd, J = 16.0, 6.8 Hz, 0.80H, the *E* isomer), 5.50 (dd, J = 11.2, 9.6 Hz, 0.20H, the *Z* isomer), 2.65-2.55 (m, 0.20H, the *Z* isomer), 2.20-2.08 (m, 0.80H, the *E* isomer), 1.85-1.65 (m, 5H), 1.38-1.15

(m, 5H) ppm; ^{13}C NMR (100 MHz, $CDCl_3$) δ 139.1 (the *Z* isomer), 138.2 (the *E* isomer), 138.1 (the *Z* isomer), 137.0 (the *E* isomer), 128.7 (the *Z* isomer), 128.6 (the *E* isomer), 128.3 (the *Z* isomer), 127.4(the *E* isomer), 127.0 (the *Z* isomer), 126.9 (the *E* isomer), 126.6 (the *Z* isomer), 126.1 (the *E* isomer), 41.3 (the *E* isomer), 37.0 (the *Z* isomer), 33.4 (the *Z* isomer), 33.1 (the *E* isomer), 26.3, 26.2 (the *E* isomer), 25.8 (the *Z* isomer) ppm; HRMS (EI) (m/z) [M] $C_{16}H_{21}N$ calcd. for 186.1409, found 186.1400 (the *Z* isomer), 186.1405 (the *E* isomer).

Data are consistent with the literature.[18]

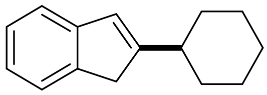

2-cyclohexyl-1H-indene (3aba)

According to **Procedure 5**, yield: 23 mg, 58%; according to **Procedure 6**, yield: 30 mg, 77%; colorless oil; ^{1}H NMR (400 MHz, $CDCl_3$) δ 7.39 (dd, *J* = 7.2, 0.4 Hz, 1H), 7.28 (d, *J* = 7.6 Hz, 1H), 7.24-7.19 (m, 1H), 7.13-7.07 (m, 1H), 6.50 (s, 3H), 3.34 (s, 2H), 2.47-2.37 (s, 1H), 1.99-1.91 (m, 2H), 1.86-1.78 (m, 2H), 1.77-1.69 (m, 1H), 1.44-1.30 (m, 4H), 1.29-1.23 (m, 1H) ppm; ^{13}C NMR (100 MHz, $CDCl_3$) δ 156.4, 145.8, 143.0, 126.4, 124.3, 123.64, 123.60, 120.1, 40.1, 39.4, 33.3, 26.6, 26.5 ppm; HRMS (EI) (m/z) [M] $C_{15}H_{18}$ calcd. for 198.1409, found 198.1407.

Data are consistent with the literature.[18]

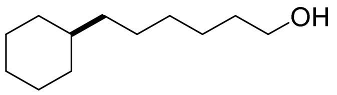

6-cyclohexylhexan-1-ol (3aca)

According to **Procedure 5**, yield: 14 mg, 37%; ^{1}H NMR (400 MHz, $CDCl_3$) δ 3.64 (t, *J* = 6.8 Hz, 2H), 1.73-1.63 (m, 4H), 1.60-1.51 (m, 3H), 1.39-1.17 (m, 14H) ppm; ^{13}C NMR (100 MHz, $CDCl_3$) δ 63.3, 37.8, 37.6, 33.6, 33.0, 29.9, 27.0, 26.9, 26.6, 25.9 ppm; HRMS (EI) (m/z) [M-H_2O] $C_{12}H_{22}$ calcd. for 166.1722, found 166.1720.

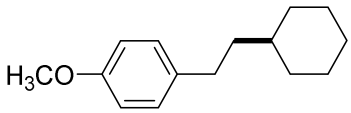

1-(2-cyclohexylethyl)-4-methoxybenzene (3ada)

According to **Procedure 5**, yield: 20 mg, 46%; colorless oil; ^{1}H NMR (400 MHz, $CDCl_3$) δ 7.11-7.06 (m, 2H), 6.84-6.79 (m, 2H), 3.79 (s, 3H), 2.58-2.52 (m, 2H), 1.80-1.62 (m, 5H), 1.50-1.42 (m, 2H), 1.25-1.12 (m, 4H), 0.98-0.88 (m, 2H) ppm; ^{13}C NMR (100 MHz, $CDCl_3$) δ 157.7, 135.5, 129.3, 113.8, 55.4, 39.8, 37.4, 33.5, 32.4, 26.9, 26.5 ppm; HRMS (EI) (m/z) [M] $C_{15}H_{22}O$ calcd. for 218.1671, found 218.1674.

Data are consistent with the literature.[20]

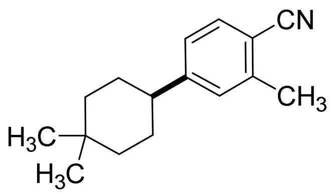

4-(4,4-dimethylcyclohexyl)-2-methylbenzonitrile (3lb)

According to **Procedure 5**, yield: 24 mg, 53%; according to **Procedure 6**, yield: 34 mg, 75%; colorless oil; ^{1}H NMR (400 MHz, $CDCl_3$) δ 7.51 (d, *J* = 8.0 Hz, 1H), 7.15 (s, 1H), 7.12 (d, *J* = 8.0 Hz, 1H), 2.52 (s, 3H), 2.45-2.37 (m, 1H), 1.70-1.57 (m, 4H), 1.53-1.47 (m, 2H), 1.37-1.28 (m, 2H), 0.98 (s, 3H), 0.96 (s, 3H) ppm; ^{13}C NMR (100 MHz, $CDCl_3$) δ 153.3, 142.0, 132.7, 129.0, 125.1, 118.7, 110.1, 44.8, 39.6, 33.2, 29.89, 29.85, 24.3, 20.7 ppm; HRMS (EI) (m/z) [M] $C_{16}H_{21}N$ calcd. for 227.1674, found 227.1670.

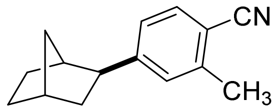

4-((1*S*,2*S*,4*R*)-bicyclo[2.2.1]heptan-2-yl)-2-methylbenzonitrile (3lc)

According to **Procedure 5**, yield: 37 mg, 88%; according to **Procedure 6**, yield: 38 mg, 90%; colorless oil; ^{1}H NMR (400 MHz, $CDCl_3$) δ 7.49 (d, *J* = 8.0 Hz, 1H), 7.14 (s, 1H), 7.10 (d, *J* = 7.6 Hz, 1H), 2.76-2.70 (m, 1H), 2.51 (s, 3H), 2.39-2.33 (m, 2H), 1.83-1.75 (m, 1H), 1.64-1.55 (m, 3H), 1.49-1.43 (m, 1H), 1.39-1.19 (m, 3H) ppm; ^{13}C NMR (100 MHz, $CDCl_3$) δ 153.1, 141.8, 132.6, 129.2, 125.1, 118.7, 109.7, 47.6, 42.7, 39.2, 36.9, 36.4, 30.6, 28.9, 20.7 ppm; HRMS (EI) (m/z) [M] $C_{15}H_{17}N$ calcd. for 211.1361, found 211.1356.

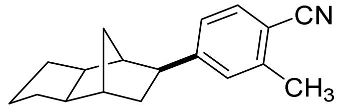

2-methyl-4-(octahydro-1*H*-4,7-methanoinden-5-yl)benzonitrile (3ld)

According to **Procedure 5**, yield: 45 mg, 90%; according to **Procedure 6**, yield: 44 mg, 89%; colorless oil; ^{1}H NMR (400 MHz, $CDCl_3$) δ 7.49 (d, *J* = 8.0 Hz, 1H), 7.15 (s, 1H), 7.11 (d, *J* = 8.0 Hz, 1H), 2.68-2.62 (m, 1H), 2.51 (s, 3H), 2.13 (s, 1H), 2.10 (d, *J* = 3.6 Hz, 1H), 2.00-1.84 (m, 4H), 1.75-1.63 (m, 2H), 1.60-1.54 (m, 1H), 1.37-1.33 (m, 1H), 1.29-1.21 (m, 1H), 1.20-1.16 (m, 1H), 1.02-0.91 (m, 2H) ppm; ^{13}C NMR (100 MHz, $CDCl_3$) δ 153.1, 141.8, 132.5, 129.3, 125.2, 118.7, 109.6, 49.2, 47.8, 47.1, 47.0, 41.4, 38.0, 32.4, 32.1, 30.2, 27.6, 20.7 ppm; HRMS (EI) (m/z) [M] $C_{18}H_{21}N$ calcd. for 251.1674, found 251.1675.

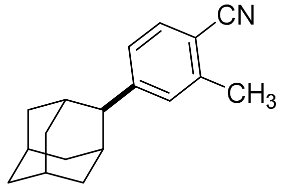

4-((1*R*,3*S*,5*r*,7*r*)-adamantan-2-yl)-2-methylbenzonitrile (3le)

According to **Procedure 6**, yield: 22 mg, 44%; colorless oil; ^{1}H NMR (400 MHz, $CDCl_3$) δ 7.54 (d, *J* = 8.0 Hz, 1H), 7.29 (s, 1H), 7.26 (d, *J* = 8.0 Hz, 1H), 2.98 (s, 1H), 2.54 (s, 3H), 2.45 (s, 2H), 2.05-1.90 (m, 5H), 1.82-1.70 (m, 5H), 1.62-1.55 (m, 2H) ppm; ^{13}C NMR (100 MHz, $CDCl_3$) δ 150.3, 141.7, 132.5, 129.0, 125.0, 118.7, 109.5, 47.2, 39.1, 37.8, 32.1, 31.1, 28.0, 27.7, 20.9 ppm; HRMS (EI) (m/z) [M] $C_{18}H_{21}N$ calcd. for 251.1674, found 251.1669.

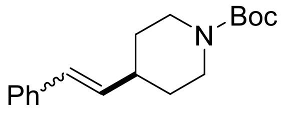

***tert*-butyl 4-styrylpiperidine-1-carboxylate (3aaf)**

According to **Procedure 6**, yield: 53 mg, 92%; colorless oil, as a mixture of isomers, *E*/*Z* = 16:1; ^{1}H NMR (400 MHz, $CDCl_3$) δ 7.37-7.27 (m, 4H), 7.23-7.18 (m, 1H), 6.40 (d, *J* = 11.2 Hz, 0.06H, the *Z* isomer), 6.39 (d, *J* = 16.0 Hz, 0.94H, the *E* isomer), 6.15 (dd, *J* = 16.0, 6.8 Hz, 0.94H, the *E* isomer), 5.46 (dd, *J* = 11.6, 10.0 Hz, 0.06H, the *Z* isomer), 4.25-4.00 (m, 2H), 2.85-2.70 (m, 2H), 2.35-2.20 (m, 1H), 1.80-1.70 (m, 2H), 1.472 (s, 8.50H, the *E* isomer), 1.465 (s, 0.50H, the *Z* isomer), 1.44-1.32 (m, 2H) ppm; ^{13}C NMR (100 MHz, $CDCl_3$) the *Z* isomer δ 155.0, 137.6, 134.5, 128.7, 128.6, 127.2, 126.2, 79.5, 43.9, 39.5, 32.0, 28.6 ppm; HRMS (ESI) (m/z) $[M+Na]^+$ $C_{18}H_{25}NNaO_2$ calcd. for 310.1778, found 310.1782.

Data are consistent with the literature.[21]

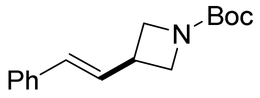

***tert*-butyl (*E*)-3-styrylazetidine-1-carboxylate (3aag)**

According to **Procedure 6**, yield: 41 mg, 79%; pale yellow oil, *E*/*Z* > 30:1; ^{1}H NMR (400 MHz, $CDCl_3$) the *E* isomer δ 7.39-7.28 (m, 4H), 7.26-7.21 (m, 1H), 6.44 (d, *J* = 16.0 Hz, 1H), 6.36 (dd, *J* = 16.0, 7.6 Hz, 1H), 4.17 (d, *J* = 8.4 Hz, 1H), 4.15 (d, *J* = 8.4 Hz, 1H), 3.83 (d, *J* = 8.8 Hz, 1H), 3.82 (d, *J* = 8.4 Hz, 1H), 3.41-3.30 (m, 1H), 1.46 (s, 9H) ppm; ^{13}C NMR (100 MHz, $CDCl_3$) δ 156.5, 136.9, 131.3, 130.5, 128.8, 127.7, 126.3, 79.6, 55.1, 32.0, 28.6 ppm; HRMS (ESI) (m/z) $[M+Na]^+$ $C_{16}H_{21}NNaO_2$ calcd. for 282.1465, found 282.1468.

Data are consistent with the literature.[22]

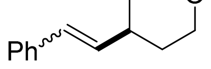

4-styryltetrahydro-2*H*-pyran (3aah)

According to **Procedure 6**, yield: 36 mg, 96%; colorless oil, as a mixture of isomers, *E*/*Z* = 16:1; ^{1}H NMR (400 MHz, $CDCl_3$) the *E* isomer δ 7.38-7.27 (m, 4H), 7.24-7.18 (m, 1H), 6.39 (d, *J* = 16.0 Hz, 1H), 6.16 (dd, *J* = 16.0, 6.8 Hz, 1H), 4.05-3.97 (m, 2H), 3.47 (dt, *J* = 11.6, 2.4 Hz, 2H), 2.44-2.32 (m, 1H), 1.75-1.67 (m, 2H), 1.63-1.51 (m, 2H) ppm; ^{13}C NMR (100 MHz, $CDCl_3$) δ 137.7, 134.8, 128.7, 128.4, 127.2, 126.2, 67.9, 38.5, 32.8 ppm; HRMS (EI) (m/z) [M] $C_{13}H_{16}O$ calcd. for 188.1201, found 188.1194 (the *Z* isomer) and 188.1199 (the *E* isomer).

Data are consistent with the literature.[23]

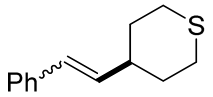

4-styryltetrahydro-2*H*-thiopyran (3aai)

According to **Procedure 6**, yield: 35 mg, 86%; colorless crystal, m.p. 63 °C; as a mixture of isomers, *E*/*Z* = 13:1; ^{1}H NMR (400 MHz, $CDCl_3$) δ 7.37-7.27 (m, 4H), 7.24-7.18 (m, 1H), 6.40-6.33 (m, 1H), 6.14 (dd, *J* = 16.0, 7.2 Hz, 0.93H, the *E* isomer), 6.14 (dd, *J* = 11.6, 10.0 Hz, 0.07H, the *Z* isomer), 2.80-2.62 (m, 4H), 2.24-1.98 (m, 3H), 1.68-1.57 (m, 2H) ppm; ^{13}C NMR (100 MHz, $CDCl_3$) the *E* isomer δ 137.7, 135.3, 128.7, 128.5, 127.2, 126.2, 40.8, 33.8, 28.5 ppm; HRMS (EI) (m/z) [M] $C_{13}H_{16}S$ calcd. for 204.0973, found 204.0973.

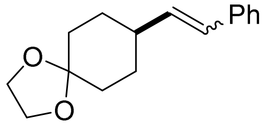

8-styryl-1,4-dioxaspiro[4.5]decane (3aaj)

According to **Procedure 6**, yield: 46 mg, 94%; colorless oil, as a mixture of two isomers, *E*/*Z* > 7.5:1; ^{1}H NMR (400 MHz, $CDCl_3$) δ 7.37-7.26 (m, 4H), 7.24-7.16 (m, 1H), 6.44-6.33 (m, 1H), 6.18 (dd, *J* = 16.0, 7.2 Hz, 0.88H, the *E* isomer), 5.52 (dd, *J* = 11.6, 7.2 Hz, 0.12H, the *Z* isomer), 3.98-3.94 (m, 4H), 2.68-2.52 (m, 0.12H, the *Z* isomer), 2.25-2.14 (m, 0.88H, the *E* isomer), 1.87-1.72 (m, 4H), 1.67-1.50 (m, 4H) ppm; ^{13}C NMR (100 MHz, $CDCl_3$) δ 137.9 (the *E* isomer), 137.7 (the *Z* isomer), 135.3 (the *E* isomer), 128.7 (the *Z* isomer), 128.6 (the *E* isomer), 128.4 (the *Z* isomer), 128.3 (the *E* isomer), 128.0 (the *Z* isomer), 127.0 (the *E* isomer), 126.7 (the *Z* isomer), 126.1 (the *E* isomer), 108.8 (the *E* isomer), 108.6 (the *Z* isomer), 64.42 (the *E* isomer), 64.40 (the *E* isomer), 40.0 (the *E* isomer), 35.7 (the *Z* isomer), 34.4 (the *E* isomer), 34.2 (the *Z* isomer), 30.6 (the *Z* isomer), 30.2 (the *E* isomer) ppm; HRMS (EI) (m/z) [M] $C_{16}H_{20}O_2$ calcd. for 244.1463, found 244.1456 (the *Z* isomer) and 244.1461 (the *E* isomer).

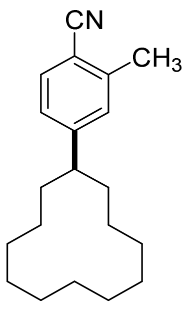

4-cyclododecyl-2-methylbenzonitrile (3lk)

According to **Procedure 6**, yield: 27 mg, 48%; colorless oil; ^{1}H NMR (400 MHz, $CDCl_3$) δ 7.50 (d, *J* = 8.0 Hz, 1H), 7.11 (s, 1H), 7.09 (d, *J* = 8.0 Hz, 1H), 2.77 (pent, *J* = 6.4 Hz, 1H), 2.52 (s, 3H), 1.84-1.73 (m, 2H), 1.49-1.22 (m, 20H) ppm; ^{13}C NMR (100 MHz, $CDCl_3$) δ 153.4, 141.9, 132.6, 129.8, 125.8, 118.7, 110.1, 40.2, 31.4, 24.0, 23.9, 23.6, 23.3, 22.7, 20.7 ppm; HRMS (EI) (m/z) [M] $C_{20}H_{29}N$ calcd. for 283.2300, found 283.2293.

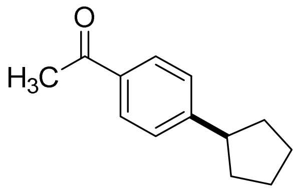

1-(4-cyclopentylphenyl)ethan-1-one (3fl)

According to **Procedure 5**, yield: 24 mg, 64%; pale yellow oil; ^{1}H NMR (400 MHz, $CDCl_3$) δ 7.92-7.85 (m, 2H), 7.35-7.29 (m, 2H), 3.12-2.97 (m, 1H), 2.58 (s, 3H), 2.15-2.02 (m, 2H), 1.88-1.58 (m, 6H) ppm; ^{13}C NMR (100 MHz, $CDCl_3$) δ 198.0, 152.7, 135.1, 128.6, 127.4, 46.1, 34.7, 26.7, 25.7 ppm; HRMS (ESI) (m/z) $[M+H]^+$ $C_{13}H_{17}O$ calcd. for 189.1274, found 189.1276.

Data are consistent with the literature.[24]

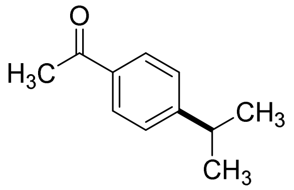

1-(4-isopropylphenyl)ethan-1-one (3fm)

According to **Procedure 5**, yield: 19 mg, 59%; colorless oil; ^{1}H NMR (400 MHz, $CDCl_3$) δ 7.92-7.87 (m, 2H), 7.34-7.29 (m, 2H), 2.97 (hept, *J* = 6.4 Hz, 1H), 2.58 (s, 3H), 1.27 (d, *J* = 6.8 Hz, 6H) ppm; ^{13}C NMR (100 MHz, $CDCl_3$) δ 198.0, 154.8, 135.2, 128.7, 126.8, 34.4, 26.7, 23.8 ppm; HRMS (EI) (m/z) [M] $C_{11}H_{14}O$ calcd. for 162.1045, found 162.1038.

Data are consistent with the literature.[25]

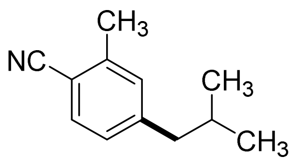

4-isobutyl-2-methylbenzonitrile (3ln)

According to **Procedure 5**, yield: 28 mg, 80%; according to **Procedure 6**, yield: 22 mg, 65%; colorless oil; ^{1}H NMR (400 MHz, $CDCl_3$) δ 7.49 (d, *J* = 8.0 Hz), 7.08 (s, 1H), 7.04 (d, *J* = 8.0 Hz), 2.51 (s, 3H), 2.48 (d, *J* = 7.2 Hz, 2H), 1.94-1.79 (m, 1H), 0.90 (d, *J* = 7.2 Hz, 6H) ppm; ^{13}C NMR (100 MHz, $CDCl_3$) δ 147.3, 141.8, 132.4, 131.1, 127.2, 118.6, 110.1, 45.5, 30.2, 22.4, 20.6 ppm; HRMS (EI) (m/z) [M] $C_{12}H_{15}N$ calcd. for 173.1204, found 173.1201.

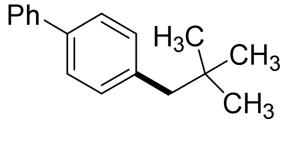

4-neopentyl-1,1'-biphenyl (3qo)

According to **Procedure 5**, yield: 38 mg, 84%; colorless crystal, m.p. 57 °C; ^{1}H NMR (400 MHz, $CDCl_3$) δ 7.62-7.57 (m, 2H), 7.53-7.49 (m, 2H), 7.46-7.40 (m, 2H), 7.36-7.31 (m, 1H), 7.23-7.18 (m, 2H), 2.54 (s, 2H), 0.95 (s, 9H) ppm; ^{13}C NMR (100 MHz, $CDCl_3$) δ 139.1, 138.7, 131.0, 128.9, 128.8, 127.3, 127.1, 126.5, 50.0, 32.0, 29.6 ppm; HRMS (ESI) (m/z) $[M+H]^+$ $C_{17}H_{20}$ calcd. for 224.1565, found 224.1561.

Data are consistent with the literature.[26]

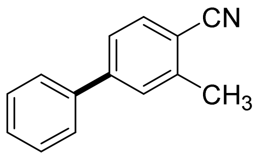

3-methyl-[1,1'-biphenyl]-4-carbonitrile (3lp)

According to **Procedure 5**, yield: 15 mg, 38%; white solid, m.p. 71 °C; ^{1}H NMR (400 MHz, $CDCl_3$) δ 7.66 (d, *J* = 8.0 Hz, 1H), 7.60-7.56 (m, 2H), 7.53 (s, 1H), 7.50-7.45 (m, 3H), 7.44-7.39 (m, 1H), 2.62 (s, 3H) ppm; ^{13}C NMR (100 MHz, $CDCl_3$) δ 145.7, 142.5, 139.5, 133.1, 129.2, 129.0, 128.6, 127.4, 125.1, 118.4, 111.5, 20.8 ppm; HRMS (EI) (m/z) [M] $C_{14}H_{11}N$ calcd. for 193.0891, found 193.0890.

Data are consistent with the literature.[27]

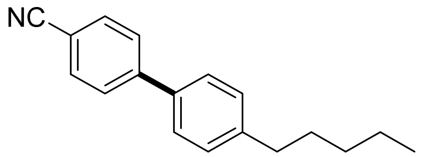

3-cyclohexylpropyl 3-(4,5-diphenyloxazol-2-yl)propanoate (3jq)

According to **Procedure 5**, yield: 12 mg, 25%; colorless oil; ^{1}H NMR (400 MHz, $CDCl_3$) δ 7.73-7.65 (m, 4H), 7.54-7.48 (m, 2H), 7.32-7.27 (m, 2H), 2.66 (t, *J* = 7.6 Hz, 2H), 1.70-1.60 (m, 2H), 1.40-1.30 (m, 4H), 0.94-0.88 (m, 3H) ppm; ^{13}C NMR (100 MHz, $CDCl_3$) δ 145.8, 144.0, 136.6, 132.7, 129.3, 127.6, 127.2, 119.2, 110.7, 35.7, 31.6, 31.2, 22.7, 14.2 ppm; HRMS (EI) (m/z) [M] $C_{18}H_{19}N$ calcd. for 249.1517, found 249.1516.

Data are consistent with the literature.[28]

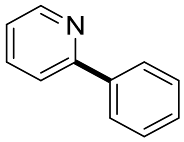

2-phenylpyridine (3wp)

According to **Procedure 5**, yield: 11 mg, 35%; colorless oil; ^{1}H NMR (400 MHz, $CDCl_3$) δ 8.73-8.68 (m, 1H), 8.03-7.97 (m, 2H), 7.78-7.70 (m, 2H), 7.51-7.46 (m, 2H), 7.44-7.38 (m, 1H), 7.25-7.21 (m, 1H) ppm; ^{13}C NMR (100 MHz, $CDCl_3$) δ 157.6, 149.8, 139.6, 136.9, 129.1, 128.9, 127.1, 122.2, 120.7 ppm; HRMS (EI) (m/z) [M] $C_{11}H_9N$ calcd. for 155.0735, found 155.0727.

Data are consistent with the literature.[29]

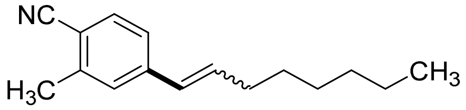

2-methyl-4-(oct-1-en-1-yl)benzonitrile (3lr)

According to **Procedure 5**, yield: 32 mg, 71%; colorless oil, as a mixture of isomers, *E*/*Z* = 2.7:1; ^{1}H NMR (400 MHz, $CDCl_3$) δ 7.54 (d, *J* = 8.0 Hz, 0.27H, the *Z* isomer), 7.50 (d, *J* = 8.0 Hz, 0.73H, the *E* isomer), 7.25-7.14 (m, 2H), 6.40-6.30 (m, 1.73H), 5.79 (dt, *J* = 11.6, 7.6 Hz, 0.27H, the *Z* isomer), 2.53 (s, 0.80H, the *Z* isomer), 2.52 (s, 2.20H, the *E* isomer), 2.32-2.20 (m, 2H), 1.51-1.41 (m, 2H), 1.36-1.25 (m, 6H), 0.92-0.85 (m, 3H) ppm; ^{13}C NMR (100 MHz, $CDCl_3$) δ (difficult to assign every peak) 142.5, 142.4, 142.1, 141.8, 136.4, 135.4, 132.8, 132.4, 130.6, 128.6, 127.7, 127.6, 126.6, 123.7, 118.6, 118.5, 110.52, 110.46, 33.3, 31.83, 31.79, 29.8, 29.2, 29.1, 29.0, 28.9, 22.74, 22.72, 20.66, 20.61, 14.22, 14.18 ppm; HRMS (EI) (m/z) [M] $C_{16}H_{21}N$ calcd. for 227.1668, found 227.1665 (the *Z* isomer), 227.1666 (the *E* isomer).

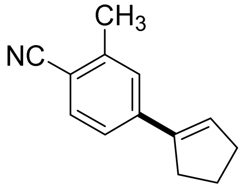

4-(cyclopent-1-en-1-yl)-2-methylbenzonitrile (3ls)

According to **Procedure 5**, yield: 16 mg, 44%; colorless oil; ^{1}H NMR (400 MHz, $CDCl_3$) δ 7.52 (d, J = 8.0 Hz, 1H), 7.33 (s, 1H), 7.32-7.29 (m, 1H), 6.35-6.31 (m, 1H), 2.73-2.65 (m, 2H), 2.60-2.52 (m, 2H), 2.53 (s, 3H), 2.10-1.97 (m, 2H) ppm; ^{13}C NMR (100 MHz, $CDCl_3$) δ 141.9, 141.5, 141.2, 132.6, 130.5, 127.3, 123.5, 118.7, 110.6, 33.70, 33.1, 23.4, 20.7 ppm; HRMS (EI) (m/z) [M] $C_{13}H_{13}N$ calcd. for 183.1042, found 183.1041.

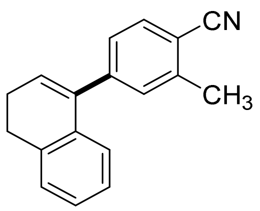

4-(3,4-dihydronaphthalen-1-yl)-2-methylbenzonitrile (3lt)

According to **Procedure 5**, yield: 35 mg, 71%; colorless oil; ^{1}H NMR (400 MHz, $CDCl_3$) δ 7.60 (d, J = 8.0 Hz, 1H), 7.31 (s, 1H), 7.27-7.17 (m, 3H), 7.15-7.10 (m, 1H), 6.91 (d, J = 7.2 Hz, 1H), 6.13 (t, J = 4.8 Hz, 1H), 2.86 (d, J = 8.0 Hz, 2H), 2.57 (s, 3H), 2.46-2.39 (m, 2H) ppm; ^{13}C NMR (100 MHz, $CDCl_3$) δ 145.6, 142.1, 139.0, 136.9, 134.3, 132.6, 130.5, 129.5, 127.9, 127.6, 126.8, 126.5, 125.2, 118.5, 111.3, 28.2, 23.6, 20.7 ppm; HRMS (EI) (m/z) [M] $C_{18}H_{15}N$ calcd. for 245.1204, found 245.1204.

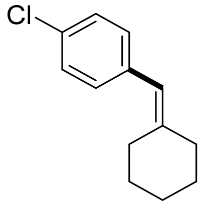

1-chloro-4-(cyclohexylidenemethyl)benzene (3du)

According to **Procedure 5**, yield: 26 mg, 63%; colorless oil; ^{1}H NMR (400 MHz, $CDCl_3$) δ 7.28-7.24 (m, 2H), 7.14-7.09 (m, 2H), 6.16 (s, 1H), 2.36-2.30 (m, 2H), 2.27-2.22 (m, 2H), 1.68-1.51 (m, 6H) ppm; ^{13}C NMR (100 MHz, $CDCl_3$) δ 144.4, 137.0, 131.6, 130.4, 128.3, 120.9, 37.8, 29.6, 28.7, 28.0, 26.8 ppm; HRMS (EI) (m/z) [M] $C_{13}H_{15}{}^{35}Cl$ calcd. for 206.0862, found 206.0861.

Data are consistent with the literature.[30]

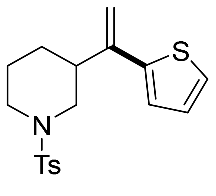

3-(1-(thiophen-2-yl)vinyl)-1-tosylpiperidine (3uv)

According to **Procedure 5**, yield: 46 mg, 67%; colorless oil; ^{1}H NMR (400 MHz, $CDCl_3$) δ 7.66-7.61 (m, 2H), 7.34-7.29 (m, 2H), 7.18 (dd, J = 5.2, 1.2 Hz, 1H), 7.18 (dd, J = 4.0, 1.2 Hz, 1H); 6.99 (dd, J = 4.8, 4.0 Hz, 1H), 5.43 (s, 1H), 4.89 (d, J = 0.8 Hz, 1H), 3.99-3.92 (m, 1H), 3.85-3.78 (m, 1H), 2.85-2.74 (m,

1H), 2.43 (s, 3H), 2.29 (td, *J* = 12.0, 3.2 Hz, 1H), 2.15 (t, *J* = 11.4 Hz, 1H), 1.99-1.90 (m, 1H), 1.85-1.68 (m, 2H), 1.33-1.21 (m, 1H) ppm; ^{13}C NMR (100 MHz, $CDCl_3$) δ 144.6, 143.5, 143.2, 133.7, 129.8, 127.8, 127.7, 124.6, 123.6, 110.9, 51.9, 46.8, 40.8, 29.8, 25.1, 21.7 ppm; HRMS (ESI) (m/z) $[M+H]^+$ $C_{18}H_{22}NO_2S_2$ calcd. for 348.1086, found 348.1086.

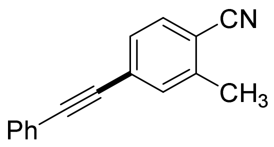

2-methyl-4-(phenylethynyl)benzonitrile (3lw)

According to **Procedure 5**, yield: 19 mg, 44%; white solid, m.p. 104-105 °C; ^{1}H NMR (400 MHz, $CDCl_3$) δ 7.57 (d, *J* = 8.0 Hz, 1H), 7.55-7.50 (m, 2H), 7.48 (s, 1H), 7.41 (d, *J* = 8.0 Hz, 1H), 7.40-7.34 (m, 3H), 2.55 (s, 3H) ppm; ^{13}C NMR (100 MHz, $CDCl_3$) δ 142.1, 133.2, 132.5, 131.9, 129.4, 129.2, 128.6, 128.1, 122.5, 118.0, 112.2, 93.3, 88.1, 20.5 ppm; HRMS (EI) (m/z) [M] $C_{16}H_{11}N$ calcd. for 217.0891, found 217.0891.

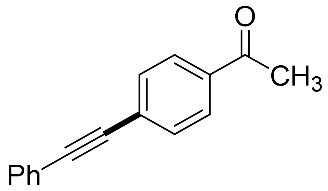

1-(4-(phenylethynyl)phenyl)ethan-1-one (3fw)

According to **Procedure 5**, yield: 15 mg, 35%; pale yellow solid, m.p. 104-105 °C; ^{1}H NMR (400 MHz, $CDCl_3$) δ 7.97-7.92 (m, 2H), 7.63-7.58 (m, 2H), 7.57-7.52 (m, 2H), 7.39-7.34 (m, 3H), 2.62 (s, 3H) ppm; ^{13}C NMR (100 MHz, $CDCl_3$) δ 197.5, 136.3, 131.9, 131.8, 129.0, 128.6, 128.42, 128.35, 122.8, 92.9, 88.8, 26.8 ppm; HRMS (EI) (m/z) [M] $C_{16}H_{12}O$ calcd. for 220.0888, found 220.0886.

Data are consistent with the literature.[31]

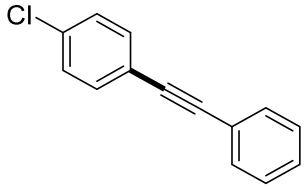

1-chloro-4-(phenylethynyl)benzene (3dw)

According to **Procedure 5**, yield: 17 mg, 41%; pale yellow solid, m.p. 82-83 °C; ^{1}H NMR (400 MHz, $CDCl_3$) δ 7.55-7.49 (m, 2H), 7.47-7.43 (m, 2H), 7.39-7.30 (m, 5H) ppm; ^{13}C NMR (100 MHz, $CDCl_3$) δ 134.4, 133.0, 131.8, 128.8, 128.6, 128.5, 123.1, 121.9, 90.5, 88.4 ppm; HRMS (EI) (m/z) [M] $C_{14}H_9{}^{35}Cl$ calcd. for 212.0393, found 212.0389.

Data are consistent with the literature.[32]

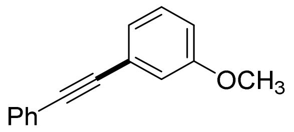

1-methoxy-3-(phenylethynyl)benzene (3aew)

According to **Procedure 5**, yield: 14 mg, 33%; pale yellow oil; ^{1}H NMR (400 MHz, $CDCl_3$) δ 7.56-7.52 (m, 2H), 7.38-7.32 (m, 3H), 7.29-7.23 (m, 1H), 7.16-7.12 (m, 1H), 7.08-7.06 (m, 1H), 6.92-6.88 (m, 1H), 3.83 (s, 3H) ppm; ^{13}C NMR (100 MHz, $CDCl_3$) δ 159.5, 131.8, 129.6, 128.50, 128.45, 124.4, 124.3, 123.3, 116.5, 115.1, 89.4, 89.3, 55.5 ppm; HRMS (EI) (m/z) [M] $C_{15}H_{12}O$ calcd. for 208.0888, found 208.0882.

Data are consistent with the literature.[33]

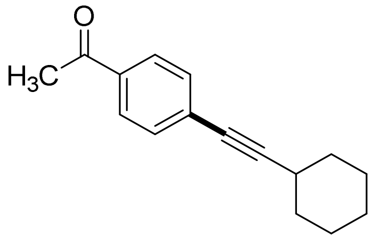

1-(4-(cyclohexylethynyl)phenyl)ethan-1-one (3fx)

According to **Procedure 5**, yield: 11 mg, 29%; colorless oil; ^{1}H NMR (400 MHz, $CDCl_3$) δ 7.88-7.83 (m, 2H), 7.48-7.43 (m, 2H), 2.65-2.58 (m, 1H), 2.58 (s, 3H), 1.93-1.84 (m, 2H), 1.80-1.71 (m, 2H), 1.59-1.47 (m, 3H), 1.42-1.30 (m, 3H) ppm; ^{13}C NMR (100 MHz, $CDCl_3$) δ 197.5, 135.7, 131.8, 129.4, 128.3, 98.5, 80.2, 32.6, 29.9, 26.7, 26.0, 25.0 ppm; HRMS (EI) (m/z) [M] $C_{15}H_{12}$ calcd. for 192.0934, found 192.0929.

Data are consistent with the literature.[34]

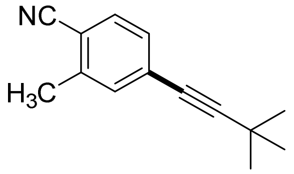

4-(3,3-dimethylbut-1-yn-1-yl)-2-methylbenzonitrile (3ly)

According to **Procedure 5**, yield: 15 mg, 37%; colorless oil; ^{1}H NMR (400 MHz, $CDCl_3$) δ 7.49 (d, J = 8.0, 1H), 7.32 (s, 1H), 7.25 (d, J = 8.4, 1H), 2.50 (s, 3H), 1.31 (s, 9H) ppm; ^{13}C NMR (100 MHz, $CDCl_3$) δ 141.8, 133.3, 132.3, 129.4, 129.0, 118.2, 111.4, 103.0, 78.3, 30.9, 20.4, 8.4 ppm; HRMS (EI) (m/z) [M] $C_{14}H_{15}N$ calcd. for 197.1204, found 197.1203.

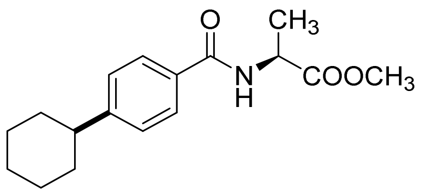

methyl (4-cyclohexylbenzoyl)-*L*-alaninate (3afa)

According to **Procedure 5**, yield: 83 mg, 91%; white solid, m.p. 111-112 °C; ^{1}H NMR (400 MHz, $CDCl_3$) δ 7.73 (d, *J* = 8.4 Hz, 2H), 7.27 (d, *J* = 8.0 Hz, 2H), 6.72 (brs, 1H), 4.87-4.73 (m, 1H), 3.79 (s, 3H), 2.60-2.50 (m, 1H), 1.92-1.81 (m, 4H), 1.79-1.72 (m, 1H), 1.51 (d, *J* = 7.2 Hz, 3H), 1.45-1.32 (m, 4H), 1.30-1.20 (m, 1H) ppm; ^{13}C NMR (100 MHz, $CDCl_3$) δ 173.9, 166.9, 152.3, 131.6, 127.24, 127.17, 52.7, 48.5, 44.7, 34.3, 26.9, 26.2, 18.9 ppm; HRMS (ESI) (m/z) $[M+H]^+$ $C_{17}H_{24}NO_3$ calcd. for 290.1751, found 290.1755.

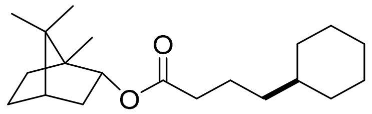

(1*S*,2*R*,4*S*)-1,7,7-trimethylbicyclo[2.2.1]heptan-2-yl 4-cyclohexylbutanoate (3aga)

According to **Procedure 5**, yield: 23 mg, 38%; colorless oil; ^{1}H NMR (400 MHz, $CDCl_3$) δ 4.91-4.85 (m, 1H), 2.39-2.31 (m, 1H), 2.29 (t, *J* = 7.2 Hz, 2H), 1.99-1.90 (m, 1H), 1.78-1.57 (m, 9H), 1.34-1.08 (m, 8H), 0.98-0.84 (m, 9H), 0.82 (s, 3H) ppm; ^{13}C NMR (100 MHz, $CDCl_3$) δ 174.4, 79.7, 48.9, 47.9, 45.1, 37.5, 37.1, 37.0, 35.2, 33.4, 28.2, 27.3, 26.8, 26.5, 22.7, 19.9, 19.0, 13.7 ppm; HRMS (ESI) (m/z) $[M+H]^+$ $C_{20}H_{34}O_2$ calcd. for 306.2559, found 306.2554.

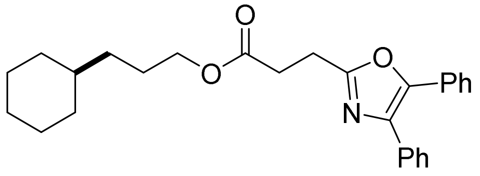

3-cyclohexylpropyl 3-(4,5-diphenyloxazol-2-yl)propanoate (3aha)

According to **Procedure 5**, yield: 21 mg, 25%; colorless oil; ^{1}H NMR (400 MHz, $CDCl_3$) δ 7.66-7.62 (m, 2H), 7.59-7.55 (m, 2H), 7.39-7.28 (m, 6H), 4.10 (d, *J* = 6.8 Hz, 2H), 3.19 (d, *J* = 7.2 Hz, 2H), 2.91 (d, *J* = 7.2 Hz, 2H), 1.70-1.58 (m, 7H), 1.25-1.05 (m, 6H), 0.90-0.78 (m, 2H) ppm; ^{13}C NMR (100 MHz, $CDCl_3$) δ 172.2, 162.0, 145.5, 135.3, 132.6, 129.2, 128.8, 128.7, 128.6, 128.2, 128.0, 126.6, 65.5, 37.4, 33.6, 33.4, 31.4, 26.7, 26.5, 26.1, 23.7 ppm; HRMS (ESI) (m/z) $[M]^+$ $C_{27}H_{32}NO_3$ calcd. for 418.2377, found 418.2382.

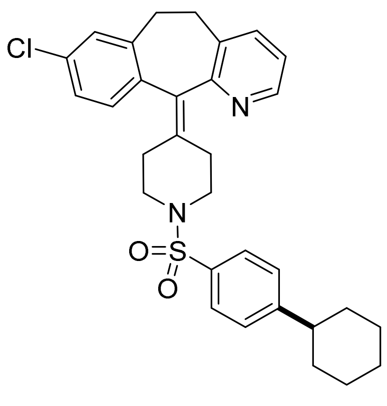

8-chloro-11-(1-((4-cyclohexylphenyl)sulfonyl)piperidin-4-ylidene)-6,11-dihydro-5*H*-benzo[5,6]cyclohepta[1,2-*b*]pyridine (3aia)

According to **Procedure 5**, yield: 65 mg, 61%; pale yellow oil; ^{1}H NMR (400 MHz, $CDCl_3$) δ 8.35 (d, *J* = 8.4, 1.2 Hz, 1H), 7.68-7.62 (m, 2H), 7.40 (dd, *J* = 7.6, 1.2 Hz, 1H), 7.34 (d, *J* = 8.0 Hz, 2H), 7.13 (d, *J* = 2.0 Hz, 1H), 7.11-7.05 (m, 2H), 7.00 (d, *J* = 8.0 Hz, 1H), 3.32-3.12 (m, 4H), 3.09-2.98 (m, 2H), 2.84-2.68 (m, 2H), 2.65-2.55 (m, 2H), 2.54-2.46 (m, 1H), 2.38-2.26 (m, 2H), 1.94-1.83 (m, 4H), 1.81-1.73 (m, 1H), 1.50-1.36 (m, 4H), 1.32-1.25 (m, 1H) ppm; ^{13}C NMR (100 MHz, $CDCl_3$) δ 156.8, 153.5, 146.8, 139.6, 137.7, 137.5, 136.0, 134.9, 133.8, 133.5, 133.2, 130.5, 129.2, 127.9, 127.7, 126.3, 122.5, 47.45, 47.43, 44.7, 34.25, 34.21, 31.7, 31.5, 30.3, 30.0, 26.8, 26.1 ppm; HRMS (ESI) (m/z) $[M+H]^+$ $C_{31}H_{34}{}^{35}ClN_2O_2S$ calcd. for 533.2024, found 533.2030.

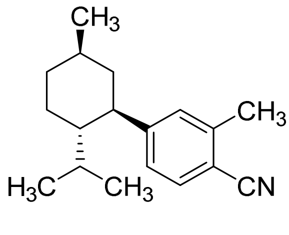

4-((1*R*,2*S*,5*R*)-2-isopropyl-5-methylcyclohexyl)-2-methylbenzonitrile (3lz)

According to **Procedure 5**, yield: 13 mg, 25%; according to **Procedure 6**, yield: 29 mg, 57%; colorless oil, dr > 30:1; ^{1}H NMR (400 MHz, $CDCl_3$) δ 7.50 (d, *J* = 7.6 Hz, 1H), 7.09 (s, 1H), 7.06 (d, *J* = 8.0 Hz, 1H), 2.52 (s, 3H), 2.45 (dt, *J* = 3.6, 11.6 Hz, 1H), 1.85-1.70 (m, 3H), 1.50-1.42 (m, 2H), 1.37-1.31 (m, 1H), 1.13-0.97 (m, 3H), 0.89 (d, *J* = 6.4 Hz, 3H), 0.80 (d, *J* = 6.8 Hz, 3H), 0.66 (d, *J* = 7.2 Hz, 3H) ppm; ^{13}C NMR (100 MHz, $CDCl_3$) δ 152.5, 142.1, 132.7, 129.6, 125.6, 118.7, 110.1, 48.4, 47.3, 45.0, 35.2, 33.2, 27.8, 24.5, 22.6, 21.6, 20.7, 15.5 ppm; HRMS (EI) (m/z) [M] $C_{18}H_{25}N$ calcd. for 255.1987, found 255.1981.

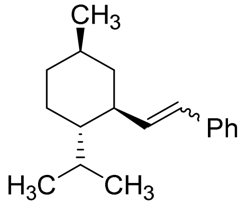

(2-((1*S*,2*S*,5*R*)-2-isopropyl-5-methylcyclohexyl)vinyl)benzene (3aaz)

According to **Procedure 5**, yield: 17 mg, 35%; according to **Procedure 6**, yield: 37 mg, 57%; colorless oil, as a mixture of isomers, dr > 30:1, *E*/*Z* = 9:1; ^{1}H NMR (400 MHz, $CDCl_3$) δ 7.37-7.33 (m, 1.80H, the *E* isomer), 7.32-7.26 (m, 2H), 7.26-7.22 (m, 0.20H, the *Z* isomer), 7.21-7.15 (m, 1H), 6.39 (d, *J* = 12.0 Hz, 0.10H, the *Z* isomer), 6.34 (d, *J* = 15.6 Hz, 0.90H, the *E* isomer), 6.01 (dd, *J* = 15.6, 9.6 Hz, 0.90H, the *E* isomer), 5.40 (dd, *J* = 11.6, 10.2 Hz, 0.10H, the *E* isomer), 2.57-2.45 (m, 0.10H, the *Z* isomer), 2.13-2.02 (m, 0.90H, the *E* isomer), 1.93-1.83 (m, 1H), 1.80-1.61 (m, 3H), 1.48-1.37 (m, 1H), 1.13-0.91 (m, 4H), 0.91-0.87 (m, 6H), 0.84 (d, *J* = 7.2 Hz, 0.30H, the *Z* isomer), 0.75 (d, *J* = 6.8 Hz, 2.70H, the *E* isomer) ppm; ^{13}C NMR (100 MHz, $CDCl_3$) the *E* isomer δ 138.2, 136.2, 128.8, 128.6, 126.8, 126.1, 47.6, 45.4, 43.3, 35.3, 32.6, 28.5, 24.4, 22.8, 21.6, 15.7 ppm; HRMS (EI) (m/z) [M] $C_{18}H_{26}$ calcd. for 242.2029, found 242.2026 (the *Z* isomer) and 242.2019 (the *E* isomer).

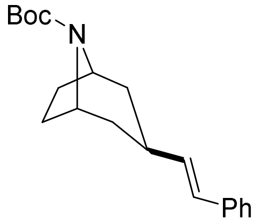

***tert*-butyl (1*R*,3*s*,5*S*)-3-styryl-8-azabicyclo[3.2.1]octane-8-carboxylate (3aaaa)**

According to **Procedure 6**, yield: 61 mg, 97%; dr > 30:1, *E*/*Z* > 30:1, colorless oil; ^{1}H NMR (400 MHz, $CDCl_3$) δ 7.37-7.26 (m, 4H), 7.22-7.16 (m, 1H), 6.37 (d, *J* = 16.0 Hz, 1H), 6.04 (dd, *J* = 16.0, 7.2 Hz, 1H), 4.35-4.12 (m, 2H), 2.75-2.61 (m, 1H), 2.05-1.91 (m, 2H), 1.76-1.58 (m, 6H), 1.48 (s, 9H) ppm; ^{13}C NMR (100 MHz, $CDCl_3$) δ 153.6, 137.7, 134.6, 128.7, 128.6, 127.2, 126.2, 79.3, 53.8, 53.1, 37.8, 36.9, 32.6, 28.7, 28.0 ppm; HRMS (ESI) (m/z) $[M+Na]^+$ $C_{20}H_{27}NNaO_2$ calcd. for 336.1934, found 336.1934.

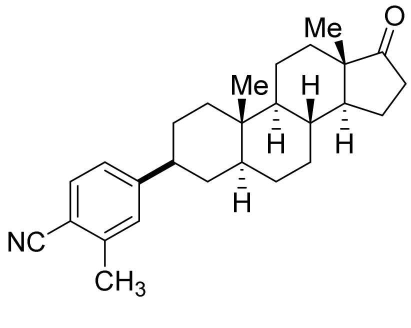

4-((8*R*,9*S*,10*S*,13*S*,14*S*)-10,13-dimethyl-17-oxohexadecahydro-1*H*-cyclopenta[*a*]phenanthren-3-yl)-2-methylbenzonitrile (3lab)

According to **Procedure 6**, yield: 64 mg, 82%; white solid, m.p. 154-155 °C; as a mixture of diastereomers, dr = 1:1; ^{1}H NMR (400 MHz, $CDCl_3$) δ 7.54-7.46 (m, 1H), 7.27-7.22 (m, 1H), 7.16-7.06 (m, 1H), 3.08-3.02 (m, 0.5H), 2.62-2.50 (m, 3.5H), 2.46-2.36 (m, 1H), 2.10-1.42 (m, 14H), 1.35-1.15 (m, 7H), 0.92-0.82 (m, 6H) ppm; ^{13}C NMR (100 MHz, $CDCl_3$) δ 221.4, 152.9, 151.2, 142.0, 141.7, 132.6, 132.4, 129.7, 128.9, 125.7, 125.0, 118.6, 110.2, 109.7, 54.9, 54.7, 51.7, 51.6, 51.5, 48.0, 47.93, 47.88,

47.0, 45.0, 43.6, 41.1, 38.9, 38.7, 36.9, 36.5, 36.4, 36.1, 36.04, 35.97, 35.93, 35.2, 35.1, 34.5, 33.1, 32.5, 31.8, 31.7, 31.6, 31.2, 31.0, 30.8, 29.5, 29.0, 28.6, 25.9, 24.7, 21.9, 21.8, 20.9, 20.6, 20.4, 20.1, 14.0, 13.9, 12.6, 12.4, 12.2 ppm; HRMS (EI) (m/z) [M] $C_{27}H_{35}NO$ calcd. for 389.2719, found 389.2709 and 389.2717.

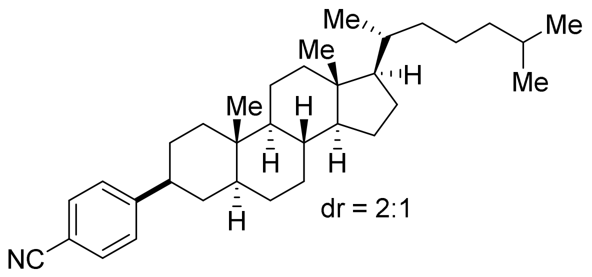

4-((5*S*,8*R*,9*S*,10*S*,13*R*,14*S*,17*R*)-10,13-dimethyl-17-((*R*)-6-methylheptan-2-yl)hexadecahydro-1H-cyclopenta[*a*]phenanthren-3-yl)benzonitrile (3jac)

According to **Procedure 6**, yield: 73 mg, 77%; white solid, m.p. 120-121 °C; as a mixture of diastereomers, dr = 2:1; ^{1}H NMR (400 MHz, $CDCl_3$) δ 7.53 (d, *J* = 8.4 Hz, 0.67H, the minor isomer), 7.50 (d, *J* = 8.4 Hz, 1.33H, the major isomer), 7.40 (d, *J* = 8.4 Hz, 0.67H, the minor isomer), 7.25 (d, *J* = 8.4 Hz, 1.33H, the major isomer), 3.07-3.00 (m, 0.33H, the minor isomer), 2.60-2.48 (m, 0.67H, the major isomer), 2.00-1.70 (m, 4H), 1.58-0.92 (m, 27H), 0.87-0.77 (m, 12H), 0.61 (s, 2H, the major isomer), 0.58 (s, 1H, the minor isomer) ppm; ^{13}C NMR (100 MHz, $CDCl_3$) δ 153.4 (the major isomer), 151.7 (the minor isomer), 132.3 (the major isomer), 132.0 (the minor isomer), 128.6 (the minor isomer), 127.8 (the major isomer), 119.4 (the minor isomer), 119.3 (the major isomer), 109.7 (the major isomer), 109.1 (the minor isomer), 56.7 (the major isomer), 56.6 (the minor isomer), 56.45 (the major isomer), 56.41 (the minor isomer), the following peaks are difficult to assign: 54.68, 47.01, 45.20, 42.75, 42.69, 41.12, 40.20, 40.08, 39.66, 39.64, 38.78, 37.14, 36.32, 36.30, 36.22, 35.95, 35.85, 35.68, 35.54, 34.42, 33.05, 32.22, 31.96, 29.61, 28.98, 28.95, 28.39, 28.36, 28.16, 28.14, 24.96, 24.34, 24.27, 24.01, 23.99, 22.96, 22.71, 21.19, 20.89, 18.83, 18.79, 12.61, 12.24, 12.22, 12.20 ppm; HRMS (EI) (m/z) [M] $C_{34}H_{51}N$ calcd. for 473.4016, found 473.4004 (the minor isomer) and 473.4006 (the major isomer).

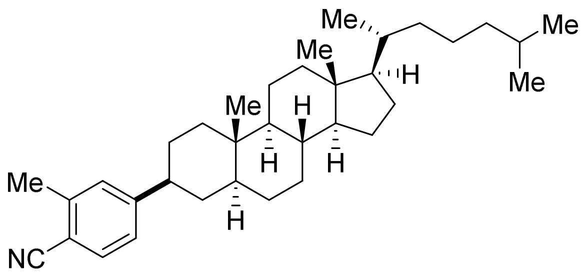

4-((3*S*,5*S*,8*R*,9*S*,10*S*,13*R*,14*S*,17*R*)-10,13-dimethyl-17-((*R*)-6-methylheptan-2-yl)hexadecahydro-1H-cyclopenta[*a*]phenanthren-3-yl)-2-methylbenzonitrile (3lac)

According to **Procedure 6**, yield: 46 mg, 93%; white solid, m.p. 138 °C; as a mixture of diastereomers, dr = 2:1; ^{1}H NMR (400 MHz, $CDCl_3$) δ 7.52 (d, *J* = 8.4 Hz, 0.33H, the minor isomer), 7.50 (d, *J* = 8.0 Hz,

0.67H, the major isomer), 7.27 (s, 0.33H, the minor isomer), 7.26 (d, *J* = 8.4 Hz, 0.33H, the minor isomer), 7.15 (s, 0.67H, the major isomer), 7.11 (d, *J* = 8.0 Hz, 0.67H, the major isomer), 3.09-3.02 (m, 0.33H, the minor isomer), 2.60-2.54 (m, 0.67H, the major isomer), 2.54 (s, 1H, the minor isomer), 2.52 (s, 2H, the major isomer), 2.02-.1.61 (m, 6H), 1.58-0.95 (m, 25H), 0.93-0.84 (m, 12H), 0.67 (s, 2H, the major isomer), 0.64 (s, 1H, the minor isomer) ppm; ^{13}C NMR (100 MHz, $CDCl_3$) δ 153.20 (the major isomer), 151.55 (the minor isomer), 141.97 (the major isomer), 141.60 (the minor isomer), 132.64 (the major isomer), 132.36 (the minor isomer), 129.77 (the minor isomer), 128.95 (the major isomer), 125.73 (the minor isomer), 125.06 (the major isomer), 118.71 (the minor isomer), 118.65 (the major isomer), 110.10 (the major isomer), 109.58 (the minor isomer), 56.70 (the major isomer), 56.60 (the minor isomer), 56.45 (the major isomer), 56.42 (the minor isomer), the following peaks are difficult to assign: 54.68, 47.03, 45.15, 42.75, 42.69, 41.12, 40.20, 40.09, 39.66, 39.64, 38.81, 37.02, 36.33, 36.31, 36.21, 35.95, 35.87, 35.69, 35.55, 34.51, 33.13, 32.23, 31.96, 29.62, 28.96, 28.39, 28.36, 28.16, 28.14, 24.88, 24.34, 24.27, 24.01, 23.99, 22.96, 22.71, 21.19, 20.89, 20.88, 20.66, 18.83, 18.80, 12.63, 12.24, 12.21, 12.20 ppm; HRMS (EI) (m/z) [M] $C_{35}H_{53}N$ calcd. for 487.4172, found 487.4163.

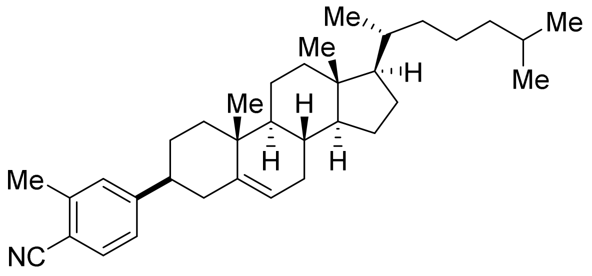

4-((8*S*,9*S*,10*R*,13*R*,14*S*,17*R*)-10,13-dimethyl-17-((*R*)-6-methylheptan-2-yl)-2,3,4,7,8,9,10,11,12,13,14,15,16,17-tetradecahydro-1*H*-cyclopenta[*a*]phenanthren-3-yl)-2-methylbenzonitrile (3lad)

According to **Procedure 6**, yield: 77 mg, 79%; pale yellow oil, as a mixture of diastereomers, dr = 1.5:1; ^{1}H NMR (400 MHz, $CDCl_3$) δ 7.52 (d, *J* = 8.0 Hz, 0.40H, the minor isomer), 7.47 (d, *J* = 8.4 Hz, 0.60H, the major isomer), 7.31 (s, 0.60H, the major isomer), 7.31-7.28 (m, 0.60H, the major isomer), 7.16 (s, 0.40H, the minor isomer), 7.14-7.10 (m, 0.40H, the minor isomer), 5.47-5.43 (m, 0.60H, the major isomer), 5.38-5.33 (m, 0.40H), 3.12-3.05 (m, 0.60H, the major isomer), 2.85-2.75 (m, 0.60H, the major isomer), 2.53 (s, 1.20H, the minor isomer), 2.49 (s, 1.80H, the major isomer), 2.44-2.35 (m, 1H), 2.18-2.14 (m, 0.40H, the minor isomer), 2.14-2.11 (m, 0.40H, the minor isomer), 2.06-1.96 (m, 2H), 1.89-1.80 (m, 1H), 1.75-1.10 (m, 19H), 1.08 (s. 3H), 1.05-0.95 (m, 4H), 0.93-0.88 (m, 3H), 0.87-0.83 (m, 6H), 0.70 (s, 1.20H, the minor isomer), 0.68 (s, 1.80H, the major isomer) ppm; ^{13}C NMR (100 MHz, $CDCl_3$) δ (difficult to assign every peak) 152.39, 152.24, 142.11, 141.93, 141.17, 140.82, 132.58, 132.00, 130.37, 128.75, 126.23, 124.82, 122.15, 120.77, 118.61, 118.48, 110.18, 109.51, 77.35, 77.03, 76.71, 56.83, 56.80, 56.18, 50.45, 49.91, 45.94, 42.33, 40.13, 39.82, 39.73, 39.61, 39.53, 38.67, 37.25, 36.89, 36.20, 35.83, 35.21, 32.97, 32.00, 31.93, 31.88, 31.78, 29.65, 28.26, 28.02, 24.31, 24.25, 23.86, 22.84, 22.58, 20.96, 20.70, 20.65, 20.55, 19.71, 19.60, 18.75, 18.71, 11.90 ppm; HRMS (EI) (m/z) [M] $C_{35}H_{51}N$ calcd. for 485.4016, found 473.4007 (the major isomer) and 473.4004 (the minor isomer).

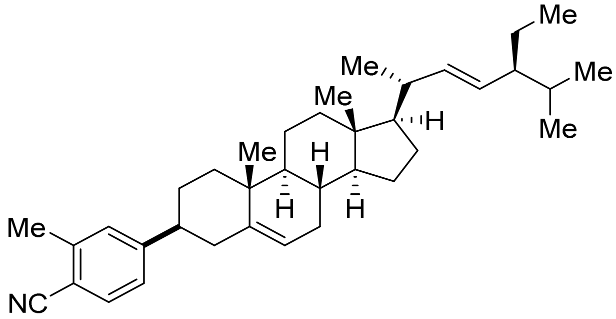

4-((3*S*,8*S*,9*S*,10*R*,13*R*,14*S*,17*R*)-17-((2*R*,5*S*,*E*)-5-ethyl-6-methylhept-3-en-2-yl)-10,13-dimethyl-2,3,4,7,8,9,10,11,12,13,14,15,16,17-tetradecahydro-1*H*-cyclopenta[*a*]phenanthren-3-yl)-2-methylbenzonitrile (3lae)

According to **Procedure 6**, yield: 78 mg, 76%; white solid, m.p. 149-150 °C; as a mixture of diastereomers, dr = 1:1; ^{1}H NMR (400 MHz, $CDCl_3$) δ 7.55-7.43 (m, 1H), 7.31 (s, 1H), 7.19-7.10 (m, 1H), 5.50-5.30 (m, 1H), 5.20-4.95 (m, 2H), 3.12-3.03 (m, 0.50H), 2.85-2.73 (m, 0.50H), 2.53 (s, 1.50H), 2.50 (s, 1.50H), 2.45-2.33 (m, 1H), 2.20-2.10 (m, 1H), 2.07-1.94 (m, 3H), 1.75-1.41 (m, 11H), 1.25-1.00 (m, 15H), 0.88-0.75 (m, 9H), 0.71 (d, *J* = 5.2 Hz, 3H) ppm; ^{13}C NMR (100 MHz, $CDCl_3$) δ 152.51, 152.35, 142.24, 142.06, 141.31, 140.94, 138.46, 132.71, 132.14, 130.49, 129.43, 128.88, 126.35, 124.95, 122.27, 120.89, 118.74, 118.61, 110.32, 109.65, 77.48, 77.16, 76.84, 57.06, 57.02, 56.12, 56.09, 51.39, 51.38, 50.60, 50.05, 46.07, 42.35, 40.66, 40.26, 39.84, 39.75, 38.79, 37.40, 37.04, 35.34, 33.11, 32.12, 32.03, 31.91, 29.78, 29.07, 28.36, 25.55, 24.51, 24.45, 21.38, 21.36, 21.24, 21.08, 20.81, 20.79, 20.68, 19.84, 19.73, 19.14, 12.40, 12.22 ppm; HRMS (EI) (m/z) [M] $C_{37}H_{53}N$ calcd. for 511.4178, found 511.4161.

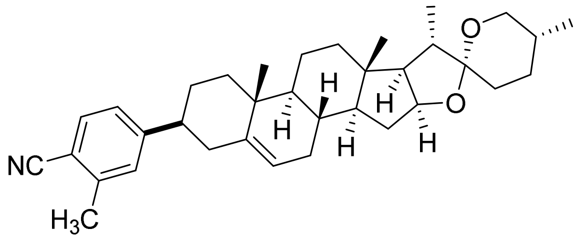

2-methyl-4-((5'*R*,6*aR*,6*bS*,8*aS*,8*bR*,9*S*,10*R*,11*aS*,12*aS*,12*bS*)-5',6*a*,8*a*,9-tetramethyl-1,3,3',4,4',5,5',6,6*a*,6*b*,6',7,8,8*a*,8*b*,9,11*a*,12,12*a*,12*b*-icosahydrospiro[naphtho[2',1':4,5]indeno[2,1-*b*]furan-10,2'-pyran]-4-yl)benzonitrile (3laf)

According to **Procedure 6**, yield: 83 mg, 81%; white solid, m.p. 234 °C; as a mixture of diastereomers, dr = 1:1; ^{1}H NMR (400 MHz, $CDCl_3$) δ 7.51 (d, *J* = 8.0 Hz, 0.5H), 7.46 (d, *J* = 8.4 Hz, 0.5H), 7.32-7.27 (m, 1H), 7.16 (s, 0.50H), 7.12 (d, *J* = 8.0 Hz, 0.50H), 5.47-5.43 (m, 0.50H), 5.38-5.32 (m, 0.50H), 4.47-4.36 (m, 1H), 3.52-3.43 (m, 1H), 3.42-3.34 (m, 1H), 3.12-3.04 (m, 0.50H), 2.84-2.73 (m, 0.50H), 2.52 (s, 1.5H), 2.49 (s, 1.5H), 2.44-2.34 (m, 1H), 2.18-1.82 (m, 5H), 1.80-1.12 (m, 18H), 1.09 (s, 3H), 0.99-0.94 (m, 3H), 0.83-0.76 (m, 6H) ppm; ^{13}C NMR (100 MHz, $CDCl_3$) δ 152.4, 152.2, 142.2, 142.1, 141.3, 141.0, 132.7, 132.1, 130.4, 128.9, 126.3, 124.9, 122.0, 120.6, 118.7, 118.6, 110.3, 109.7, 109.4, 81.0, 67.0,

62.3, 62.2, 56.7, 56.6, 50.5, 49.9, 46.0, 41.7, 40.39, 40.38, 40.2, 39.93, 39.85, 39.7, 38.7, 37.5, 37.1, 35.3, 33.1, 32.24, 32.19, 31.98, 31.93, 31.5, 31.4, 30.4, 29.7, 28.9, 28.3, 20.9, 20.8, 20.7, 20.6, 19.8, 19.7, 17.3, 16.4, 14.67, 14.65 ppm; HRMS (EI) (m/z) [M] $C_{35}H_{47}NO_2$ calcd. for 513.3607, found 513.3609.

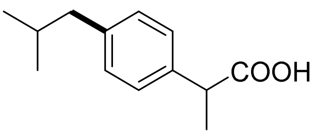

2-(4-isobutylphenyl)propanoic acid (3afn)

According to **Procedure 5**, yield: 12 mg, 29%; white solid, m.p. 76 ºC; ^{1}H NMR (400 MHz, DMSO-d_6) δ 12.22 (brs, 1H), 7.19-7.15 (m, 2H), 7.11-7.06 (m, 2H), 3.61 (q, *J* = 7.2 Hz, 1H), 2.40 (d, *J* = 7.2 Hz, 2H), 1.79 (hept, *J* = 6.8 Hz, 1H), 1.33 (d, *J* = 7.2 Hz, 3H), 0.84 ((d, *J* = 6.4 Hz, 6H) ppm; ^{13}C NMR (100 MHz, $CDCl_3$) δ 175.5, 139.5, 138.5, 129.0, 127.1, 44.3, 44.2, 29.6, 22.2, 18.5 ppm; HRMS (ESI) (m/z) $[M-H]^-$ $C_{13}H_{17}O_2$ calcd. for 205.1234, found 205.1237.

Data are consistent with the literature.[35]

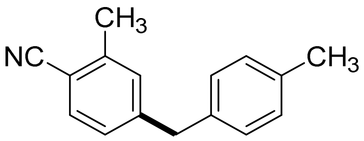

2-methyl-4-(4-methylbenzyl)benzonitrile (5)

According to **Procedure 5**, yield: 17 mg, 39%; colorless oil, ^{1}H NMR (400 MHz, CD_3CN) δ 7.50 (d, *J* = 8.0 Hz, 1H), 7.14-7.10 (m, 3H), 7.09-7.03 (m, 3H), 3.94 (s, 2H), 2.49 (s, 3H), 2.33 (s, 3H) ppm; ^{13}C NMR (100 MHz, CD_3CN) δ 147.0, 142.2, 136.6, 136.3, 132.8, 130.8, 129.5, 128.9, 126.9, 118.5, 110.5, 41.7, 21.2, 20.6 ppm; HRMS (EI) (m/z) [M] $C_{16}H_{15}N$ calcd. for 221.1199, found 221.1197.

5. Continuous Flow Experiments

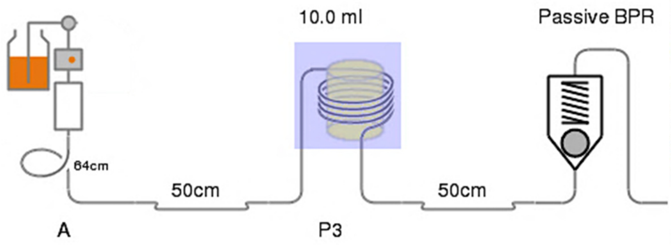

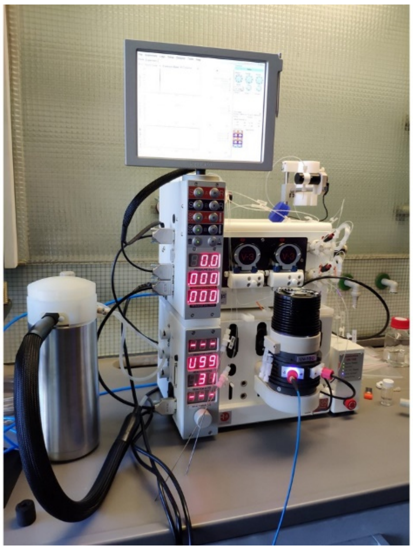

Figure S4. Schematic of the photochemical reaction setup (left). Picture of the setup (right).

To an oven-dried crimp cap vial (50 mL in volume) equipped with a magnetic stirring bar was added substrate **2a** and 4CzIPN. Then the reaction vial was sealed, degassed and backfilled with nitrogen. 2,6-lutidine, substrate **1j** and MeCN was added. The resulting mixture was bubbled with N_2 for 10 min. Afterwards, dtbbpy·$NiCl_2$ solution, prepared fresh for each reaction as per **Procedure 4**, was added under N_2 via a syringe to the vial. The resulting mixture was again bubbled with N_2 for another 10 min and degassed Et_3N (2 equiv. based on substrate **1j**) was added. A Vapourtec UV-150 Photochemical Reactor (R Series) was first primed with anhydrous MeCN (20 mL) under an N_2 atmosphere and then the reaction mixture was introduced and exposed to conditions described in Table S1 within a 10 mL PTFE coil reactor under 450 nm irradiation (60 W input power, 24 W radiant power) and collected in another closed crimp cap vial. The temperature of the coil was controlled precisely (±1 ^{o}C by a regulated stream of N_2 cooled by passing through a canister filled with dry ice), or heated by convection with an in-built electronic heating gun.

After the collection of all reaction mixture, the reaction mixture was transferred into a round-bottom flask and 1,3,5-trimethoxybenzene was added as an internal standard for calculating the ^{1}H NMR yield. Solvent was evaporated under reduced pressure and the residue was passed through a silica gel plug using 100 mL mixture of pentane and EtOAc (25% EtOAc in pentane) as eluent to give crude product **3ja**, which was quantified by ^{1}H NMR.

Table S1. Continuous Flow Experiments.

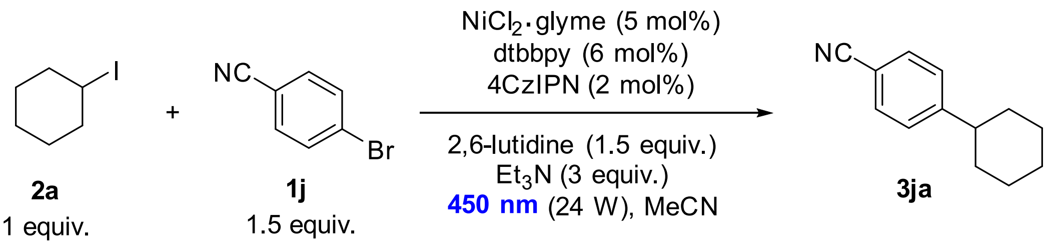

Entry	Amount **2a** (mmol)	Concentration (M)	Flow rate (mL/min)	R_T (min)	Conversion (%)	Product (%)	Productivity (mg h^{-1})
1	1.3	0.07	0.1	400[a]	70	65	23.5
2[b]	2.1	0.1	0.1	200[c]	88	38	44.3
3[d]	0.7	0.07	0.05	100	100	51	19.8
4[d]	1.3	0.03	0.05	200[c]	100	53	38.3
5	1.2	0.07	0.08	100	92	54	34.0
6	0.8	0.07	0.05	100	89	54	21.0
7[e]	1.0	0.07	0.10	100	100	**78**	60.7
8[e]	1.0	0.07	0.20	50	72	70	**108.9**

Unless otherwise specified, reactions were conducted at a controlled 25 °C and productivity was calculated assuming a single pass. [a] circulated four times, nominal productivity calculated based on the scale and overall reaction time. [b] 50 ˚C. [c] circulated two times, nominal productivity calculated based on the scale and overall reaction time. [d] 30 ˚C. [e] 1.0 equiv. **1j** as limiting reagent, 1.5 equiv. **2a**.

6. ^{1}H NMR AND ^{13}C NMR SPECTRA

^{1}H NMR of **1t** in $CDCl_3$

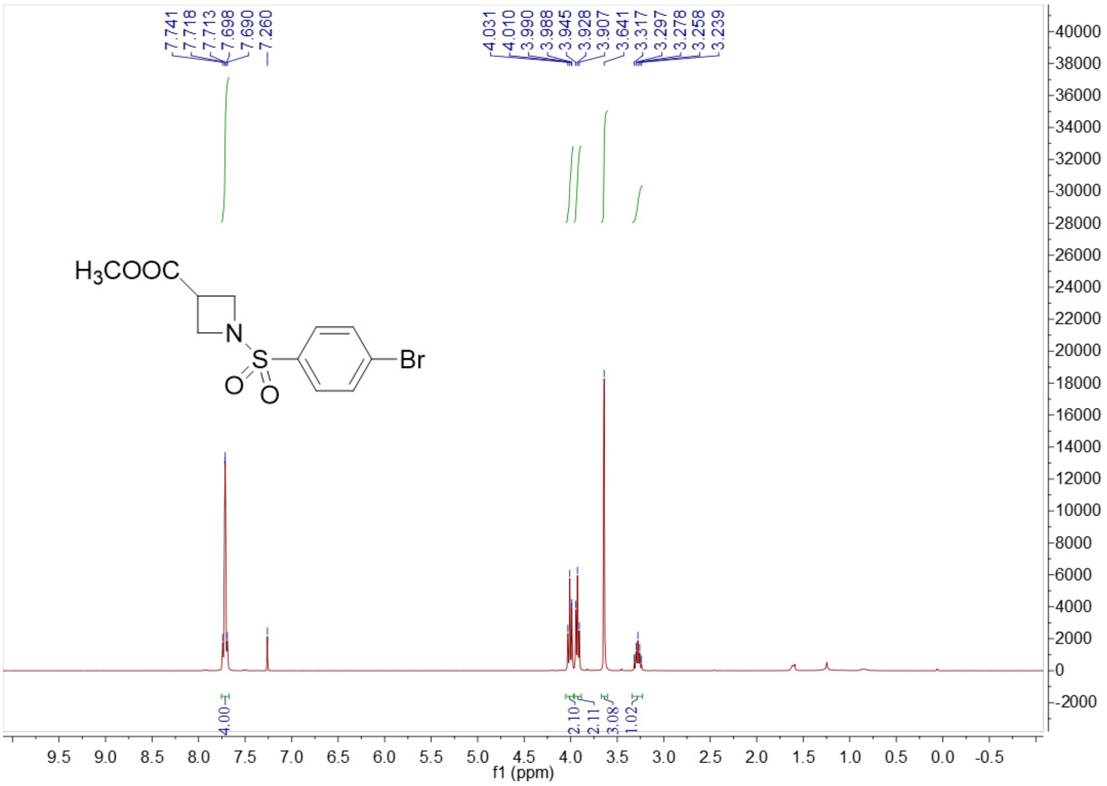

^{13}C NMR of **1t** in $CDCl_3$

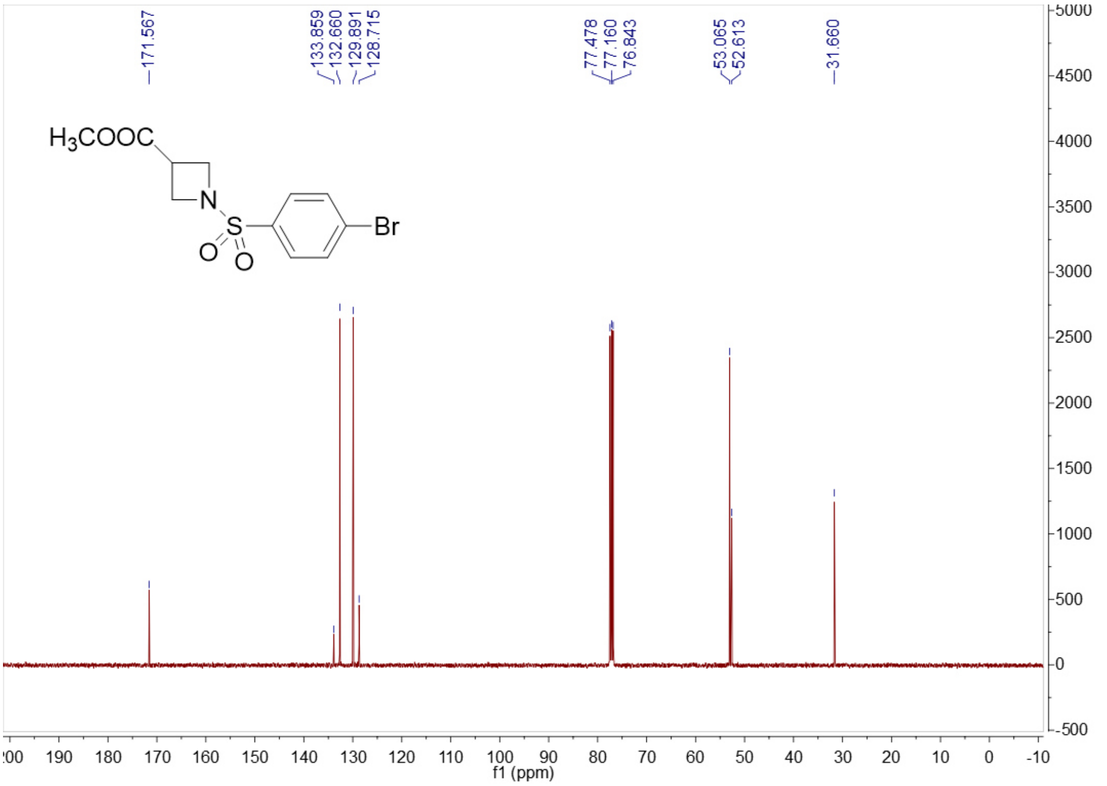

^{1}H NMR of **1af** in $CDCl_3$

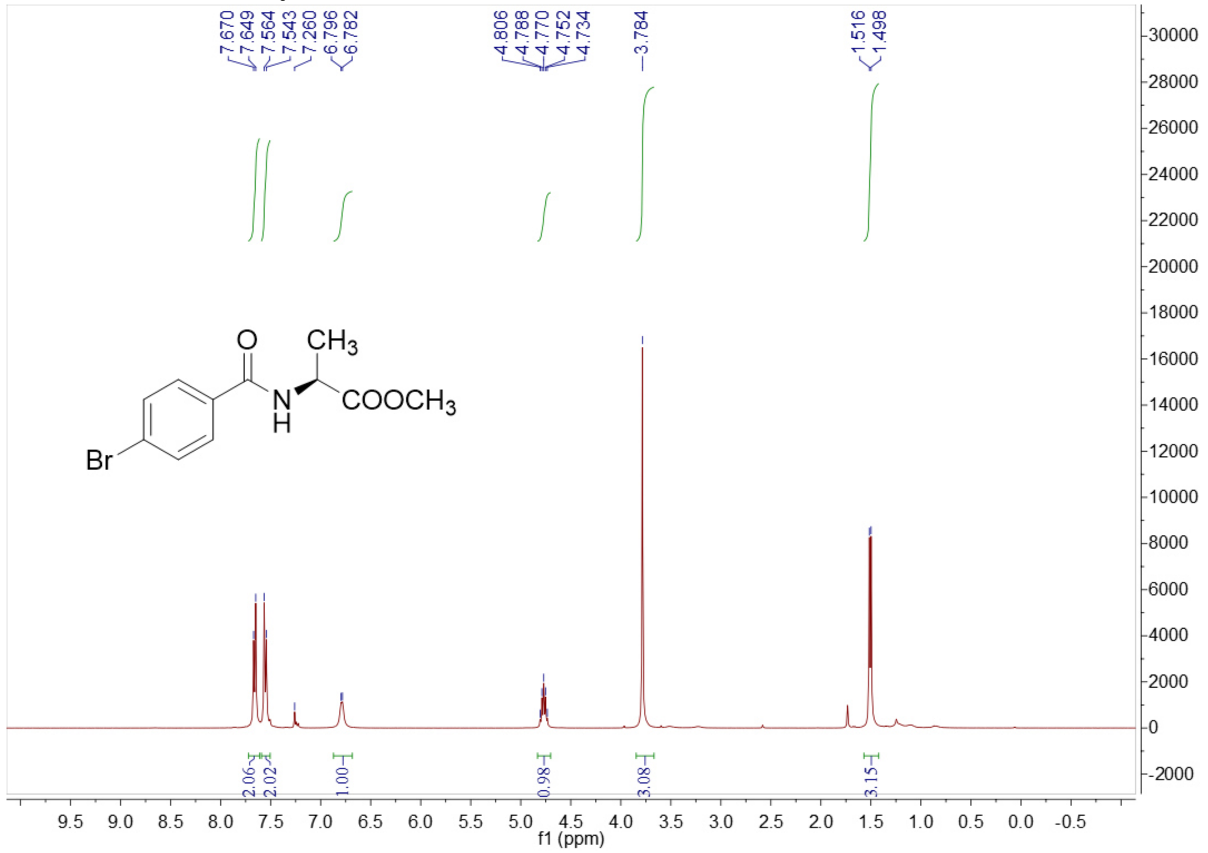

^{13}C NMR of **1af** in $CDCl_3$

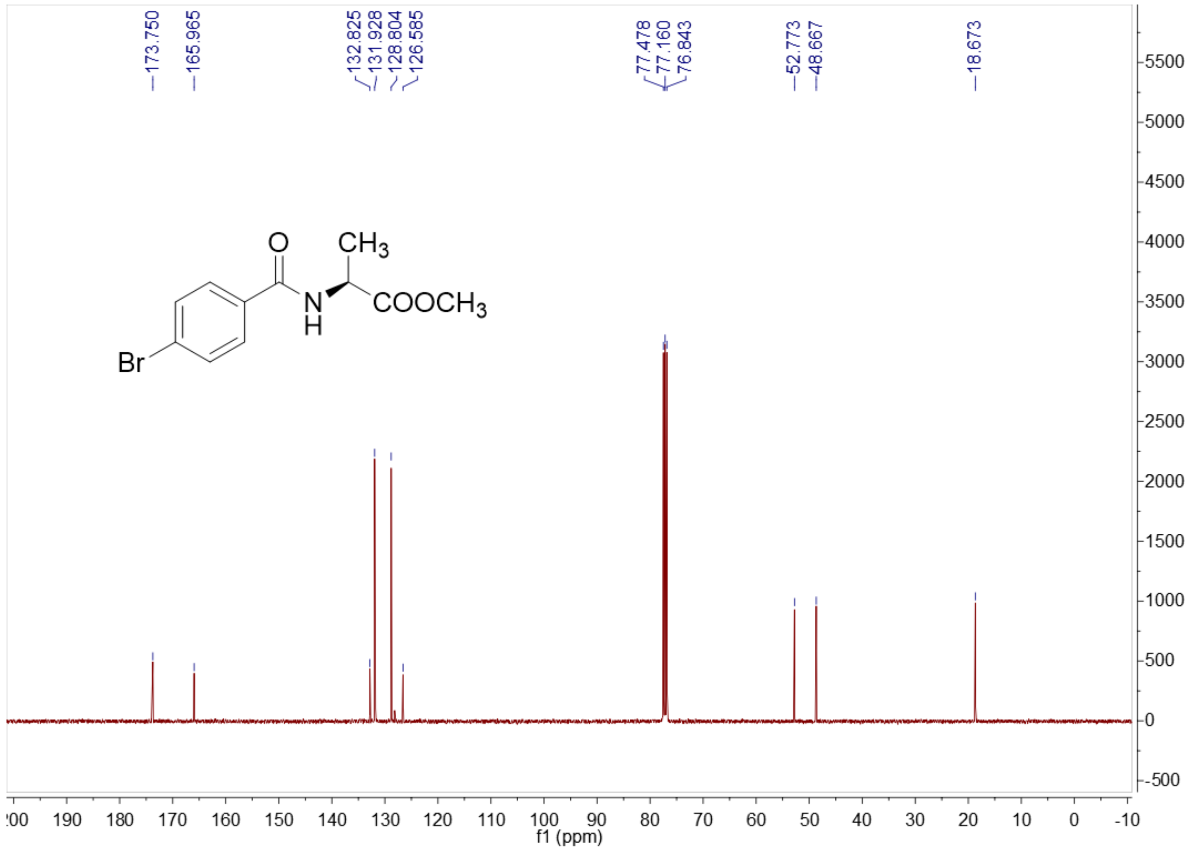

^{1}H NMR of **1ai** in $CDCl_3$

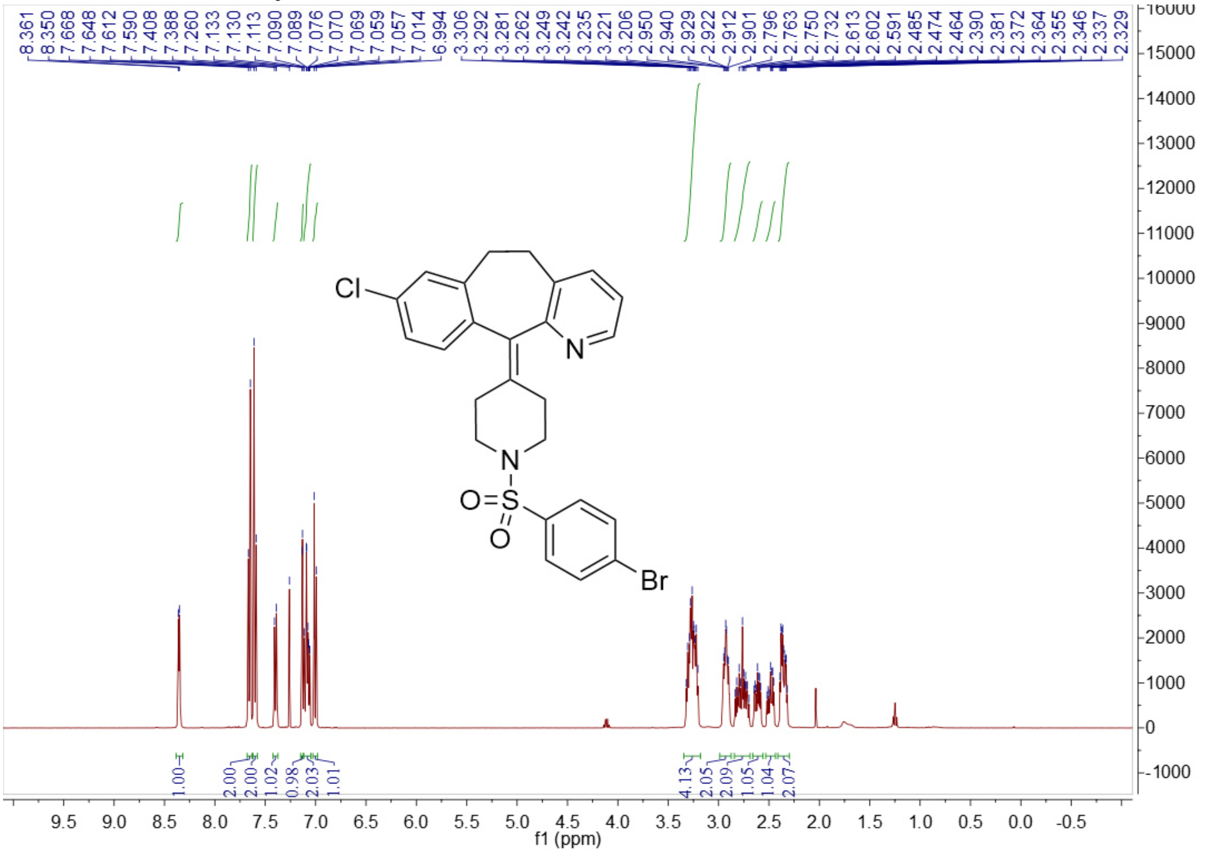

^{13}C NMR of **1ai** in $CDCl_3$

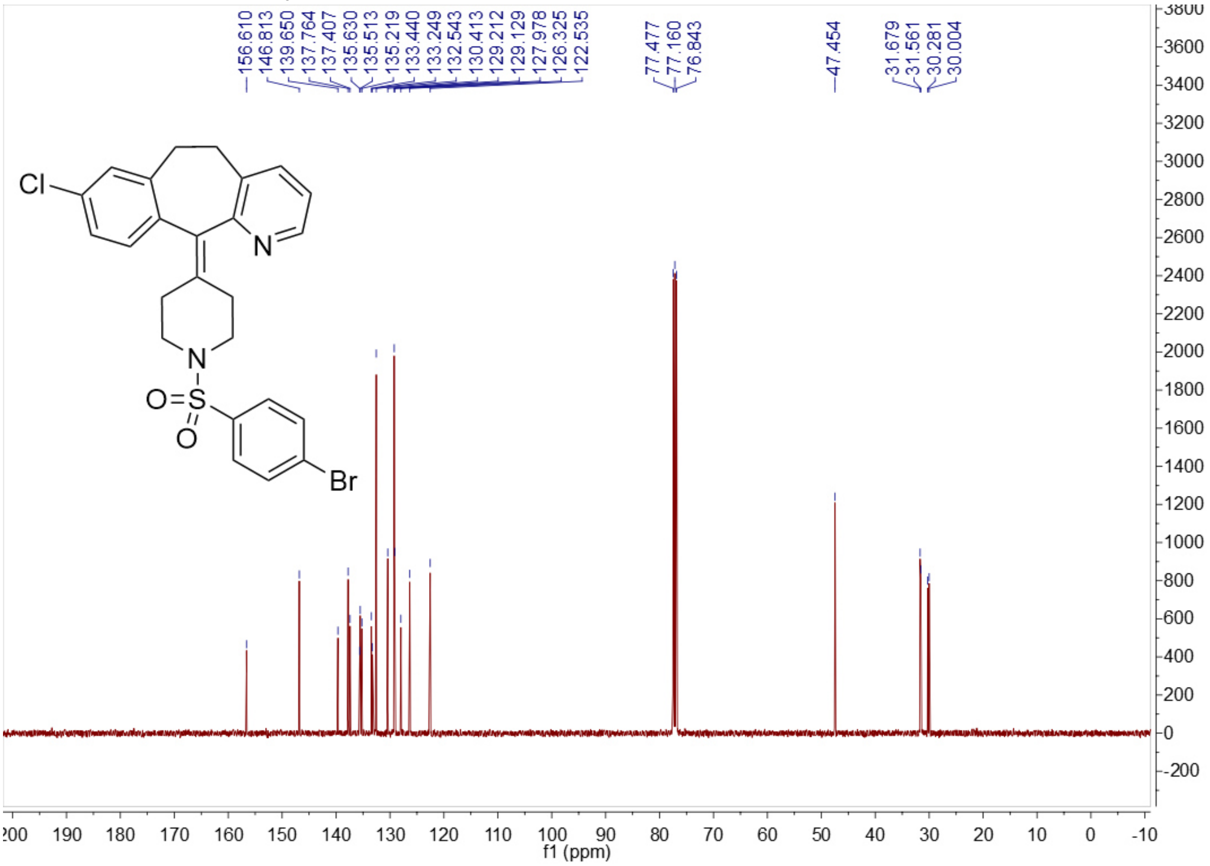

^{1}H NMR of **2c** in $CDCl_3$

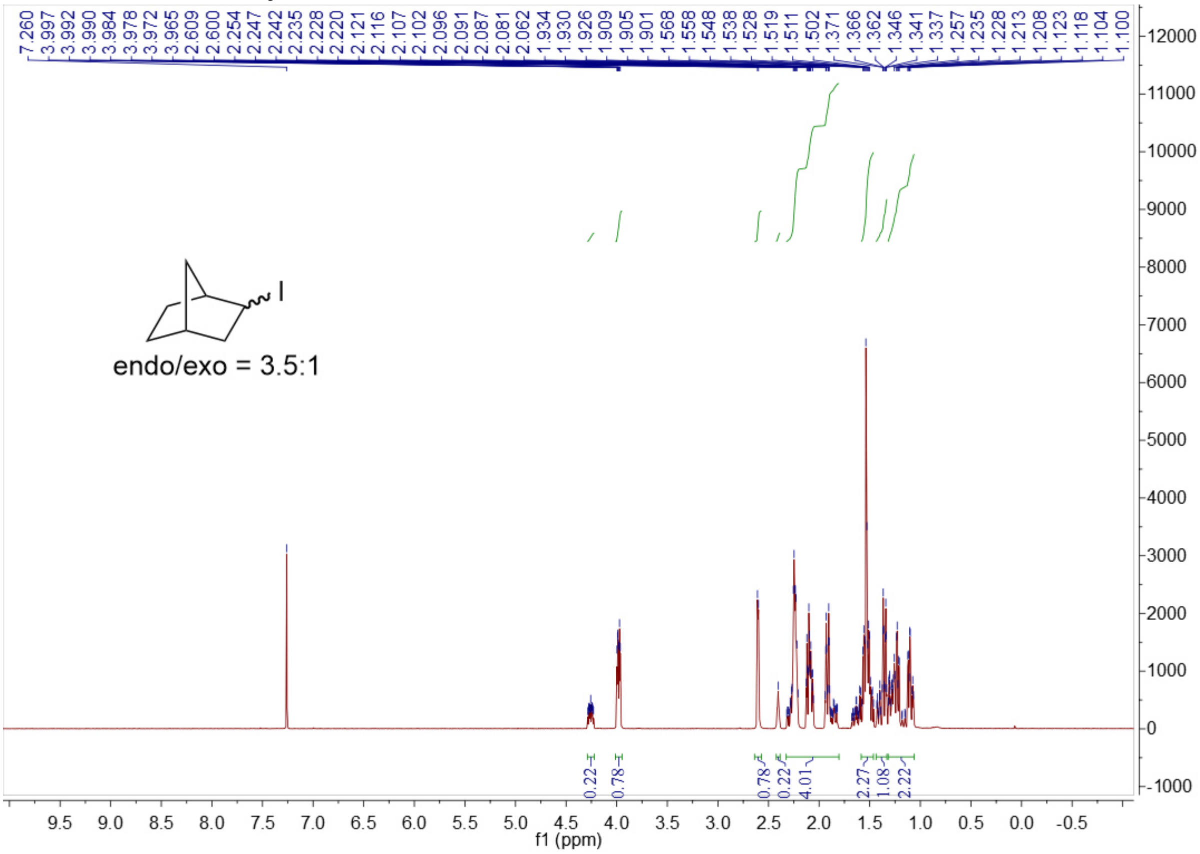

^{13}C NMR of **2c** in $CDCl_3$

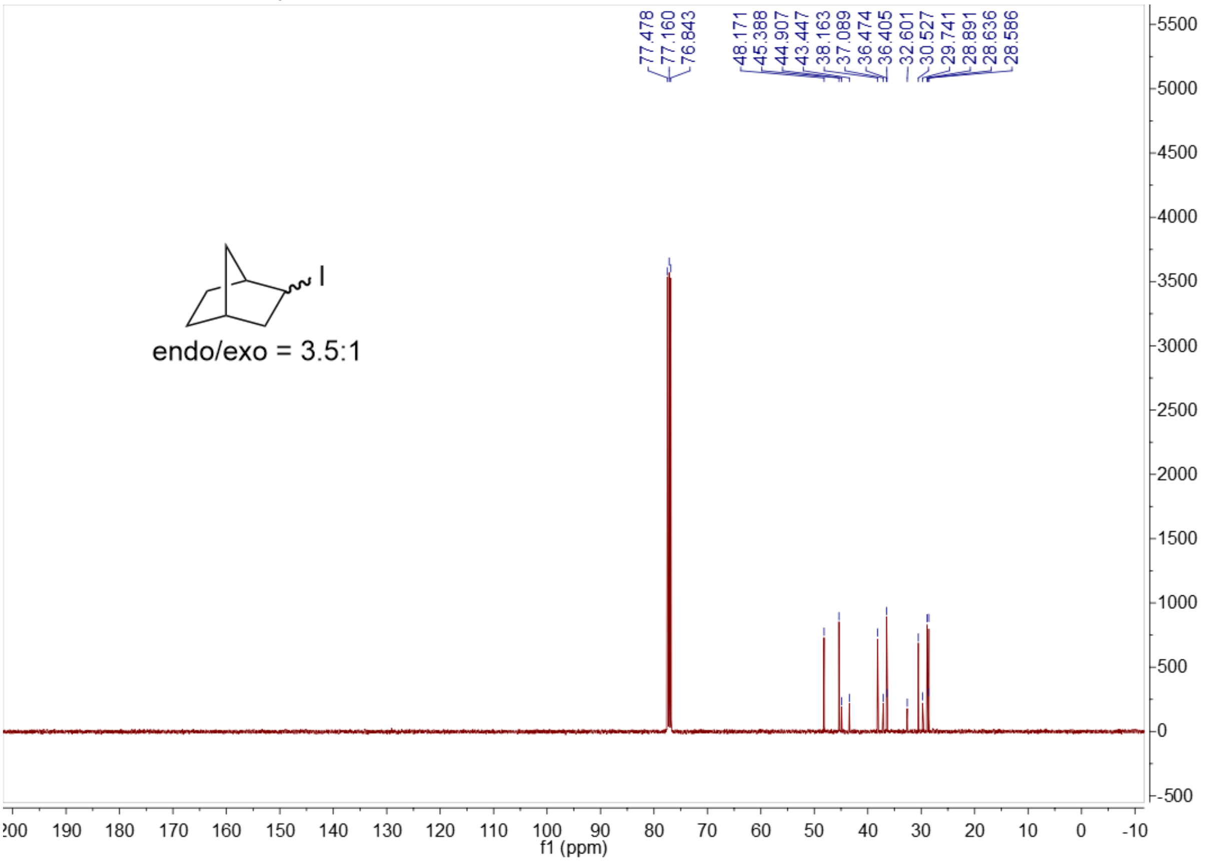

^{1}H NMR of **2f** in $CDCl_3$

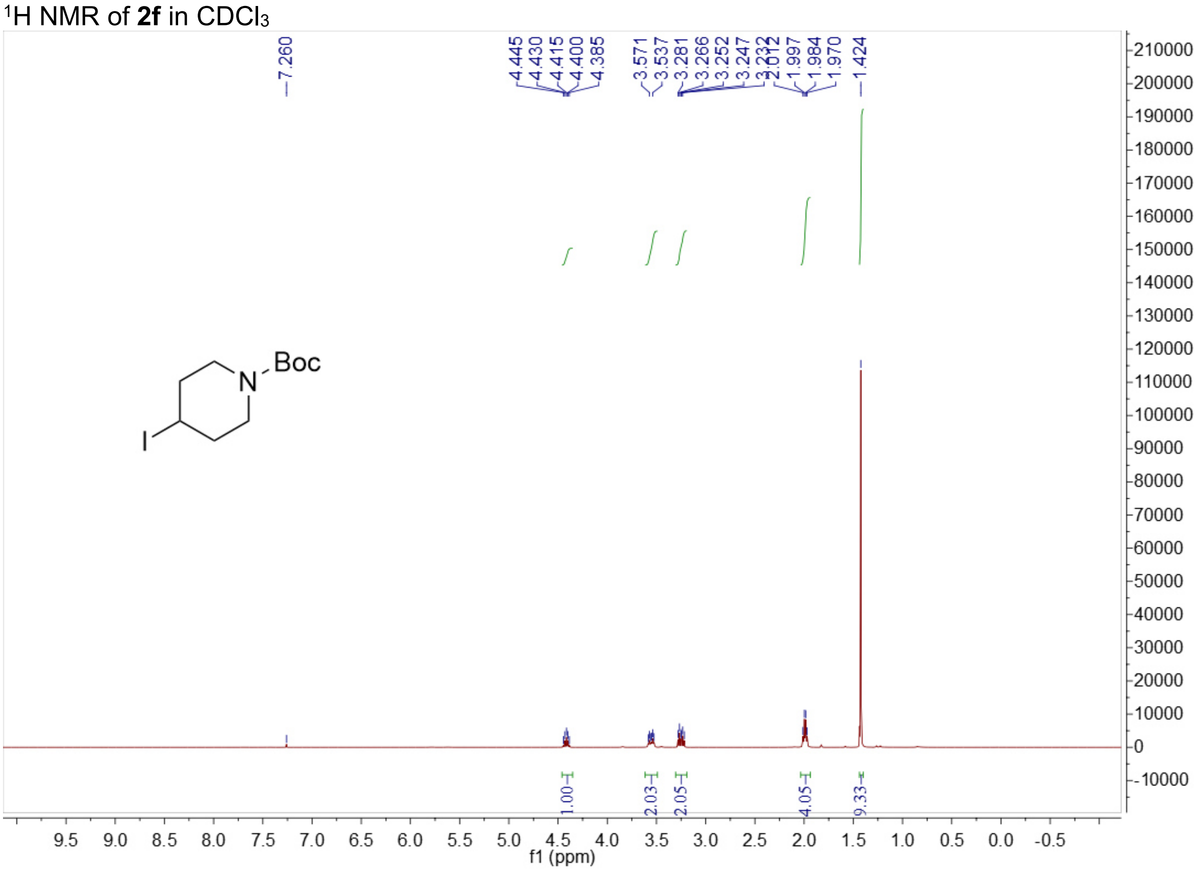

^{13}C NMR of **2f** in $CDCl_3$

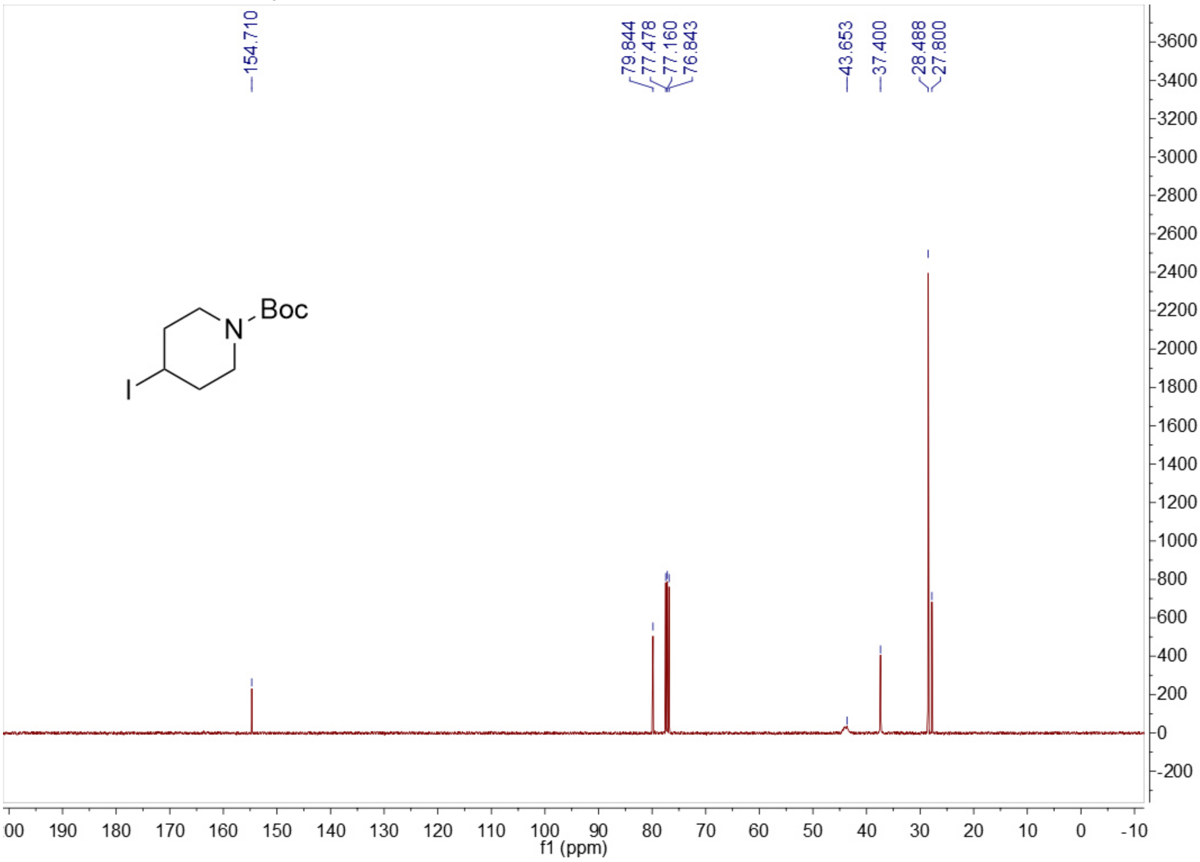

^{1}H NMR of **2h** in $CDCl_3$

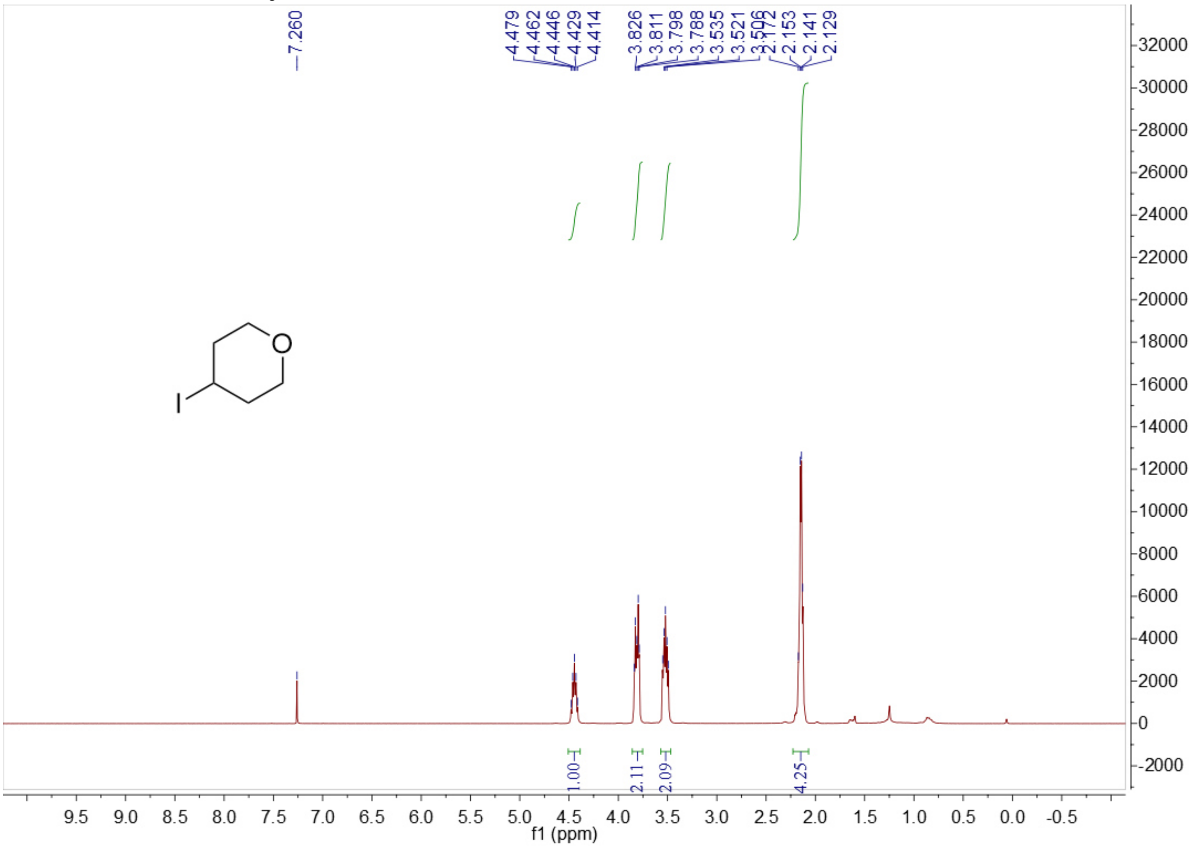

^{13}C NMR of **2h** in $CDCl_3$

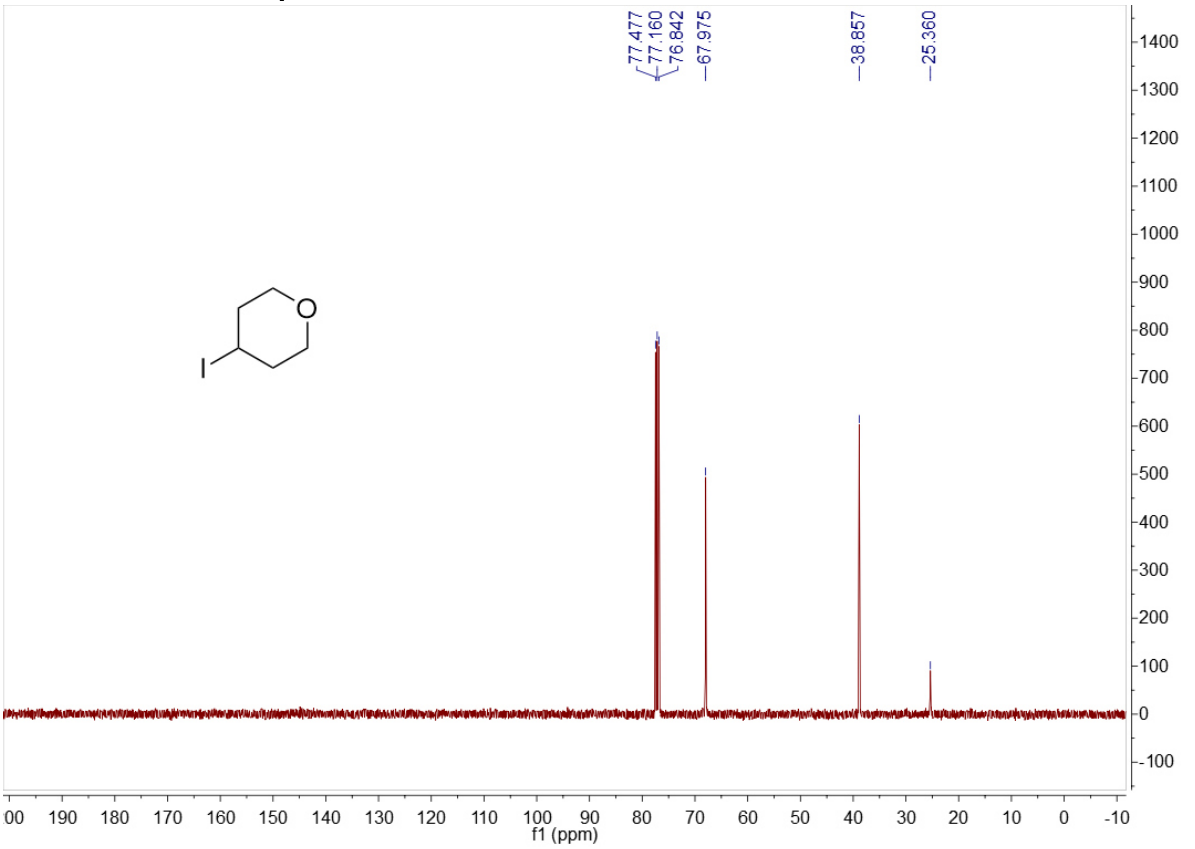

^{1}H NMR of compound **2t** in $CDCl_3$

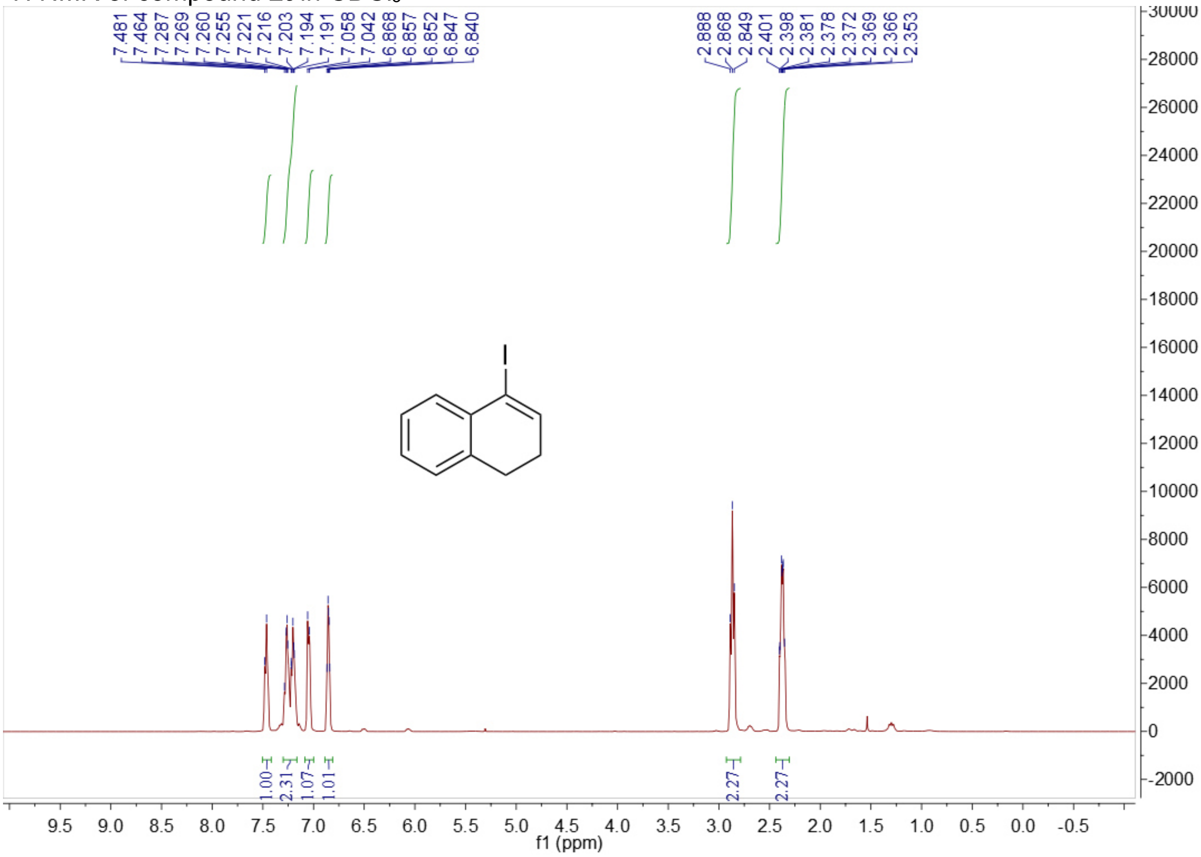

^{13}C NMR of compound **2t** in $CDCl_3$

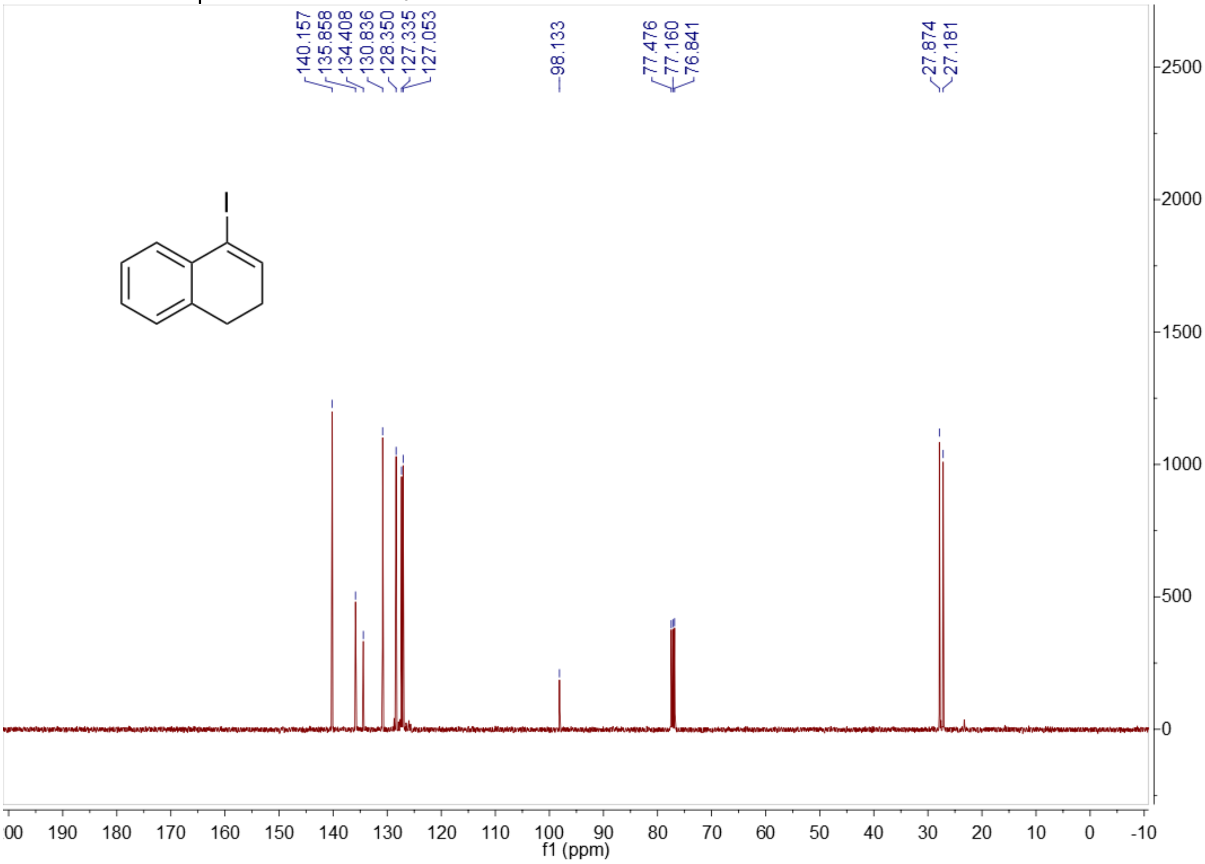

^{1}H NMR of compound **2z** in $CDCl_3$

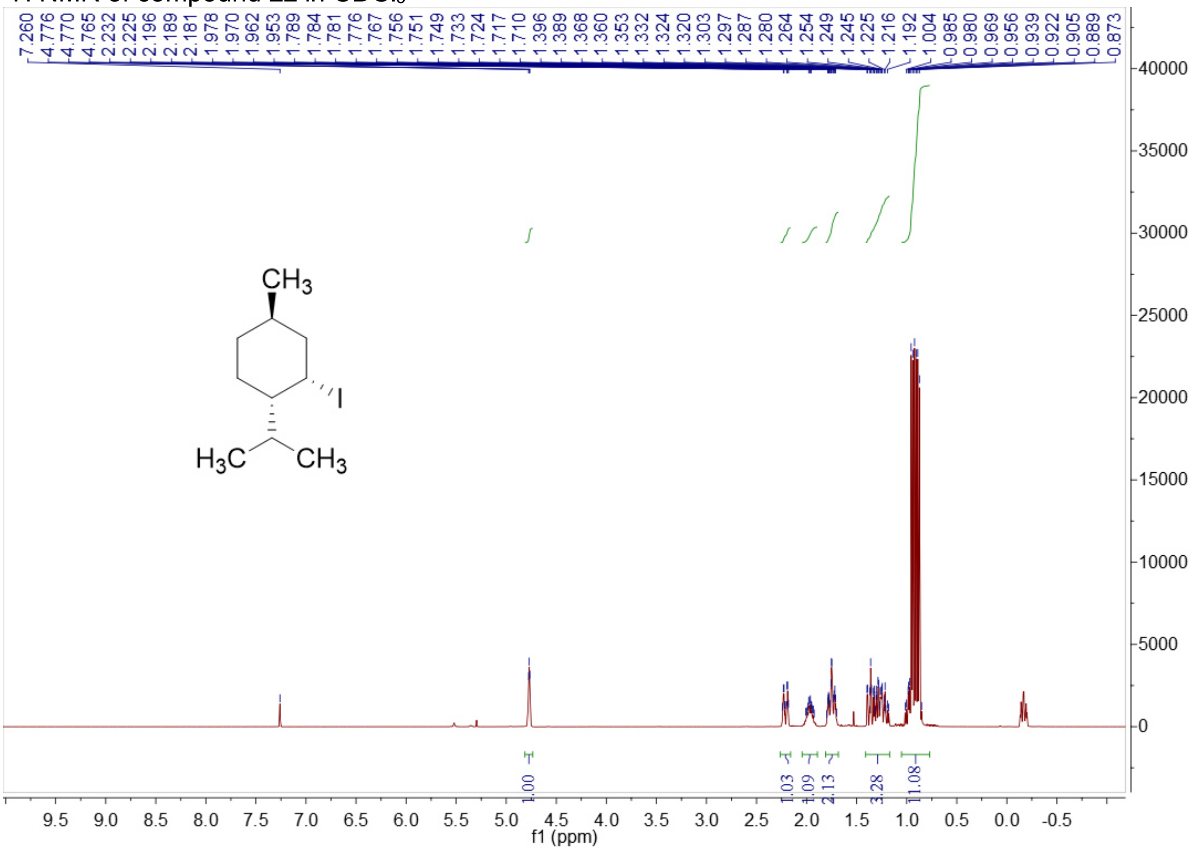

^{13}C NMR of compound **2z** in $CDCl_3$

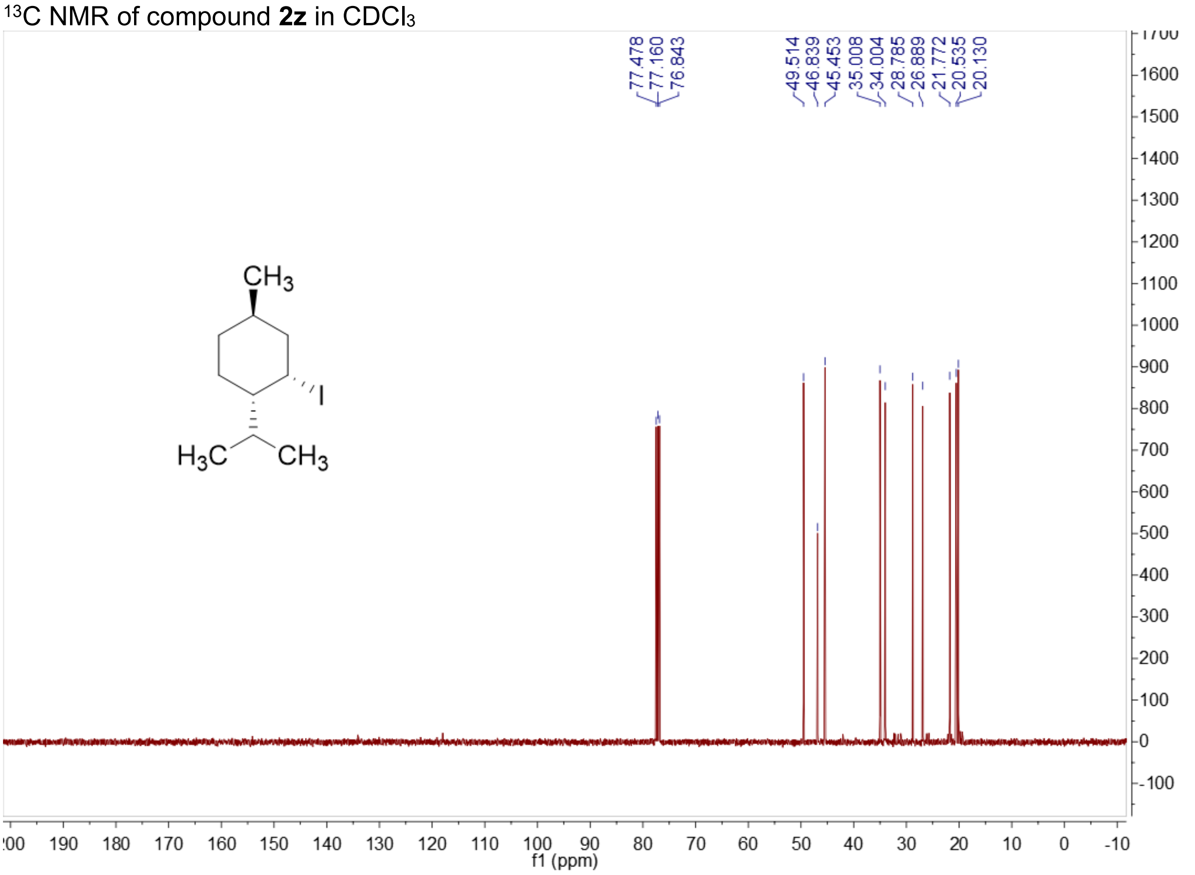

^{1}H NMR of compound **2aa** in $CDCl_3$

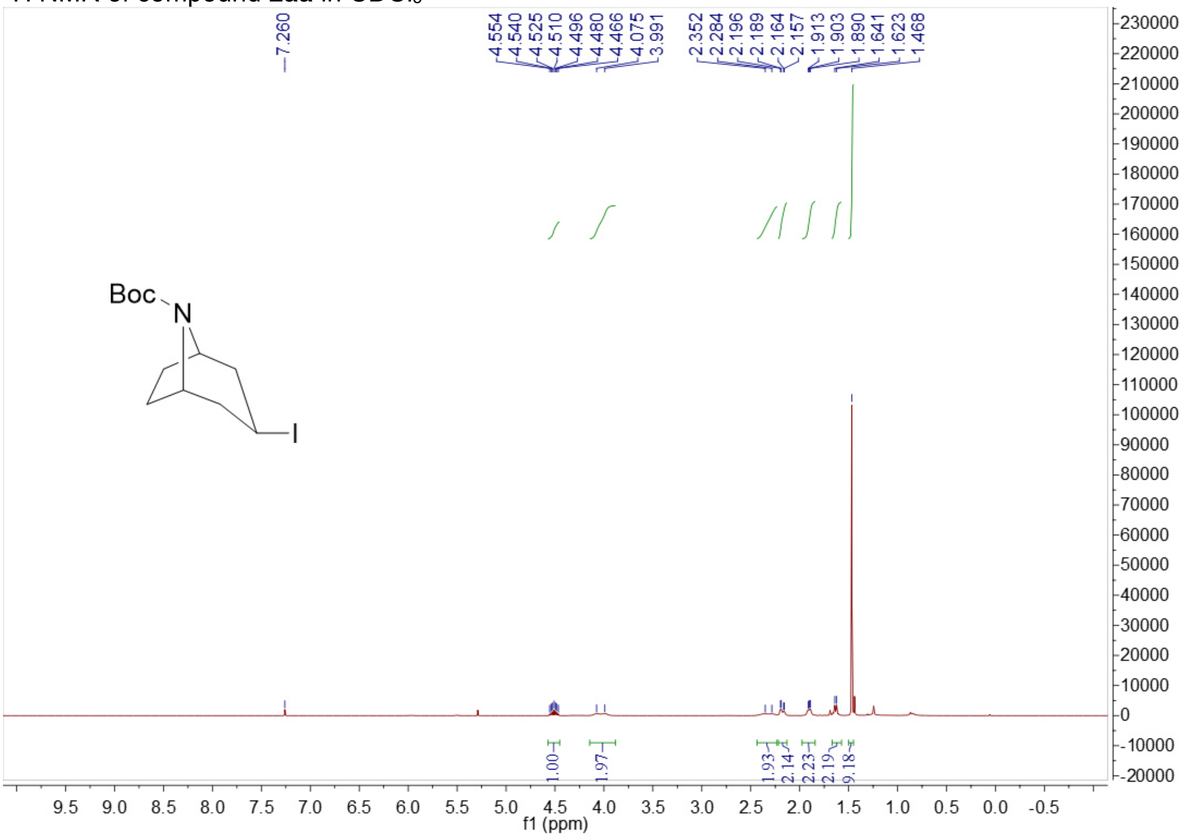

^{13}C NMR of compound **2aa** in $CDCl_3$

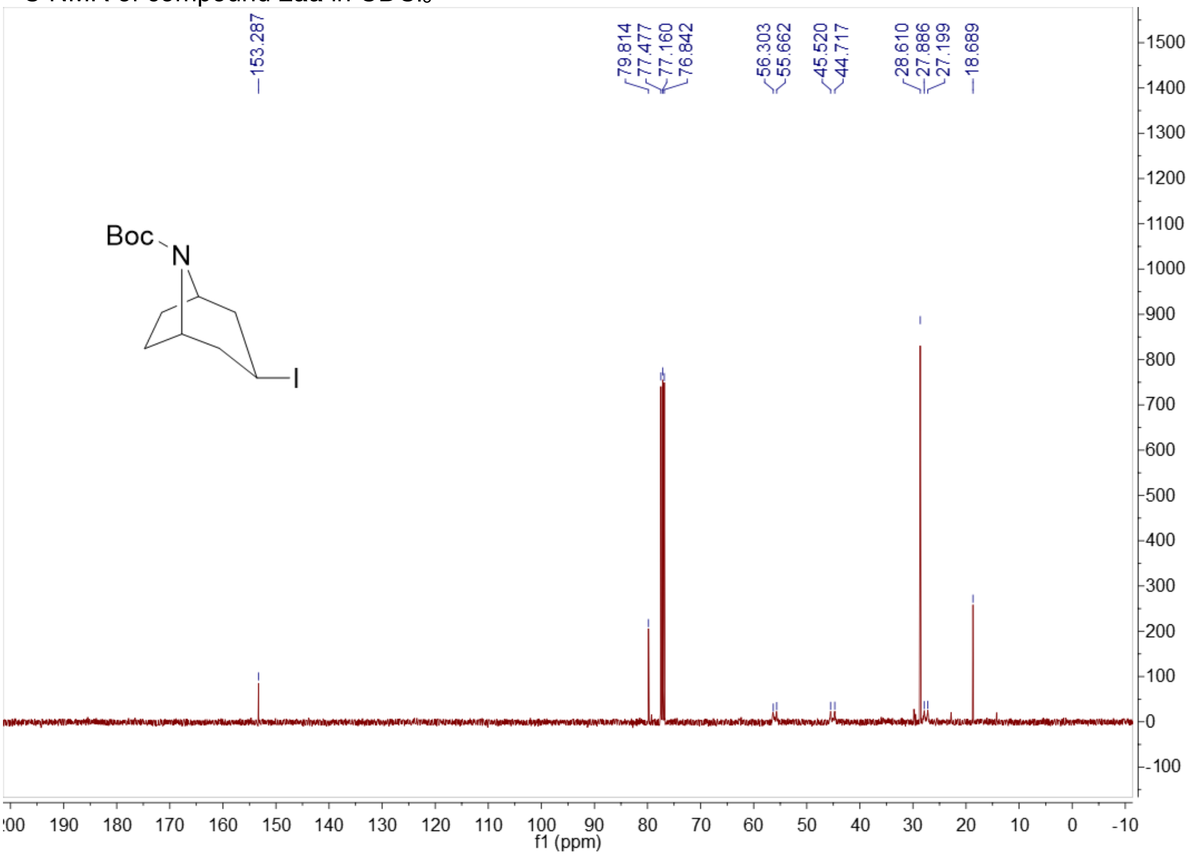

^{1}H NMR of compound **2ab** in $CDCl_3$

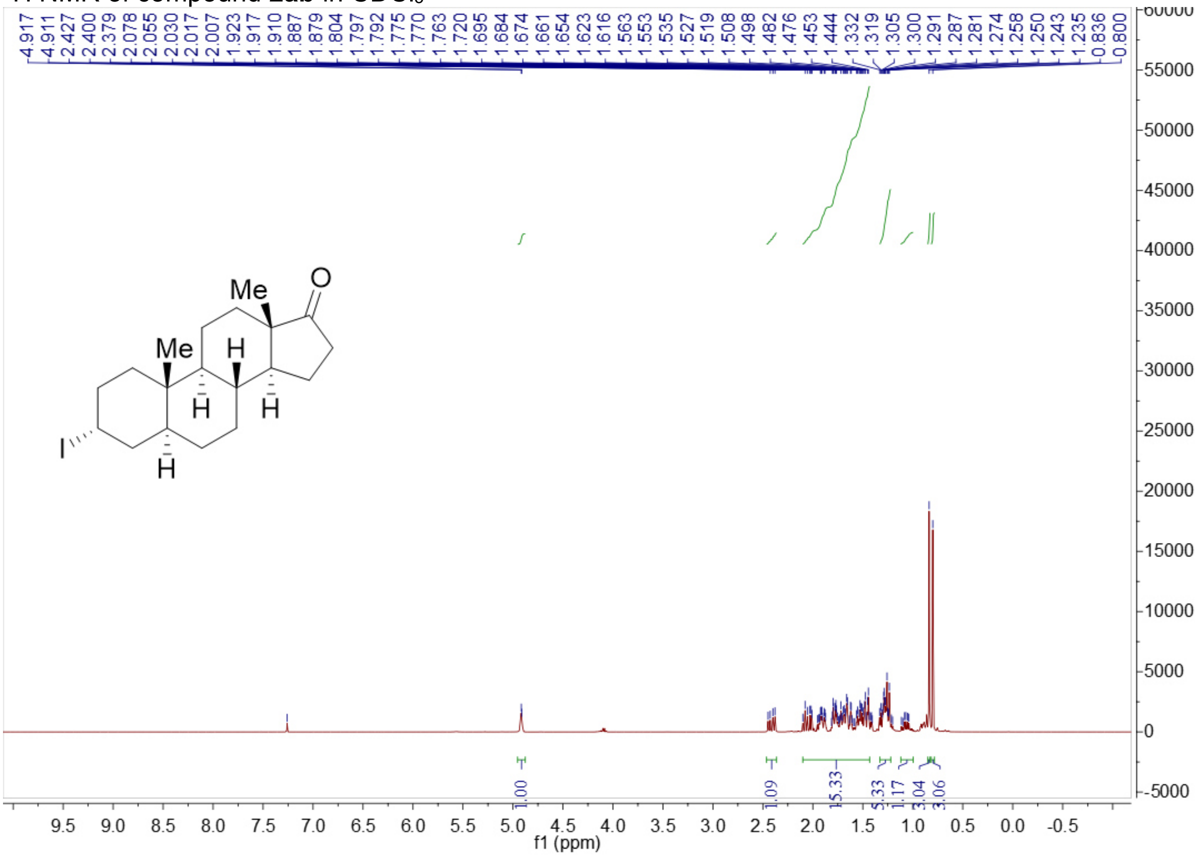

^{13}C NMR of compound **2ab** in $CDCl_3$

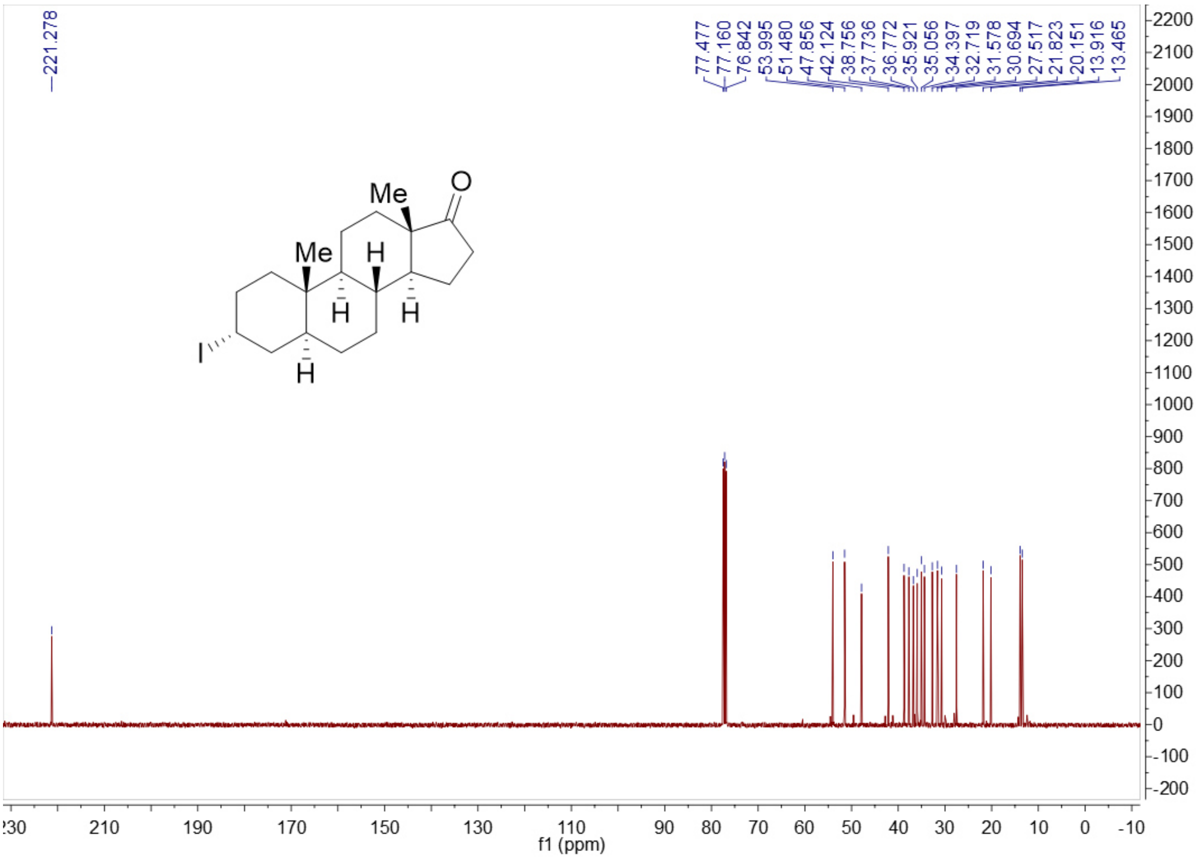

^{1}H NMR of compound **2ac** in $CDCl_3$

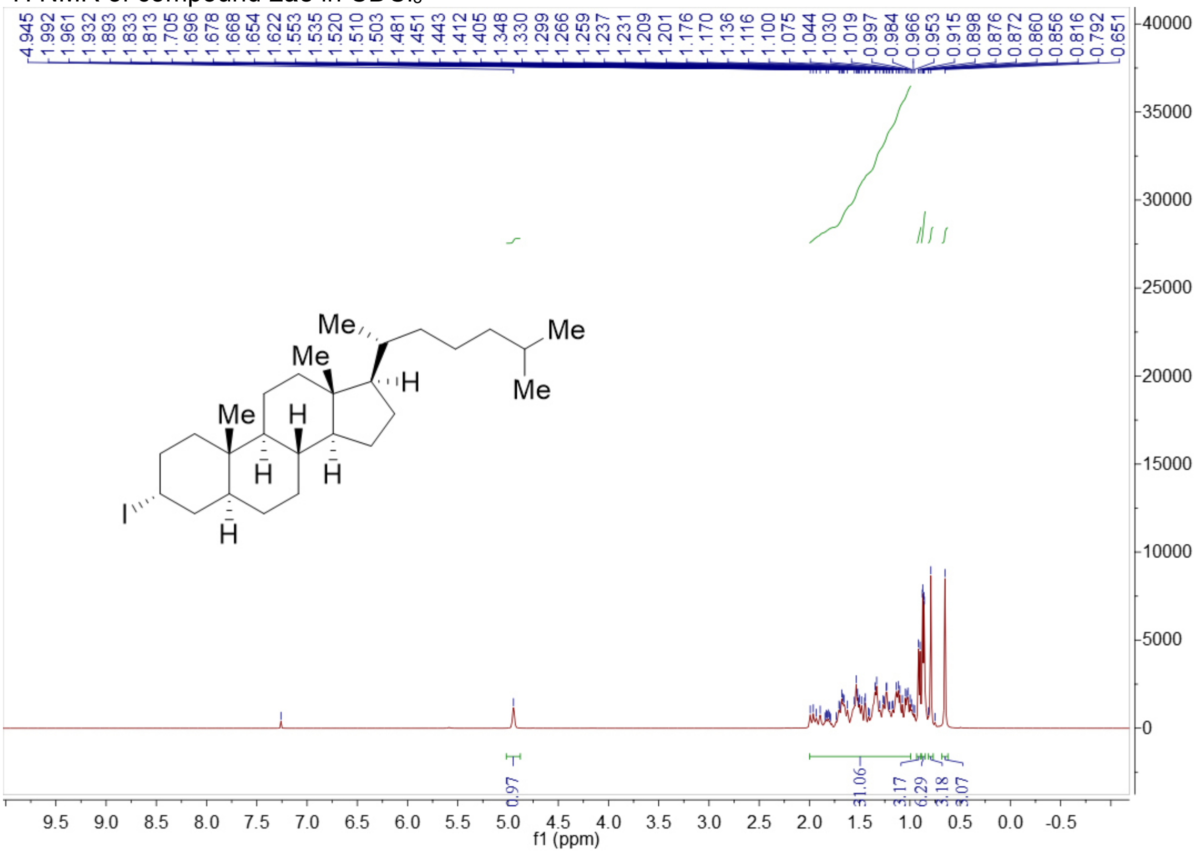

^{13}C NMR of compound **2ac** in $CDCl_3$

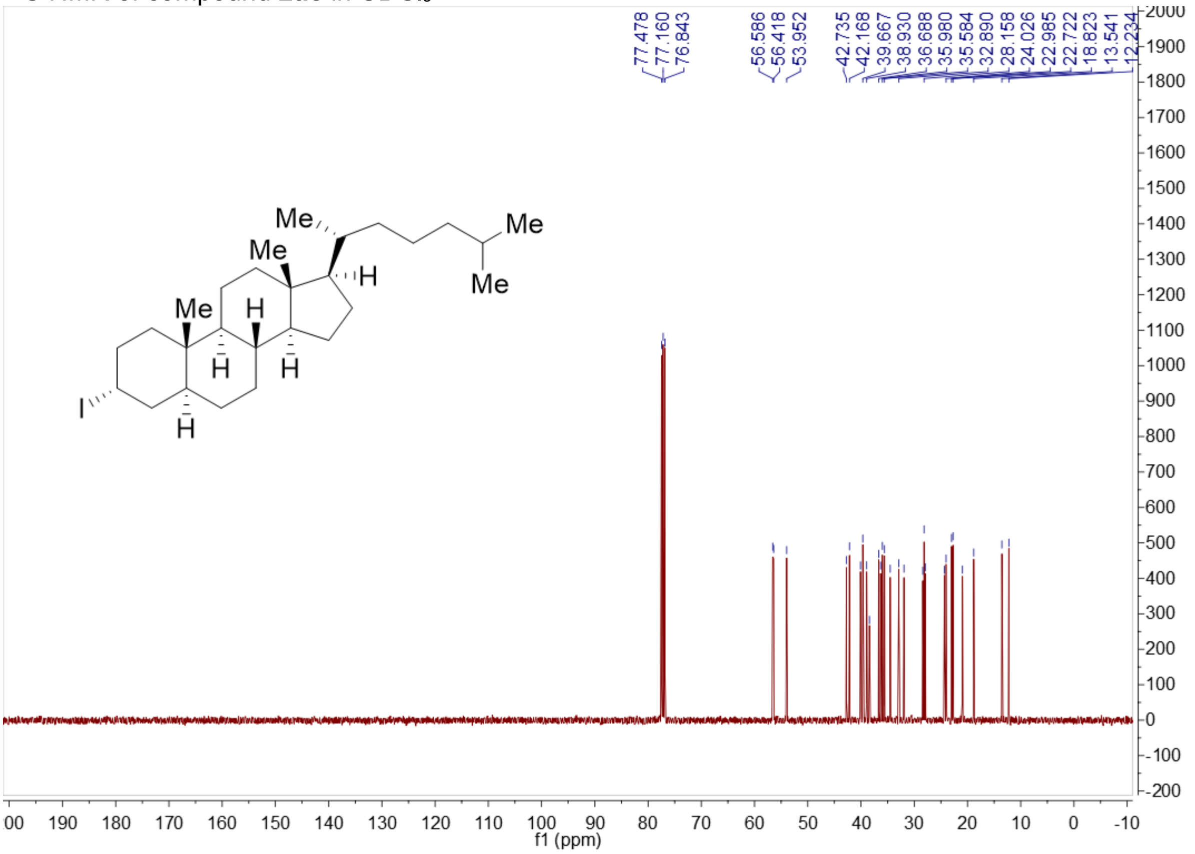

^{1}H NMR of compound **2ad** in $CDCl_3$

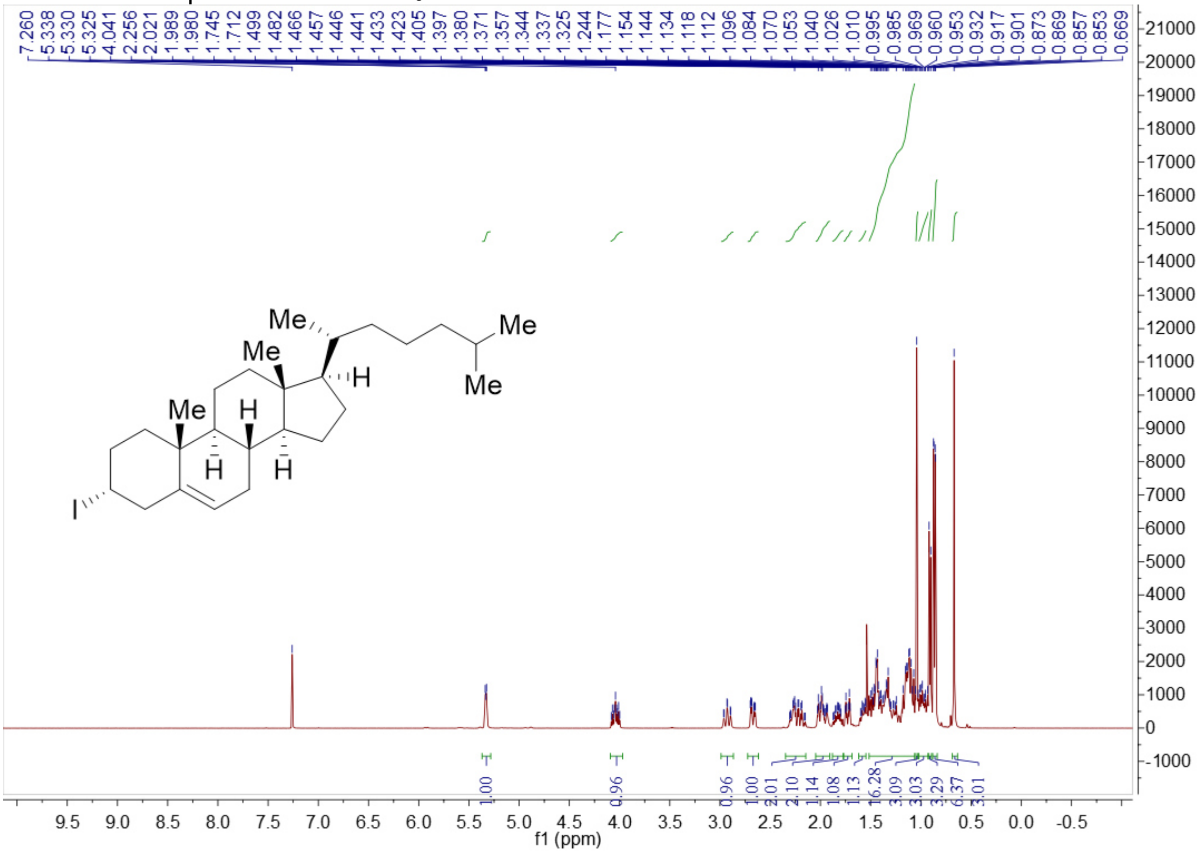

^{13}C NMR of compound **2ad** in $CDCl_3$

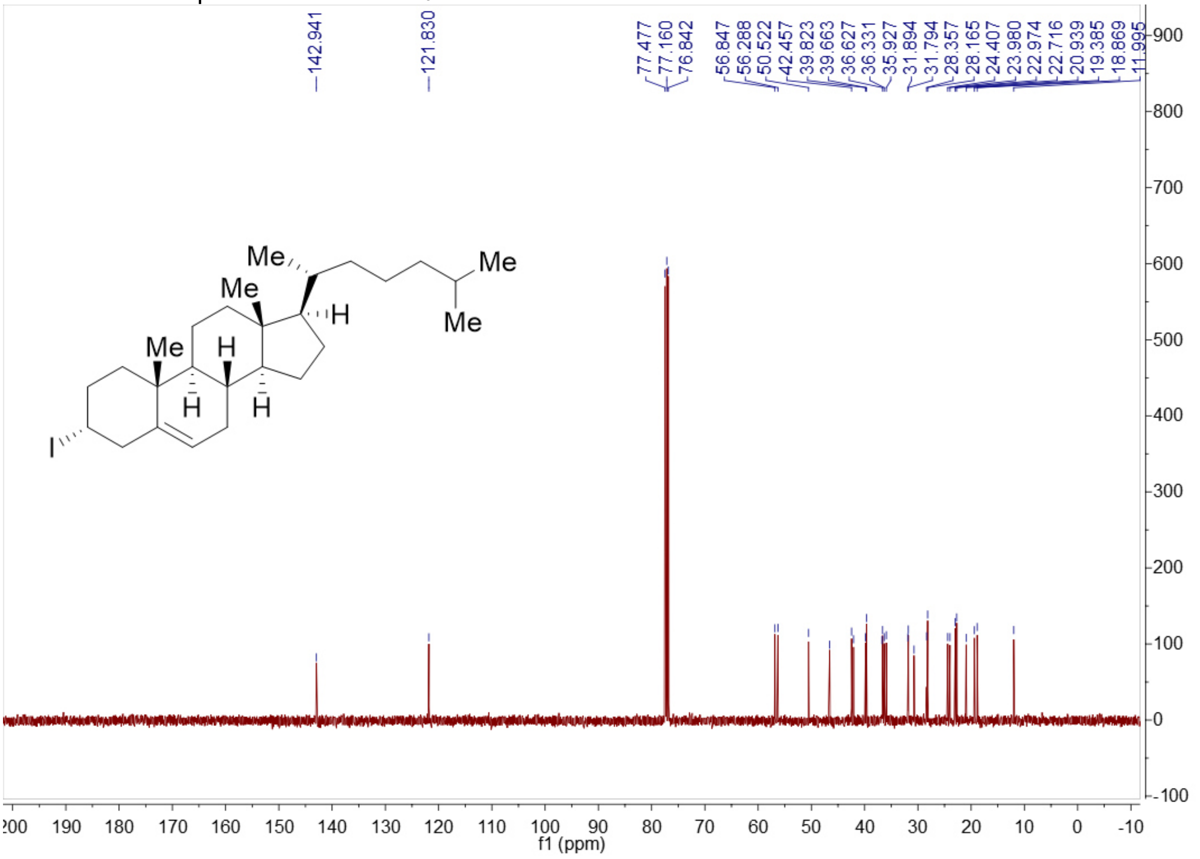

^{1}H NMR of compound **2ae** in $CDCl_3$

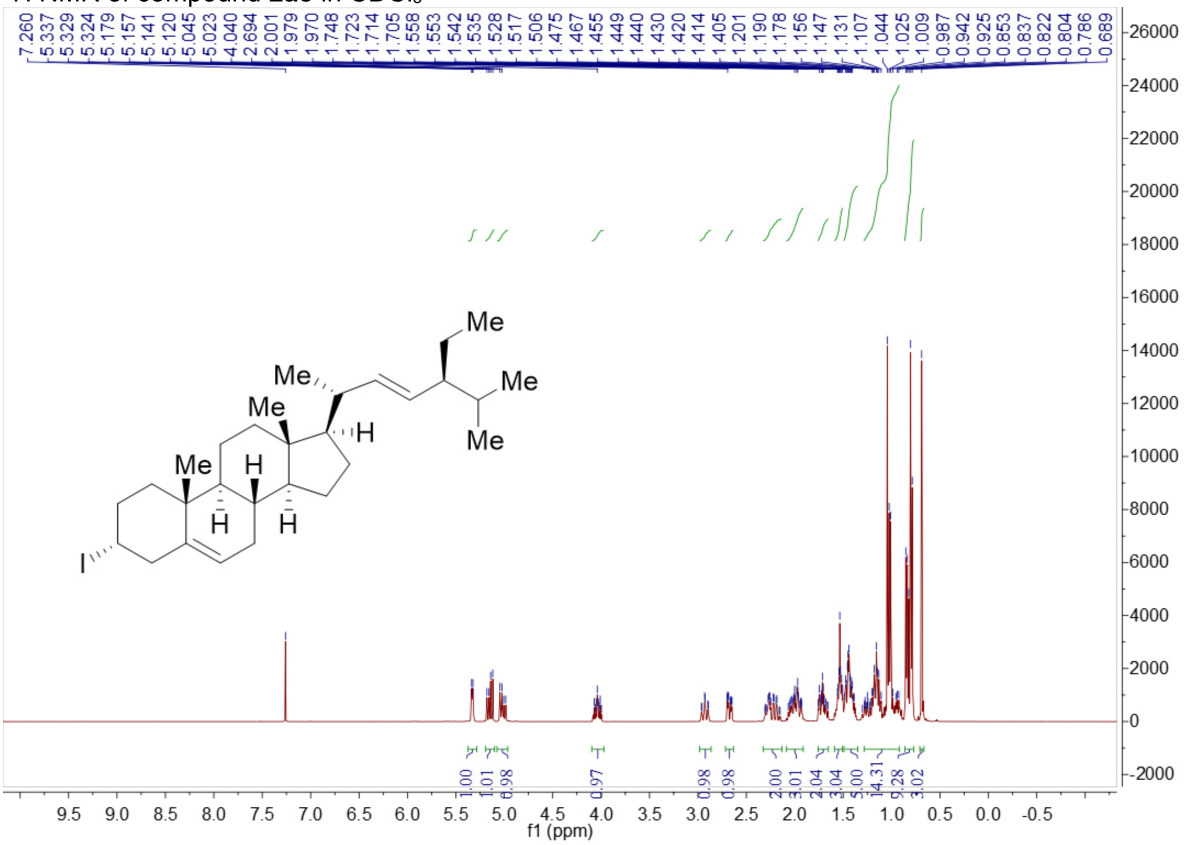

^{13}C NMR of compound **2ae** in $CDCl_3$

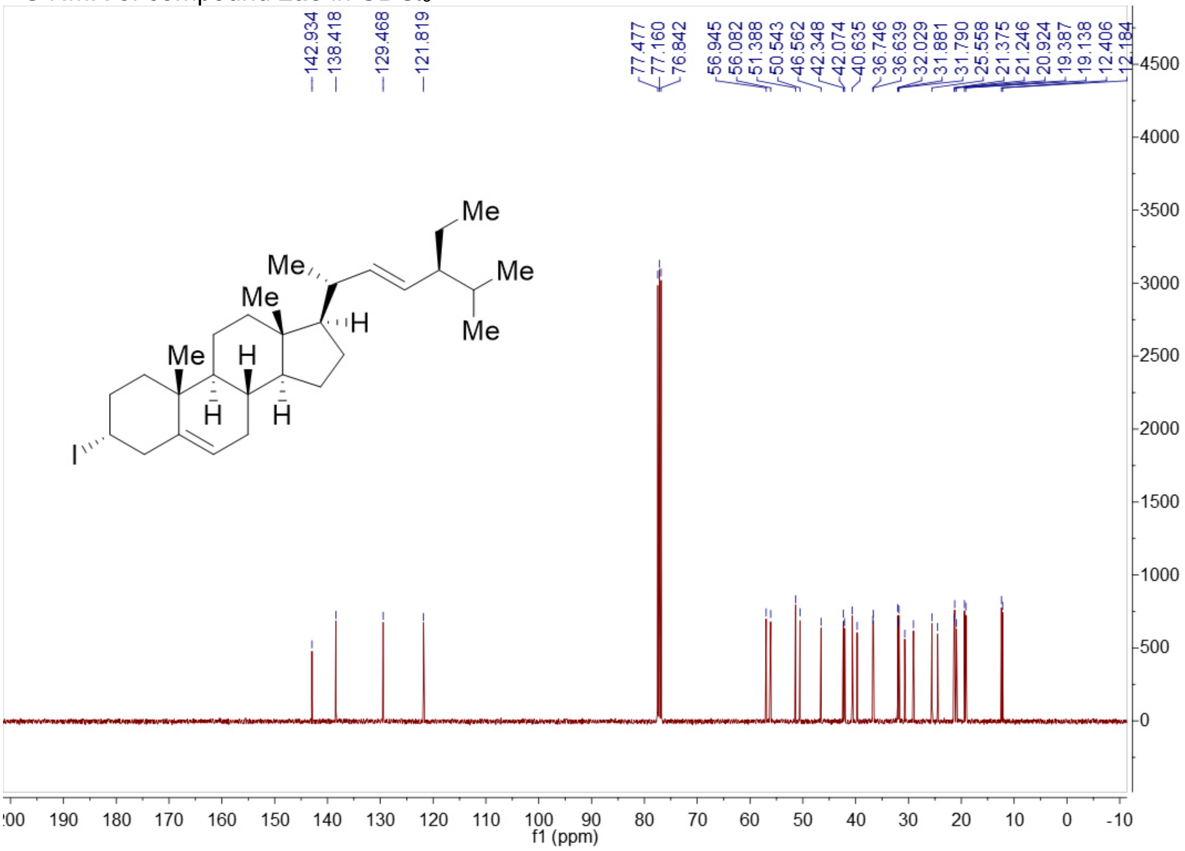

^{1}H NMR of compound **2af** in $CDCl_3$

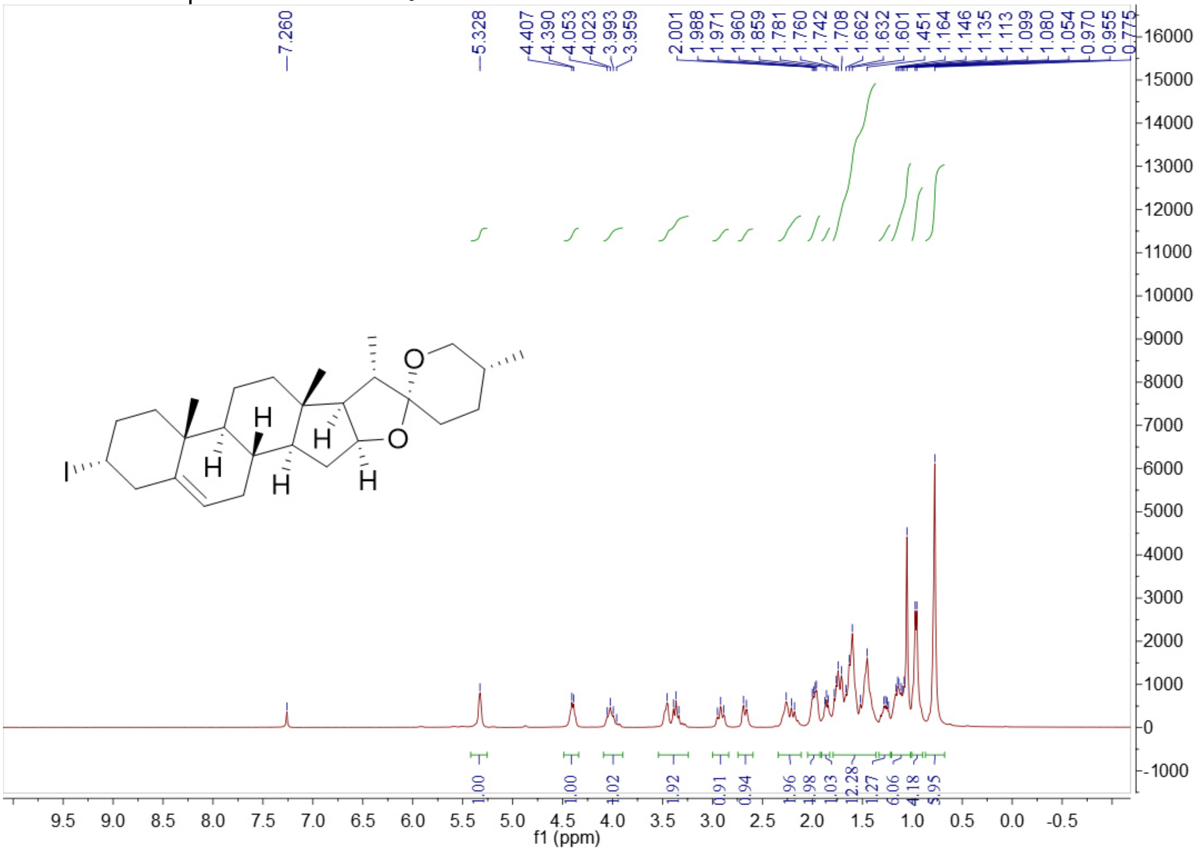

^{13}C NMR of compound **2af** in $CDCl_3$

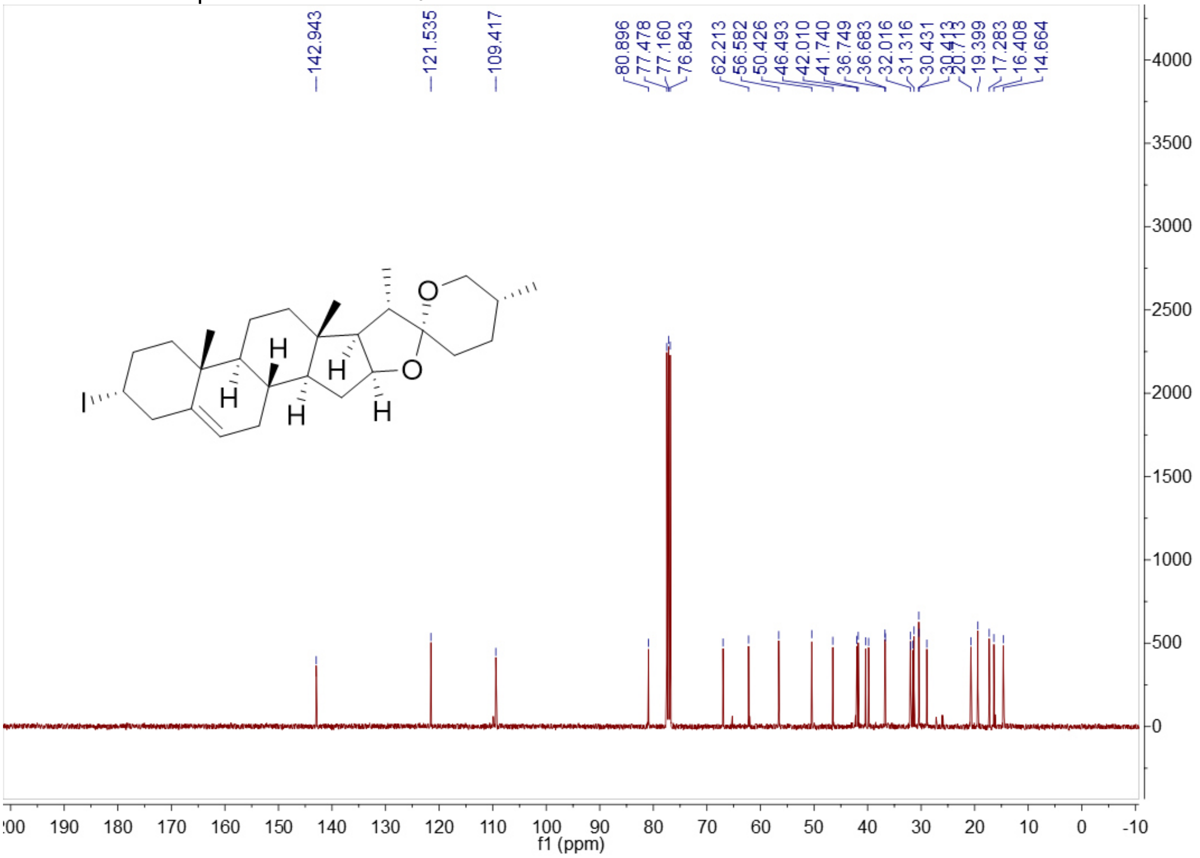

^{1}H NMR of compound **3aa** in $CDCl_3$

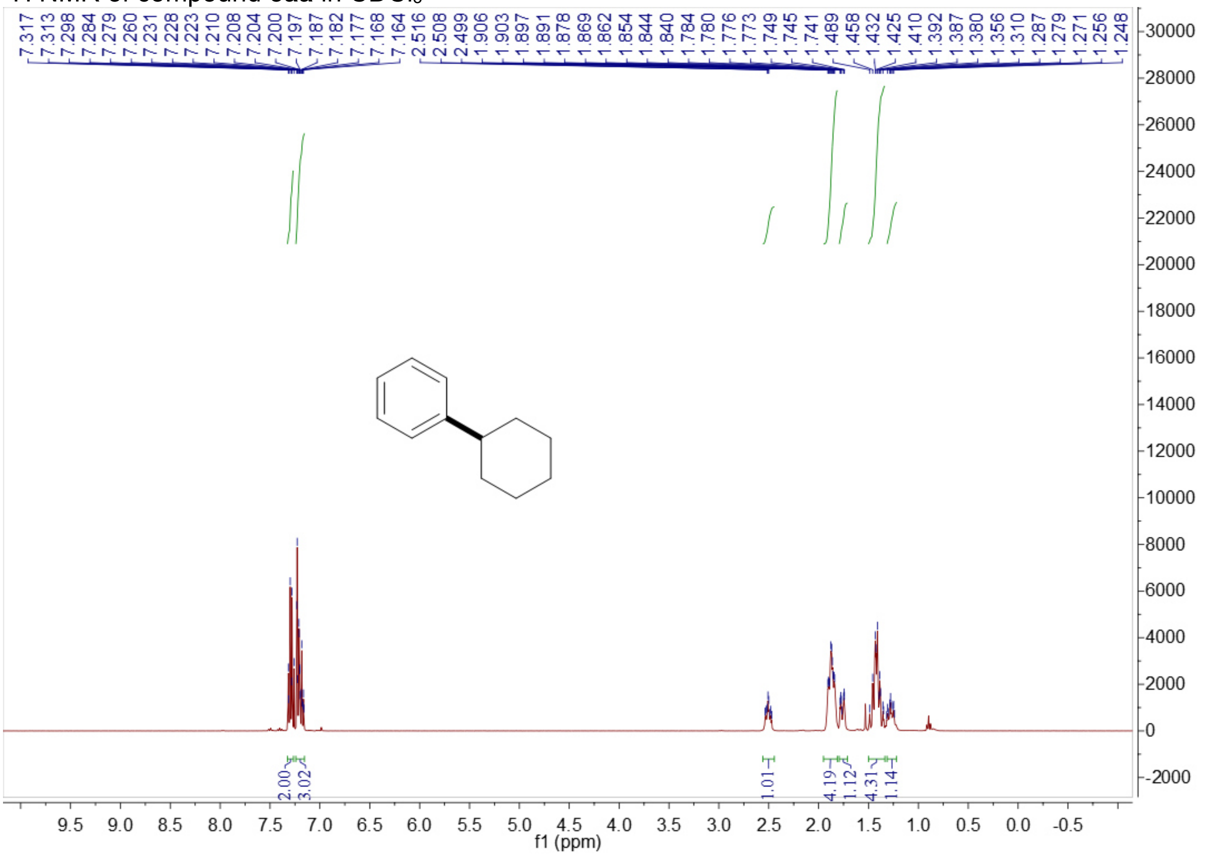

^{13}C NMR of compound **3aa** in $CDCl_3$

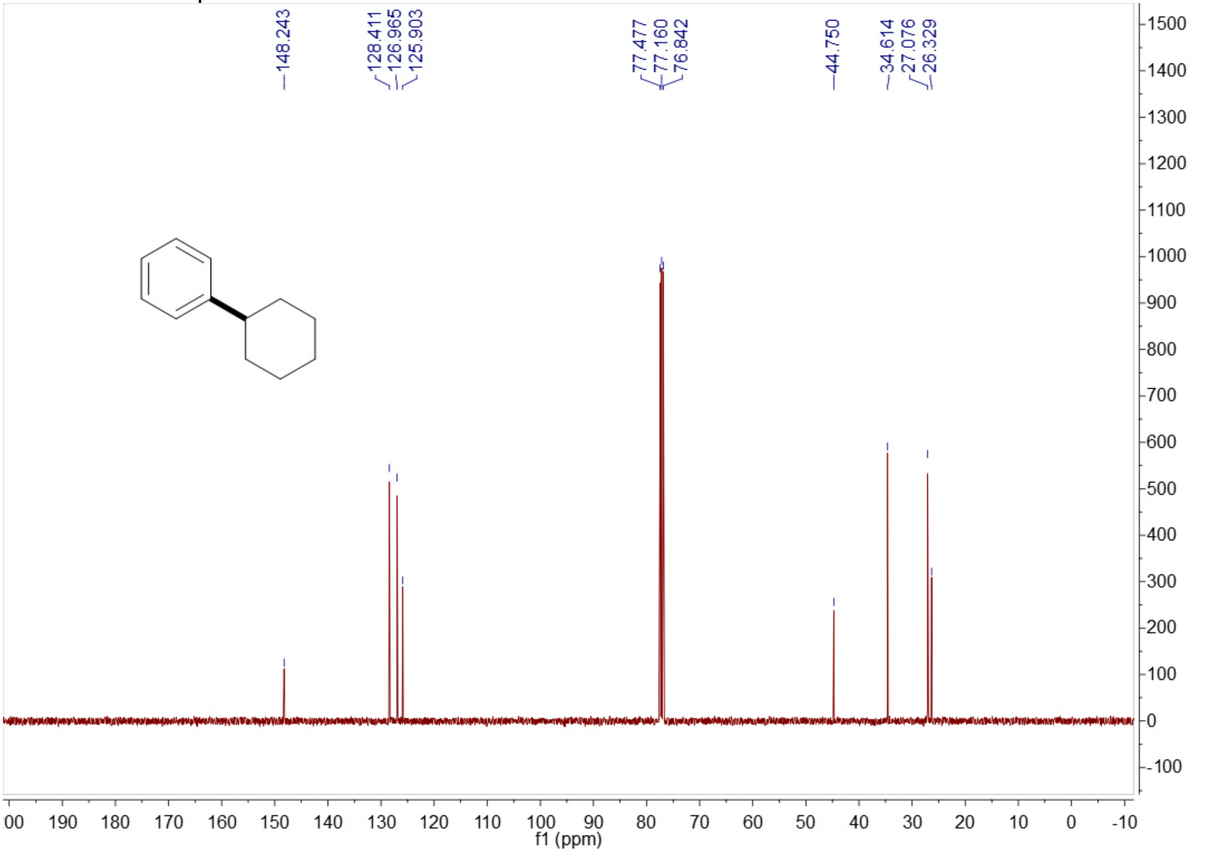

^{1}H NMR of compound **3ba** in $CDCl_3$

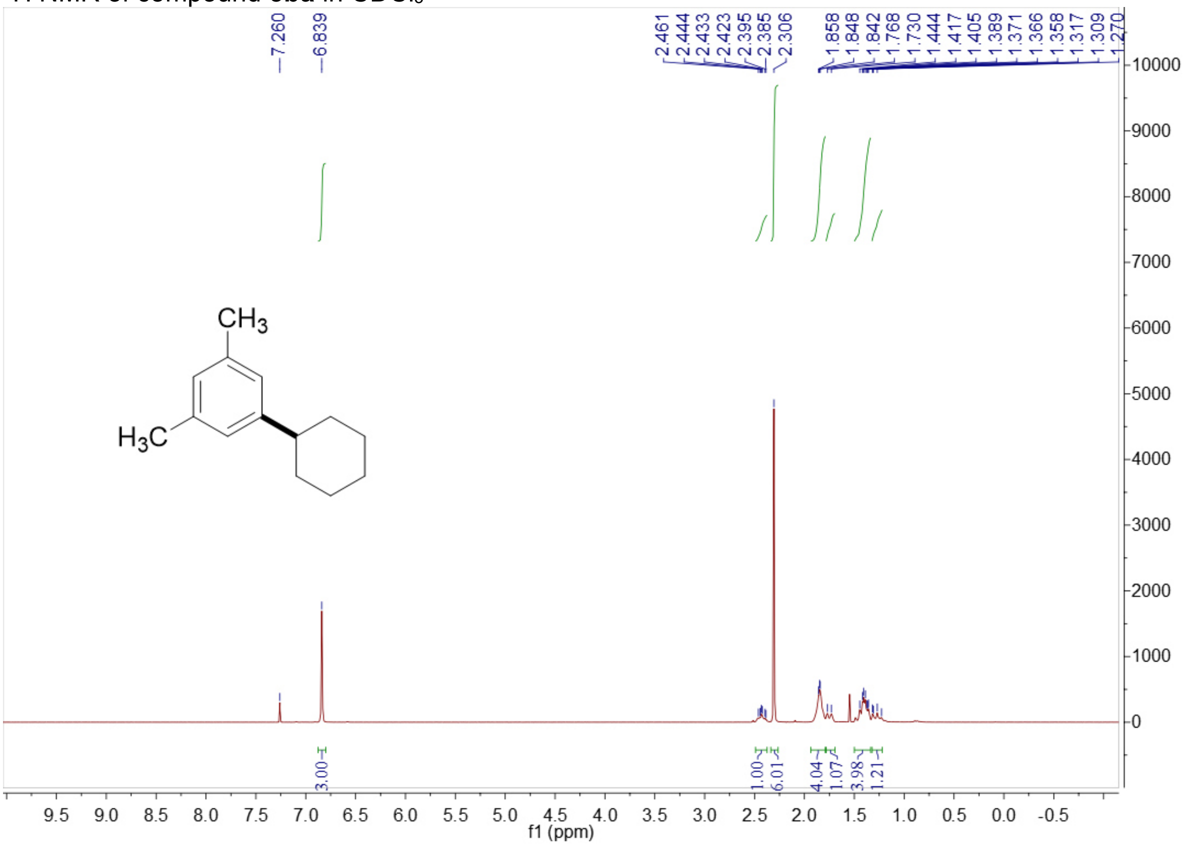

^{13}C NMR of compound **3ba** in $CDCl_3$

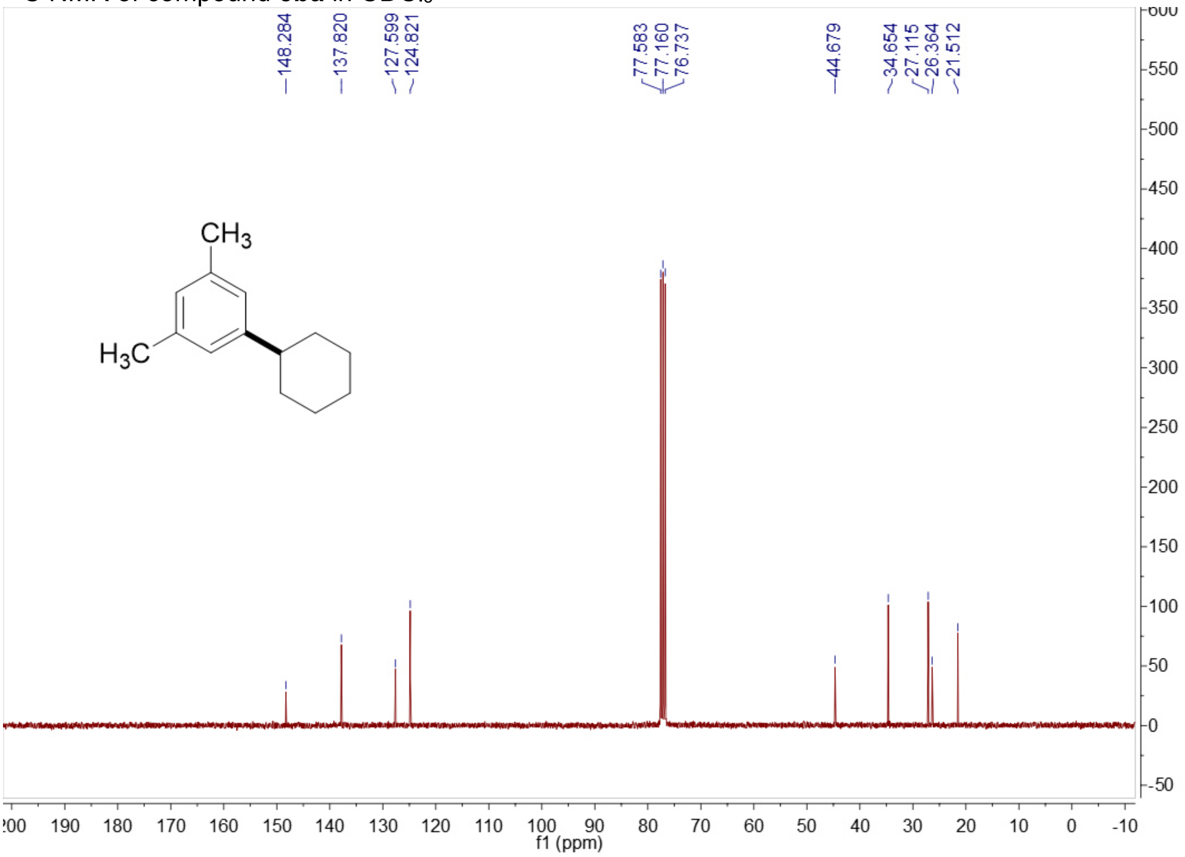

^{1}H NMR of compound **3ca** in $CDCl_3$

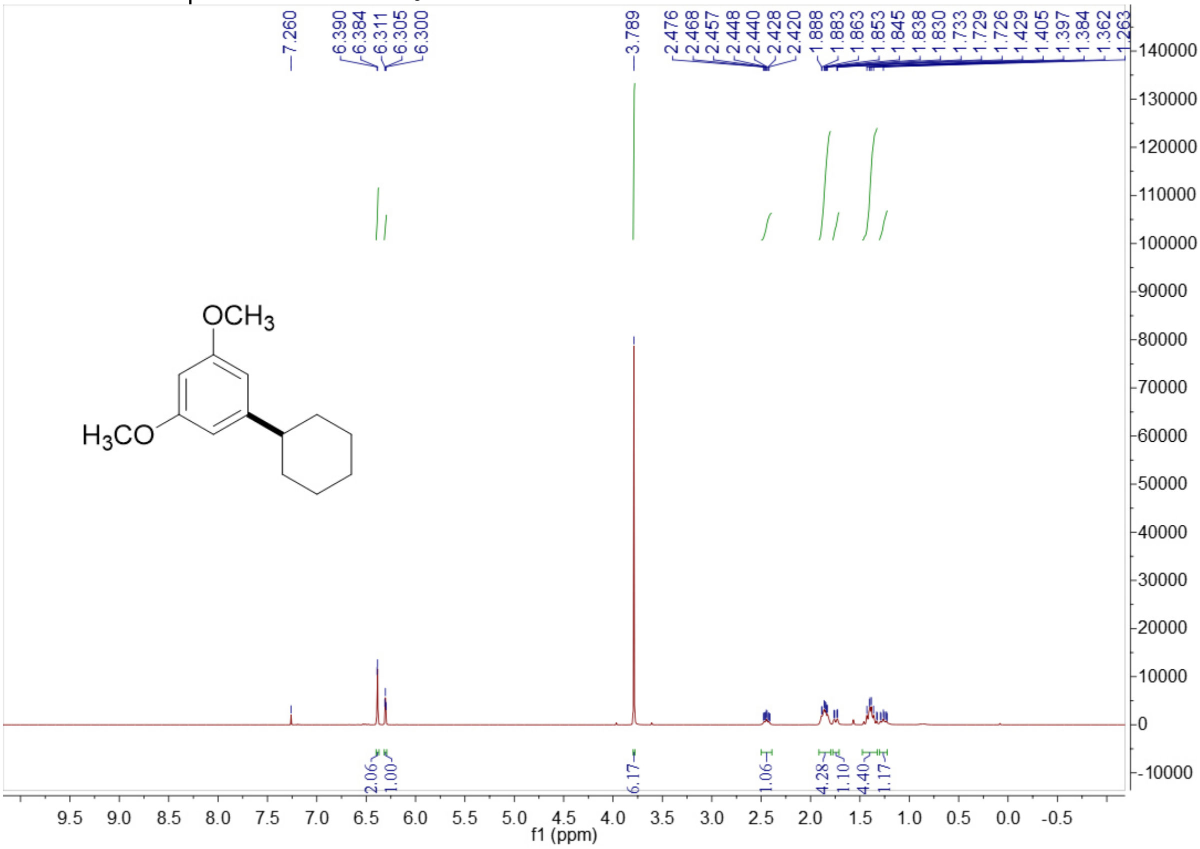

^{13}C NMR of compound **3ca** in $CDCl_3$

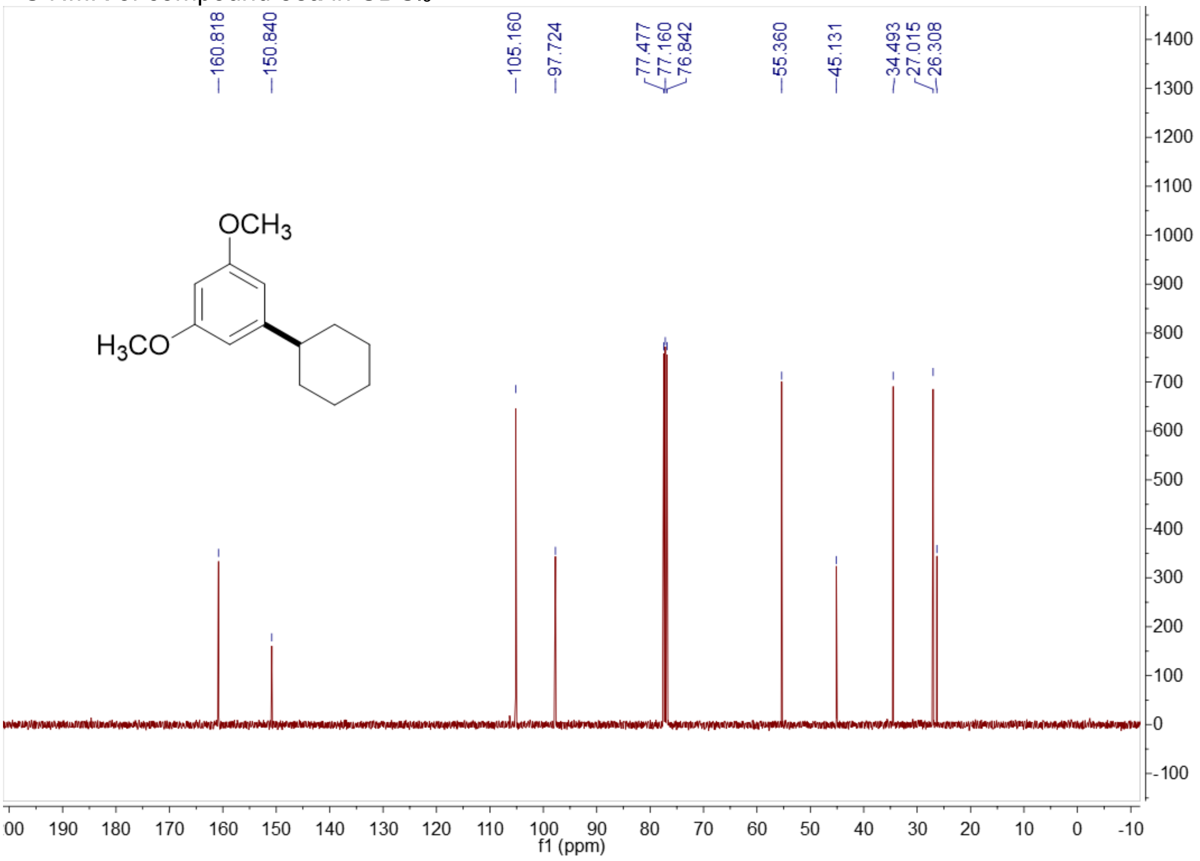

^1H NMR of compound **3da** in $CDCl_3$

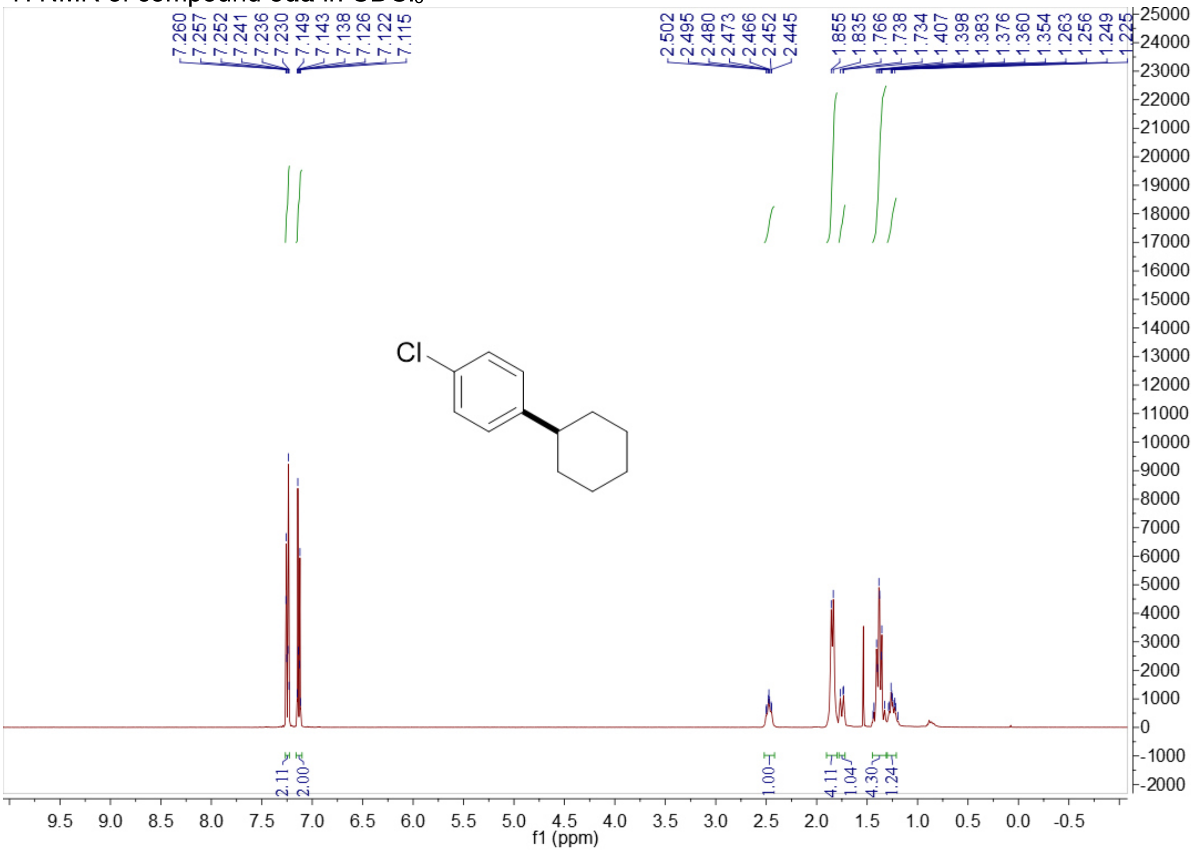

^{13}C NMR of compound **3da** in $CDCl_3$

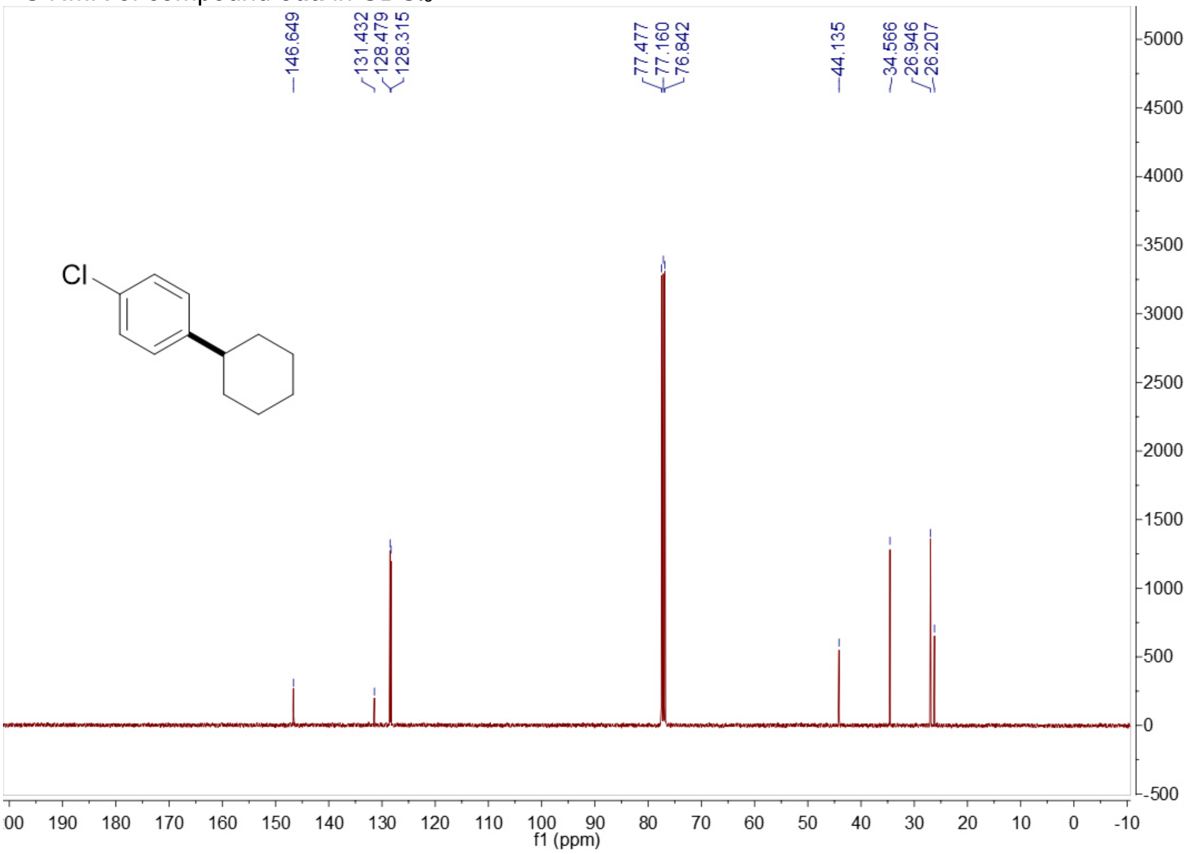

^{1}H NMR of compound **3ea** in $CDCl_3$

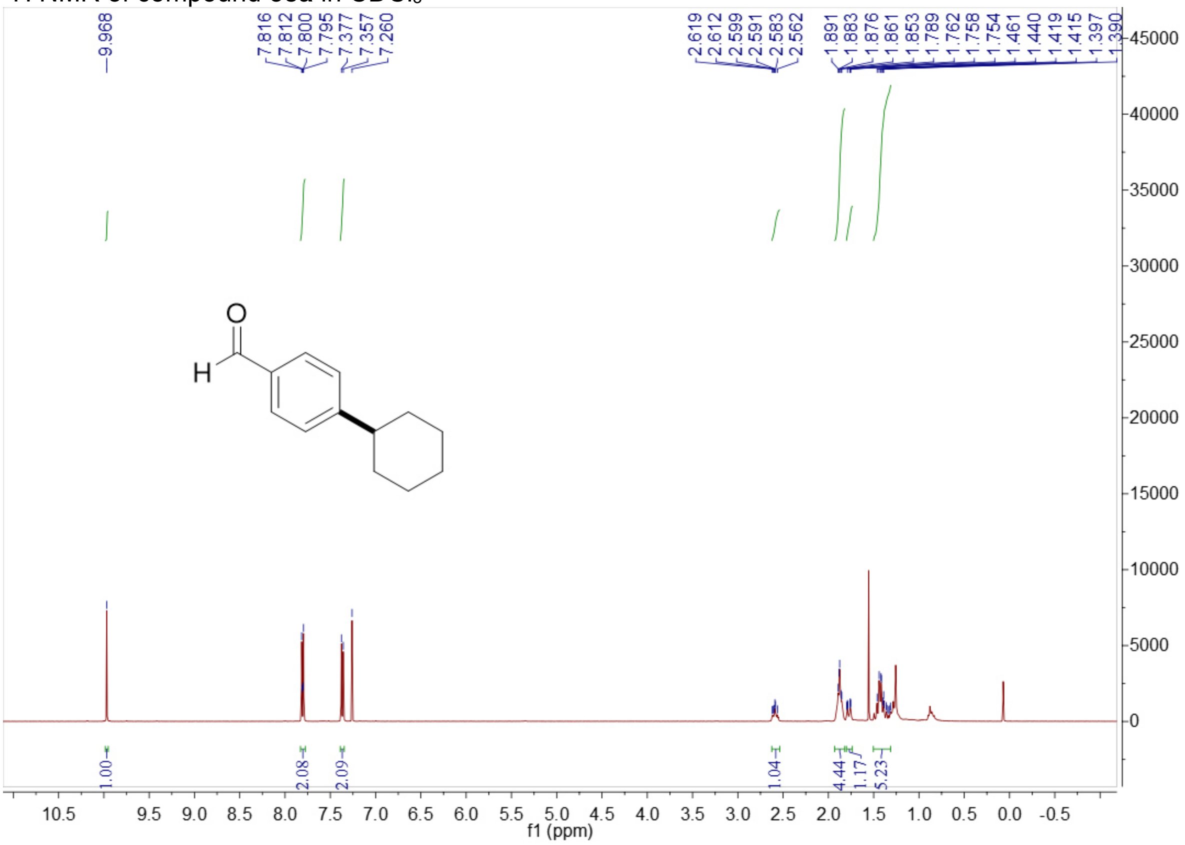

^{13}C NMR of compound **3ea** in $CDCl_3$

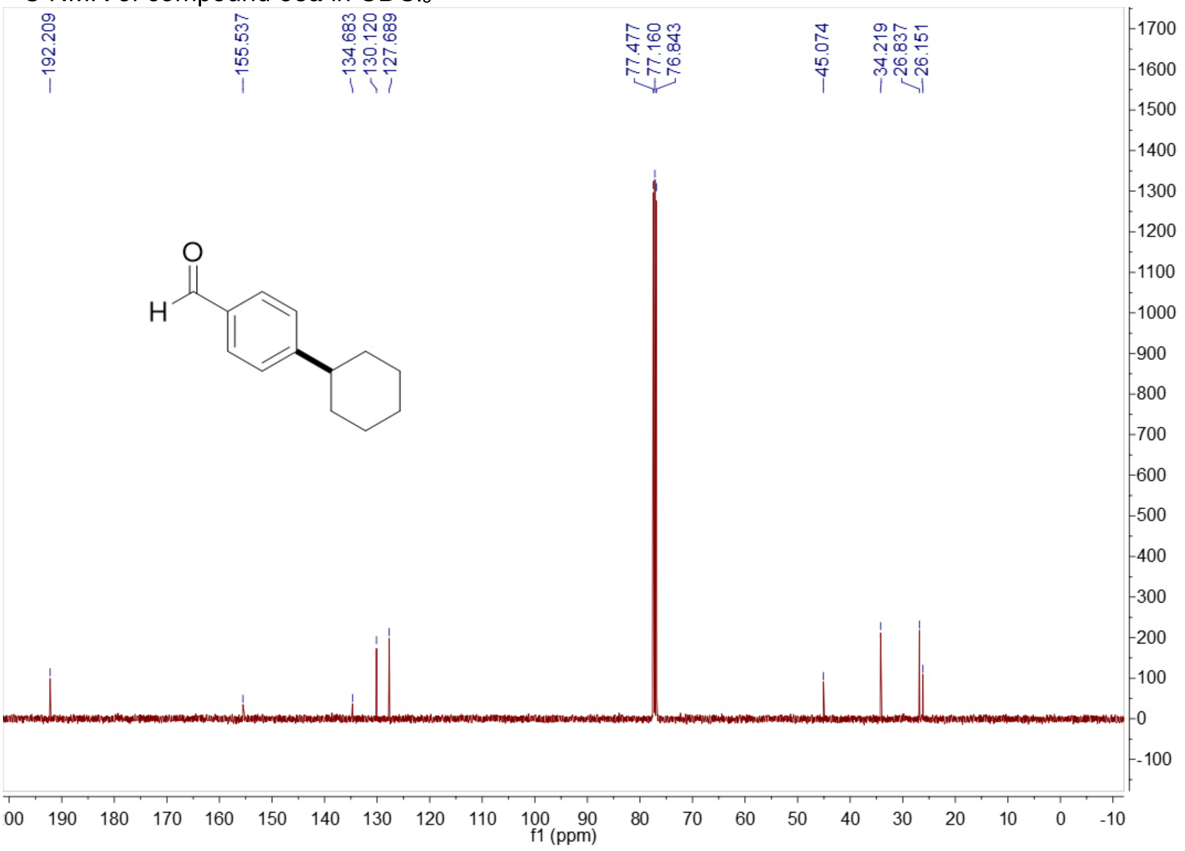

^{1}H NMR of compound **3fa** in $CDCl_3$

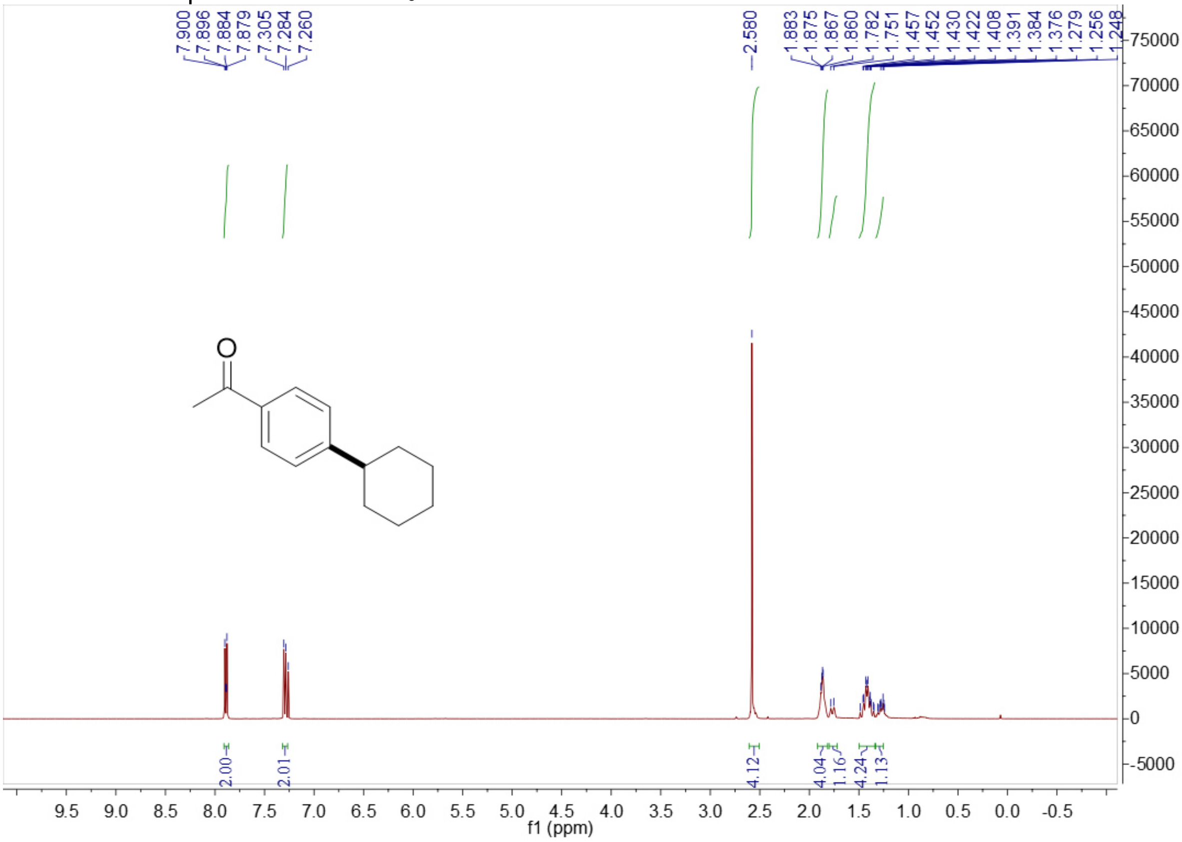

^{13}C NMR of compound **3fa** in $CDCl_3$

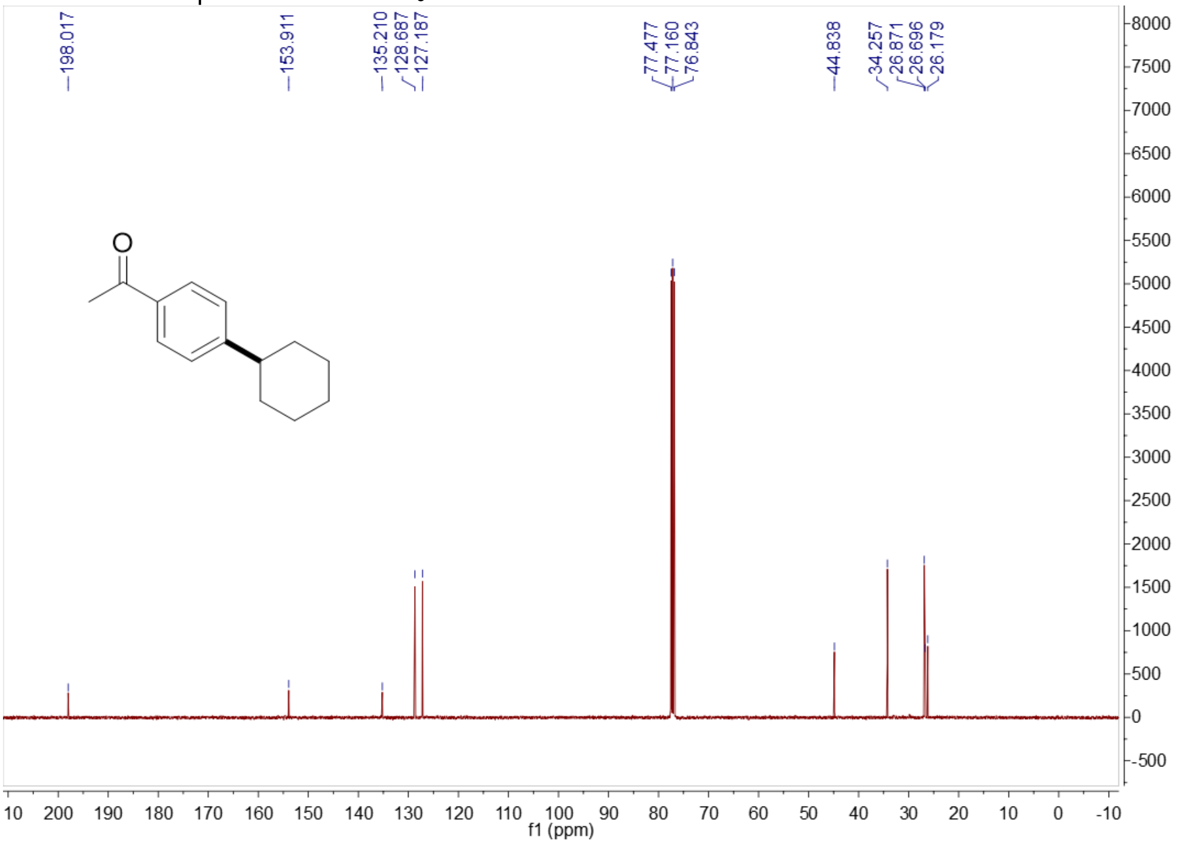

^{1}H NMR of compound **3ga** in $CDCl_3$

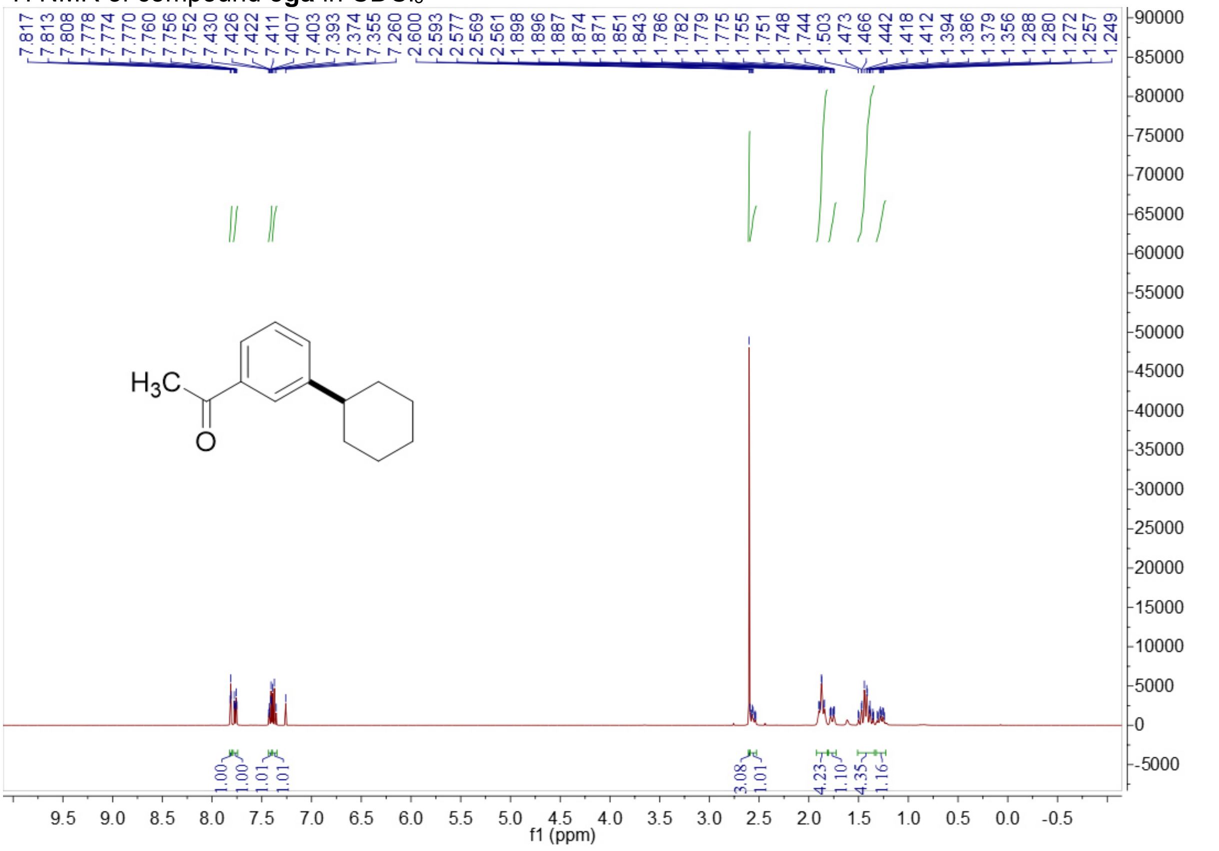

^{13}C NMR of compound **3ga** in $CDCl_3$

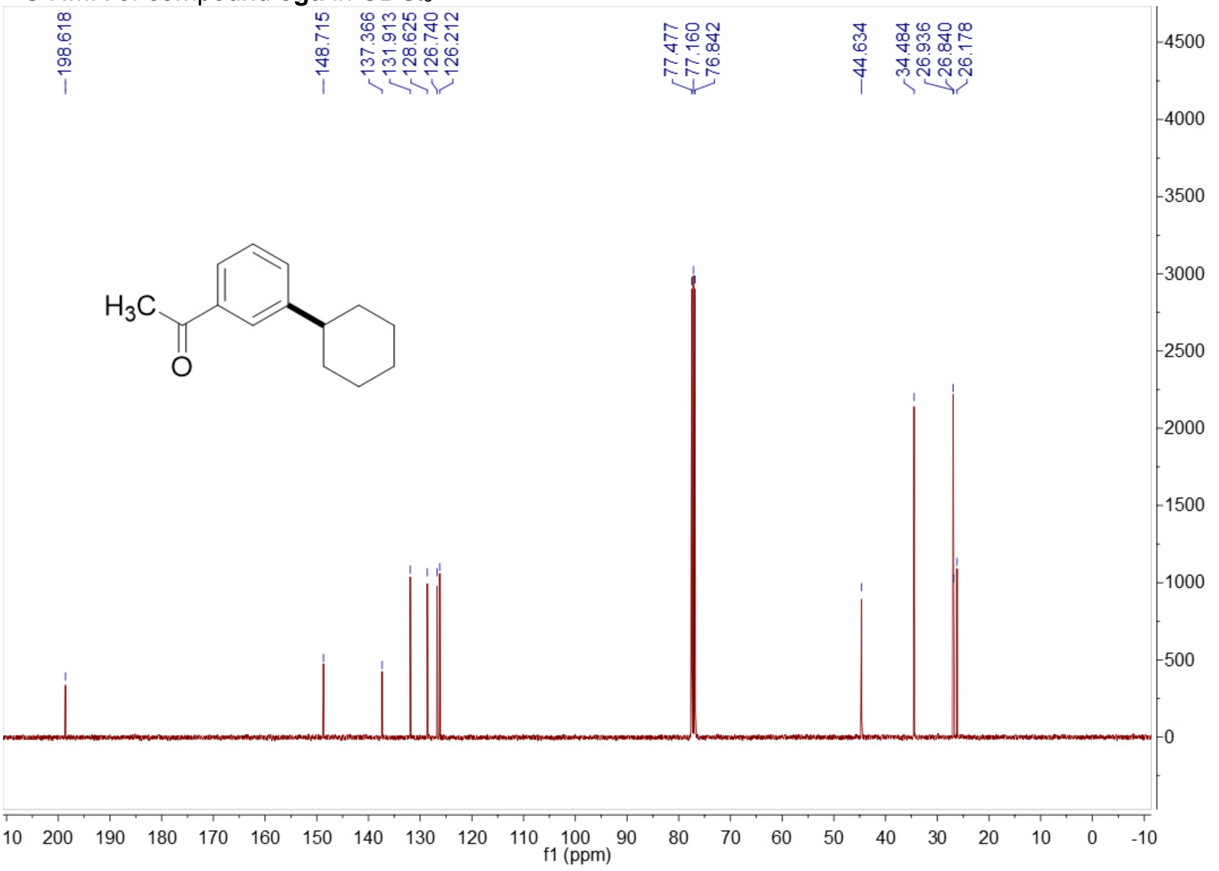

^{1}H NMR of compound **3ha** in $CDCl_3$

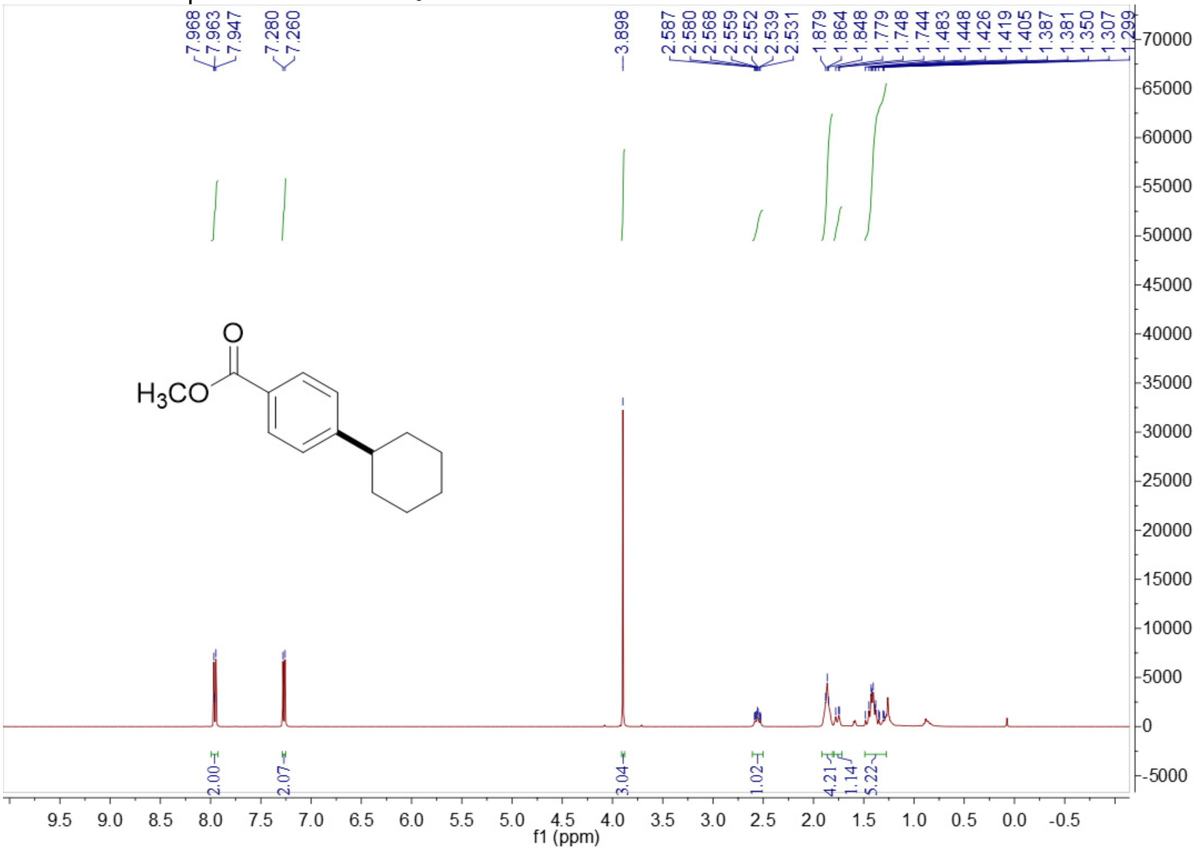

^{13}C NMR of compound **3ha** in $CDCl_3$

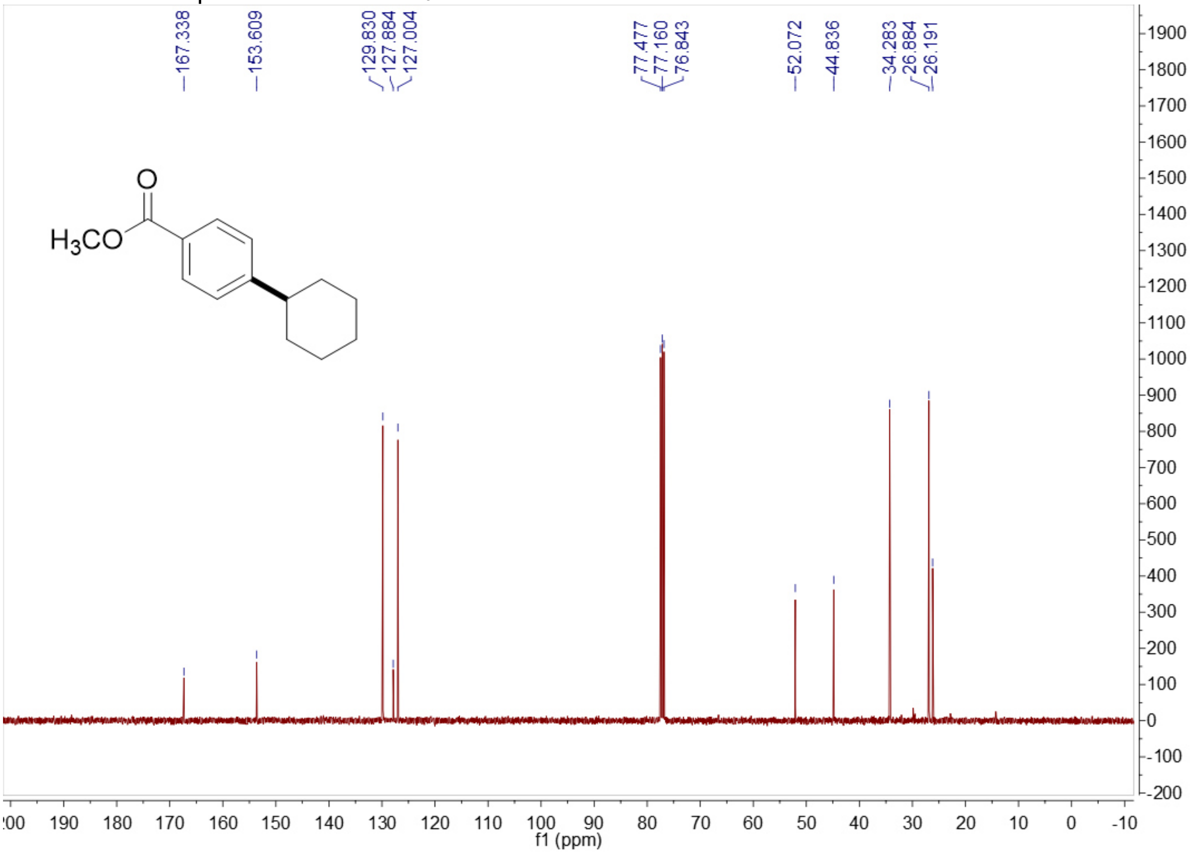

^{1}H NMR of compound **3ia** in $CDCl_3$

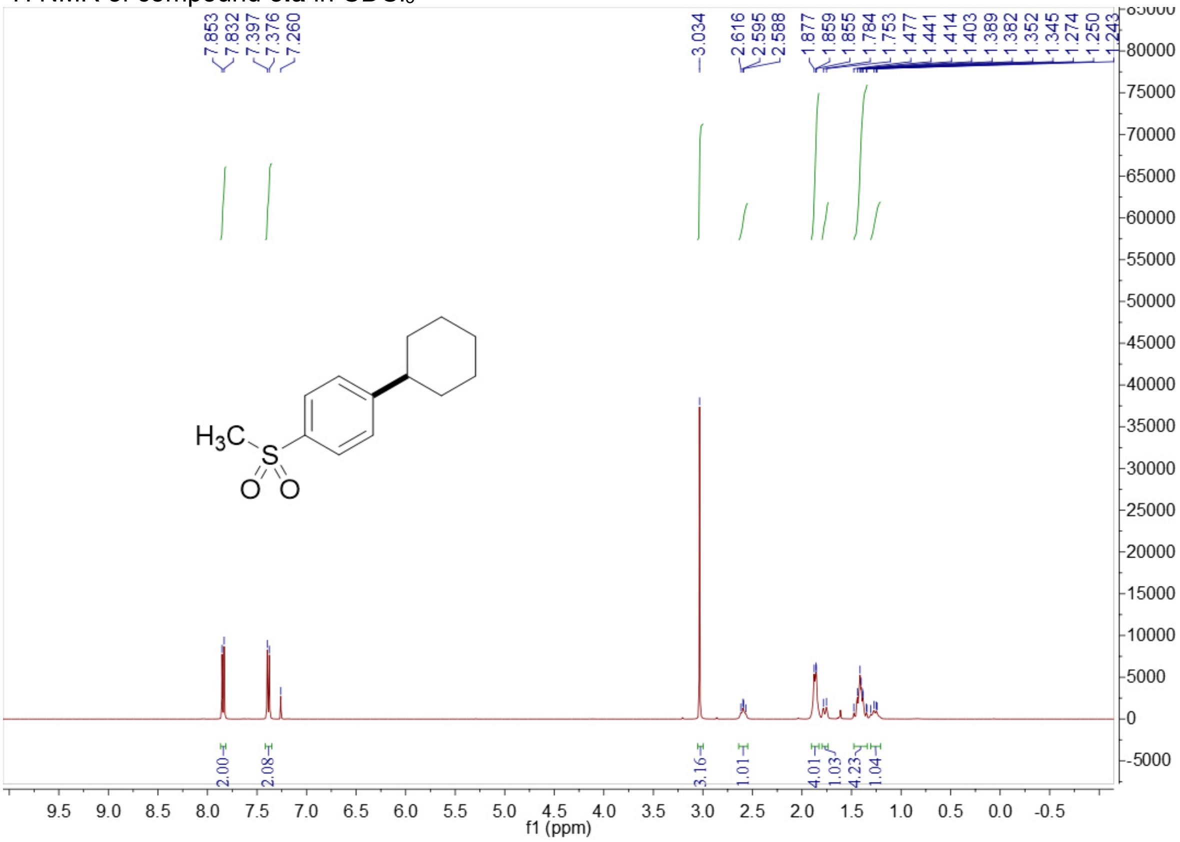

^{13}C NMR of compound **3ia** in $CDCl_3$

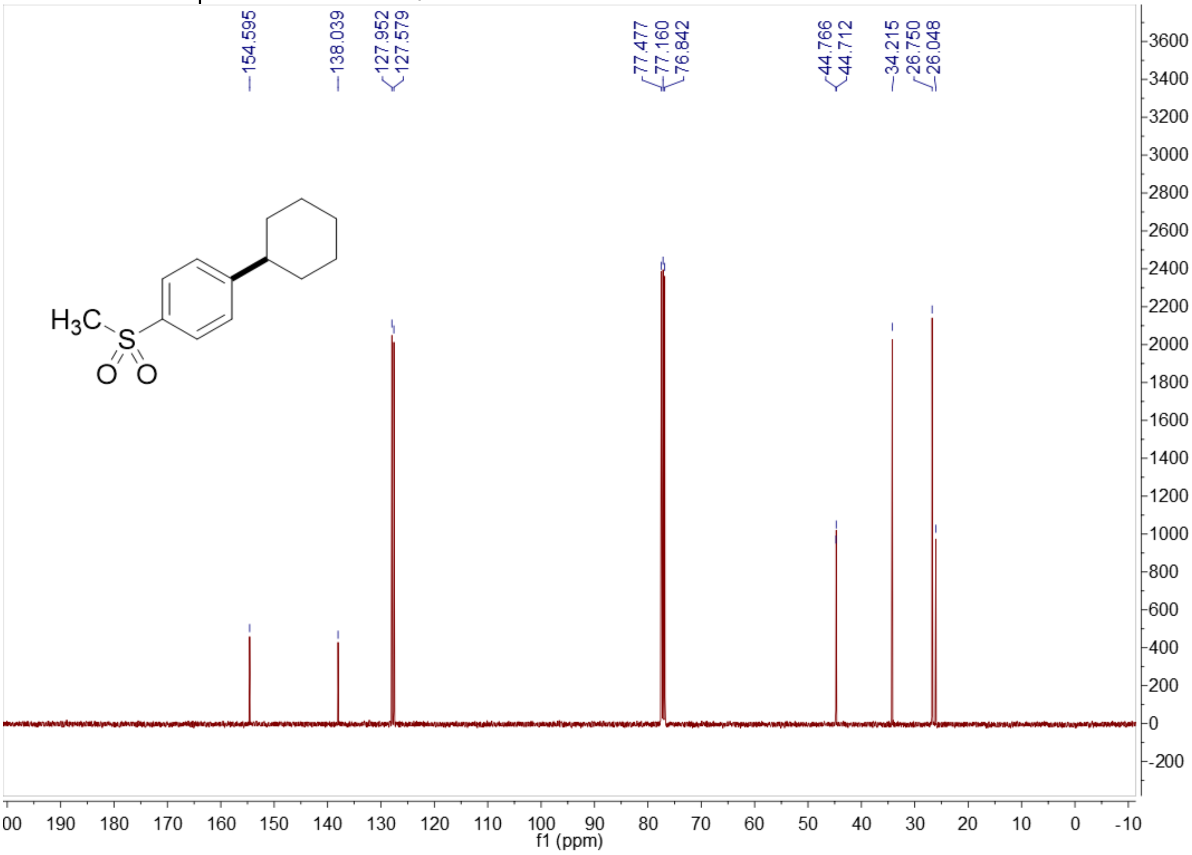

^{1}H NMR of compound **3ja** in $CDCl_3$

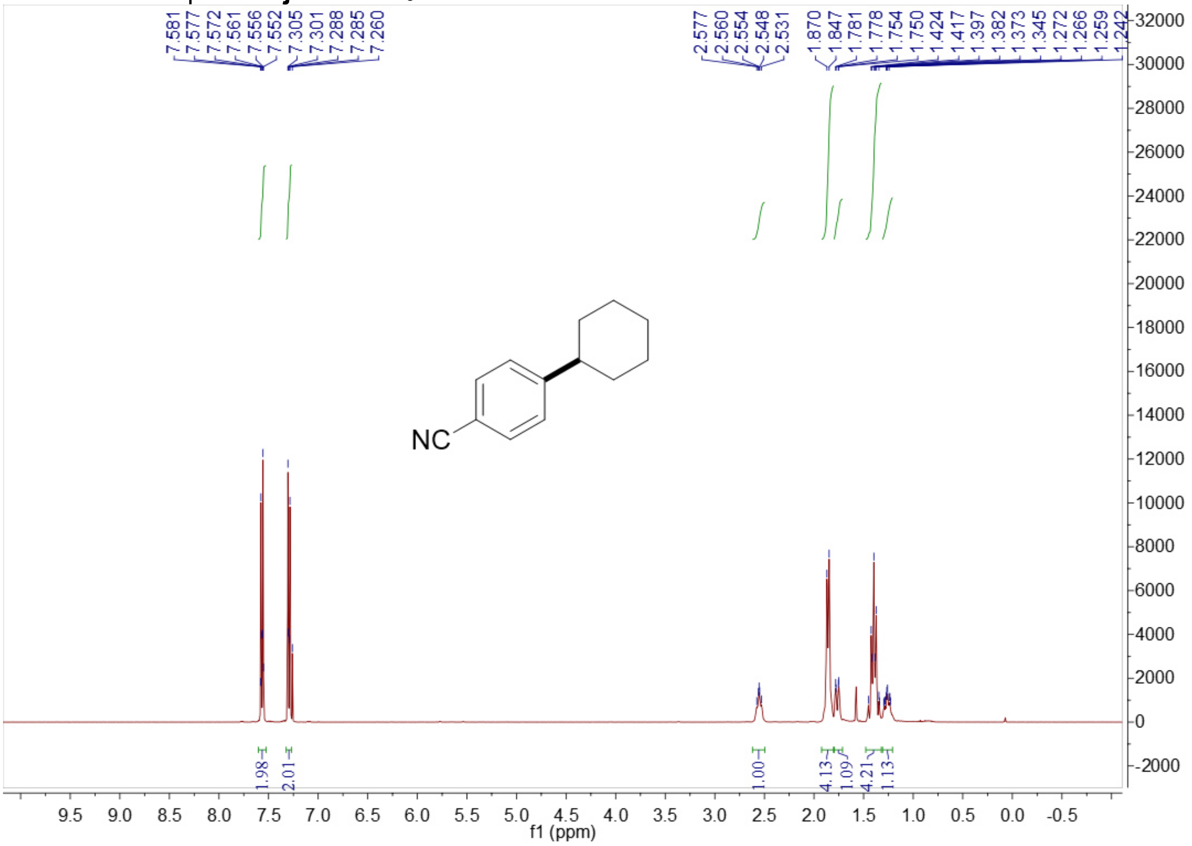

^{13}C NMR of compound **3ja** in $CDCl_3$

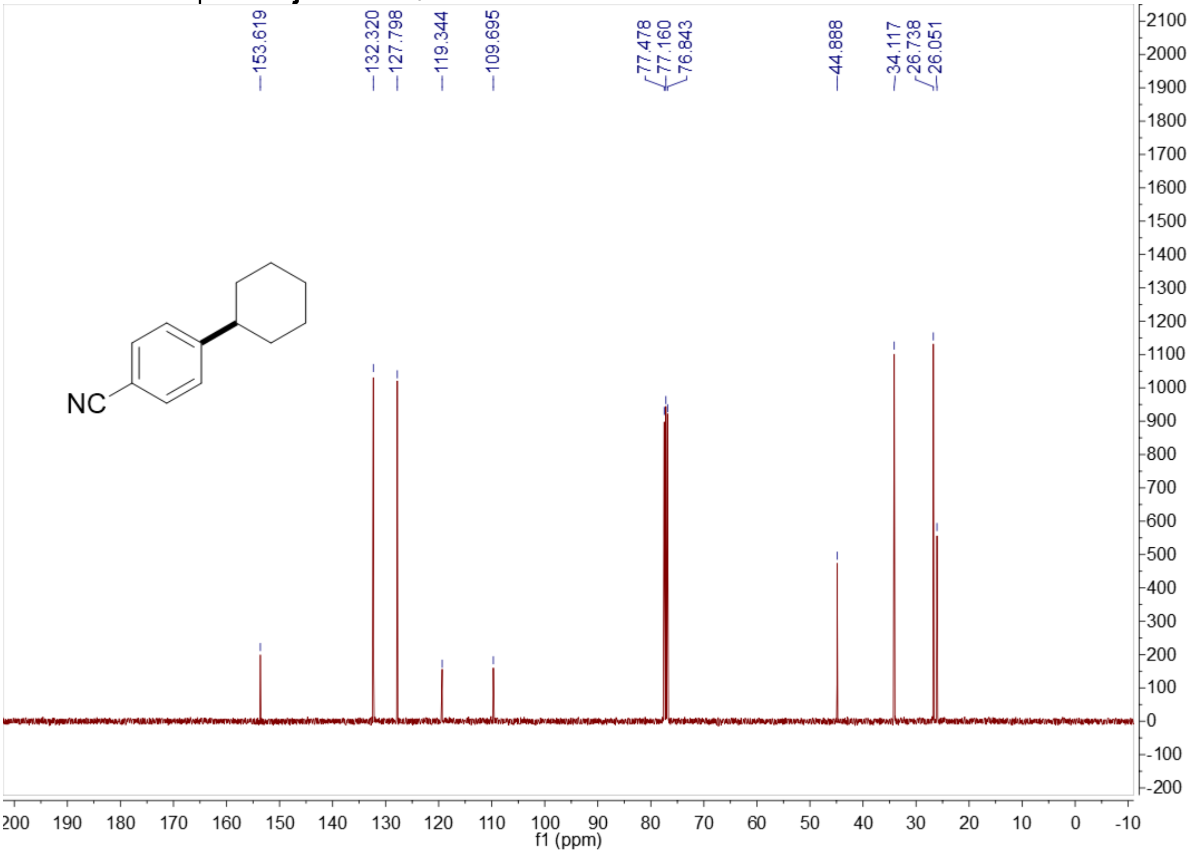

^1H NMR of compound **3ka** in $CDCl_3$

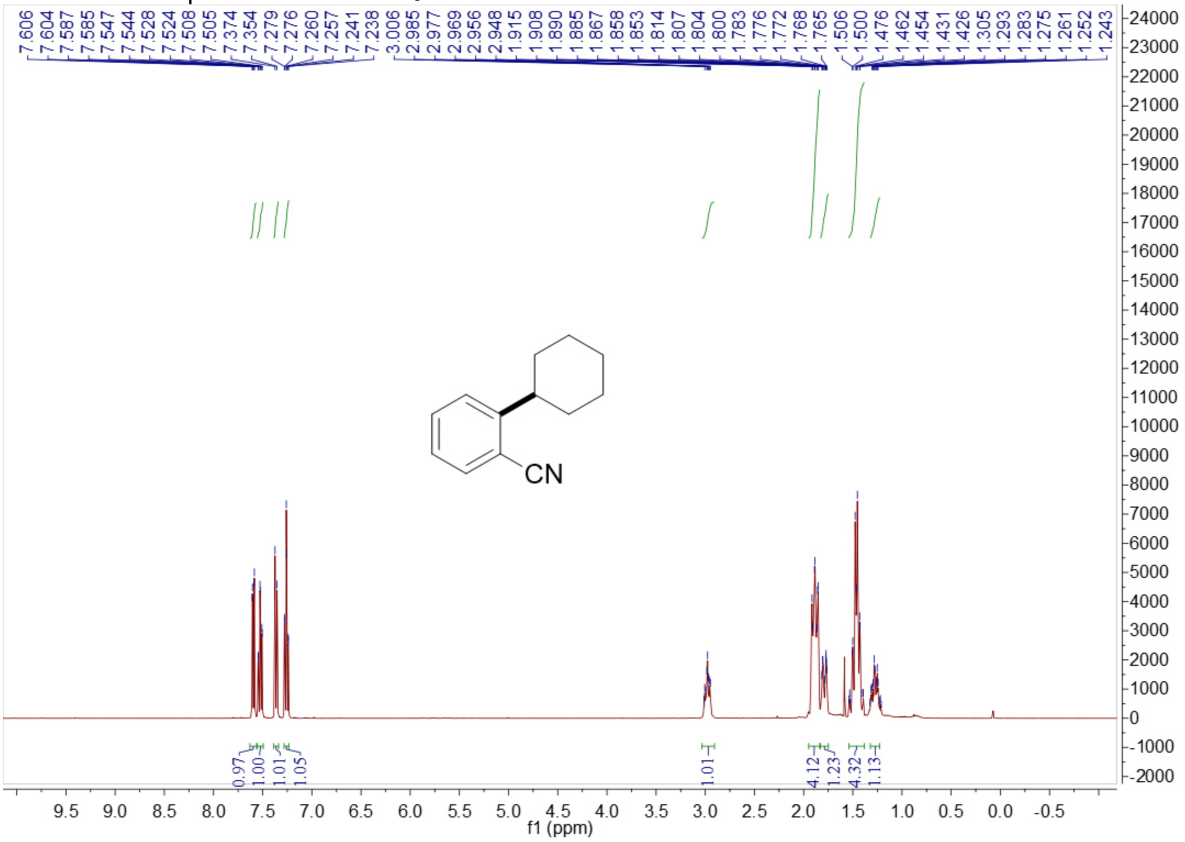

^{13}C NMR of compound **3ka** in $CDCl_3$

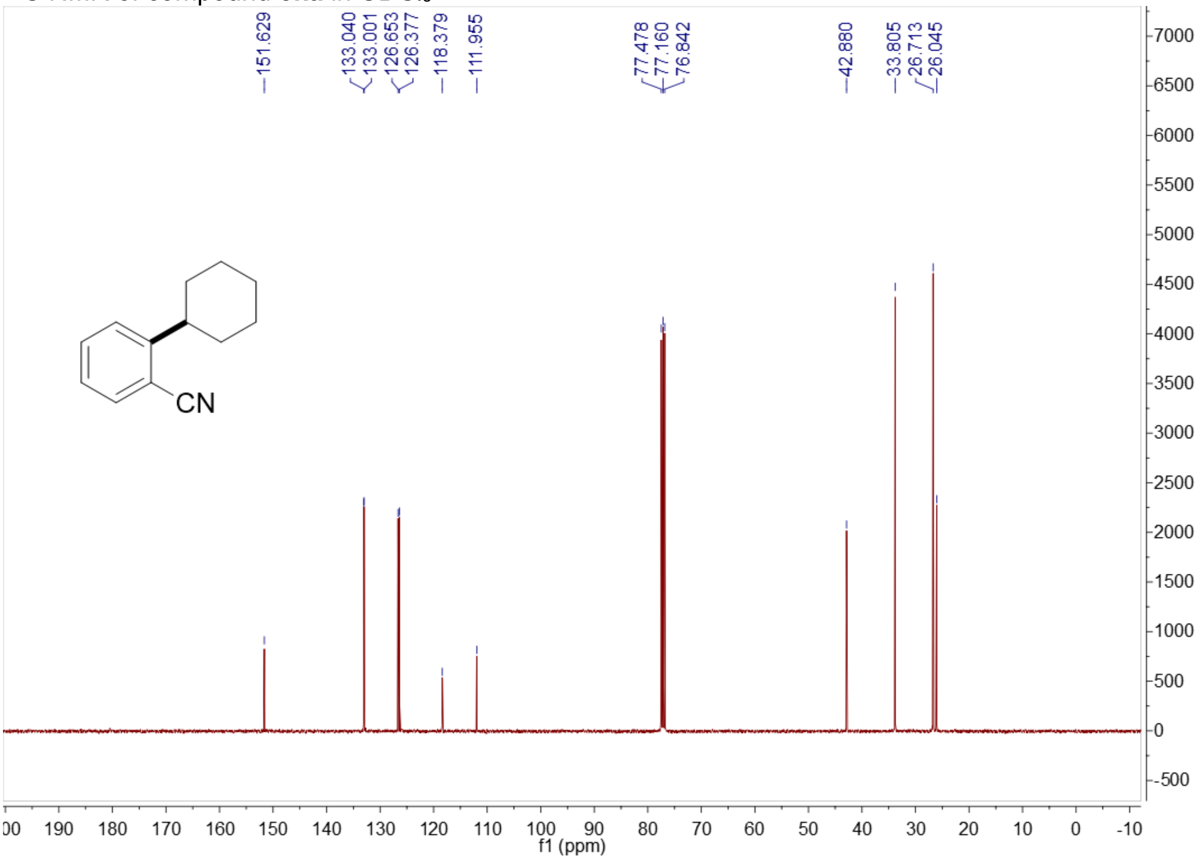

^{1}H NMR of compound **3la** in $CDCl_3$

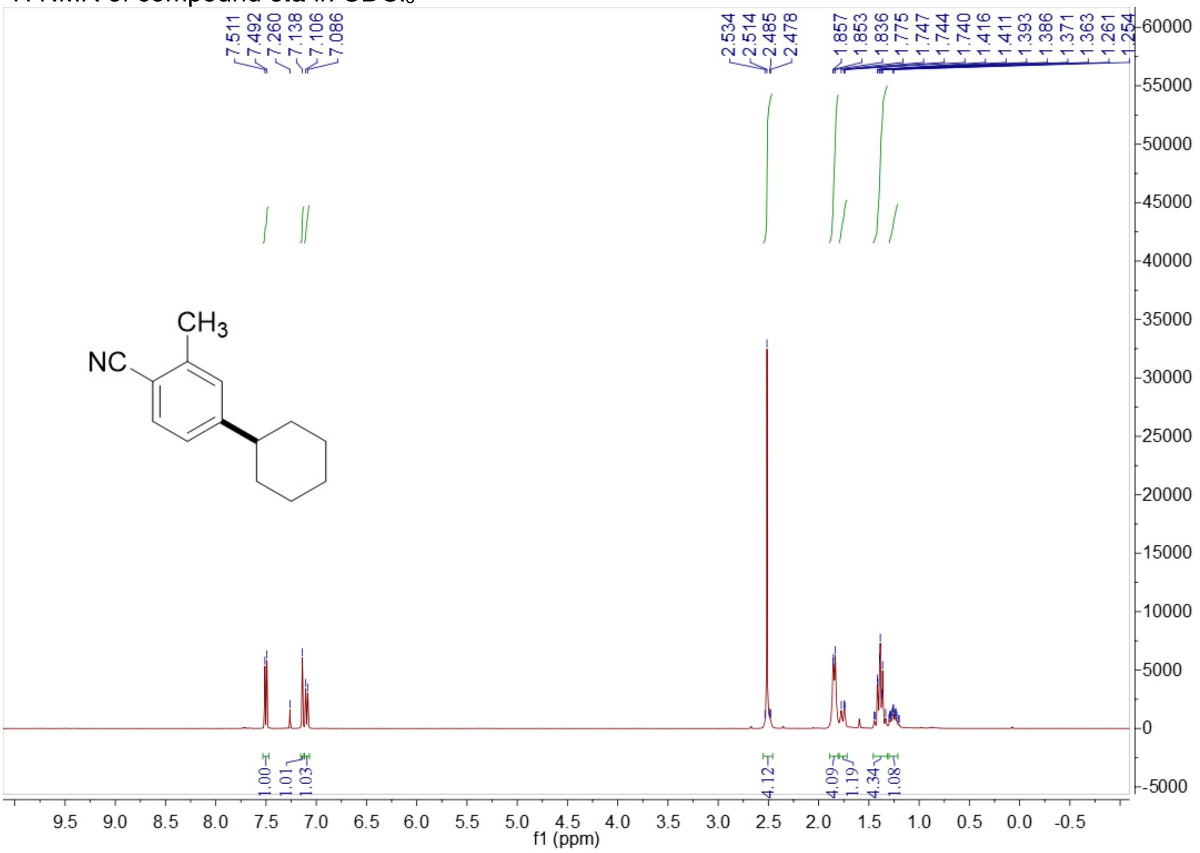

^{13}C NMR of compound **3la** in $CDCl_3$

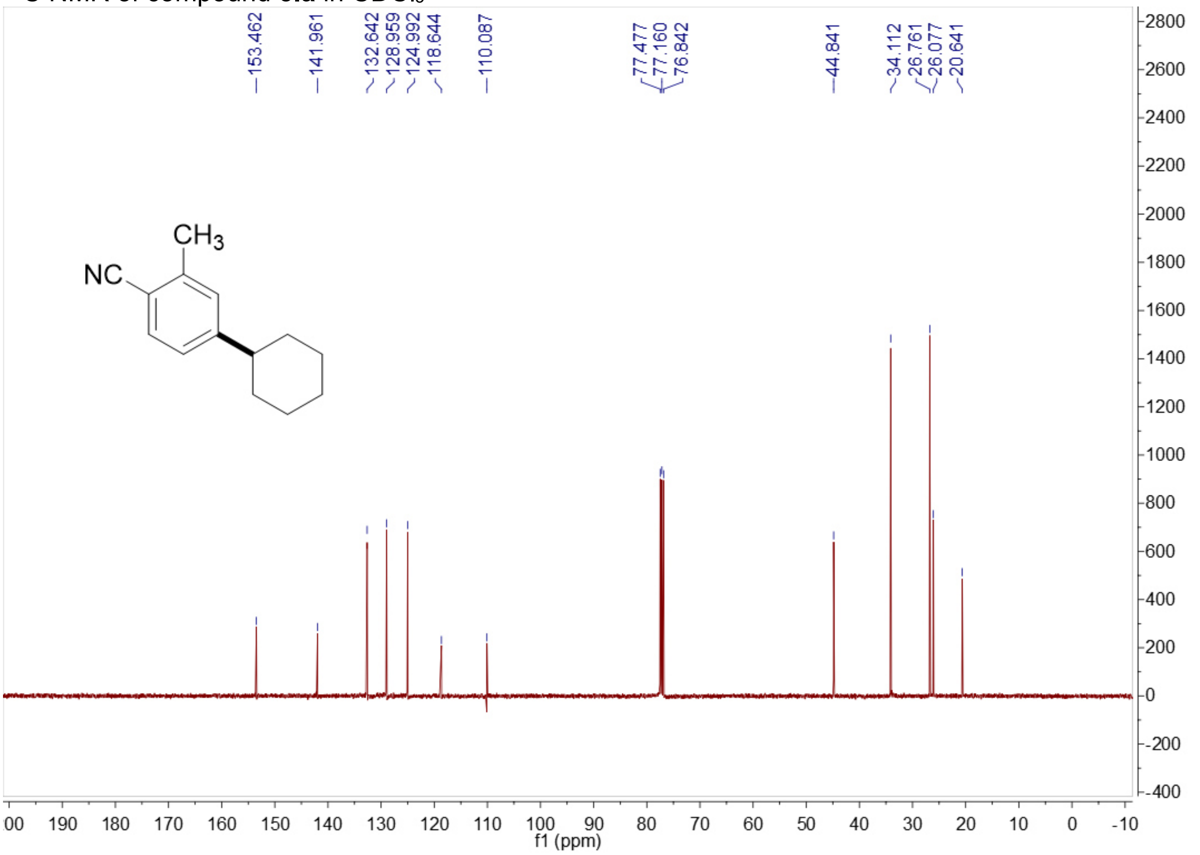

^{1}H NMR of compound **3ma** in $CDCl_3$

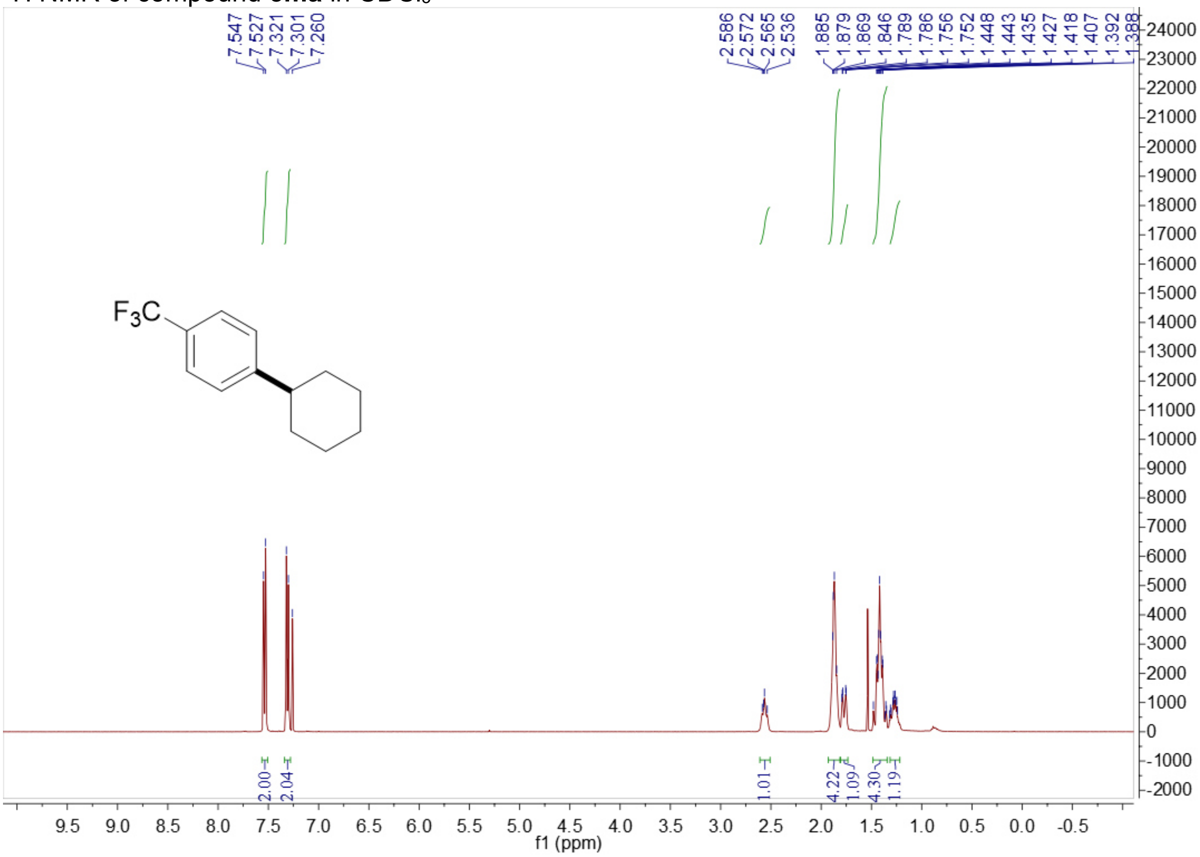

^{13}C NMR of compound **3ma** in $CDCl_3$

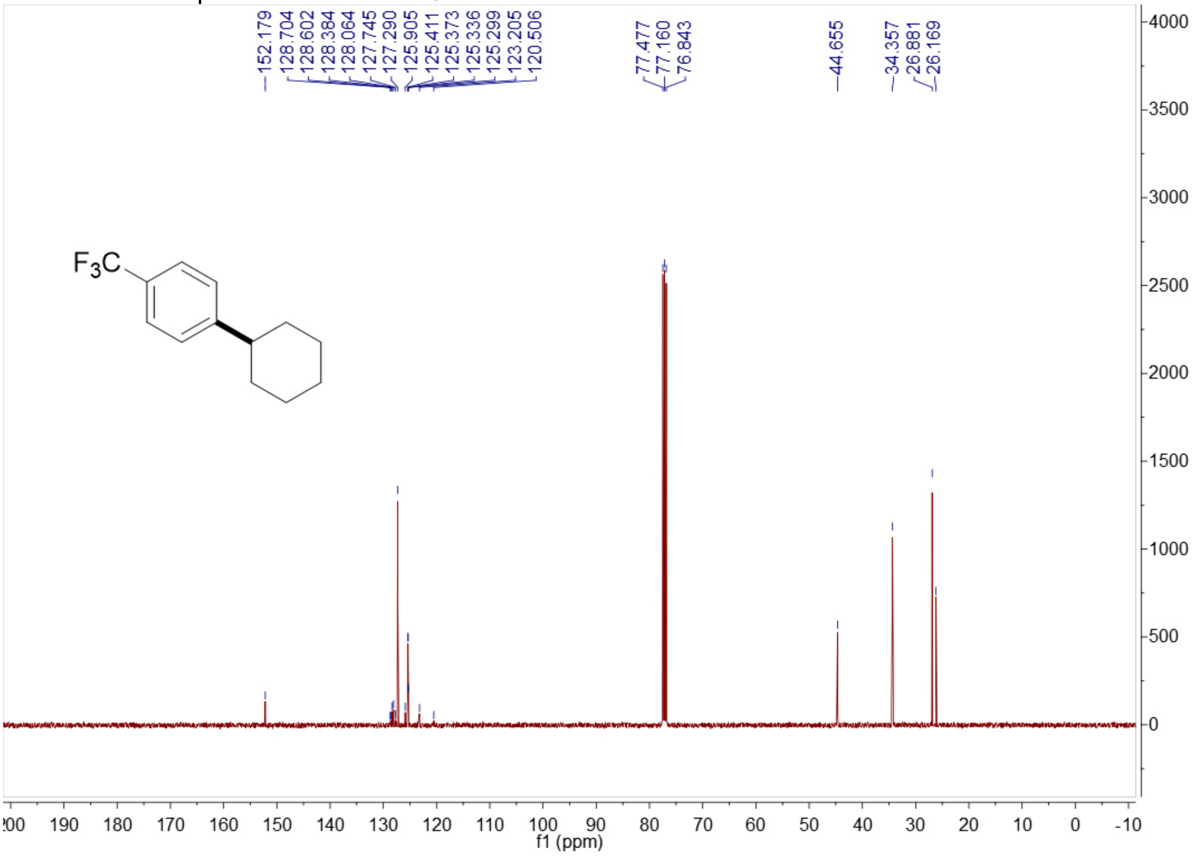

^{1}H NMR of compound **3na** in $CDCl_3$

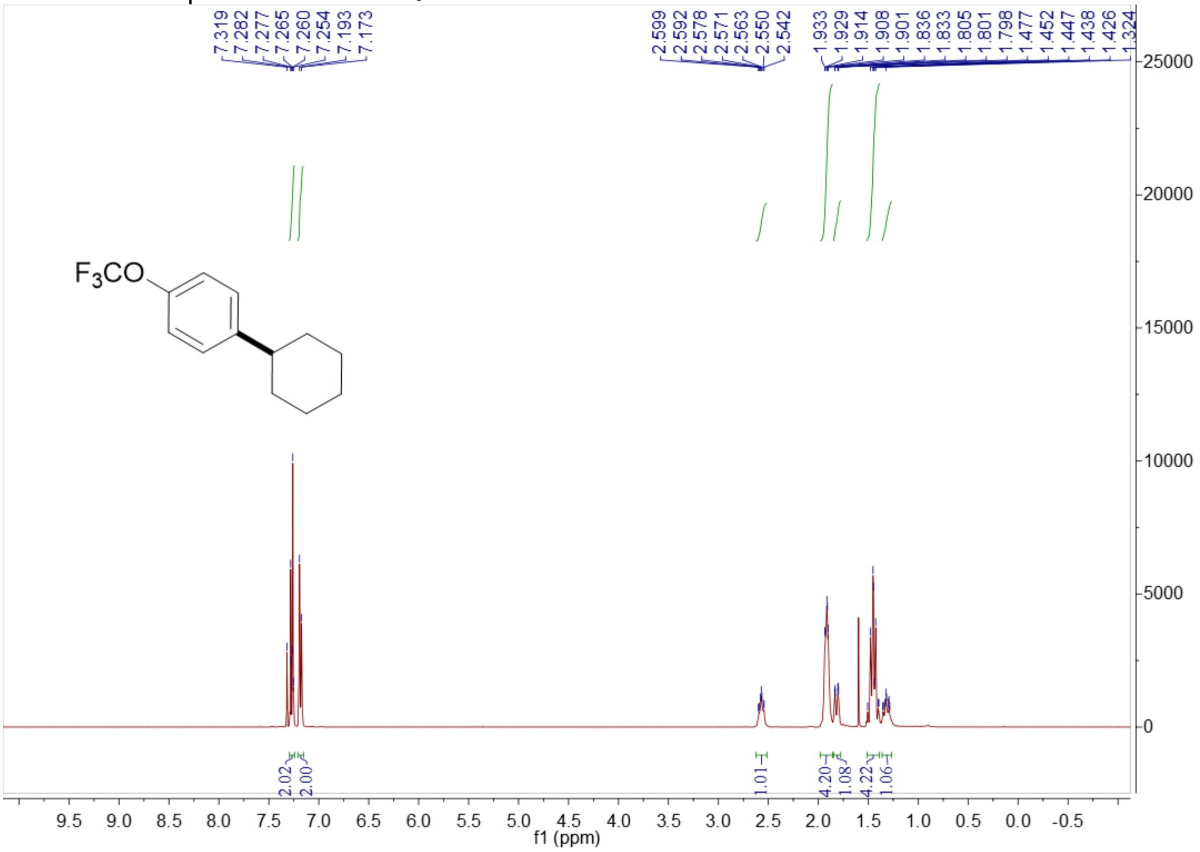

^{13}C NMR of compound **3na** in $CDCl_3$

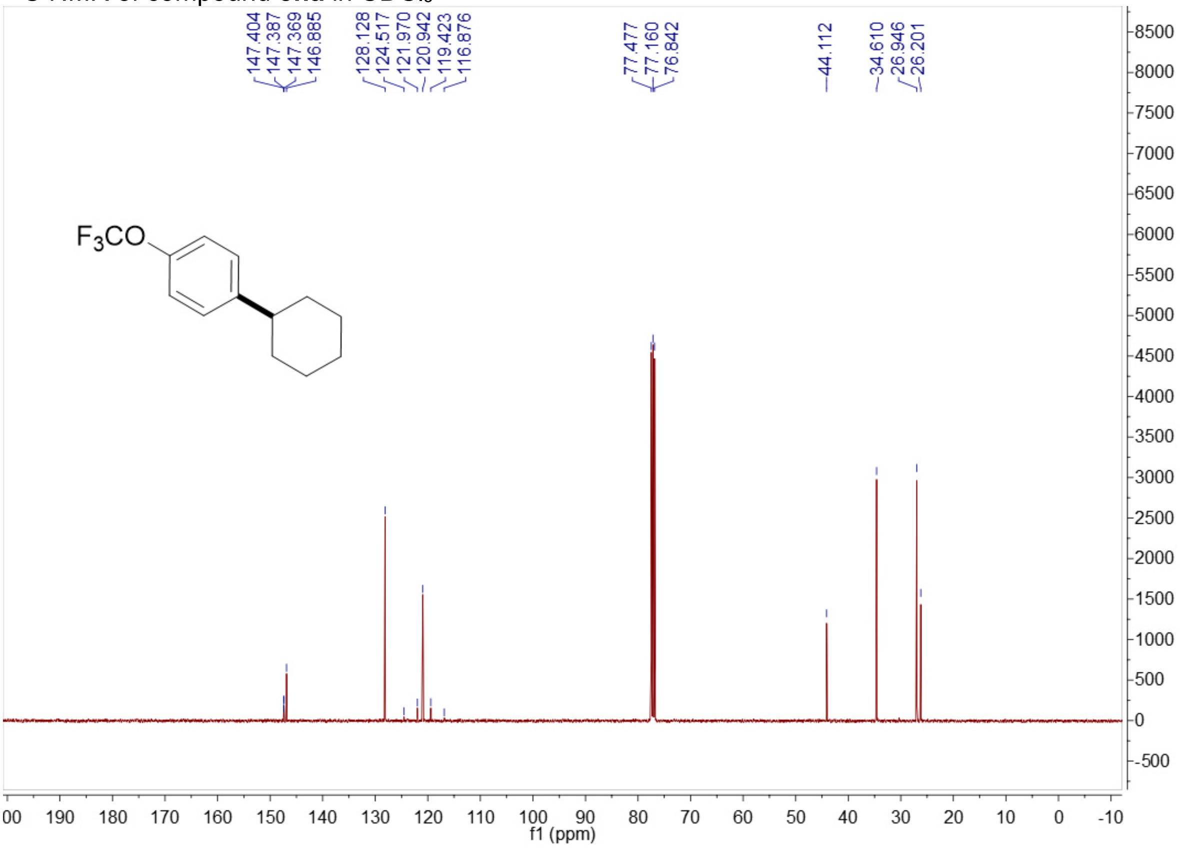

^1H NMR of compound **3oa** in $CDCl_3$

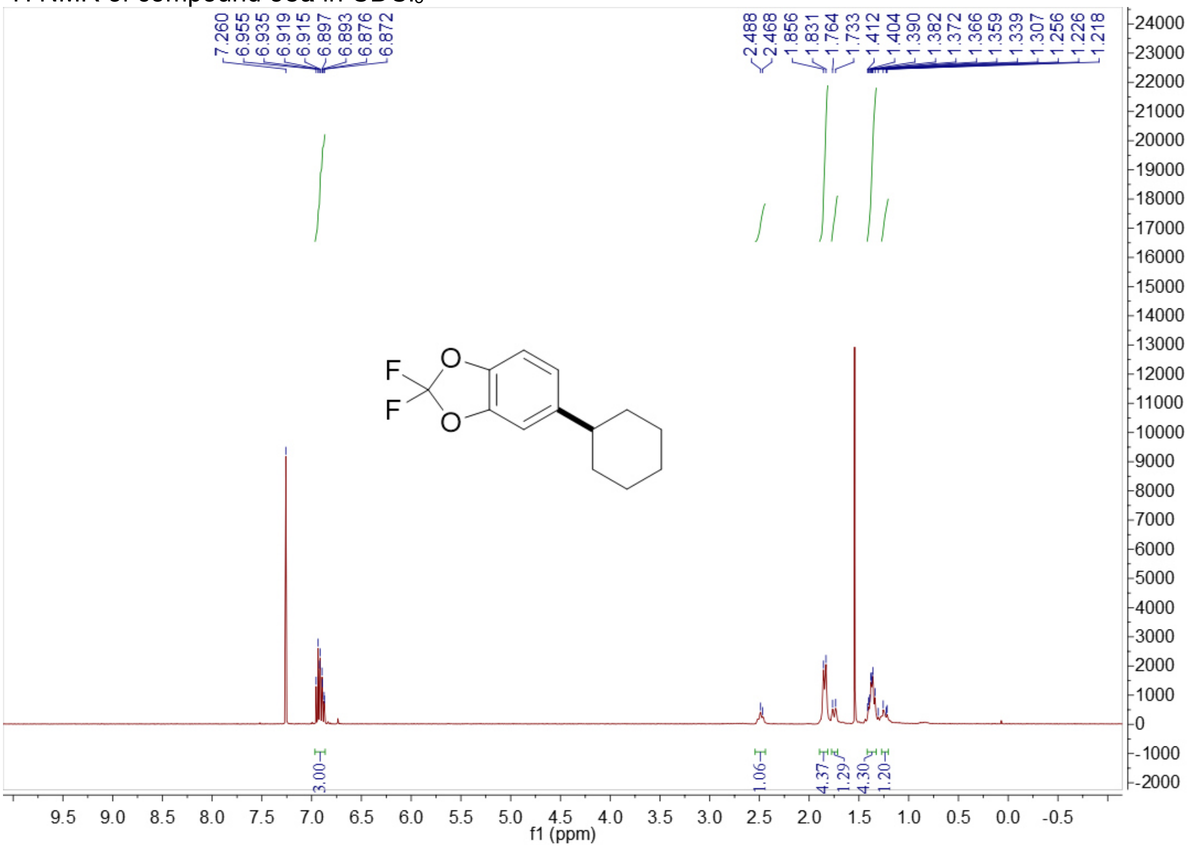

^{13}C NMR of compound **3oa** in $CDCl_3$

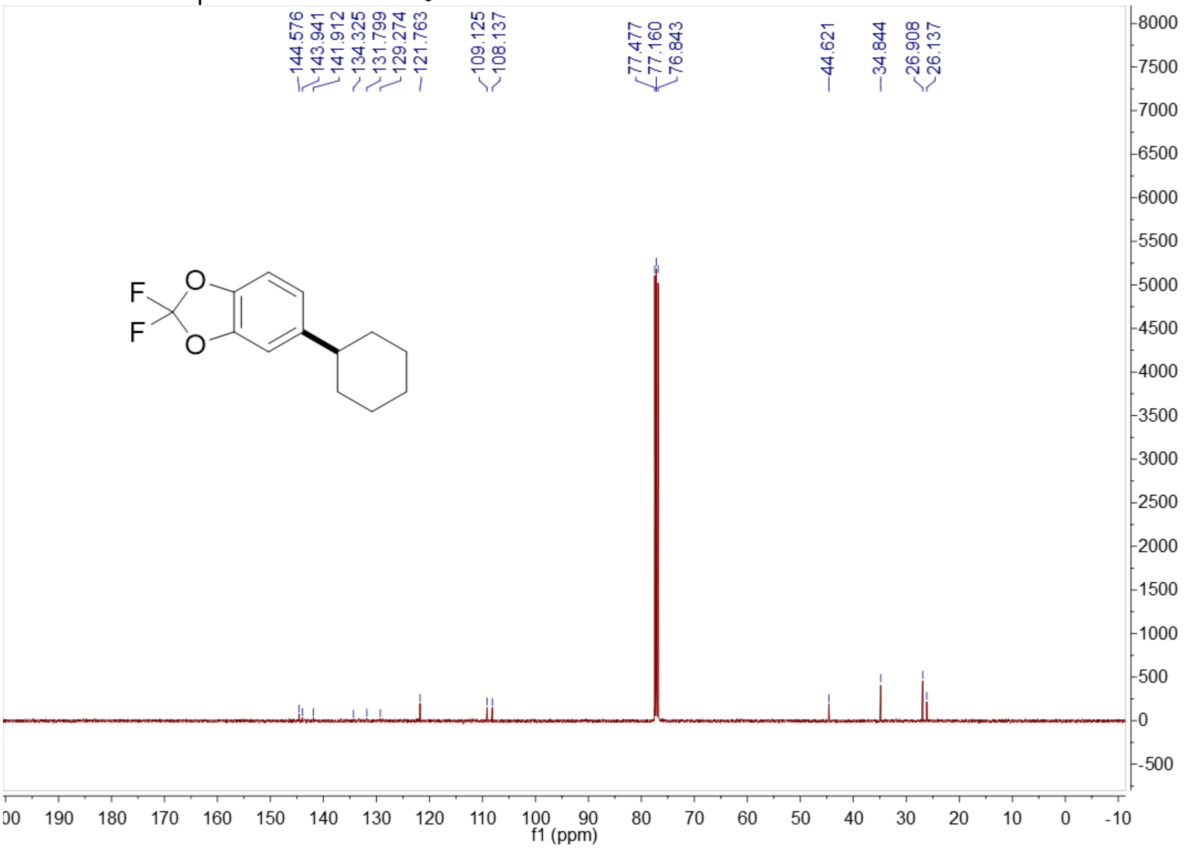

^{1}H NMR of compound **3pa** in $CDCl_3$

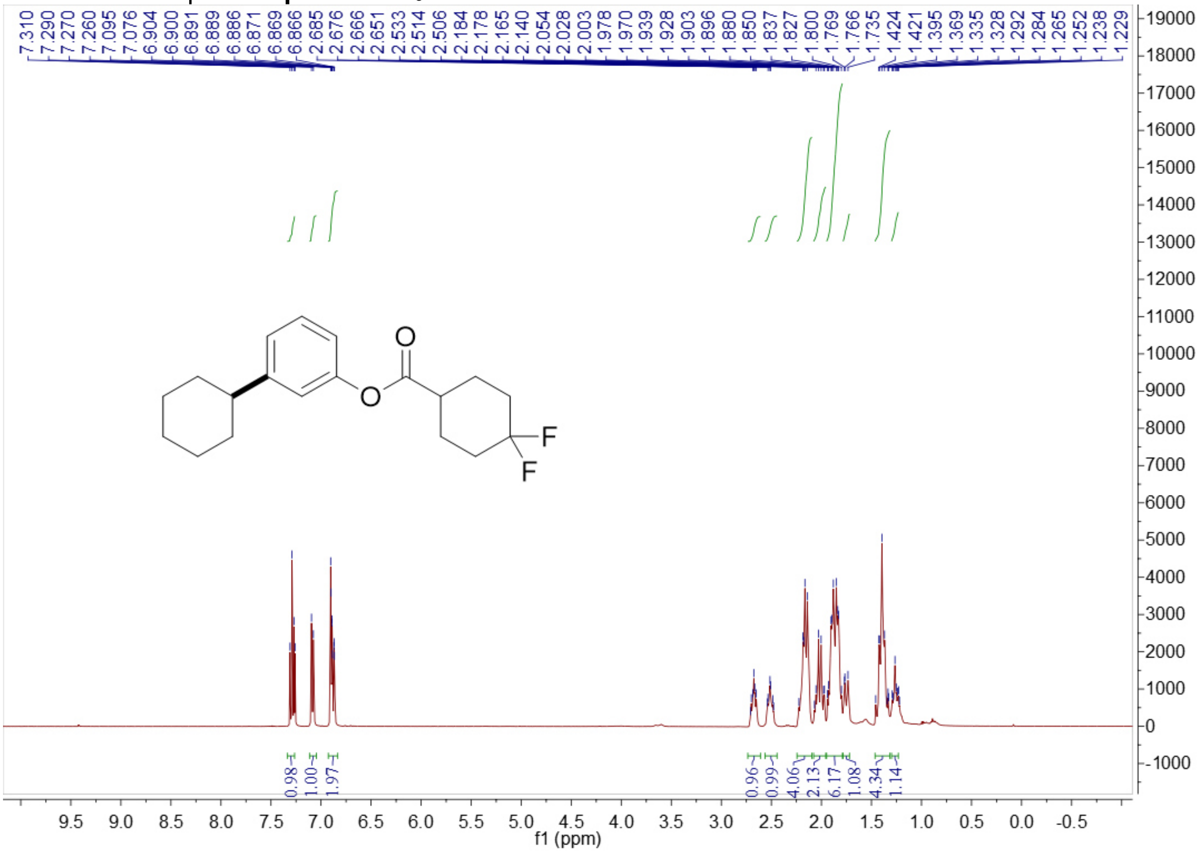

^{13}C NMR of compound **3pa** in $CDCl_3$

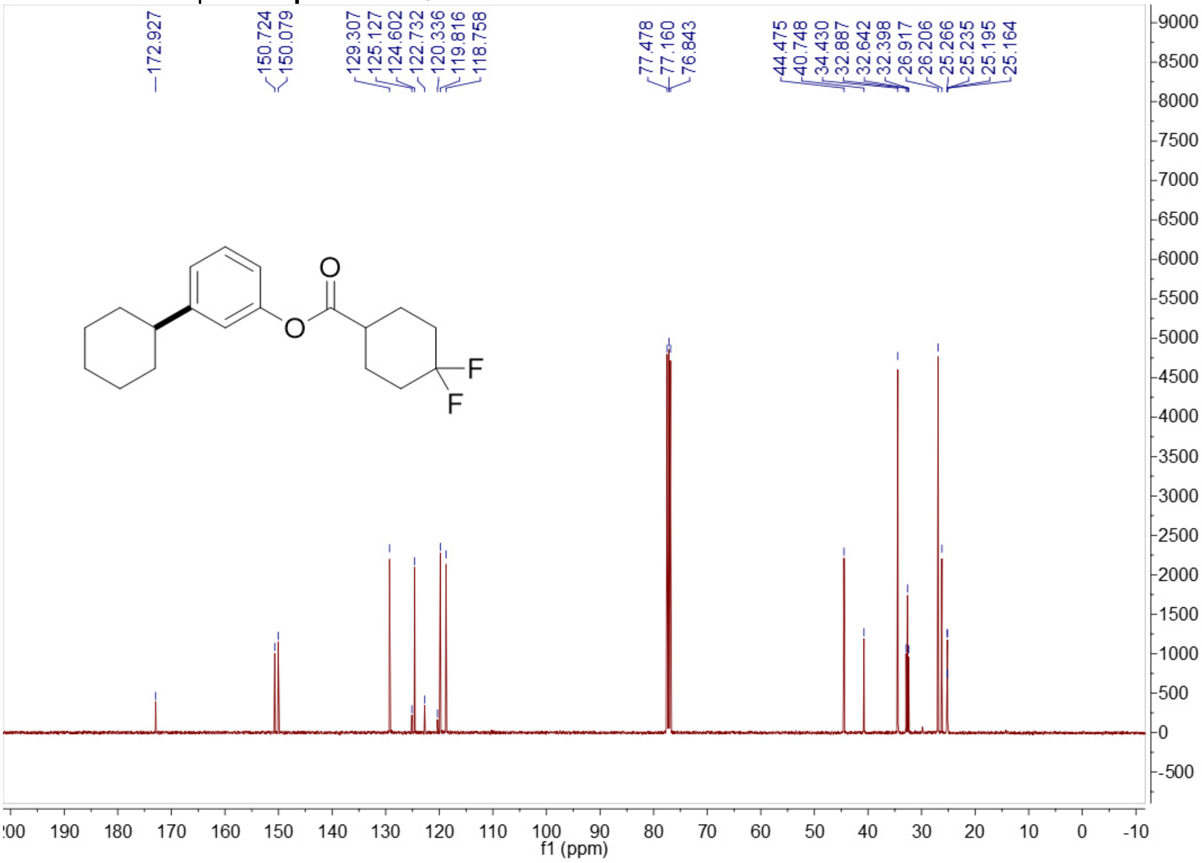

^{1}H NMR of compound **3qa** in $CDCl_3$

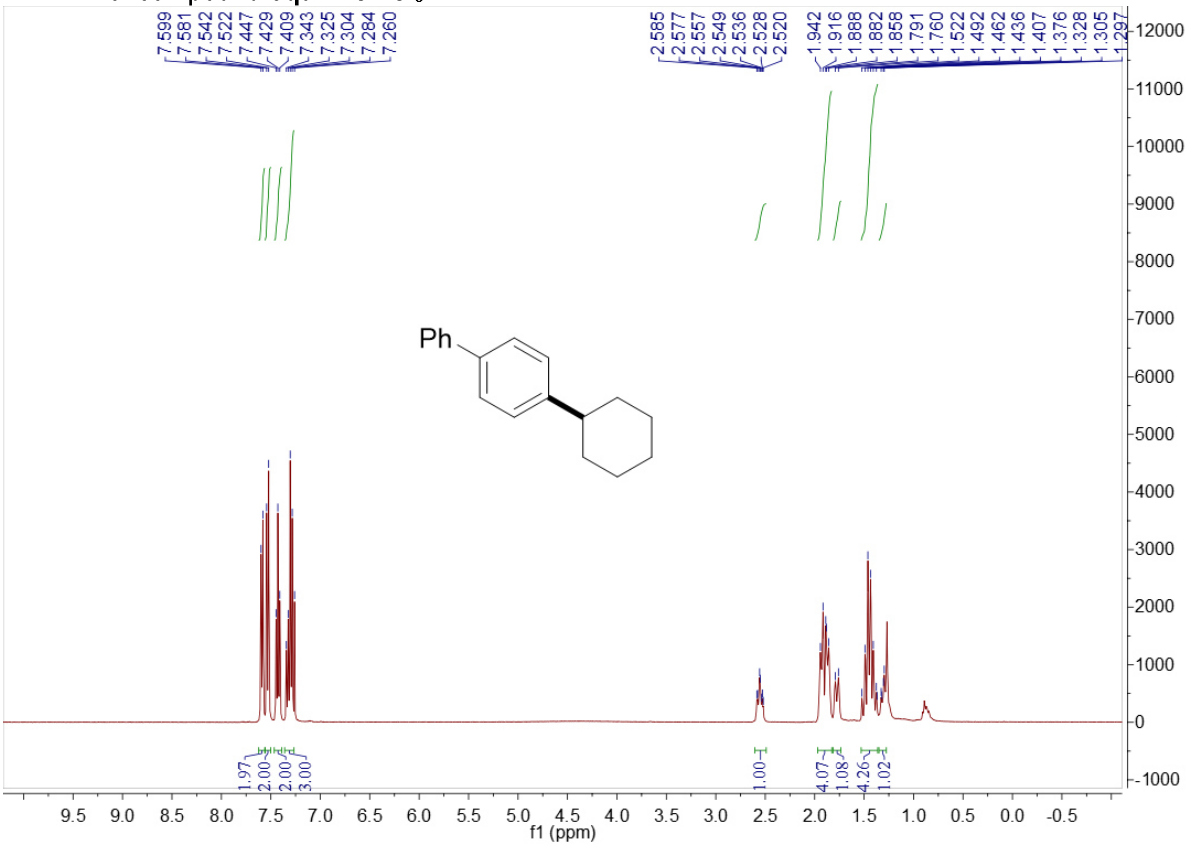

^{13}C NMR of compound **3qa** in $CDCl_3$

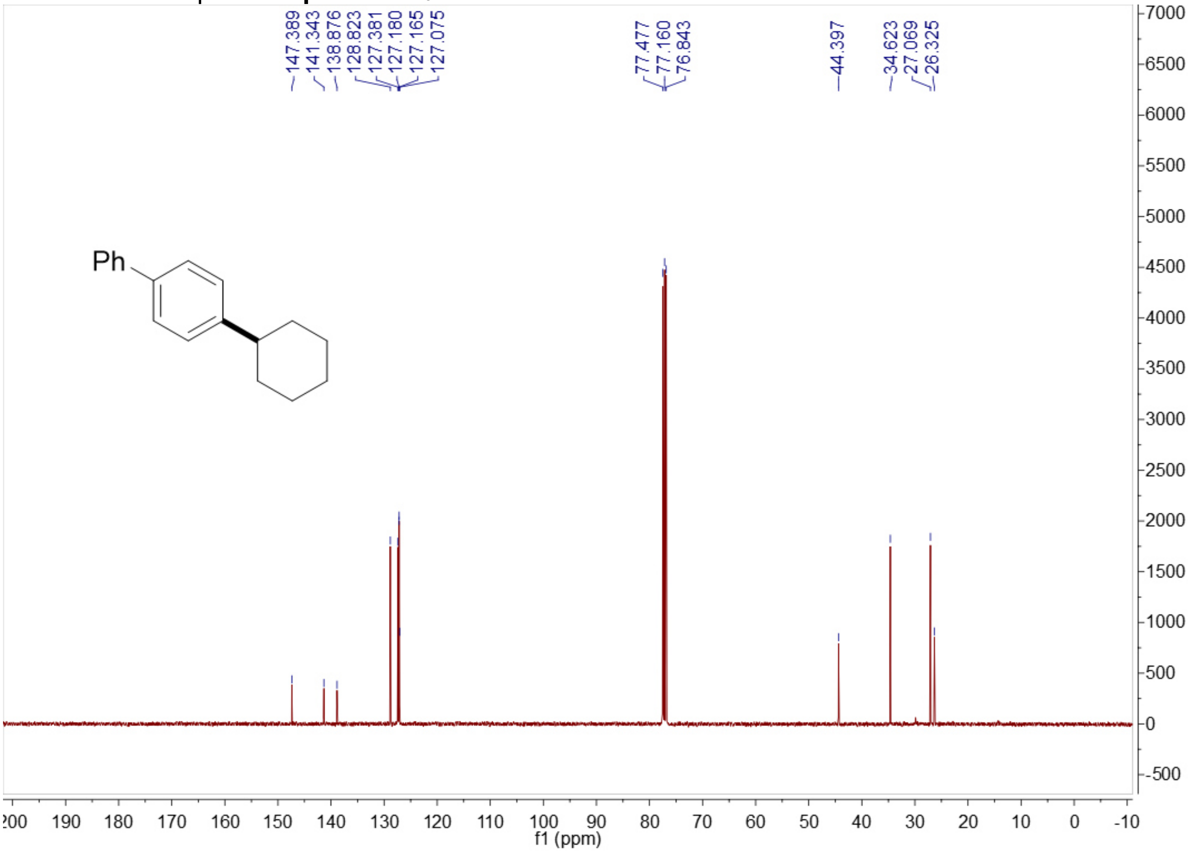

^{1}H NMR of compound **3ra** in $CDCl_3$

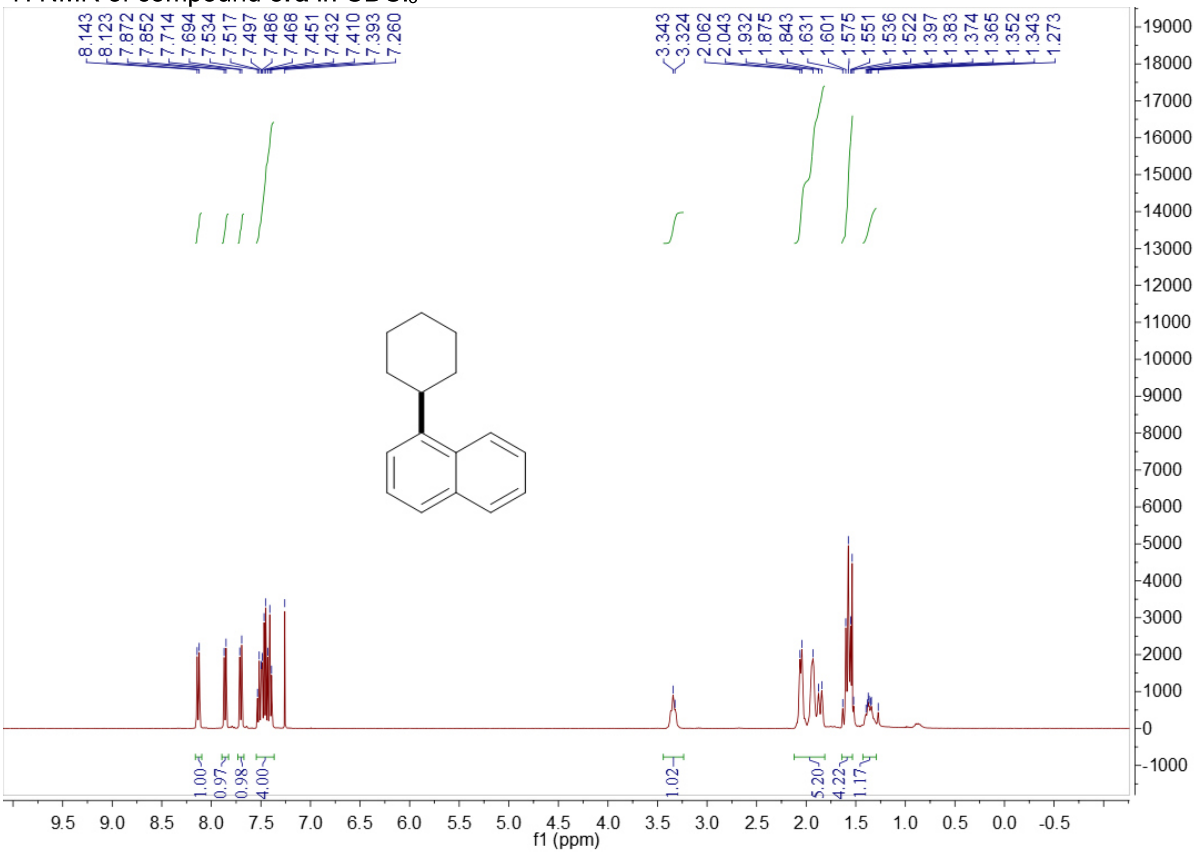

^{13}C NMR of compound **3ra** in $CDCl_3$

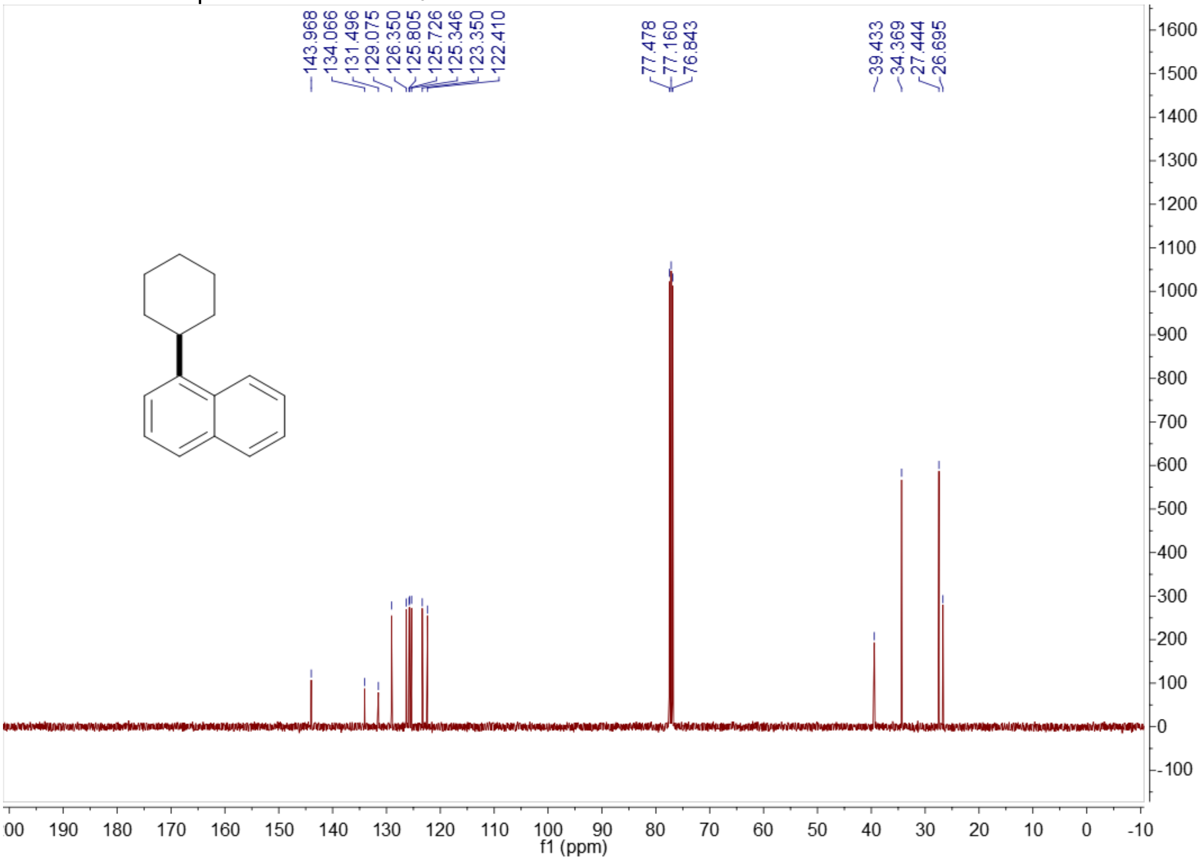

^{1}H NMR of compound **3sa** in $CDCl_3$

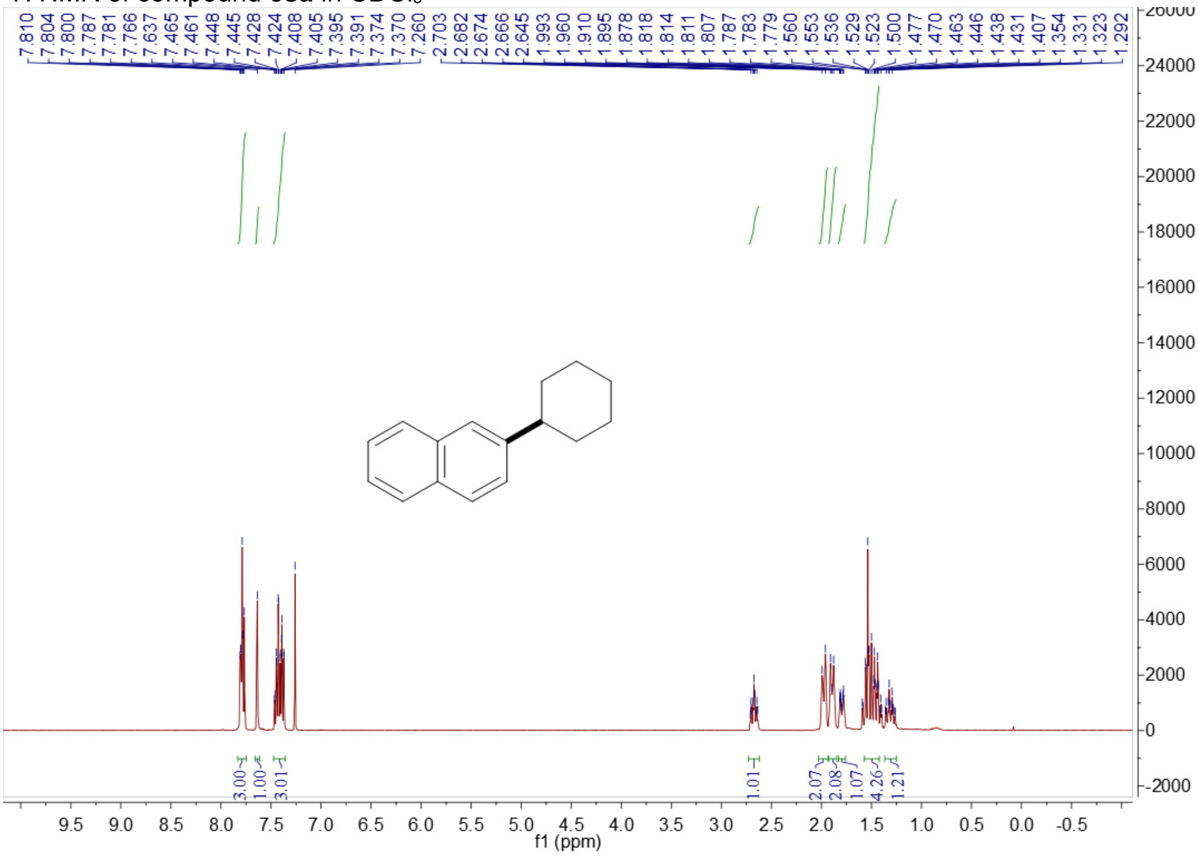

^{13}C NMR of compound **3sa** in $CDCl_3$

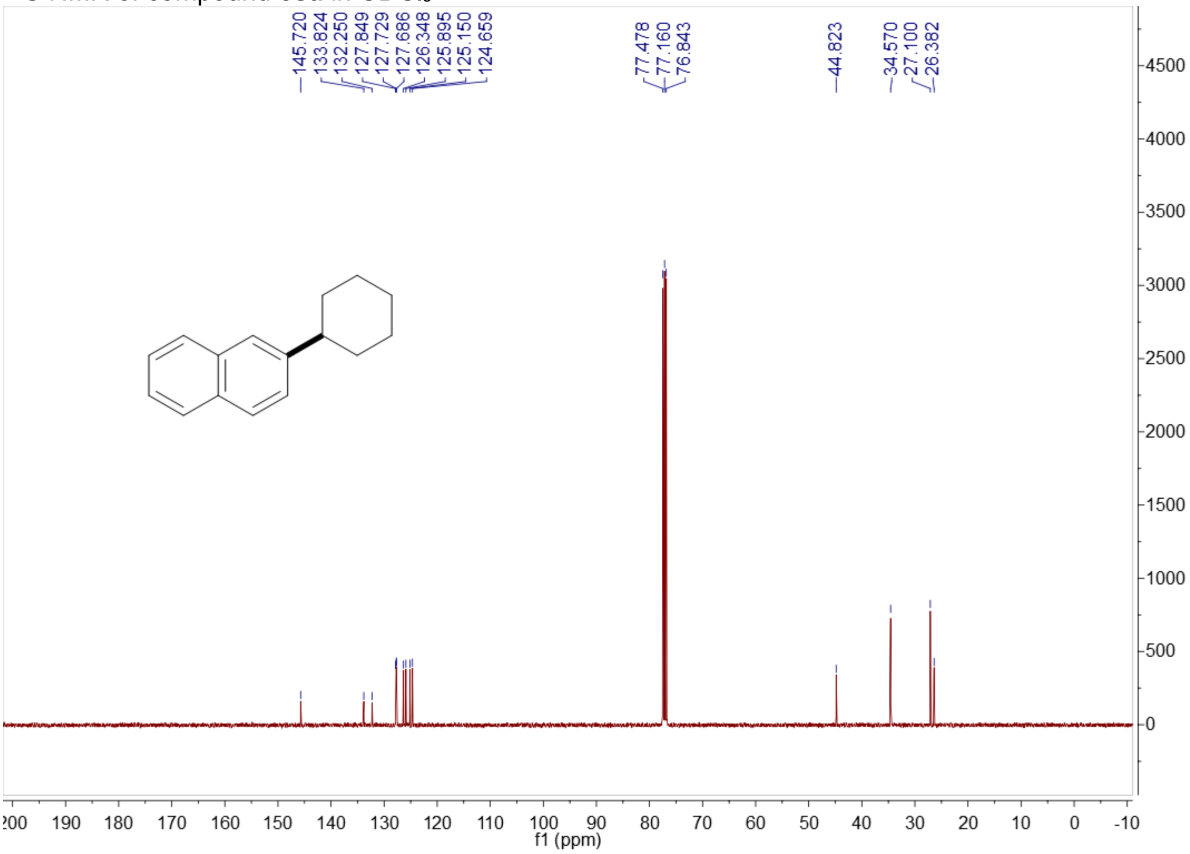

^{1}H NMR of compound **3ta** in $CDCl_3$

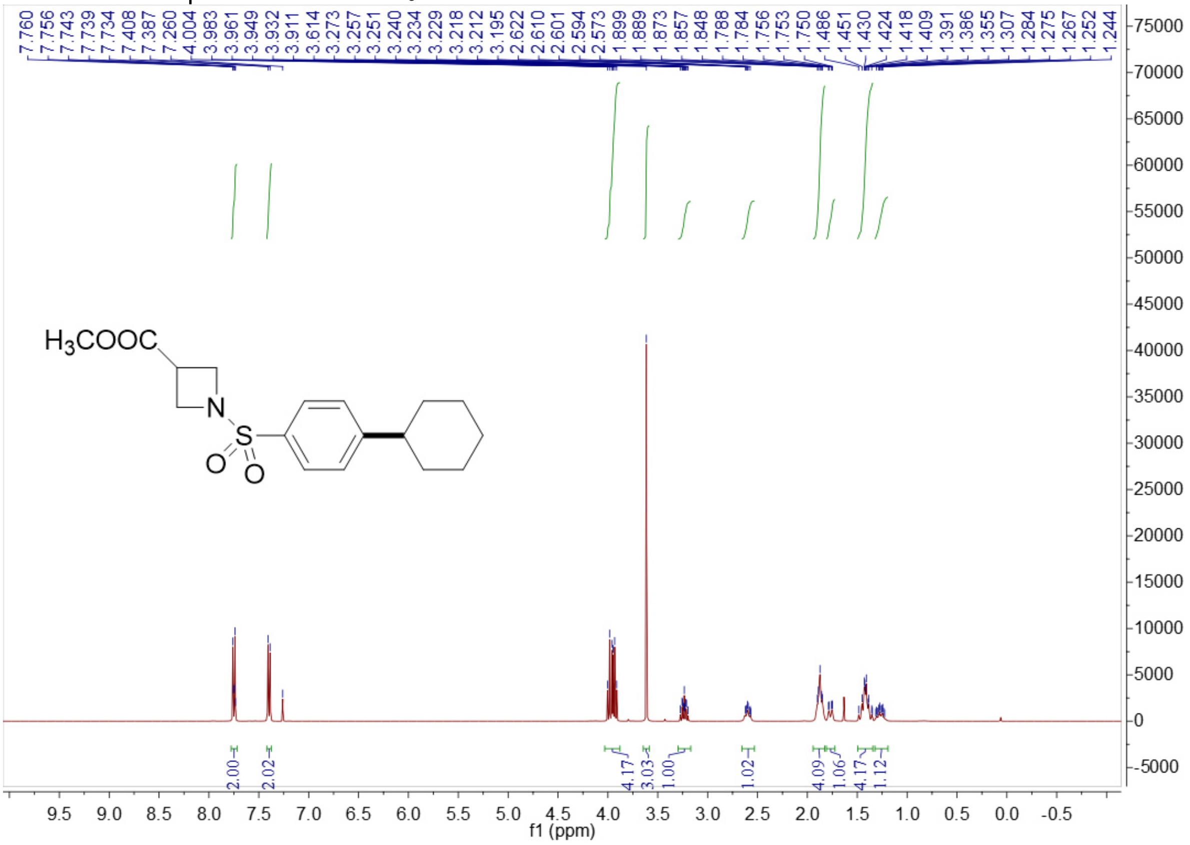

^{13}C NMR of compound **3ta** in $CDCl_3$

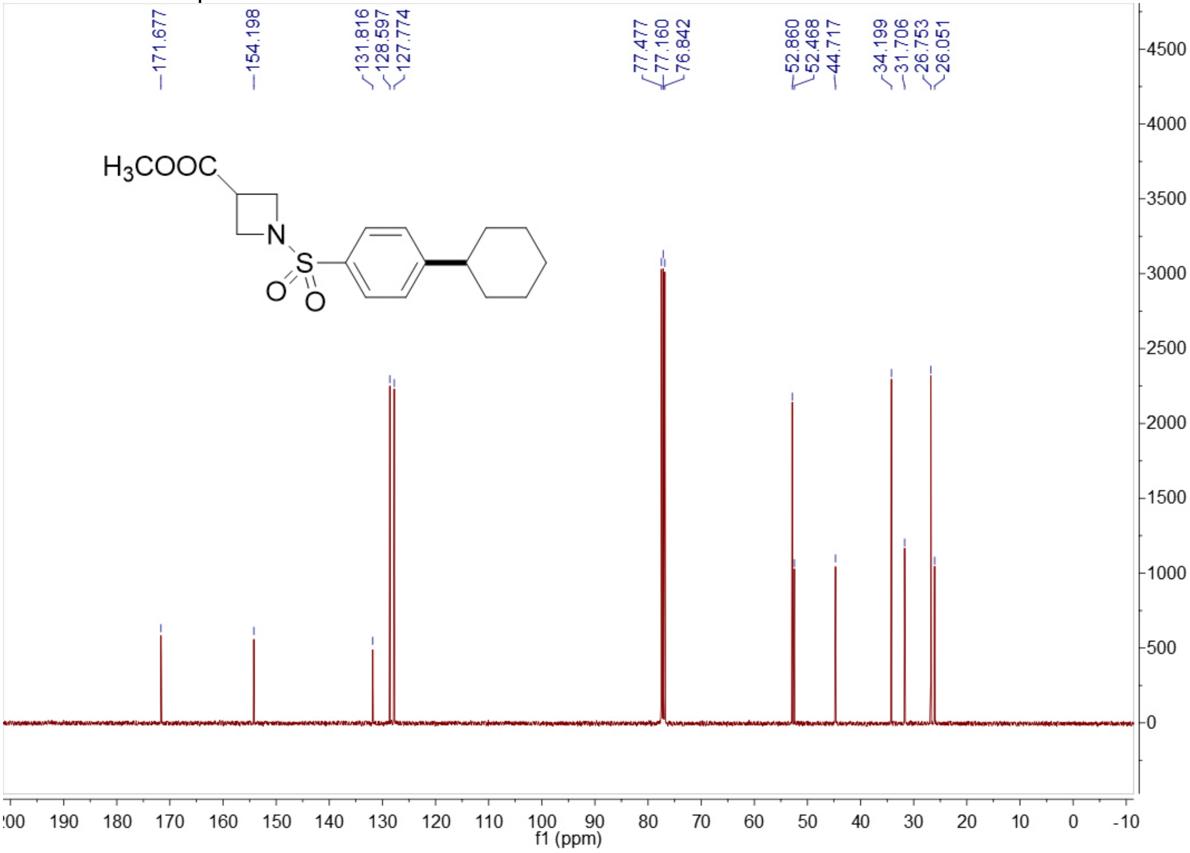

^{1}H NMR of compound **3ua** in $CDCl_3$

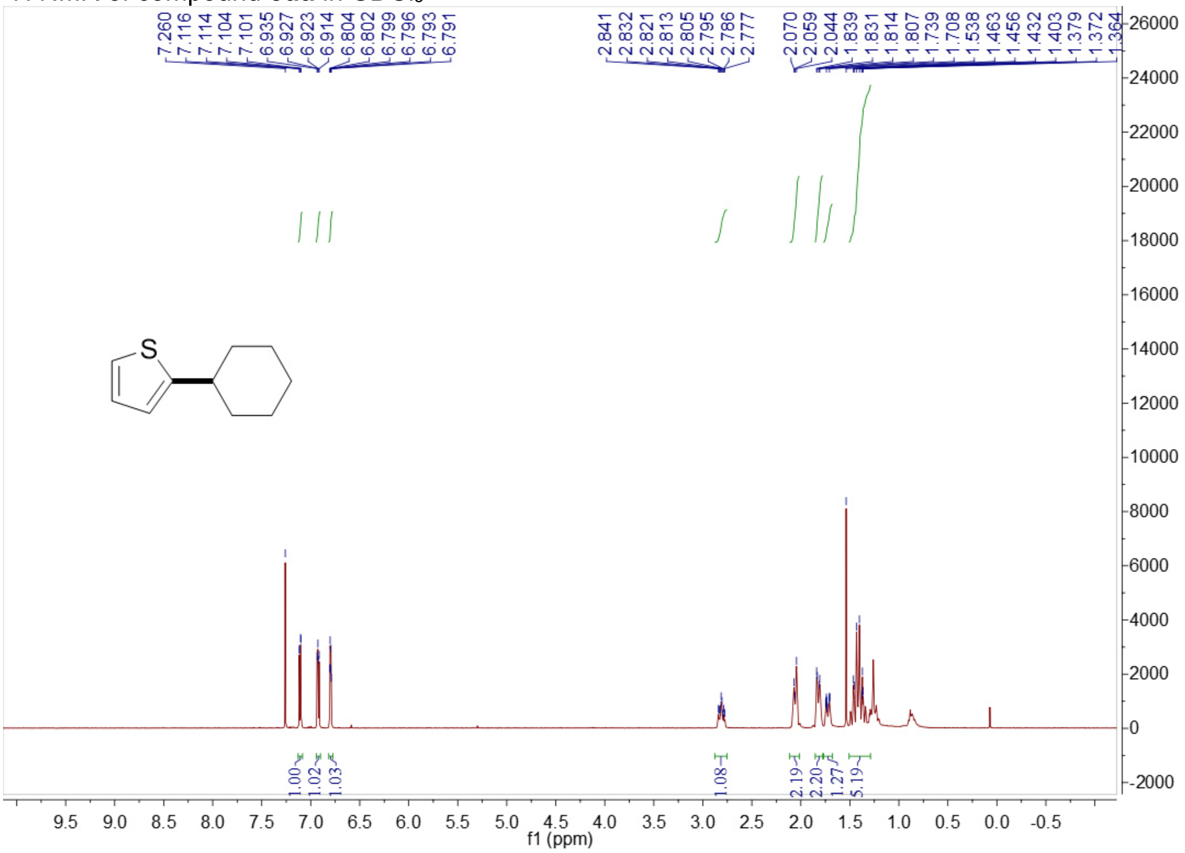

^{13}C NMR of compound **3ua** in $CDCl_3$

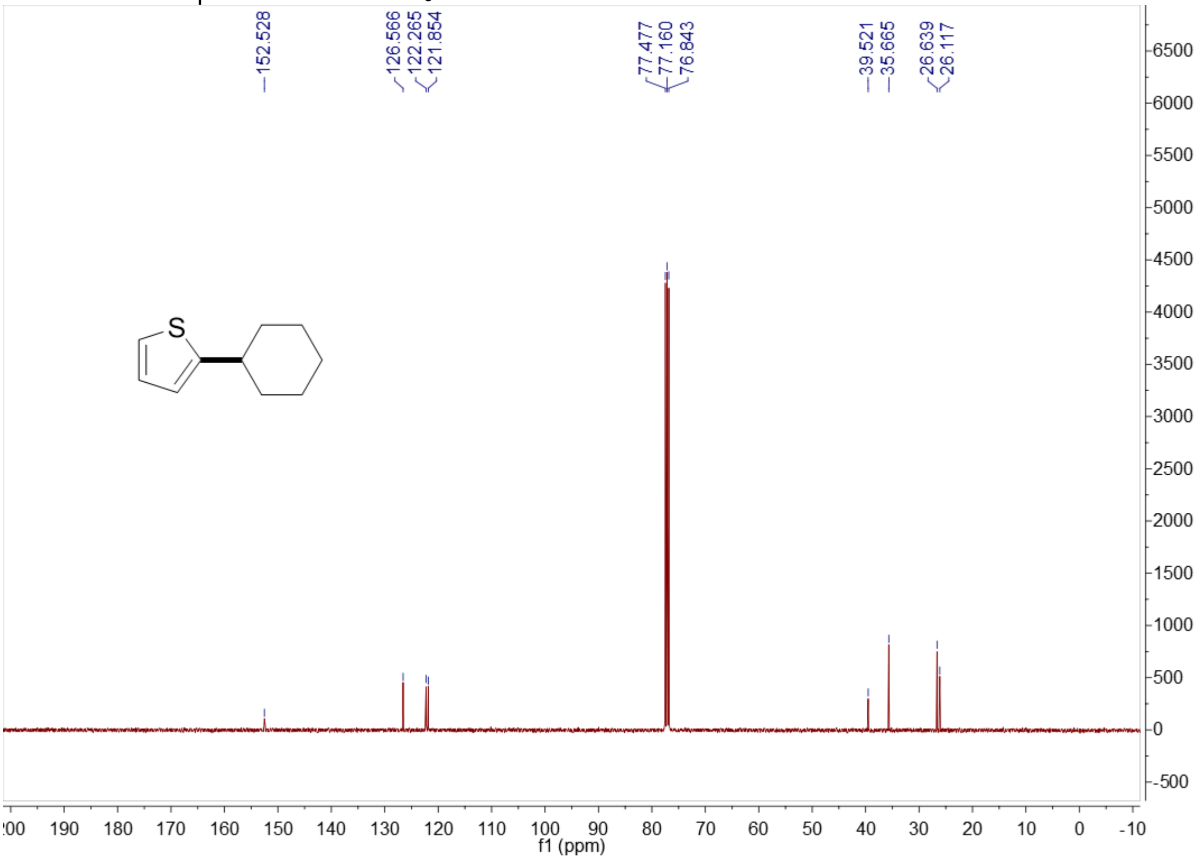

^{1}H NMR of compound **3va** in $CDCl_3$

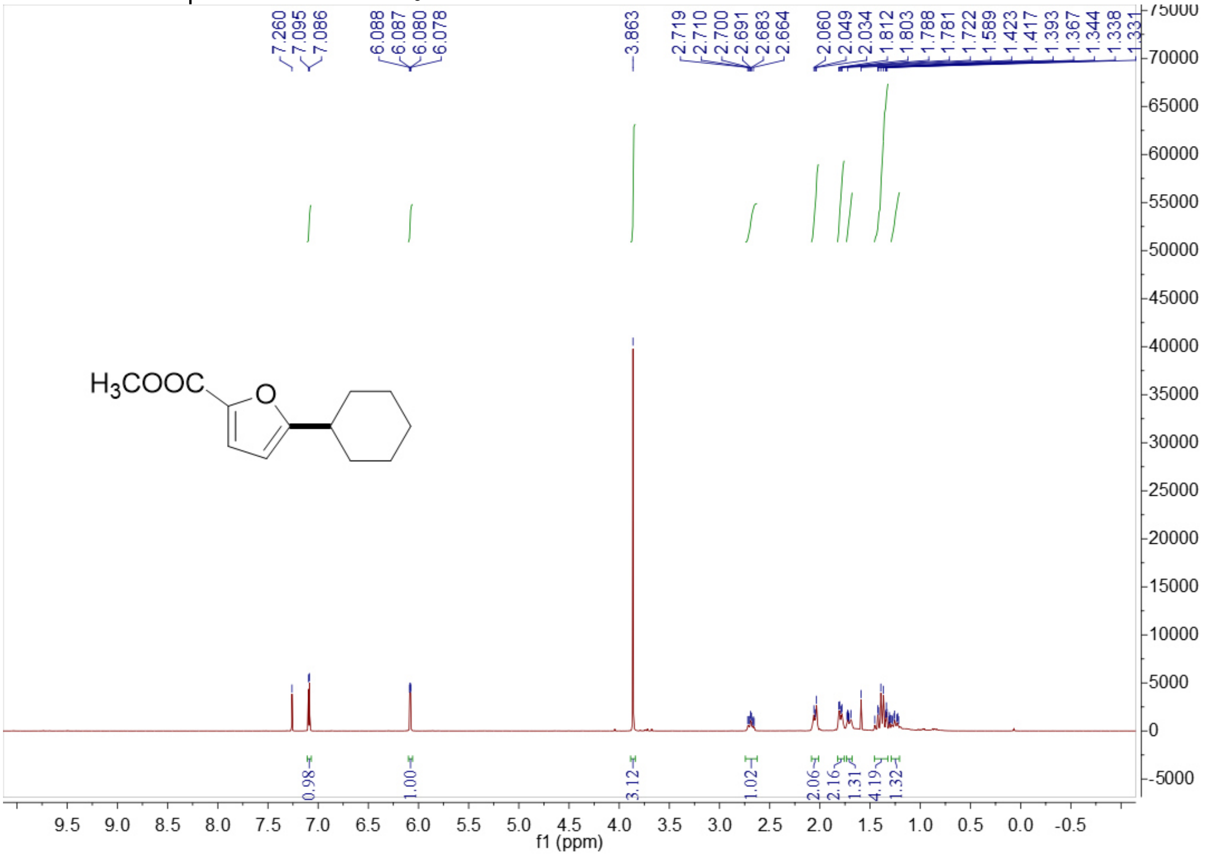

^{13}C NMR of compound **3va** in $CDCl_3$

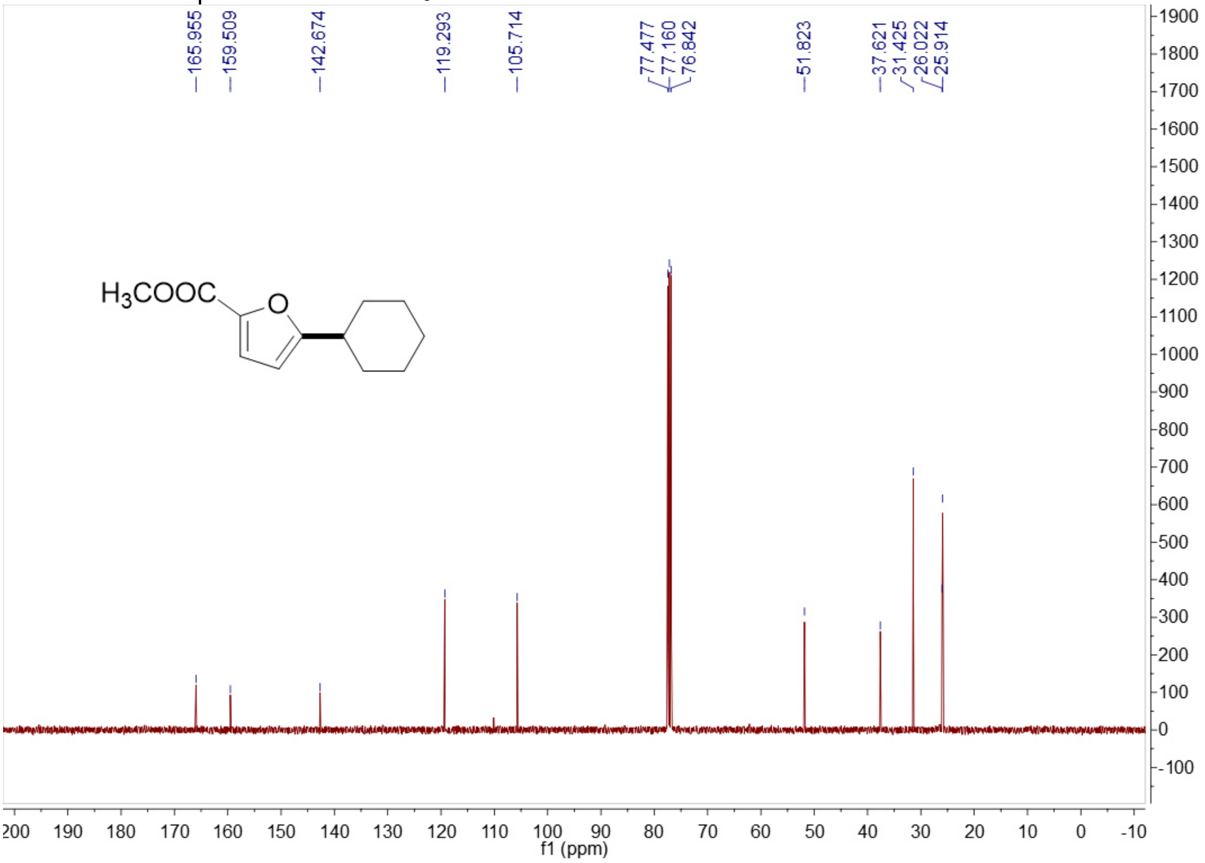

^{1}H NMR of compound **3wa** in $CDCl_3$

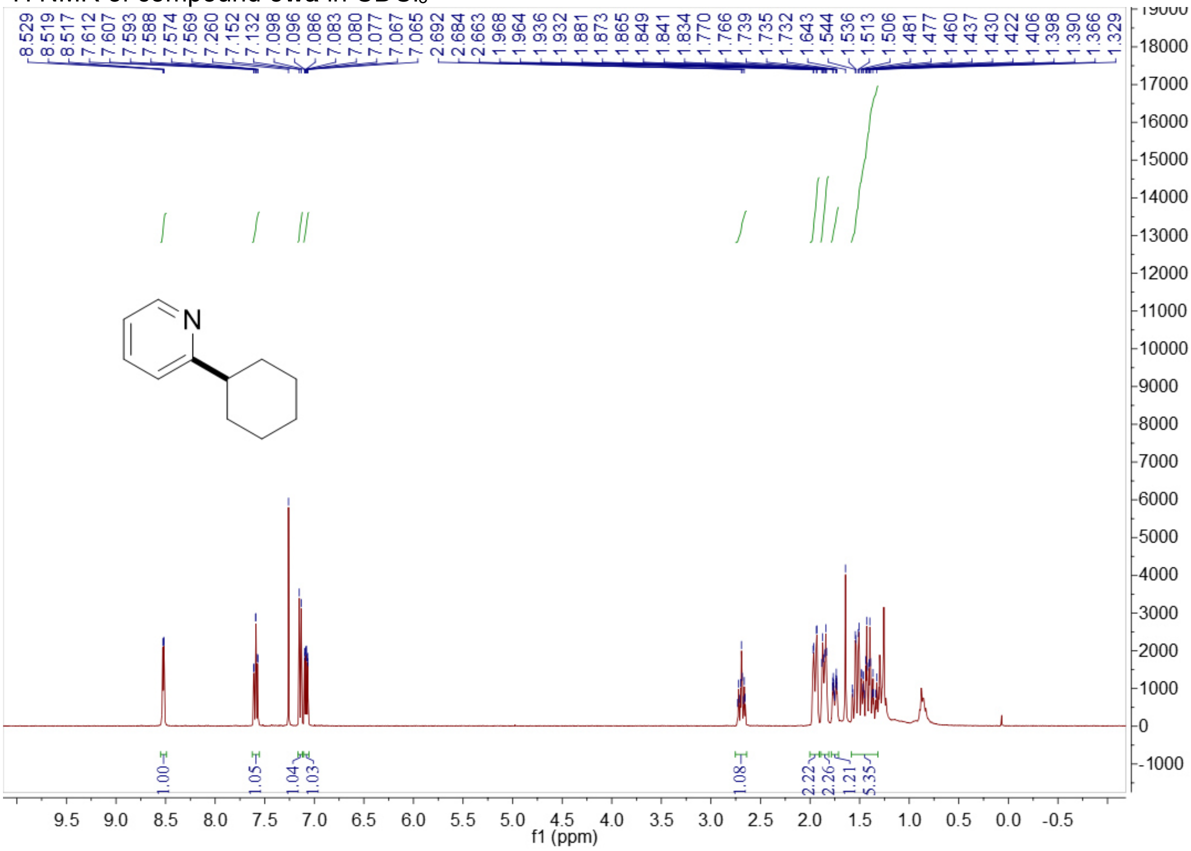

^{13}C NMR of compound **3wa** in $CDCl_3$

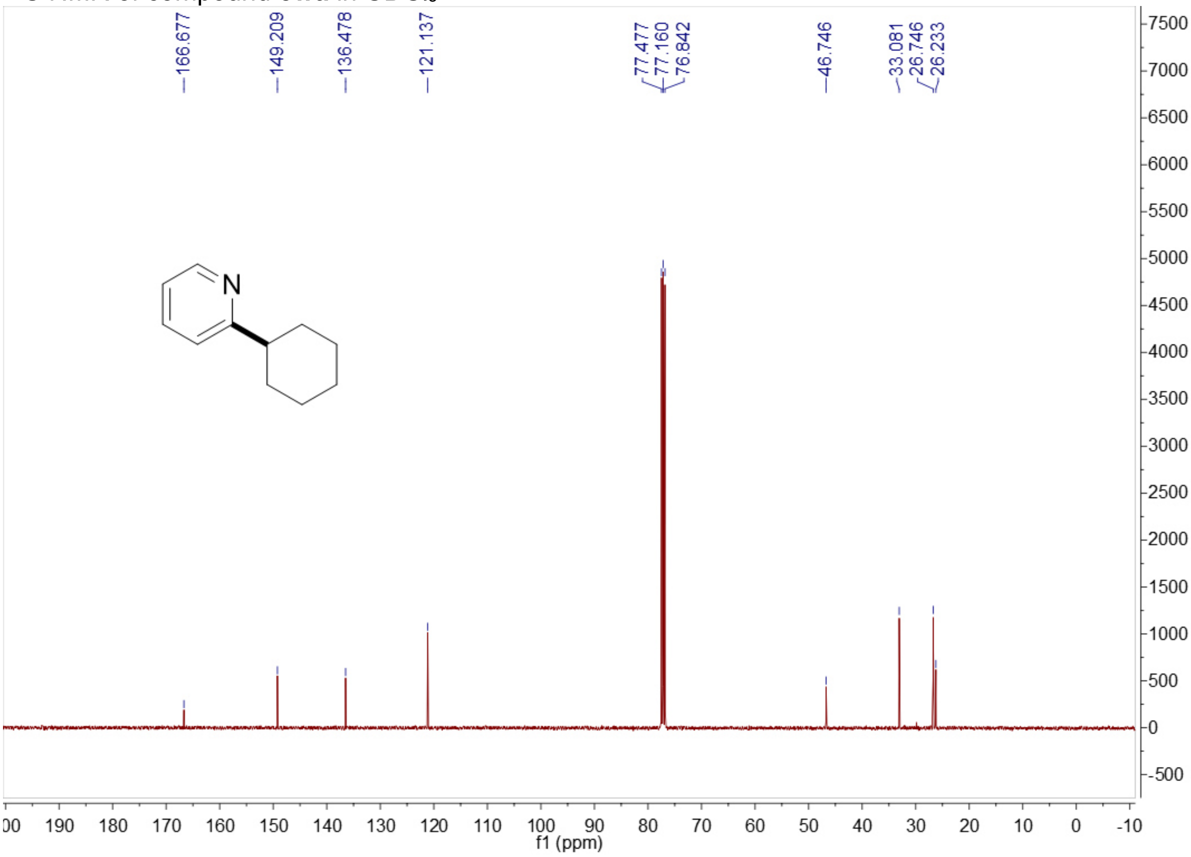

^{1}H NMR of compound **3xa** in $CDCl_3$

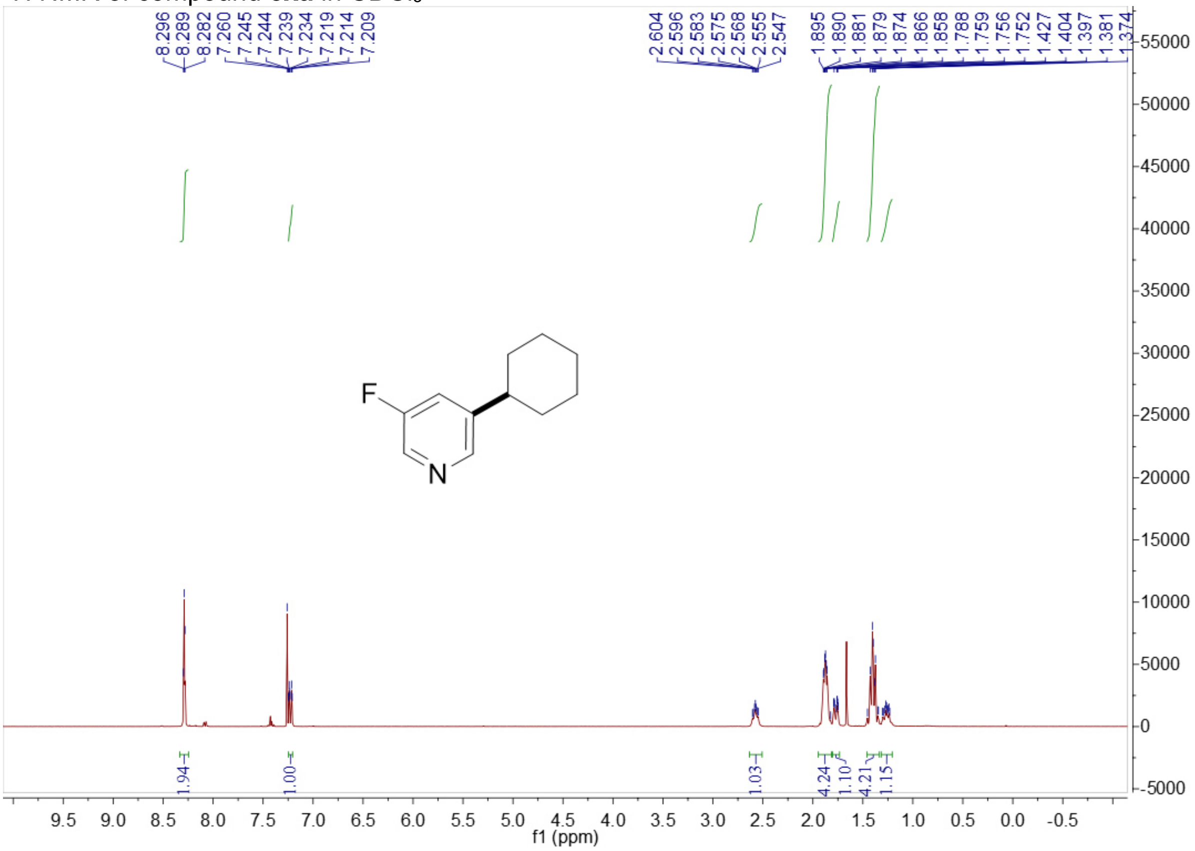

^{13}C NMR of compound **3xa** in $CDCl_3$

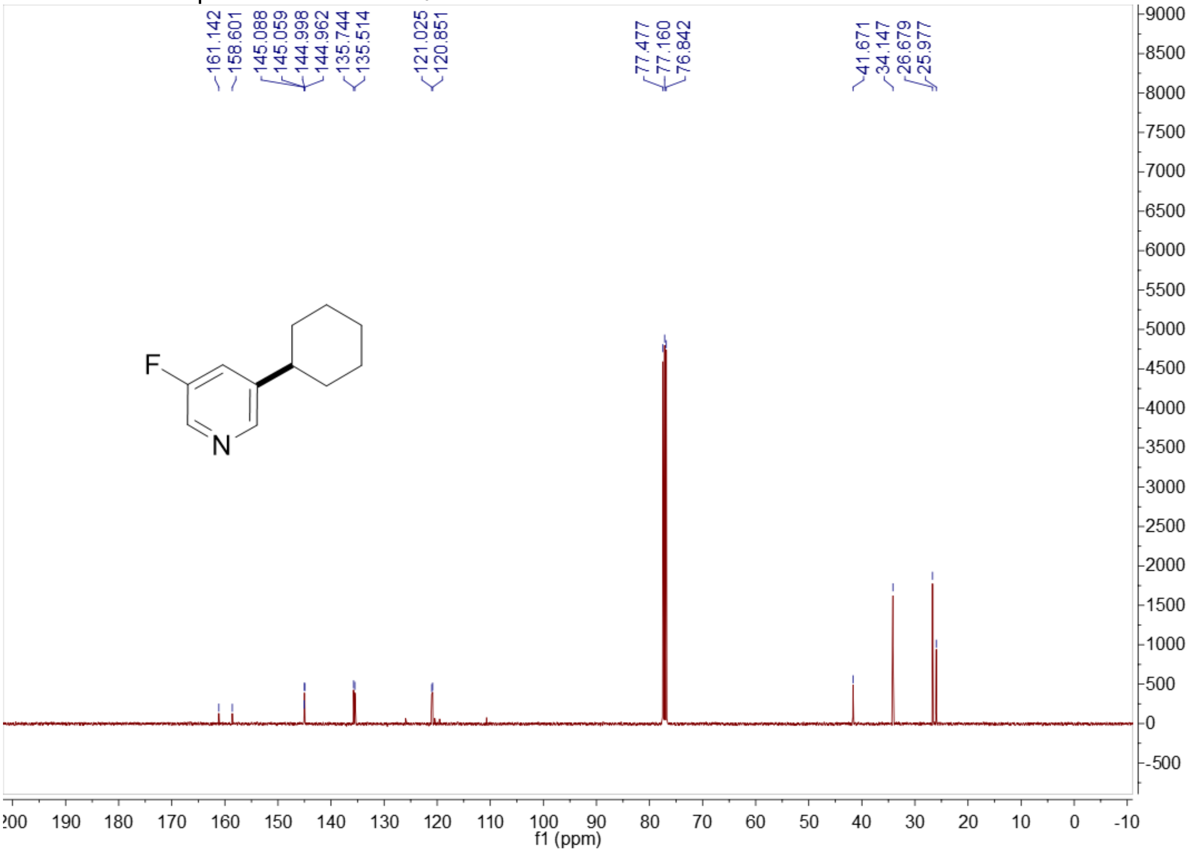

^{1}H NMR of compound **3ya** in $CDCl_3$

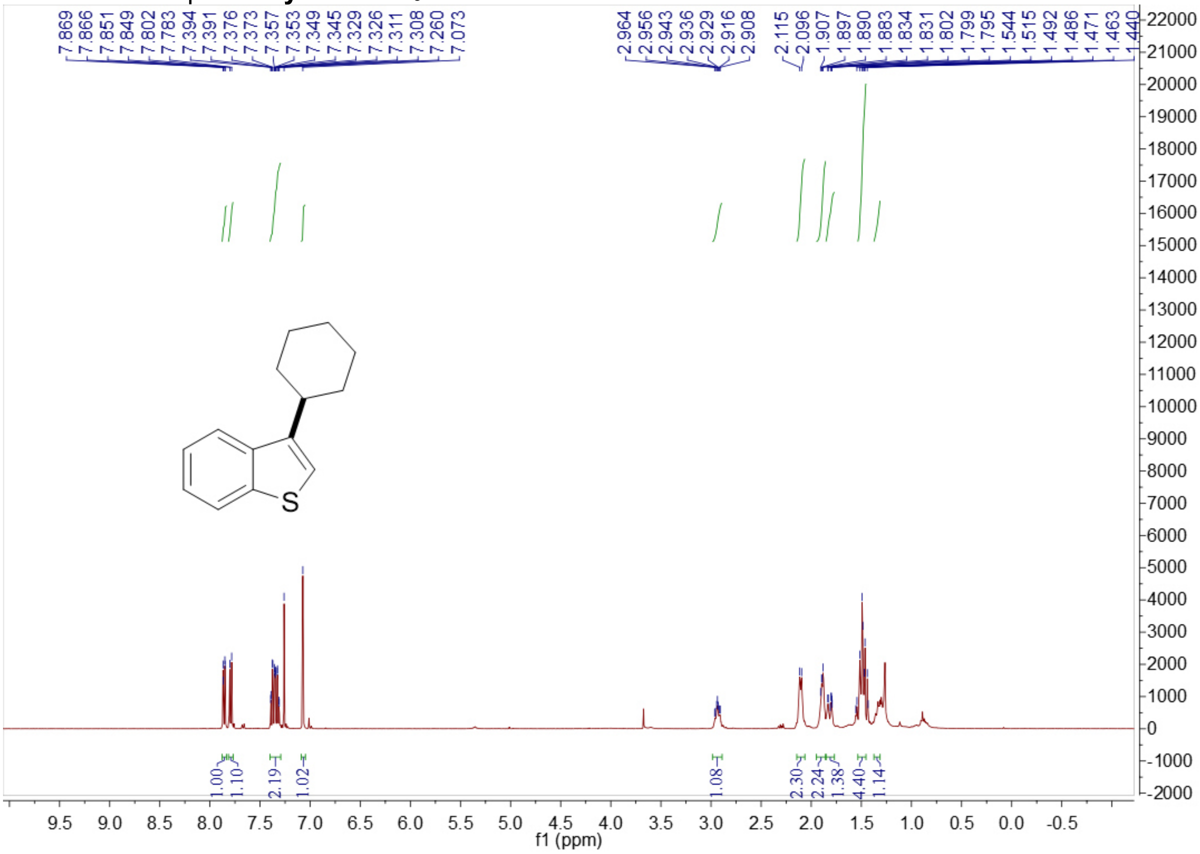

^{13}C NMR of compound **3ya** in $CDCl_3$

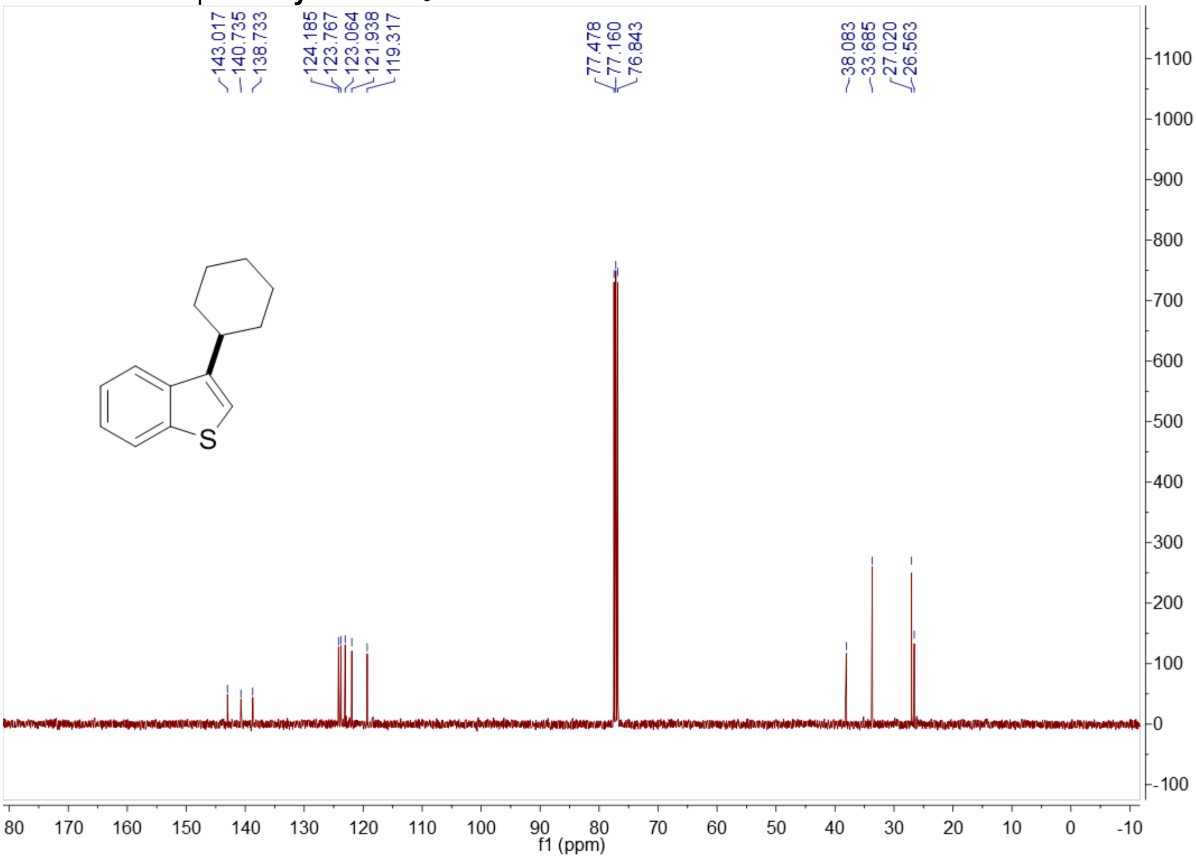

¹H NMR of compound **3za** in $CDCl_3$

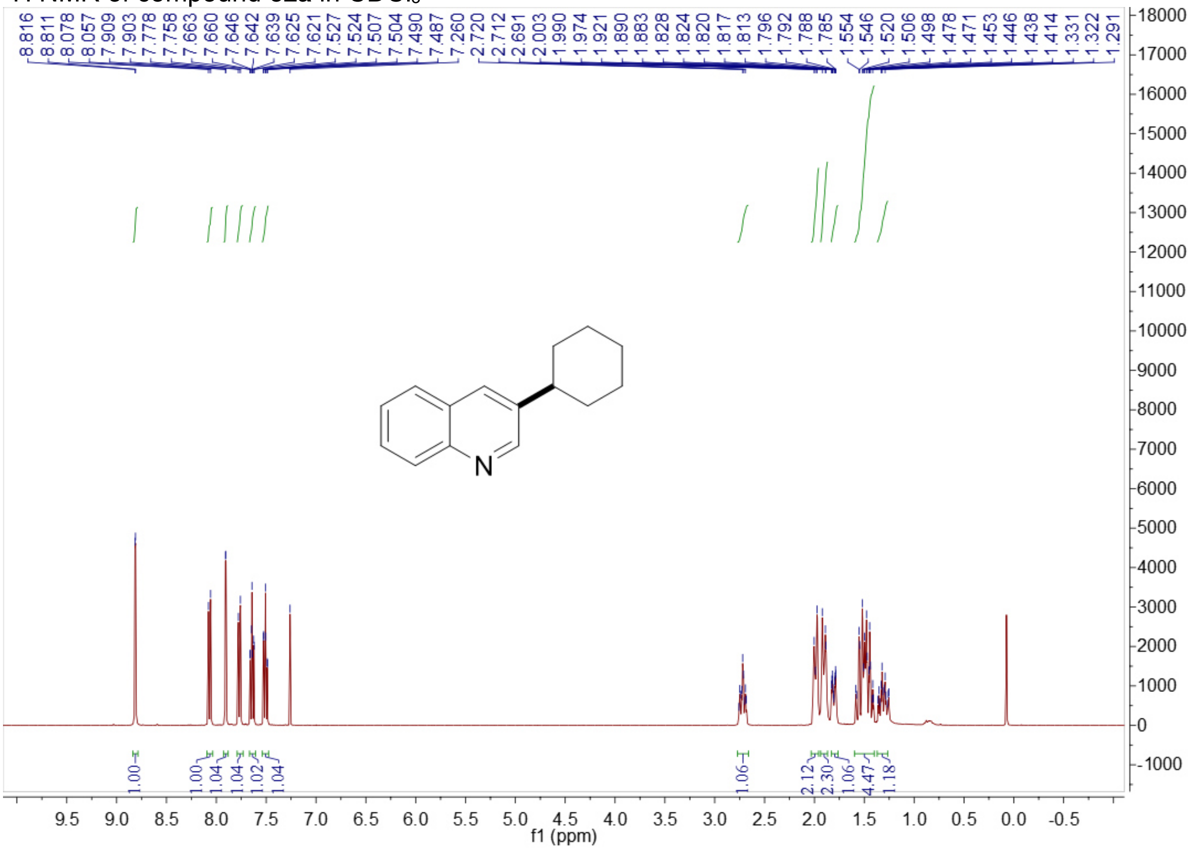

¹³C NMR of compound **3za** in $CDCl_3$

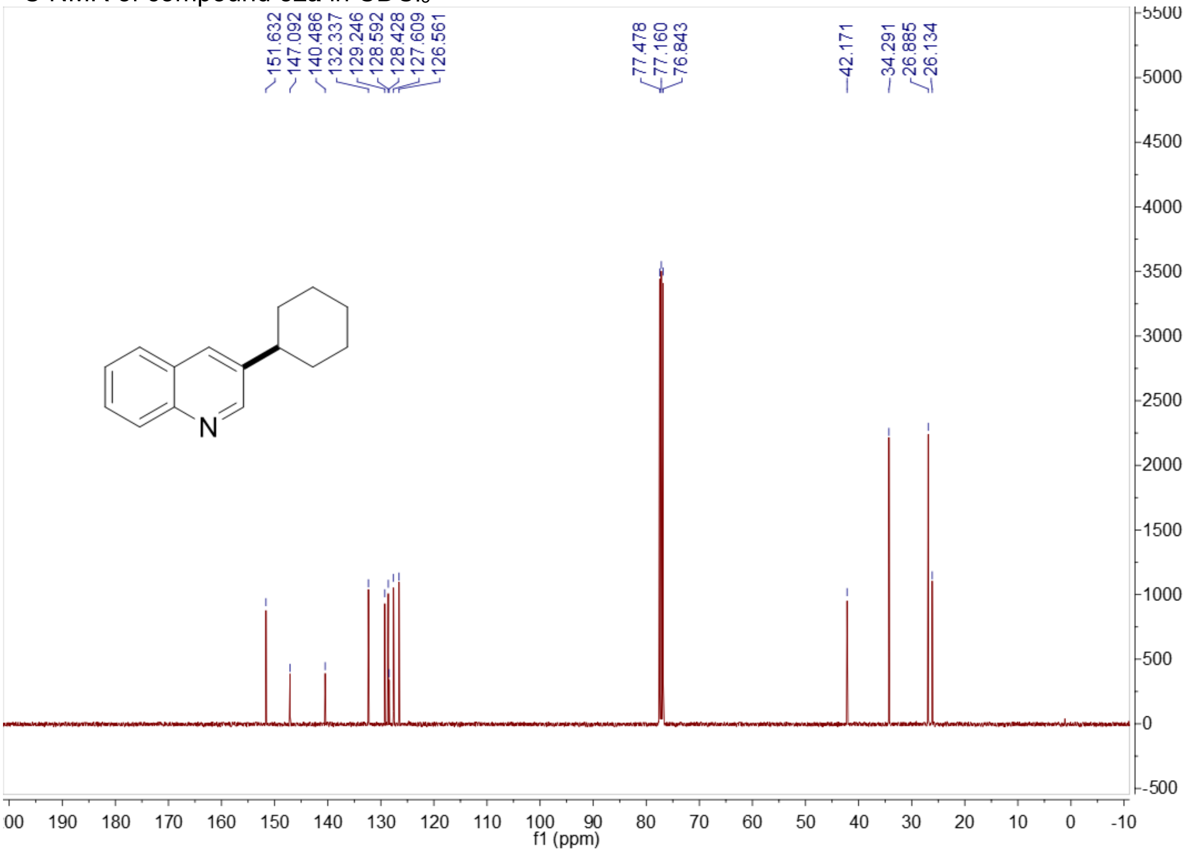

^{1}H NMR of compound **3aaa** in $CDCl_3$

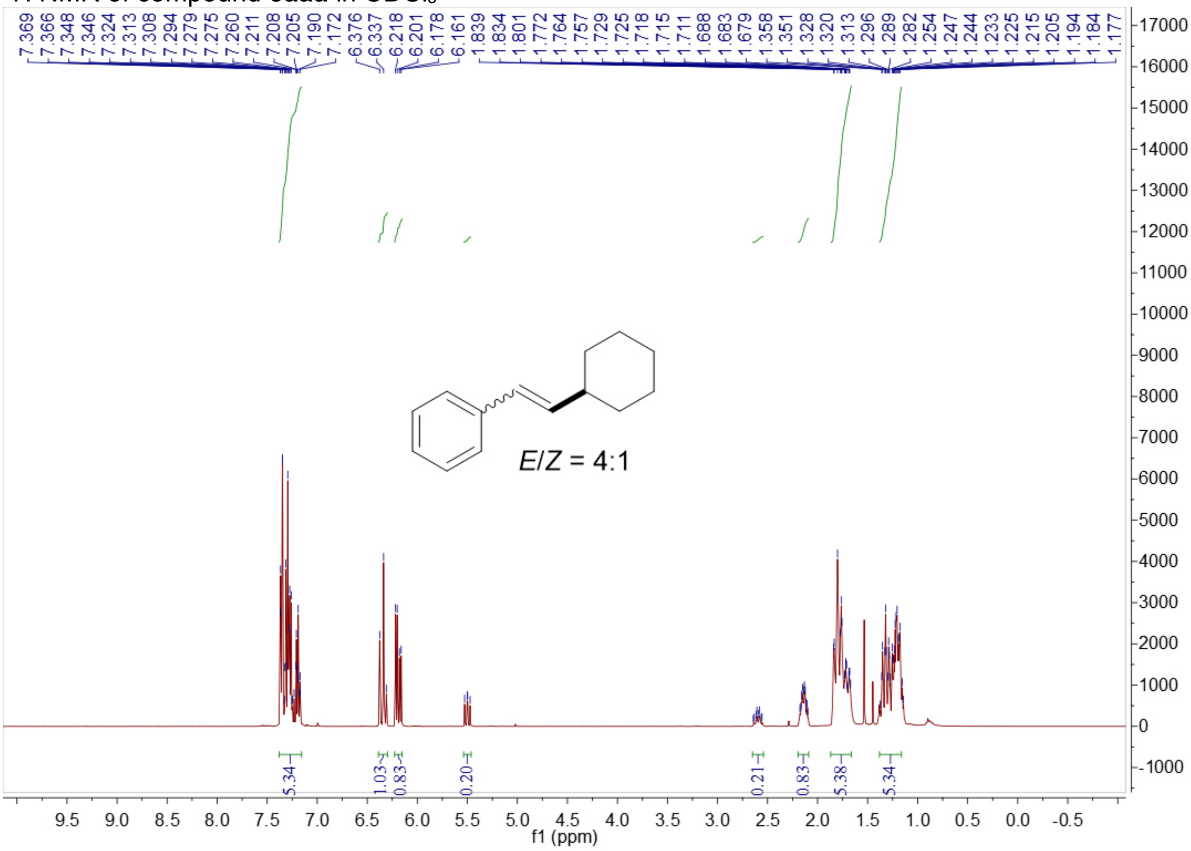

^{13}C NMR of compound **3aaa** in $CDCl_3$

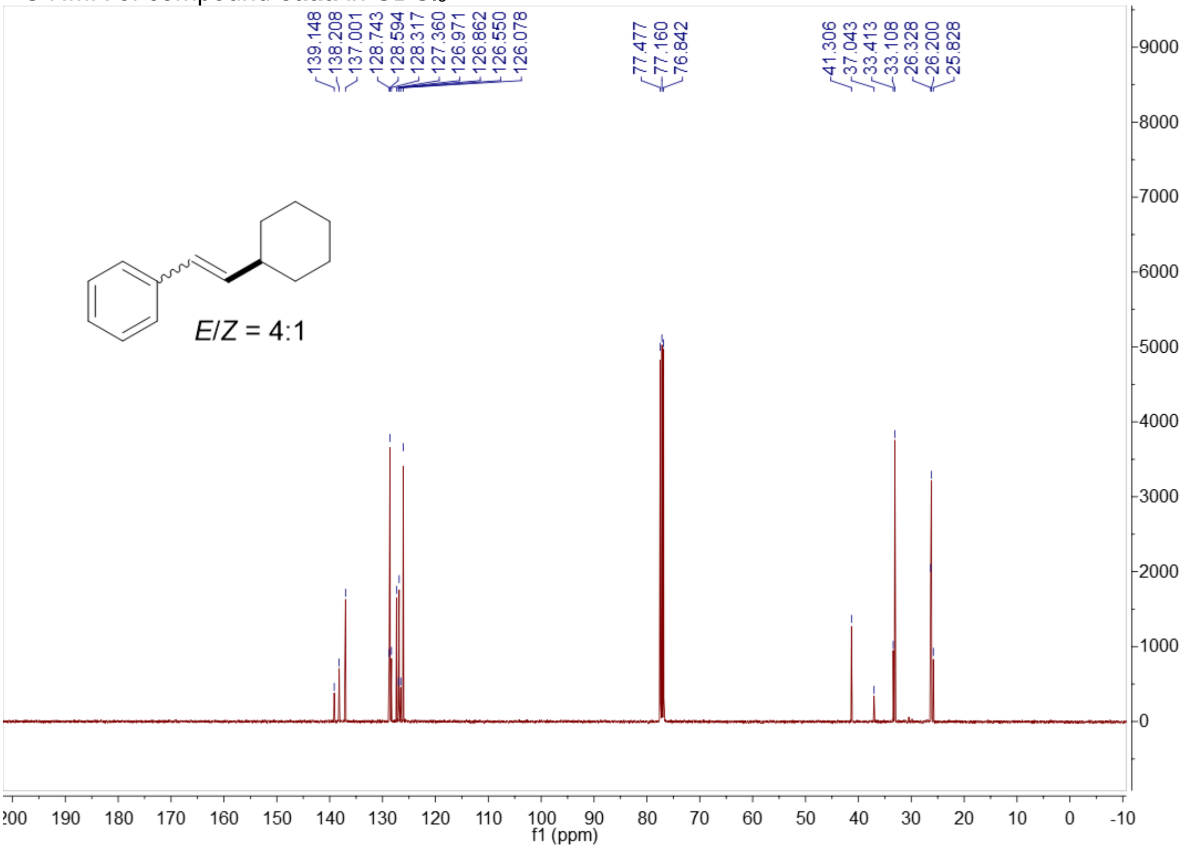

^1H NMR of compound **3aba** in $CDCl_3$

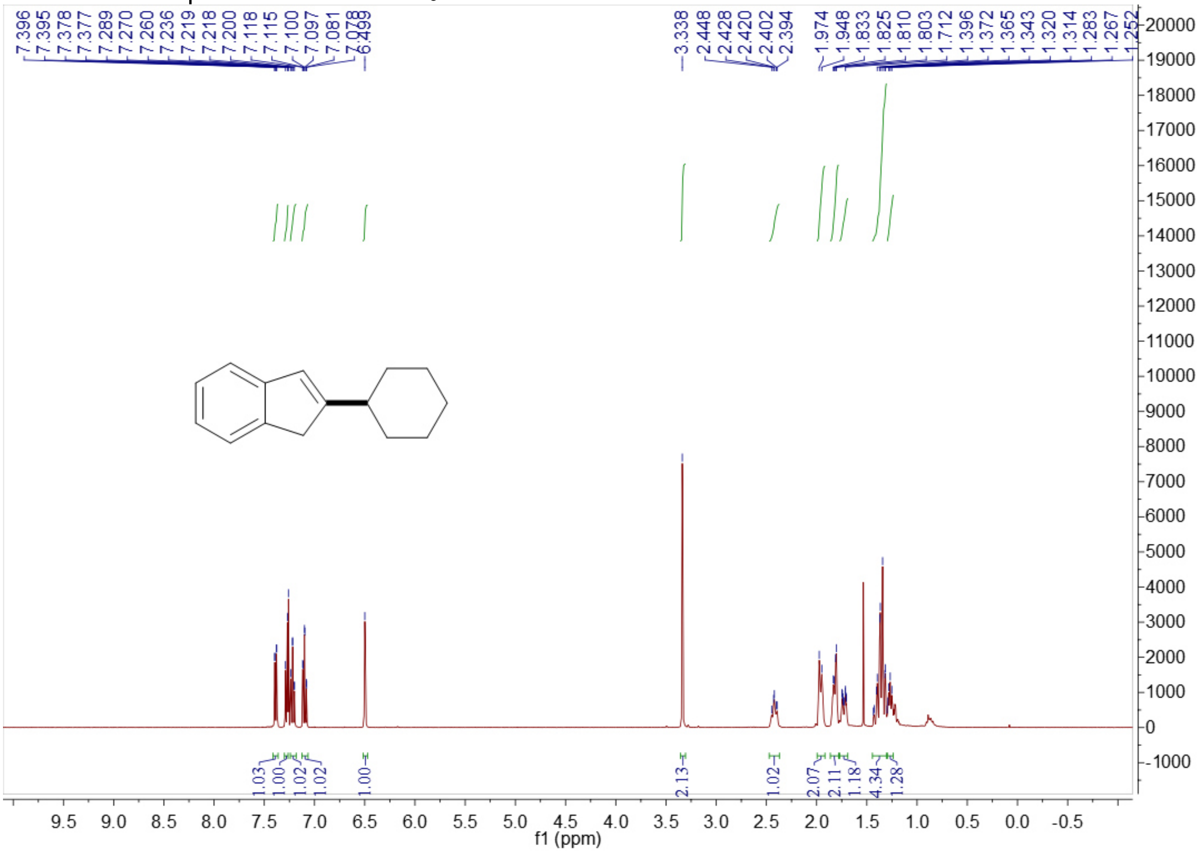

^{13}C NMR of compound **3aba** in $CDCl_3$

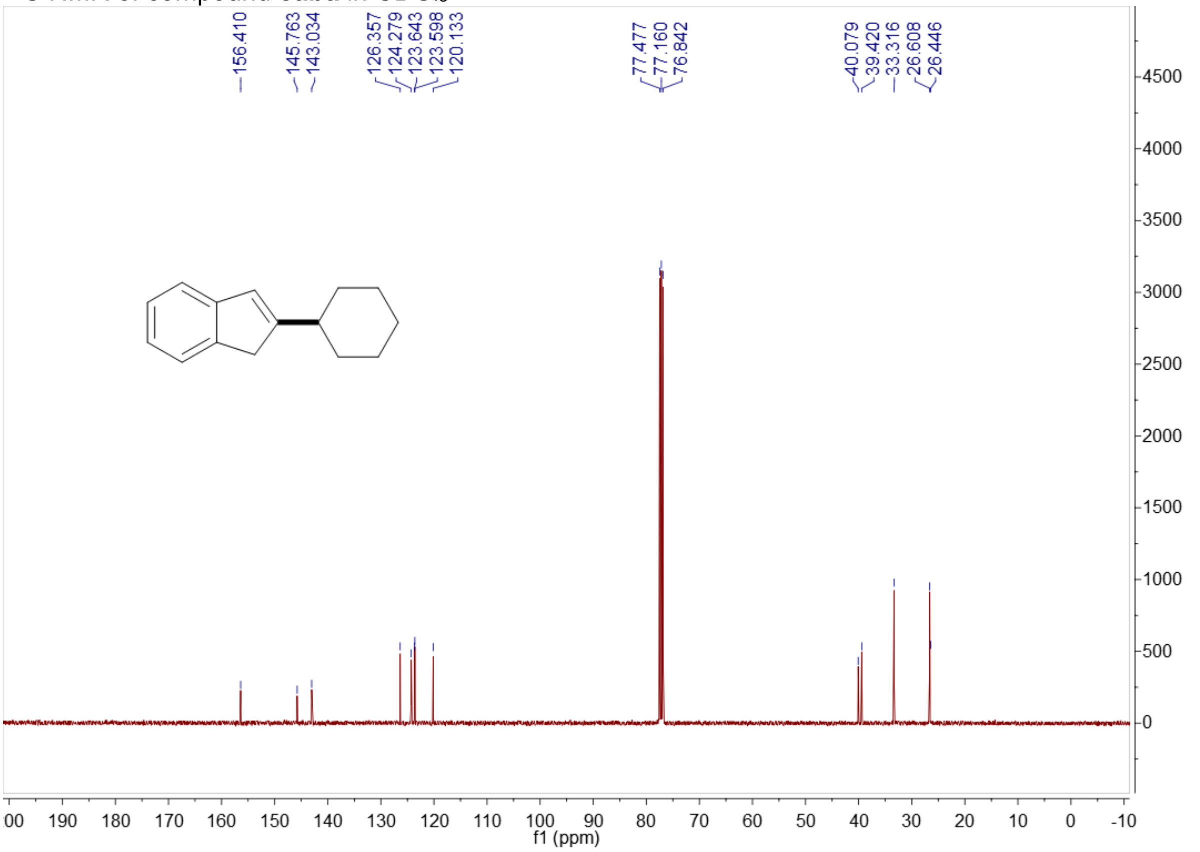

^{1}H NMR of compound **3aca** in $CDCl_3$

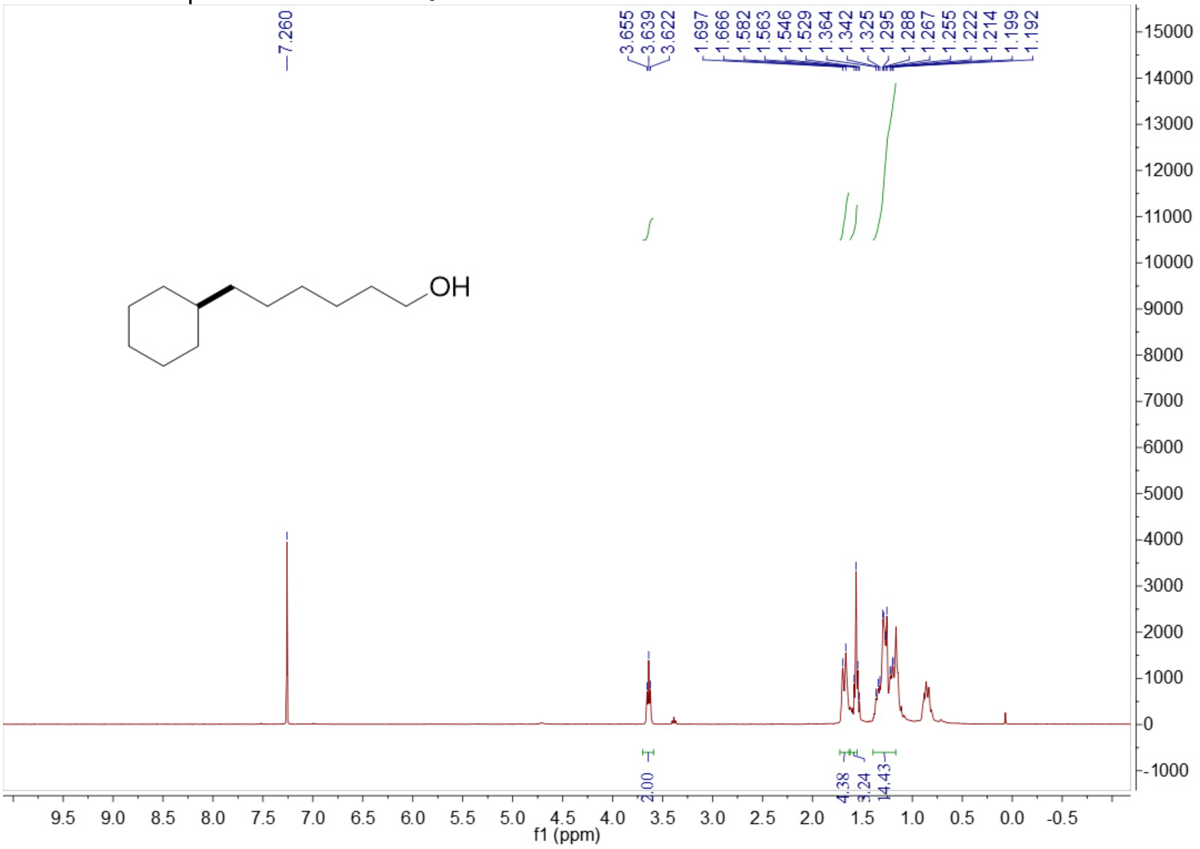

^{13}C NMR of compound **3aca** in $CDCl_3$

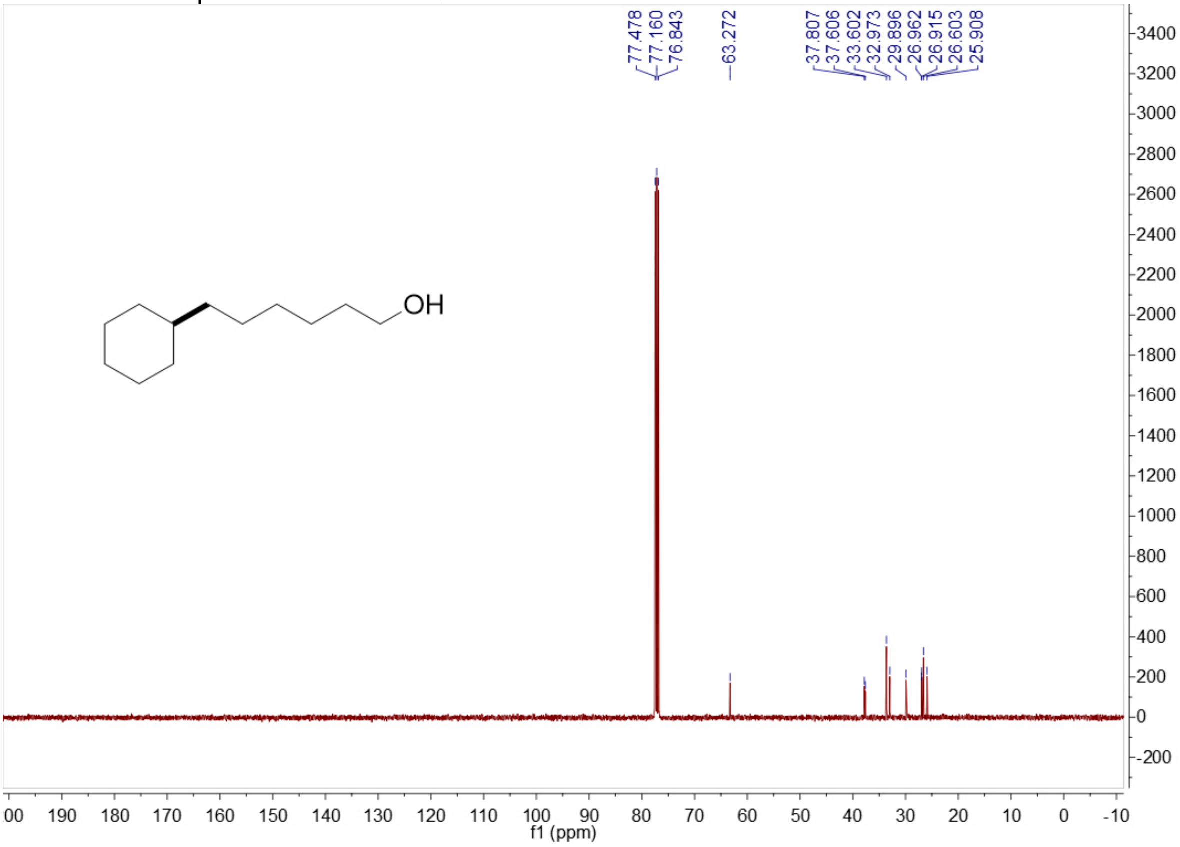

^{1}H NMR of compound **3ada** in $CDCl_3$

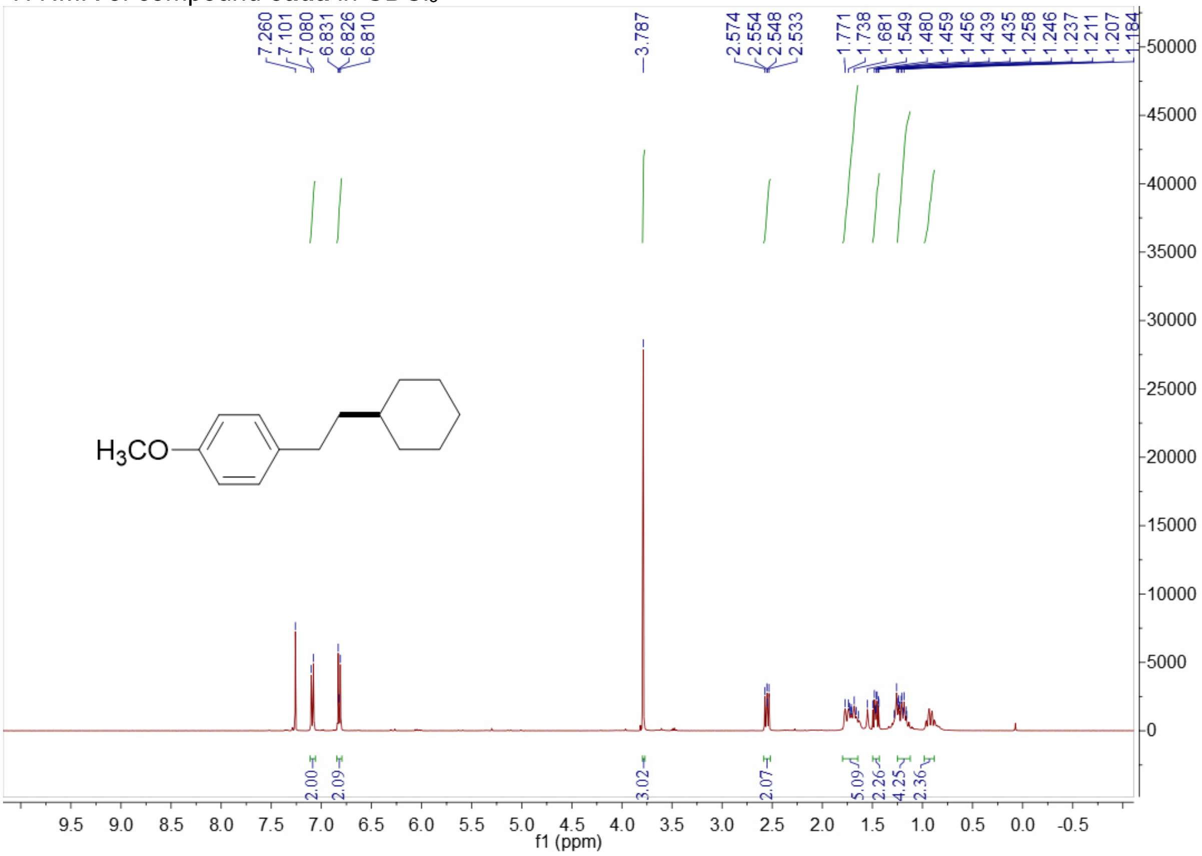

^{13}C NMR of compound **3ada** in $CDCl_3$

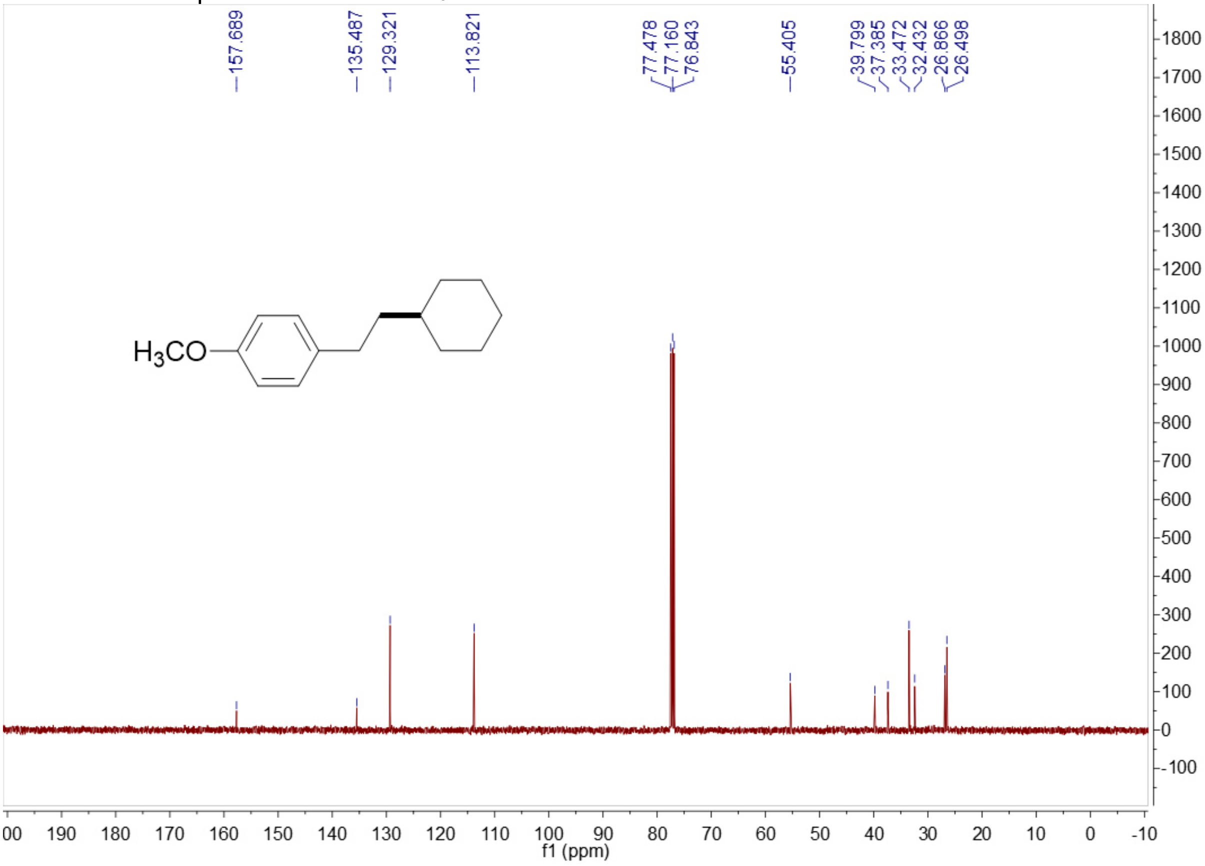

^{1}H NMR of compound **3lb** in $CDCl_3$

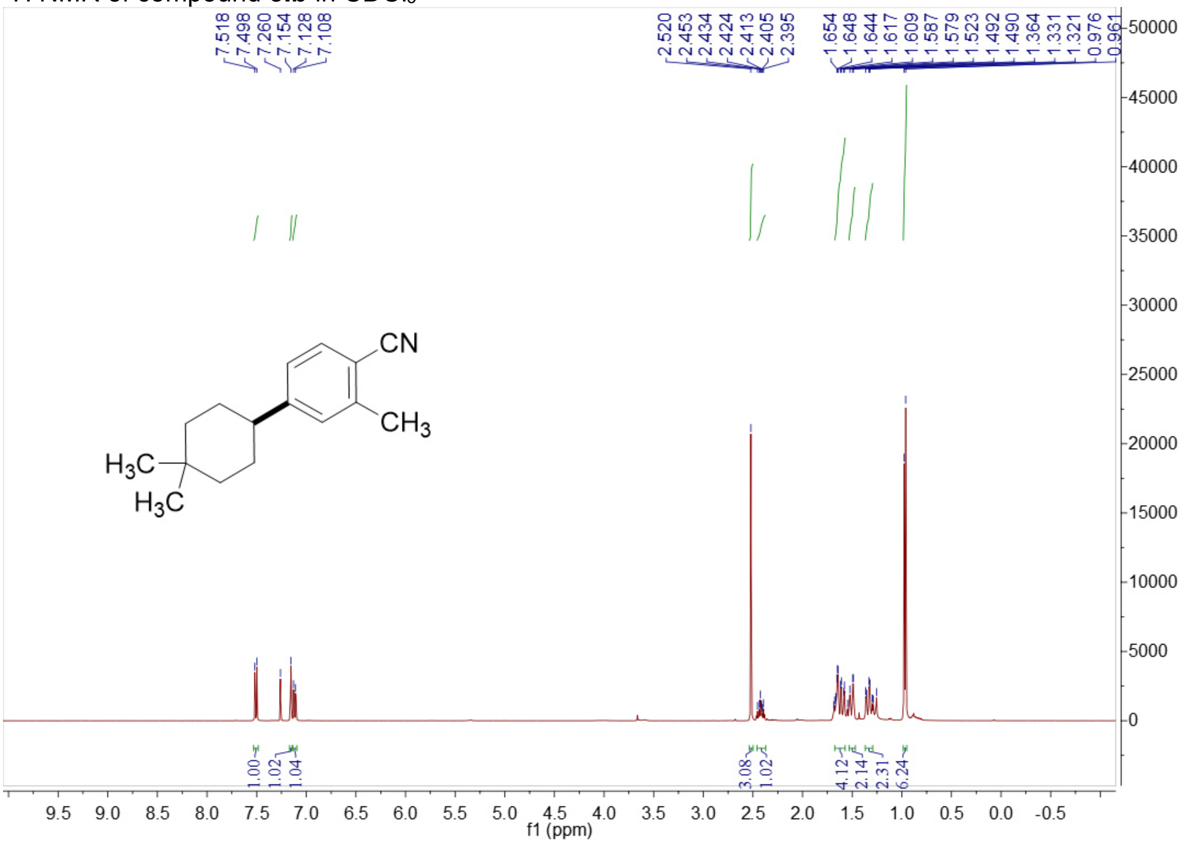

^{13}C NMR of compound **3lb** in $CDCl_3$

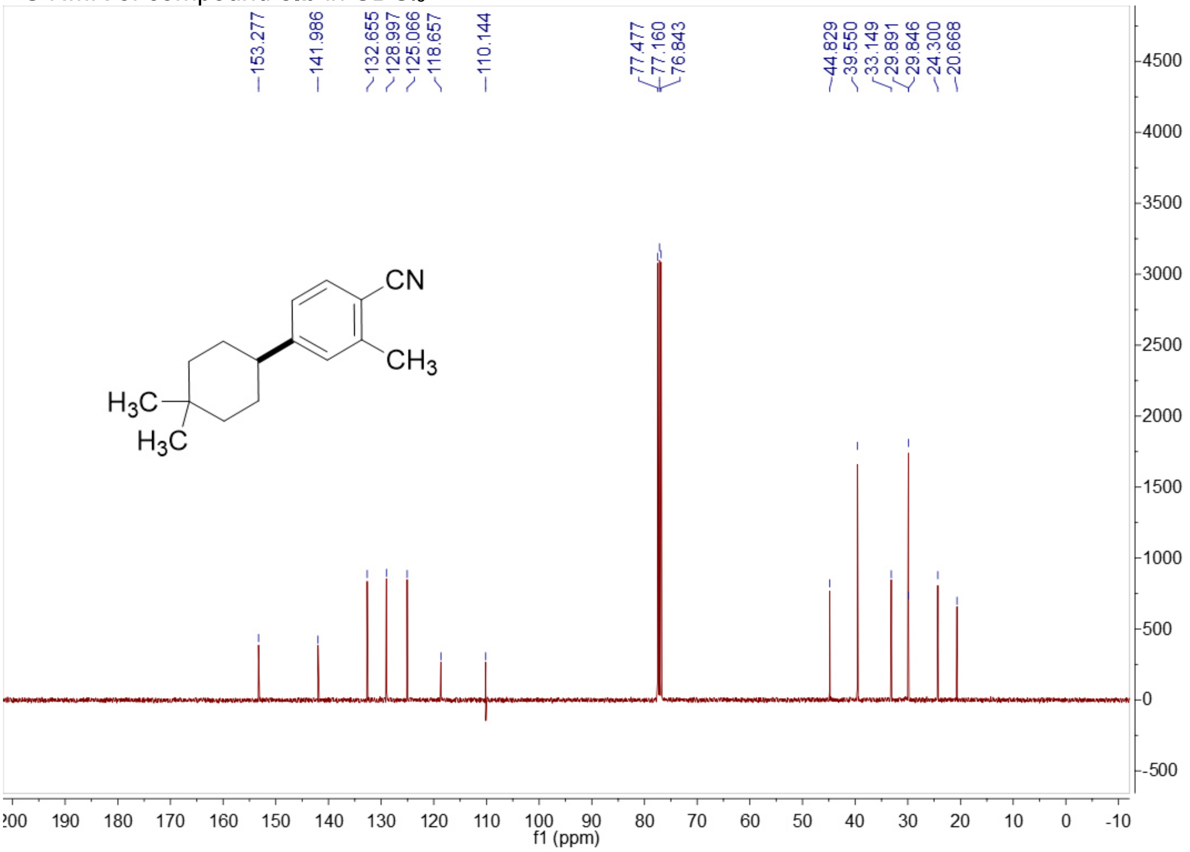

^{1}H NMR of compound **3lc** in $CDCl_3$

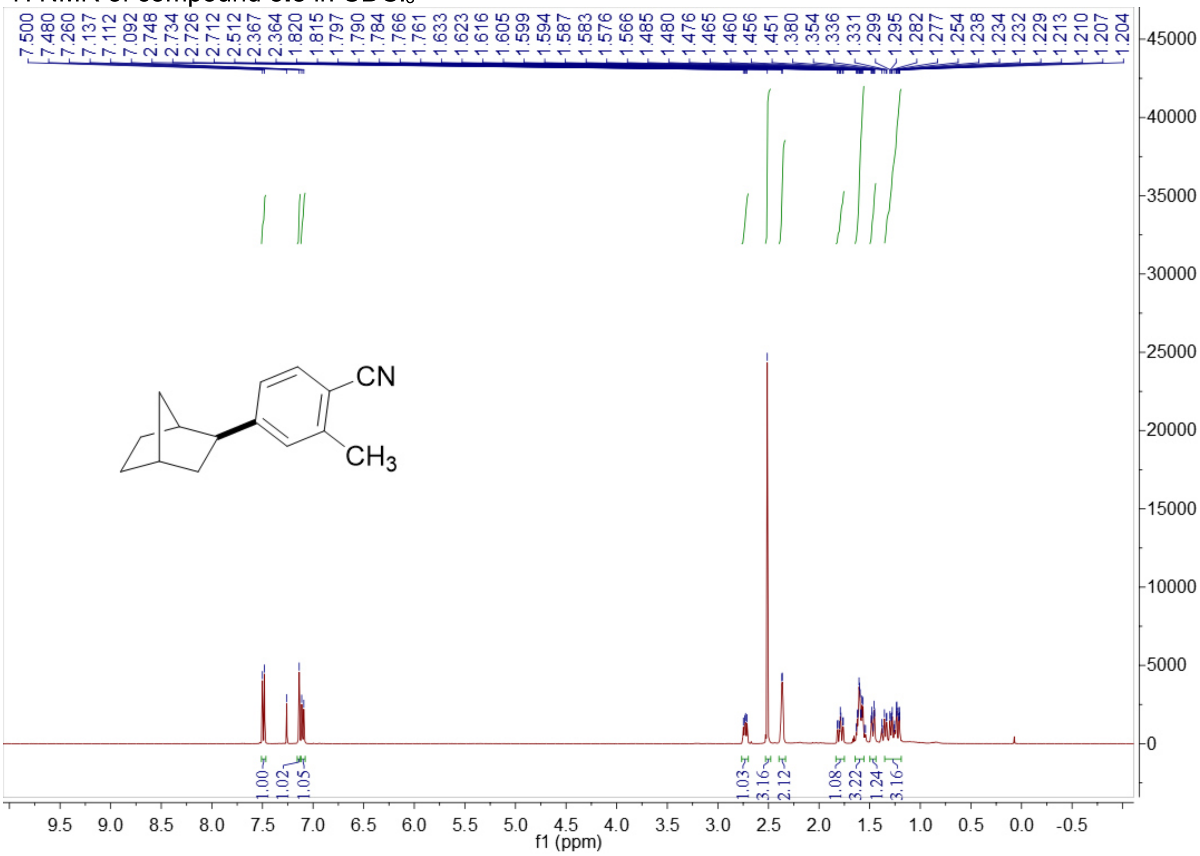

^{13}C NMR of compound **3lc** in $CDCl_3$

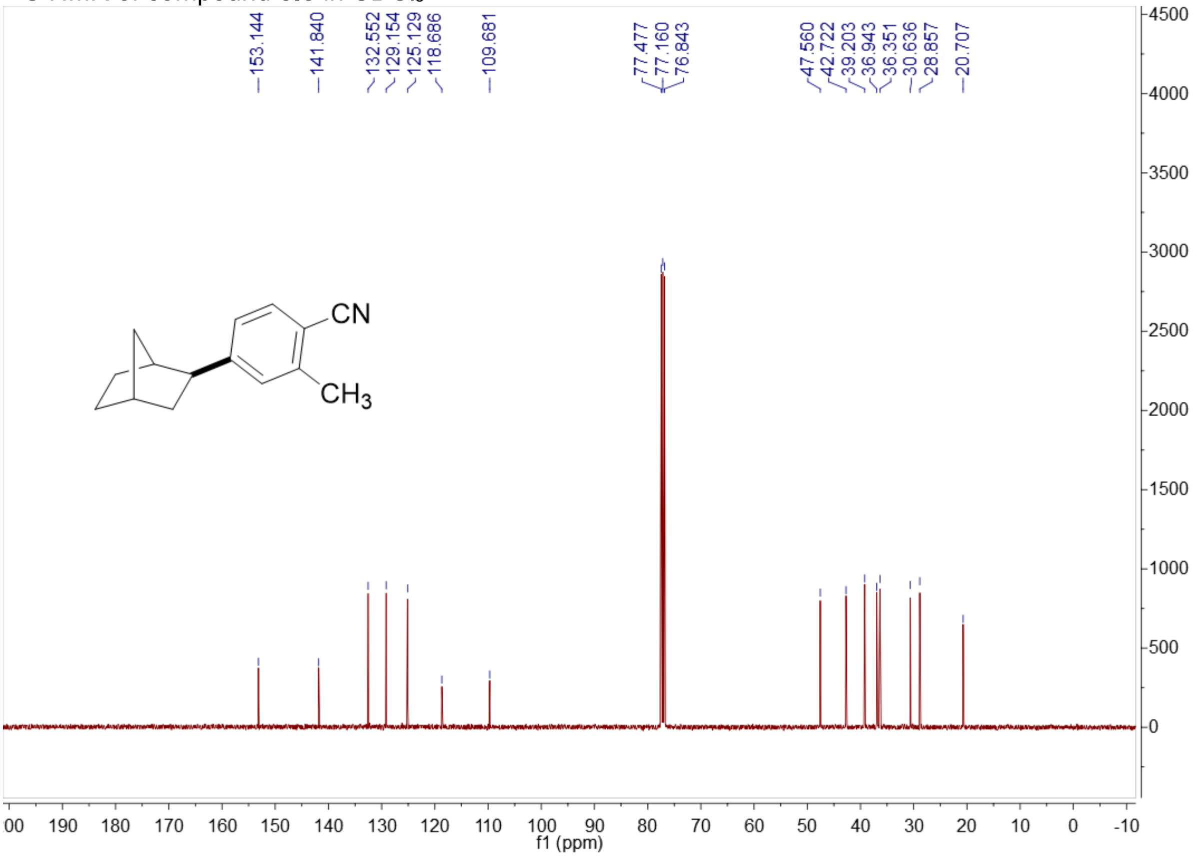

^{1}H NMR of compound **3ld** in $CDCl_3$

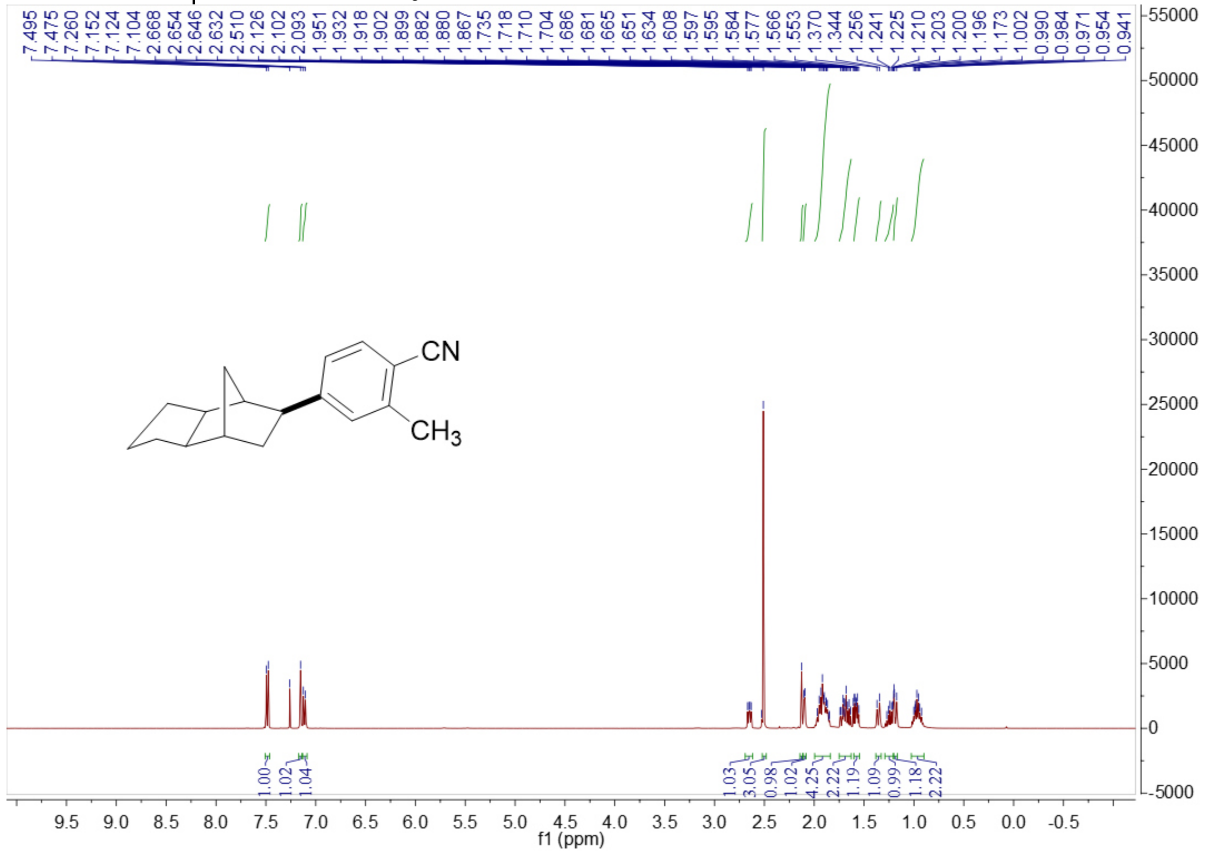

^{13}C NMR of compound **3ld** in $CDCl_3$

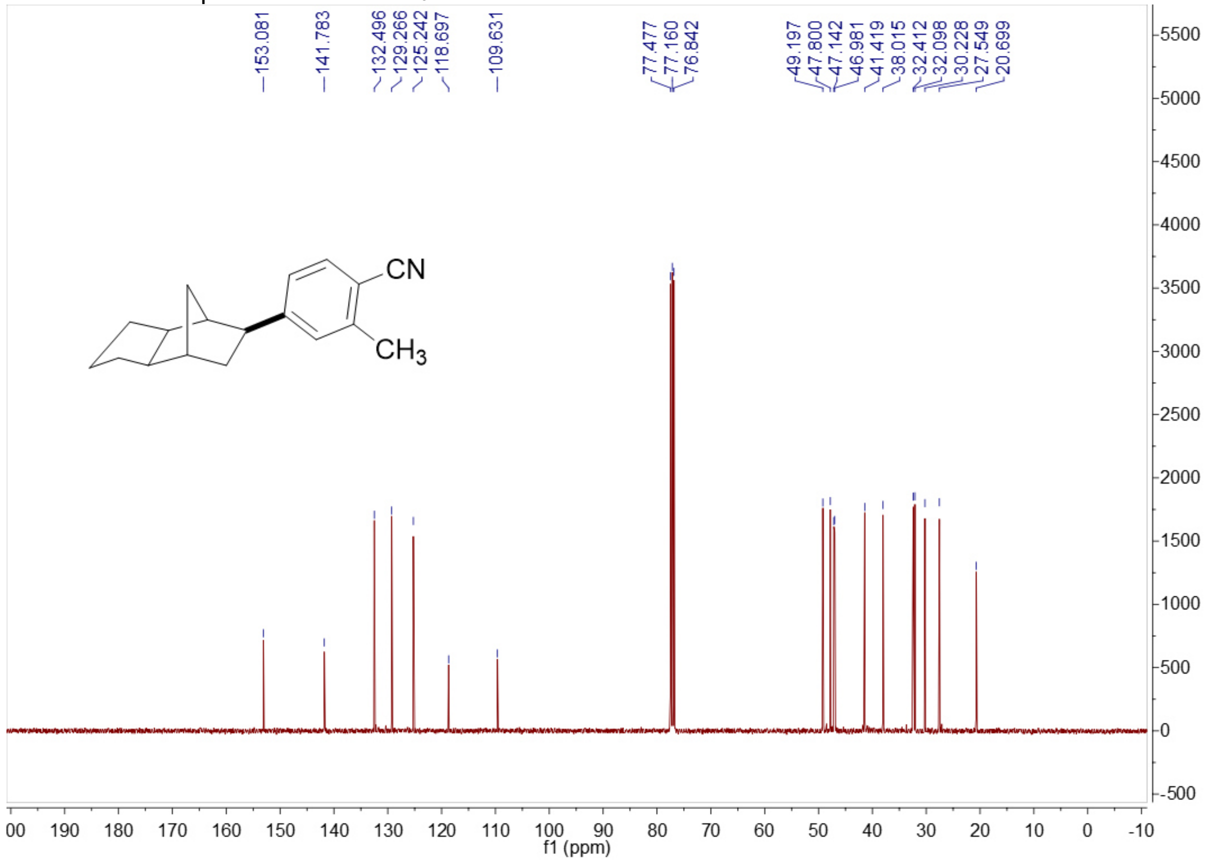

^{1}H NMR of compound **3le** in $CDCl_3$

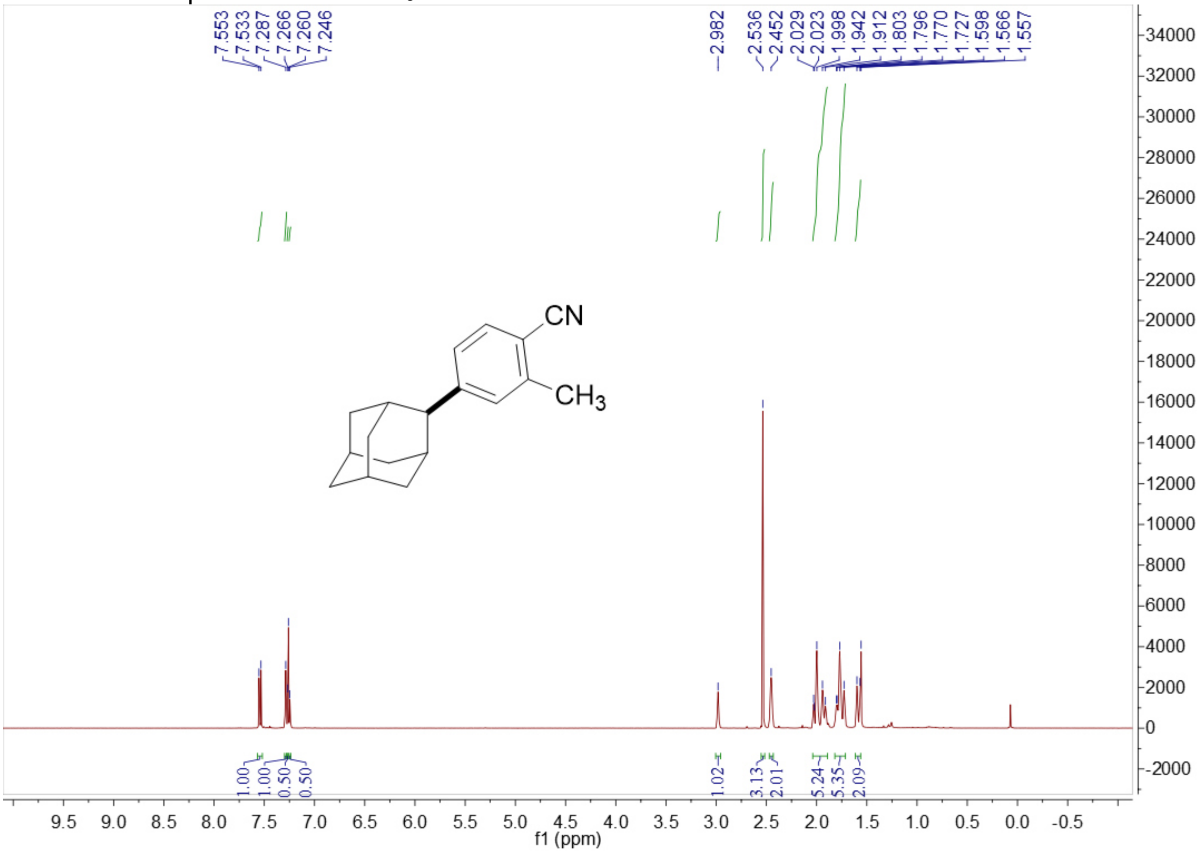

^{13}C NMR of compound **3le** in $CDCl_3$

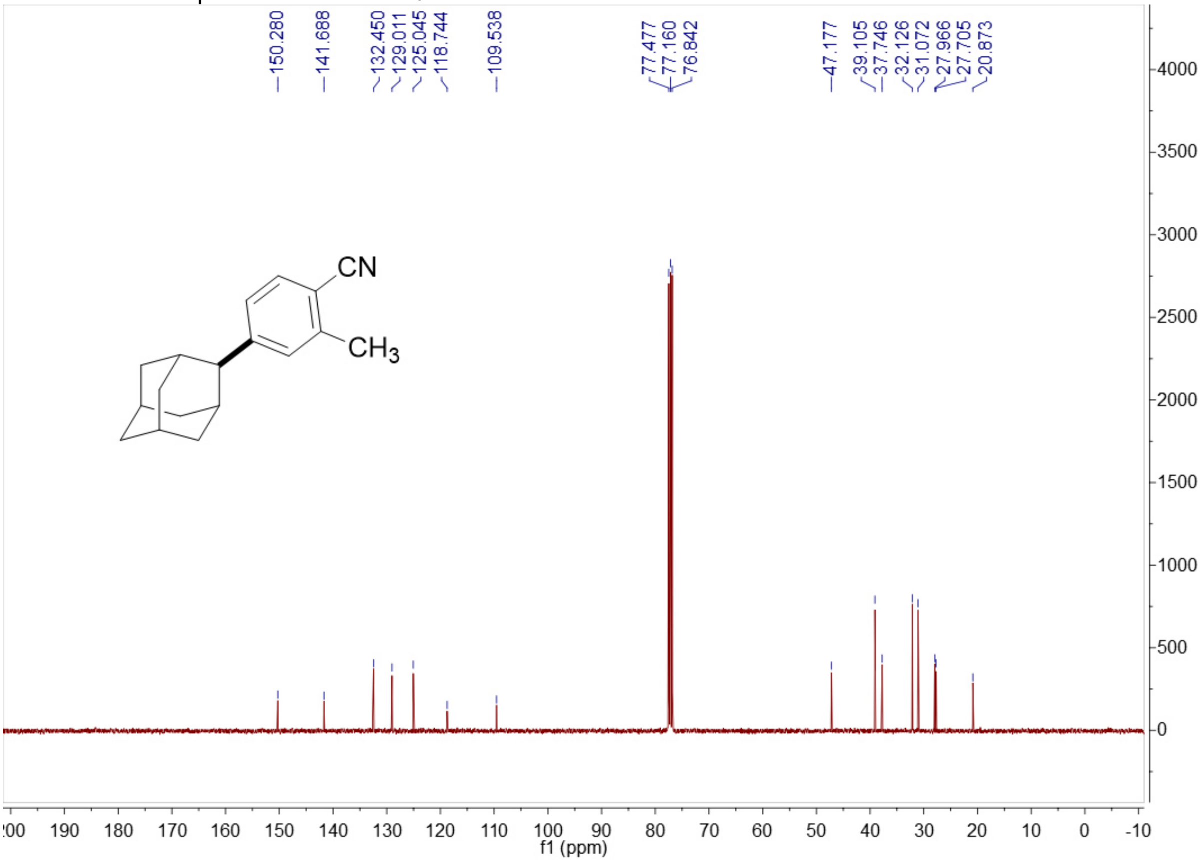

^{1}H NMR of compound **3aaf** in $CDCl_3$

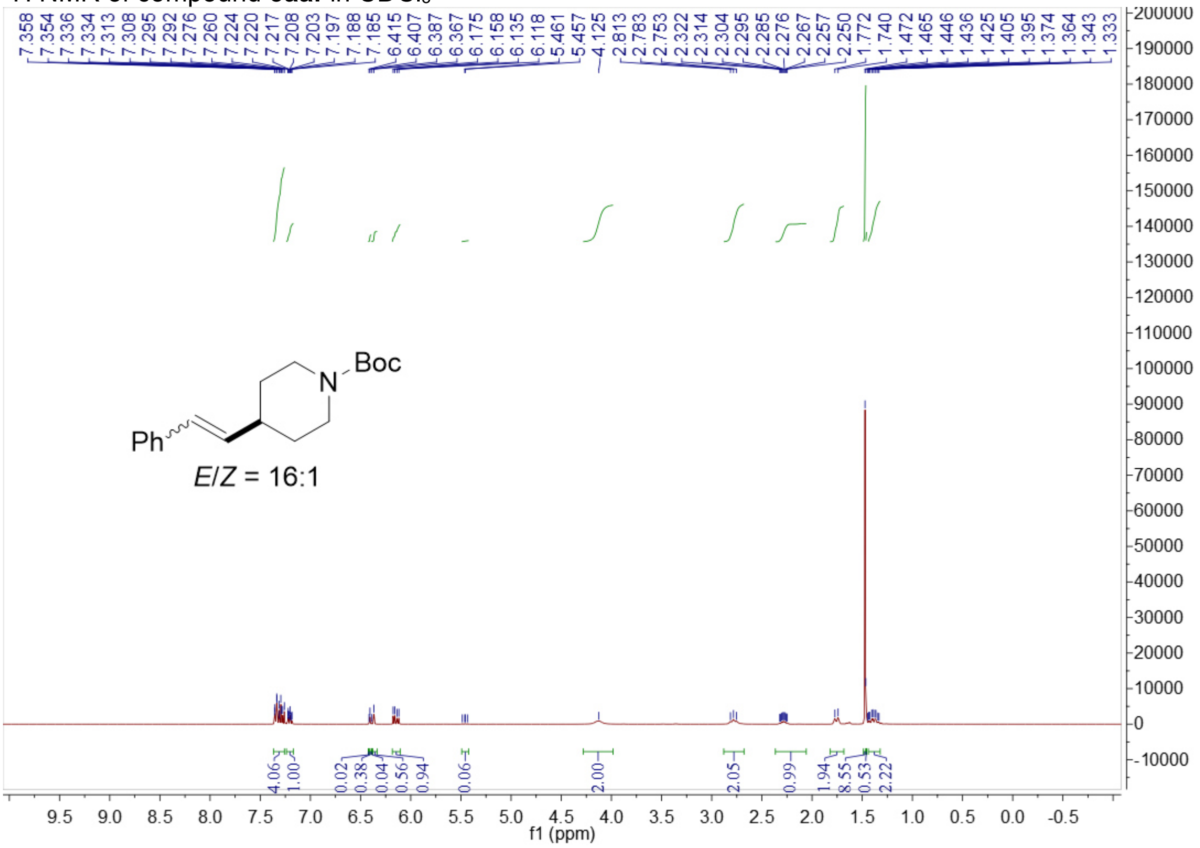

^{13}C NMR of compound **3aaf** in $CDCl_3$

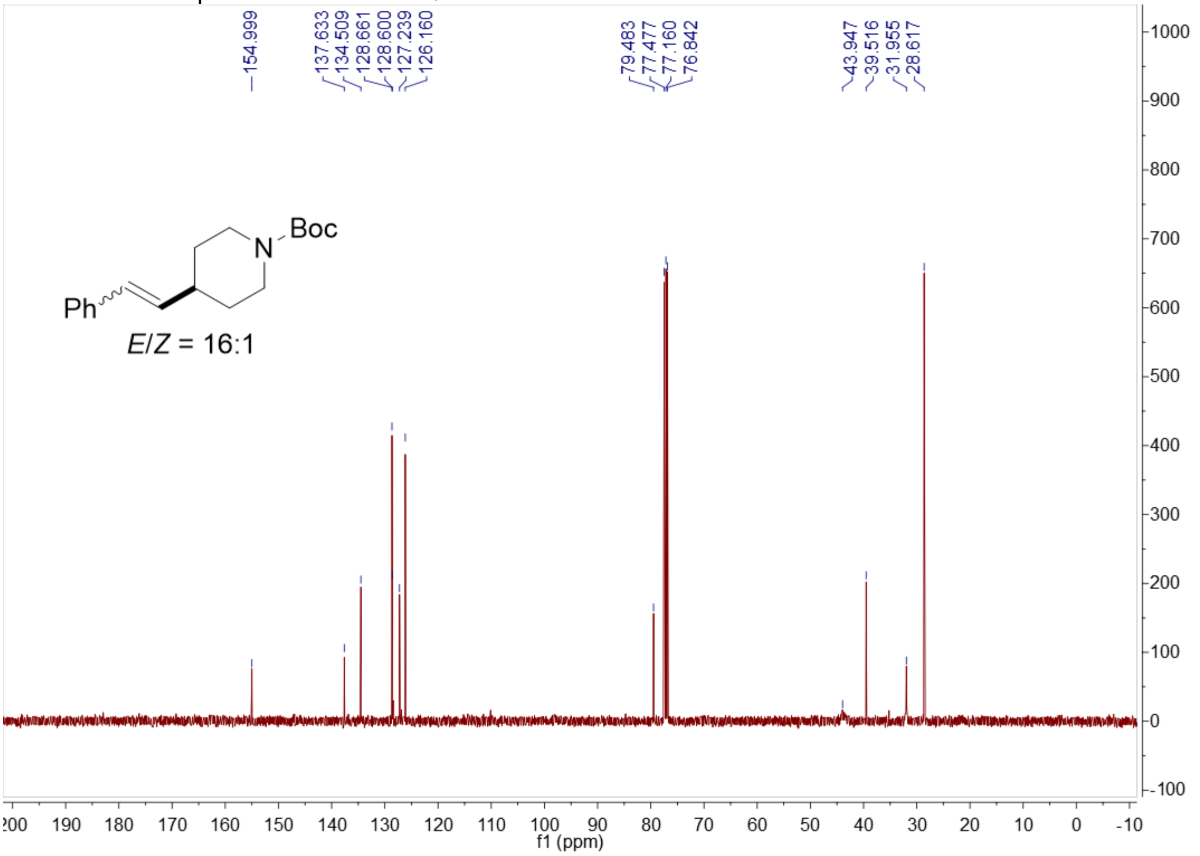

^{1}H NMR of compound **3aag** in $CDCl_3$

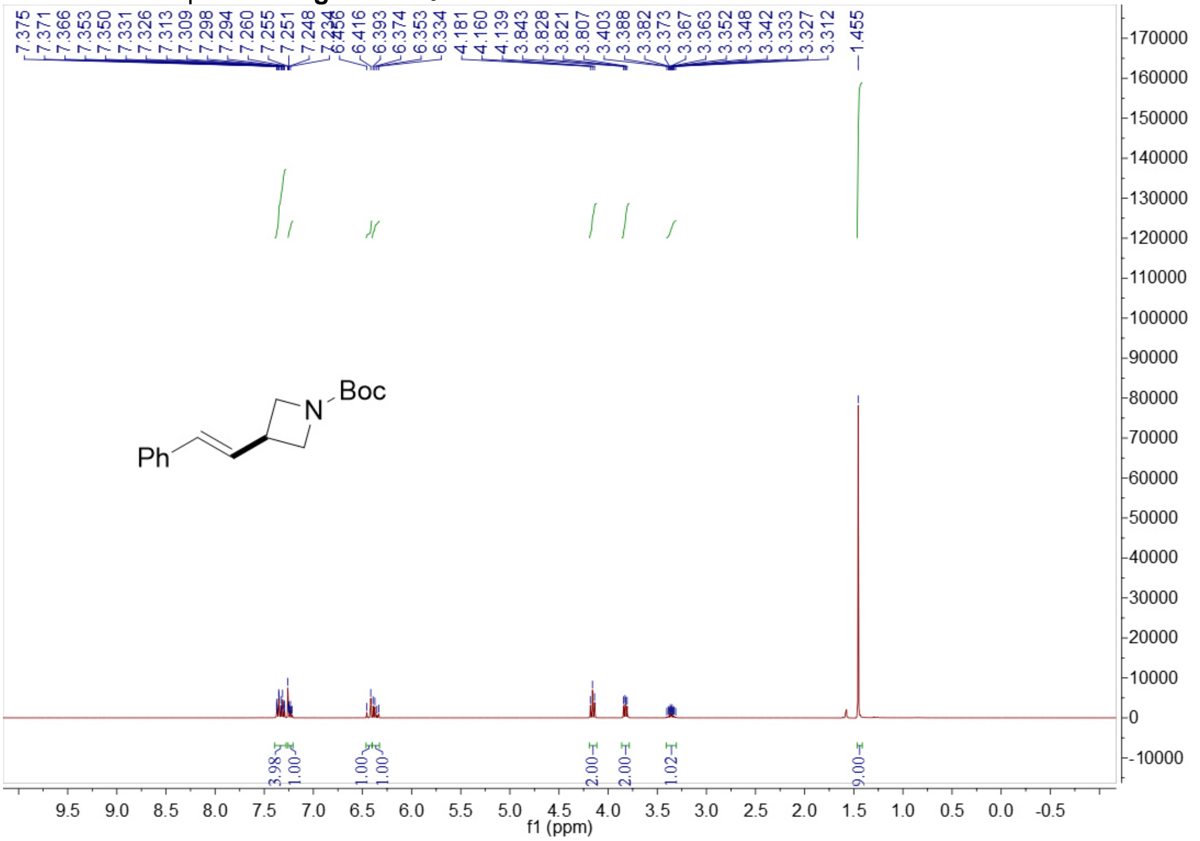

^{13}C NMR of compound **3aag** in $CDCl_3$

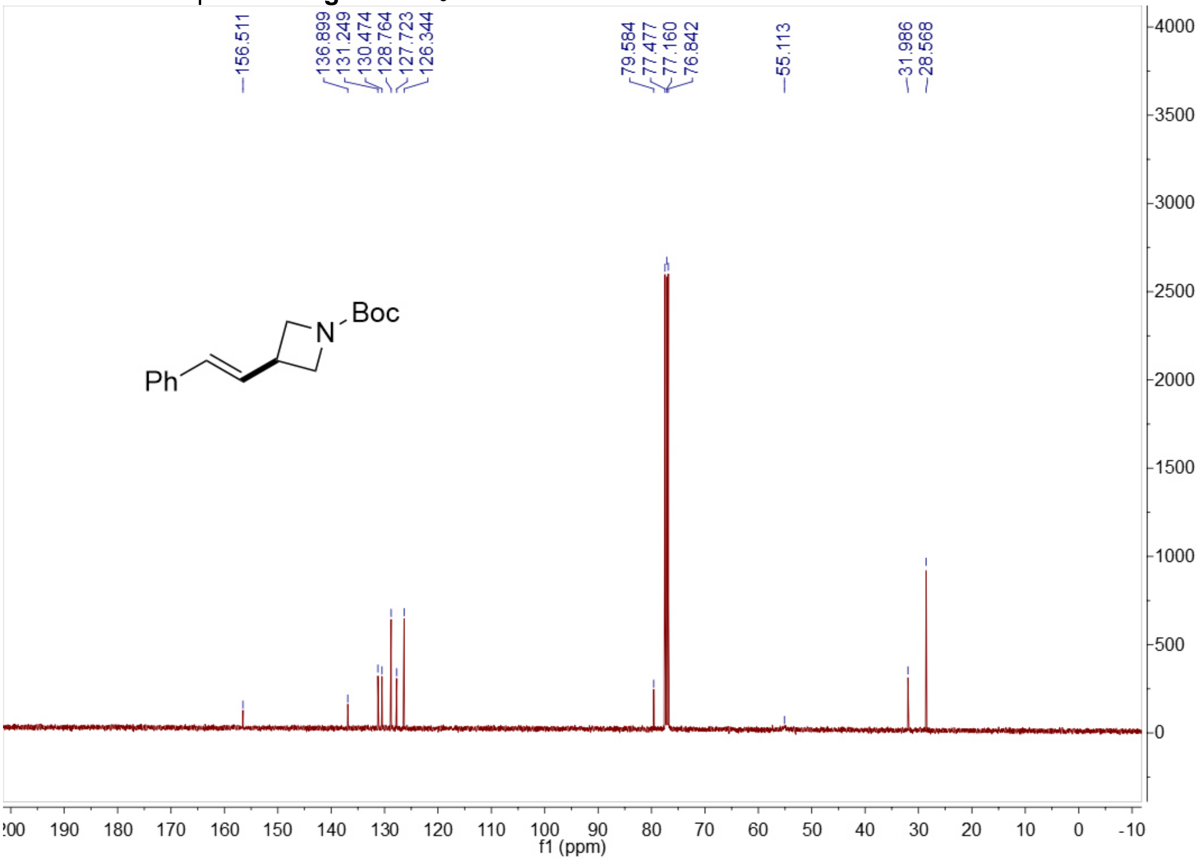

^{1}H NMR of compound **3aah** in $CDCl_3$

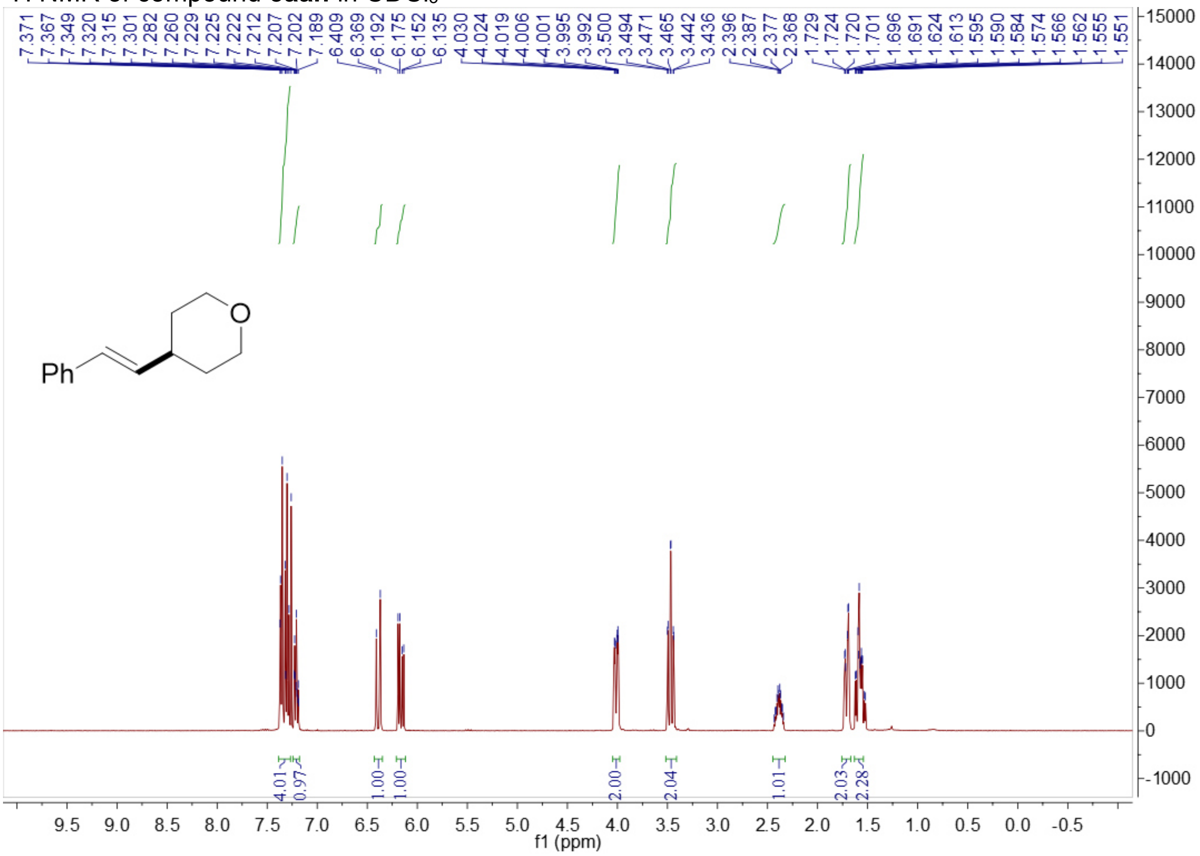

^{13}C NMR of compound **3aah** in $CDCl_3$

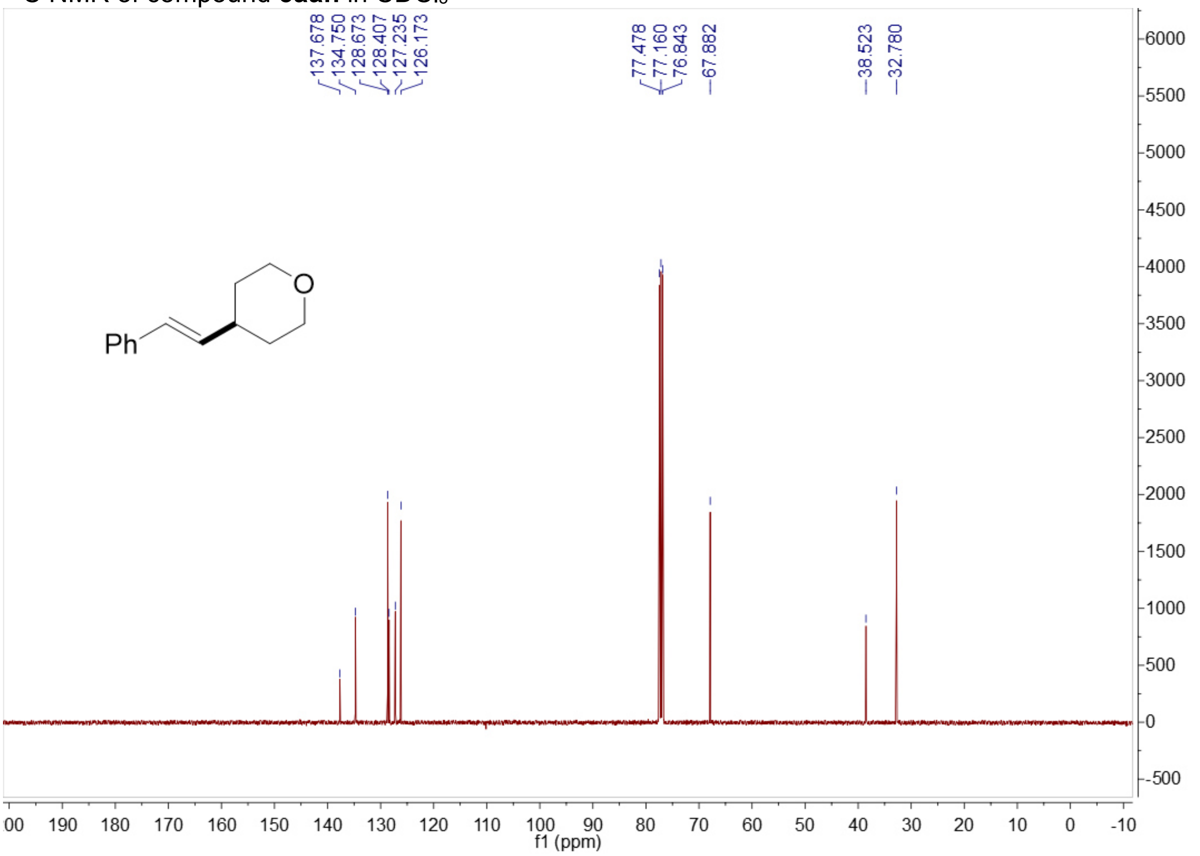

^{1}H NMR of compound **3aai** in $CDCl_3$

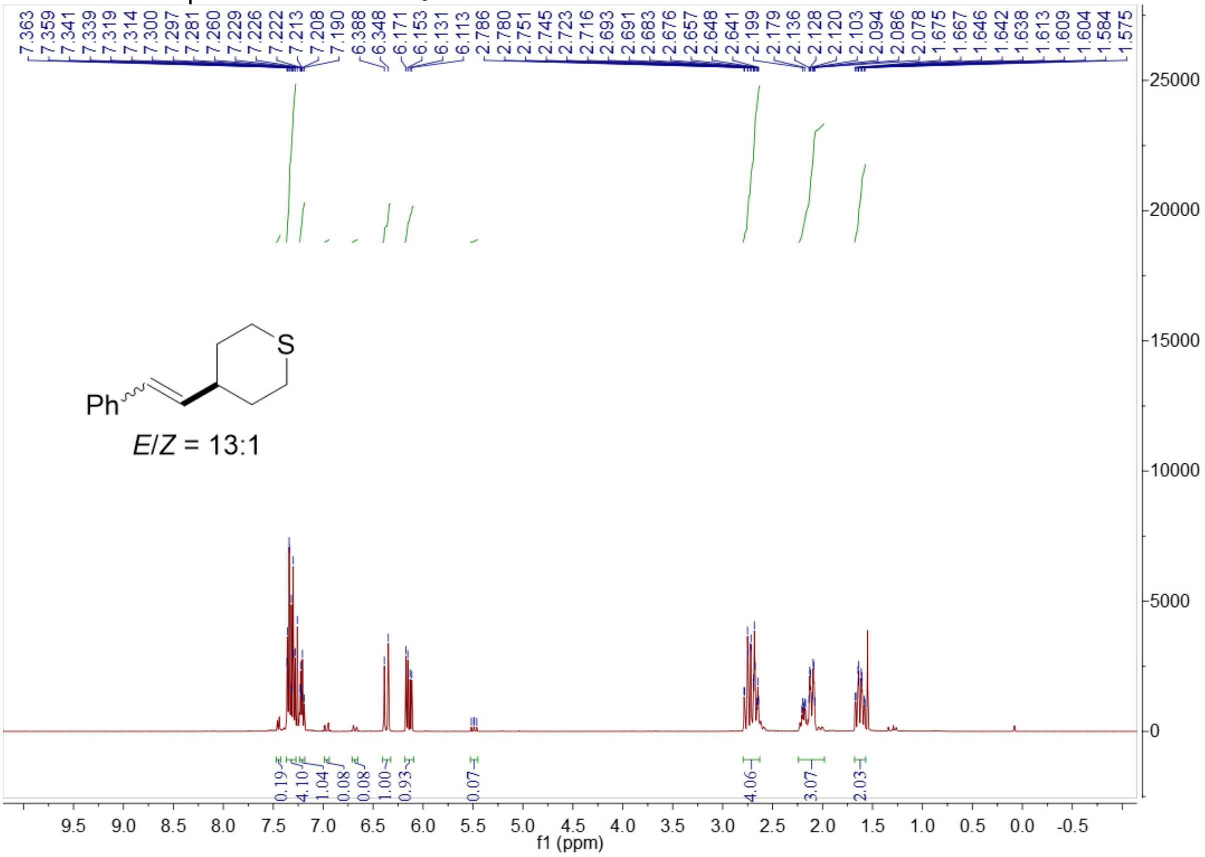

^{13}C NMR of compound **3aai** in $CDCl_3$

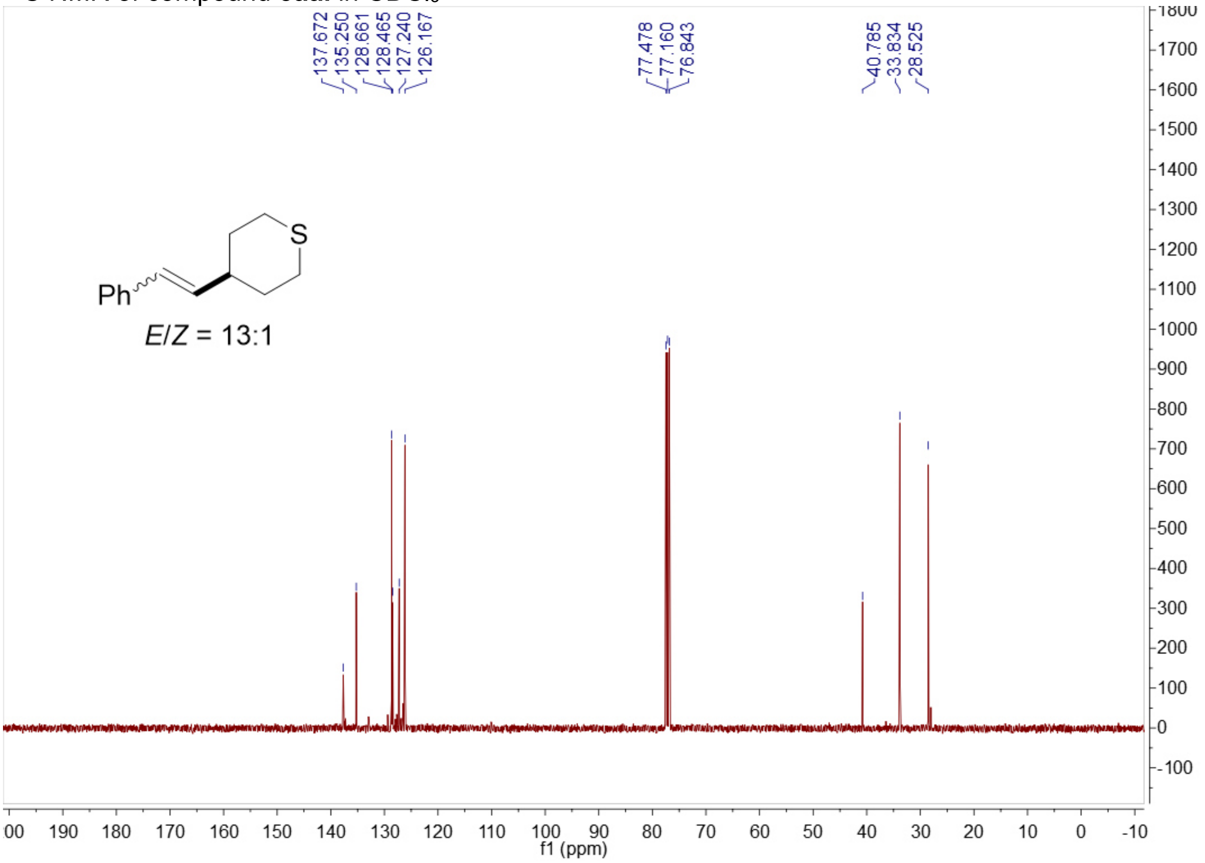

^{1}H NMR of compound **3aaj** in $CDCl_3$

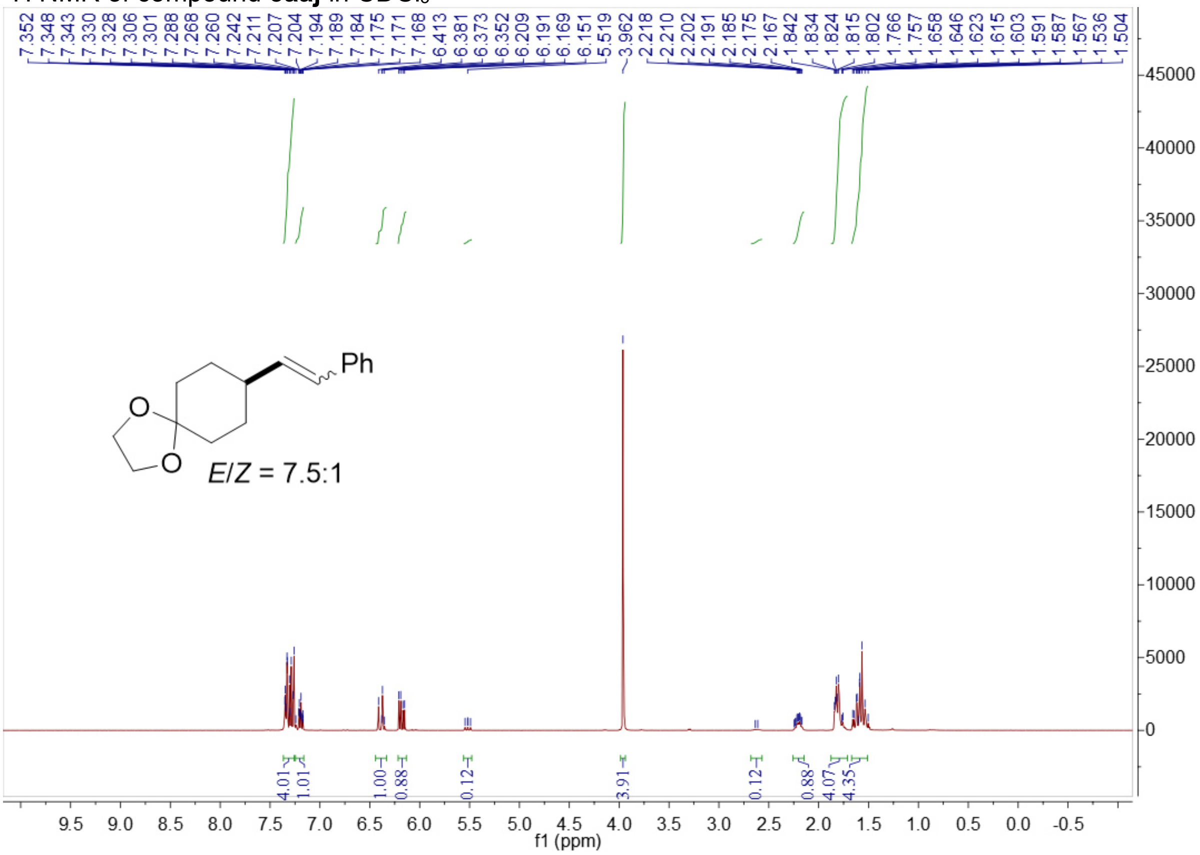

^{13}C NMR of compound **3aaj** in $CDCl_3$

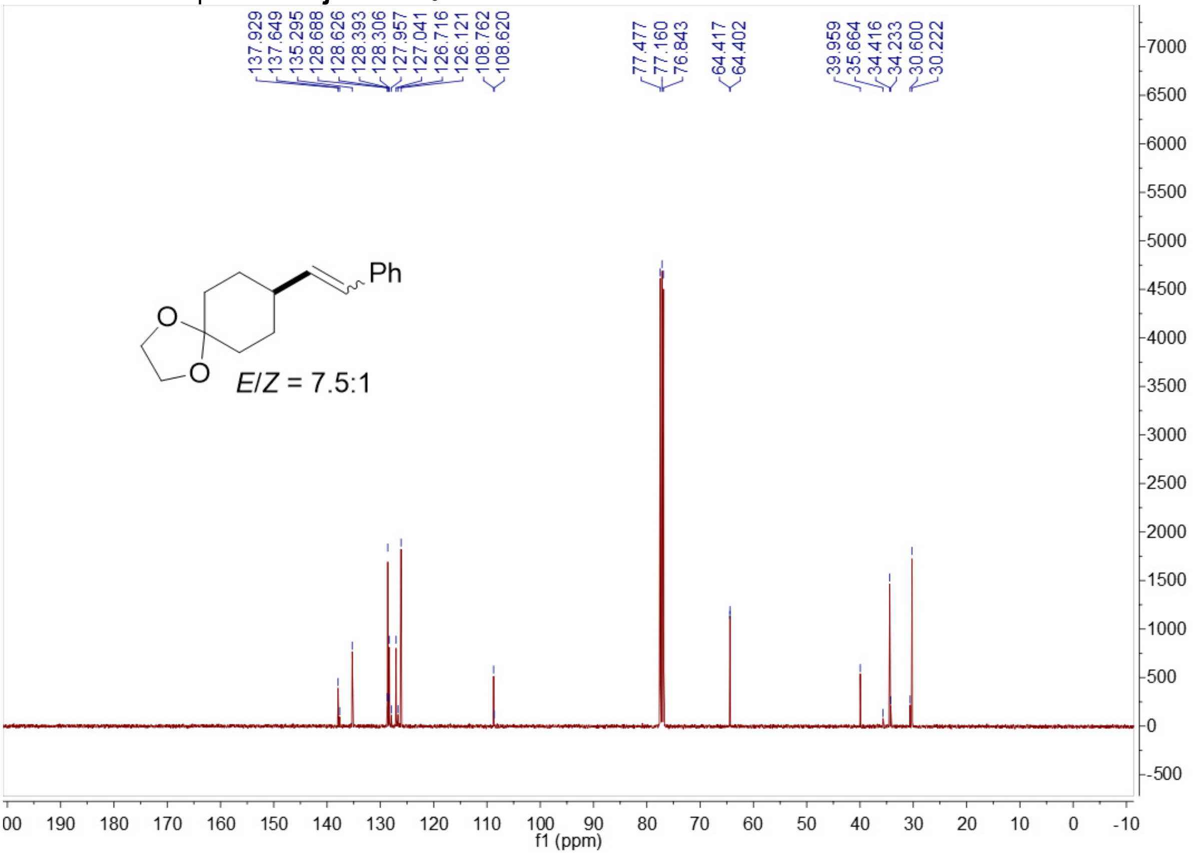

^{1}H NMR of compound **3lk** in $CDCl_3$

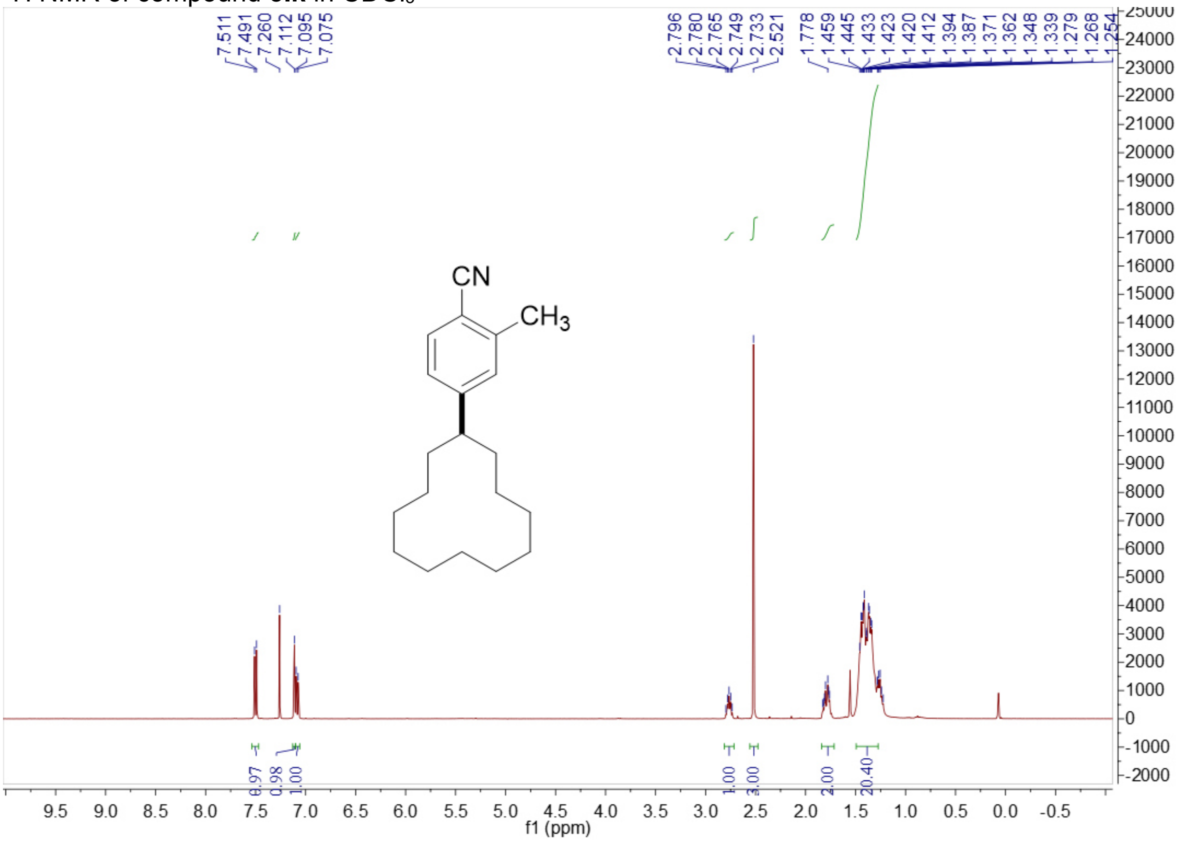

^{13}C NMR of compound **3lk** in $CDCl_3$

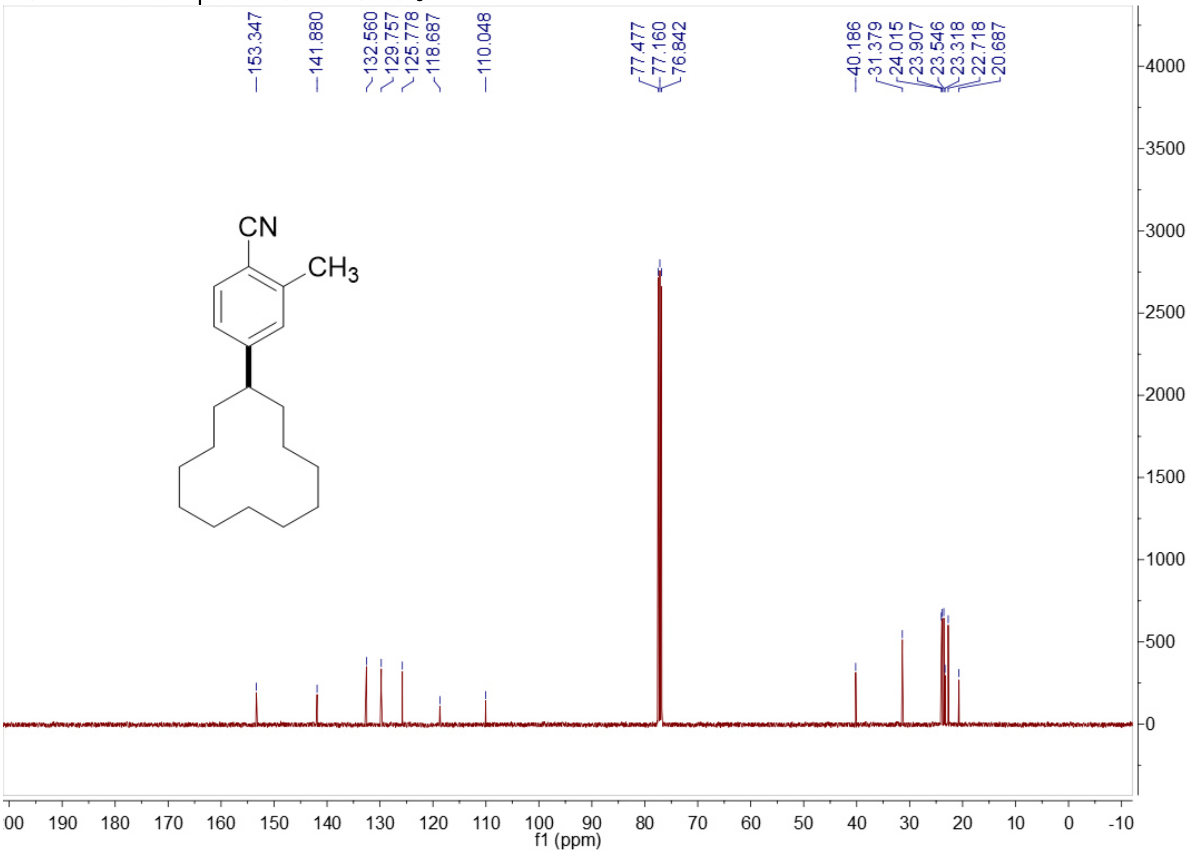

^{1}H NMR of compound **3fl** in CDCl$_3$

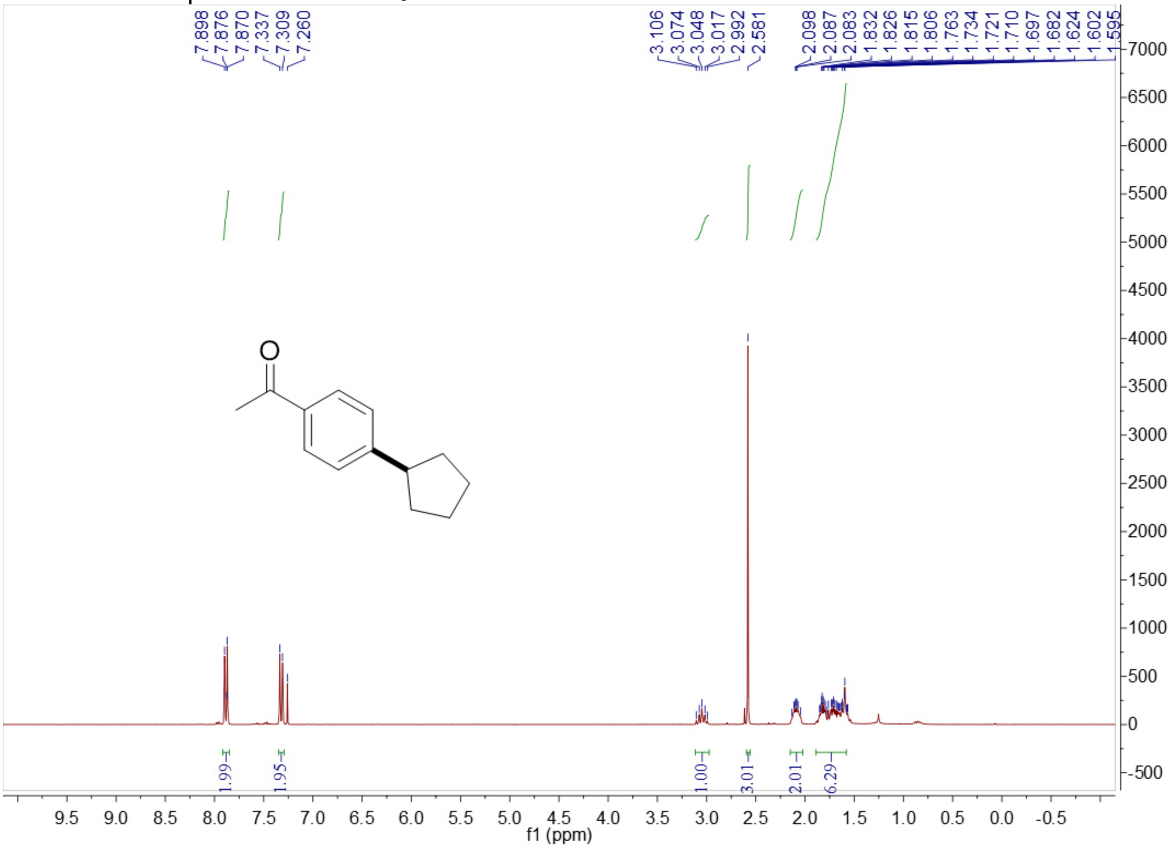

^{13}C NMR of compound **3fl** in CDCl$_3$

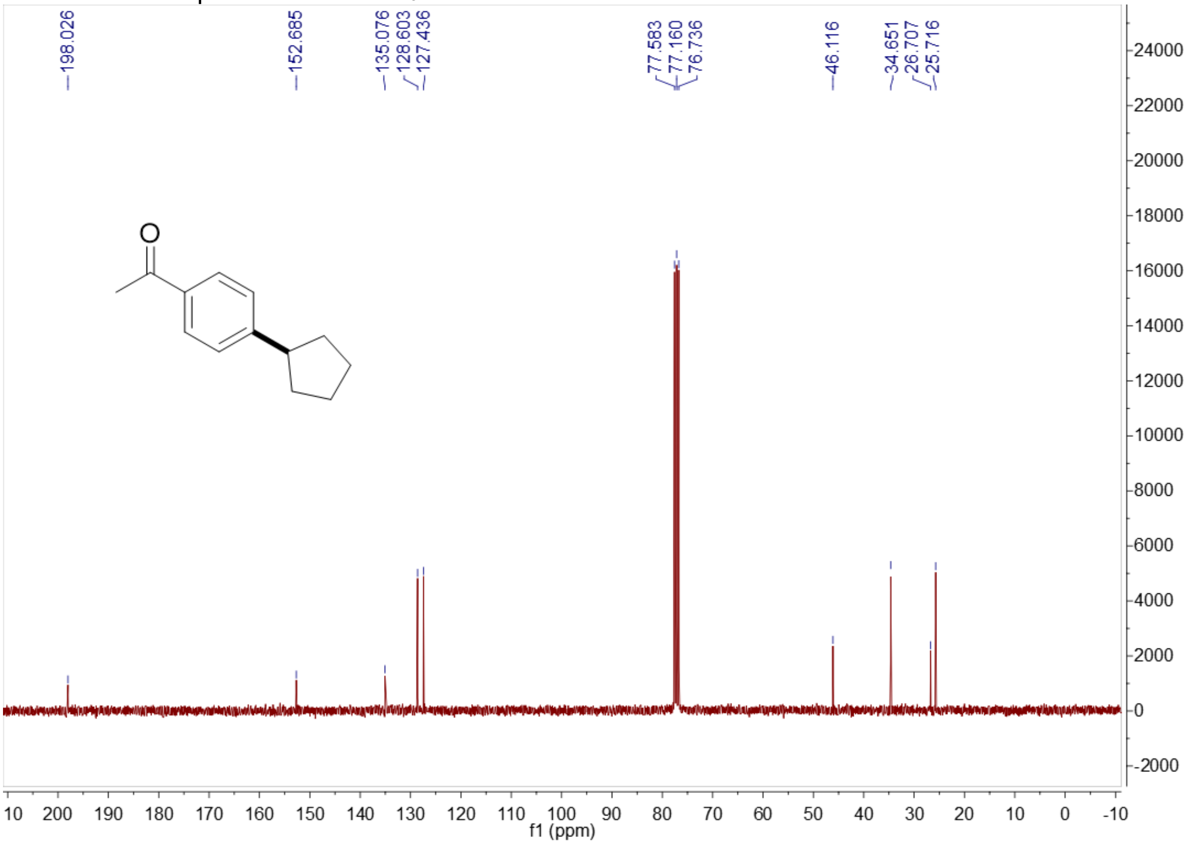

^{1}H NMR of compound **3fm** in $CDCl_3$

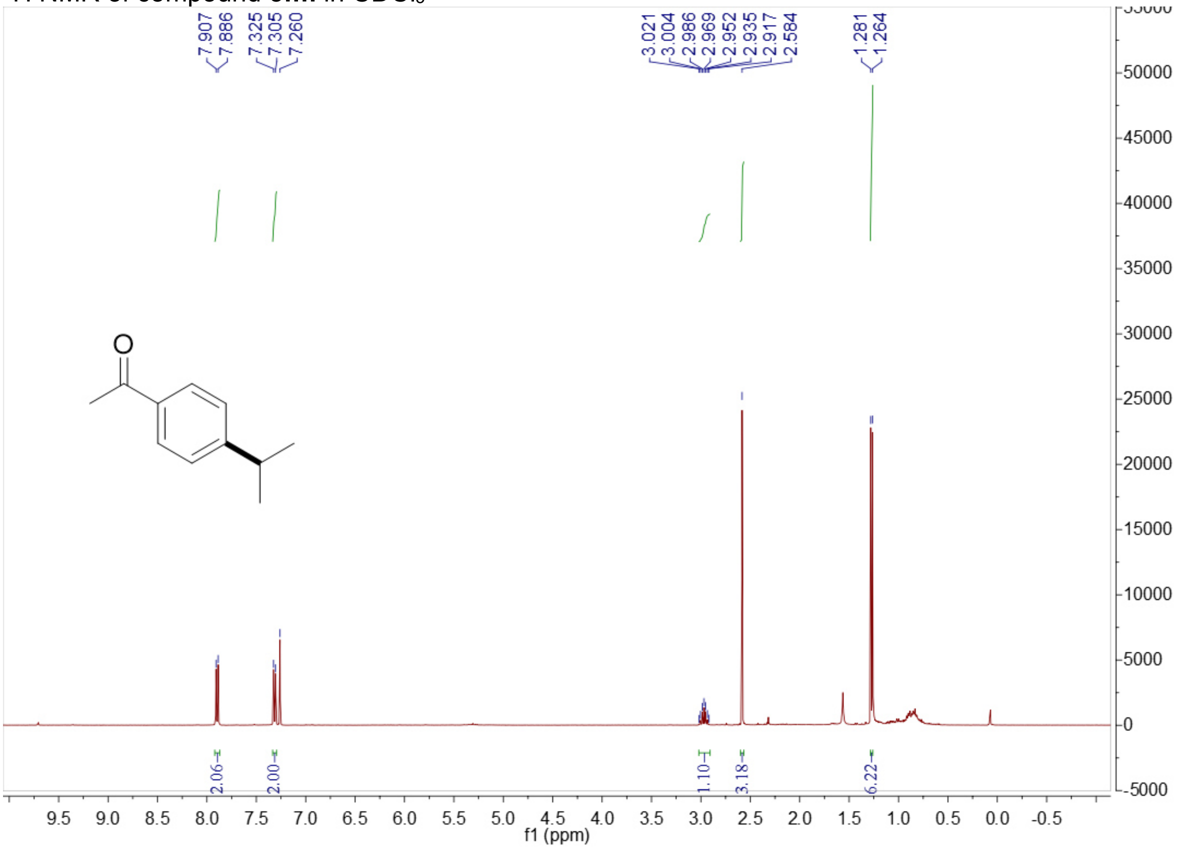

^{13}C NMR of compound **3fm** in $CDCl_3$

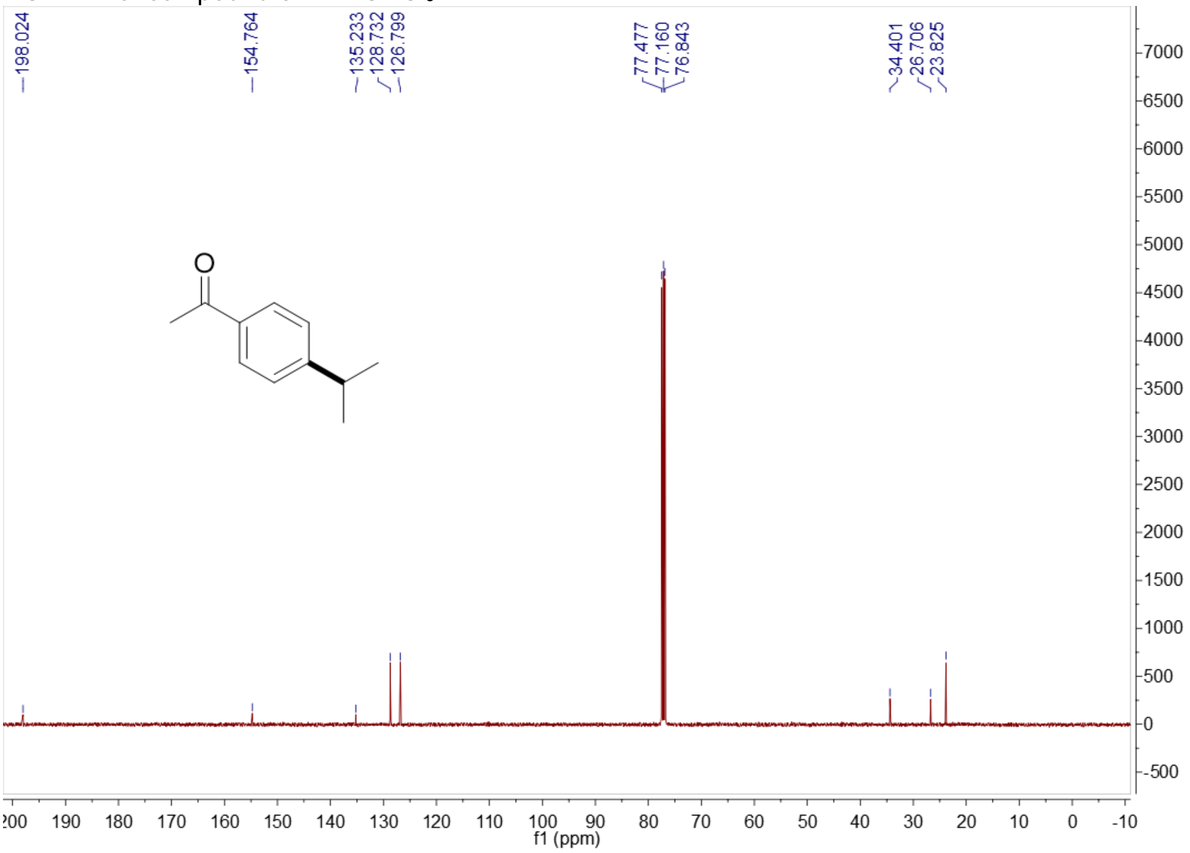

^{1}H NMR of compound **3ln** in $CDCl_3$

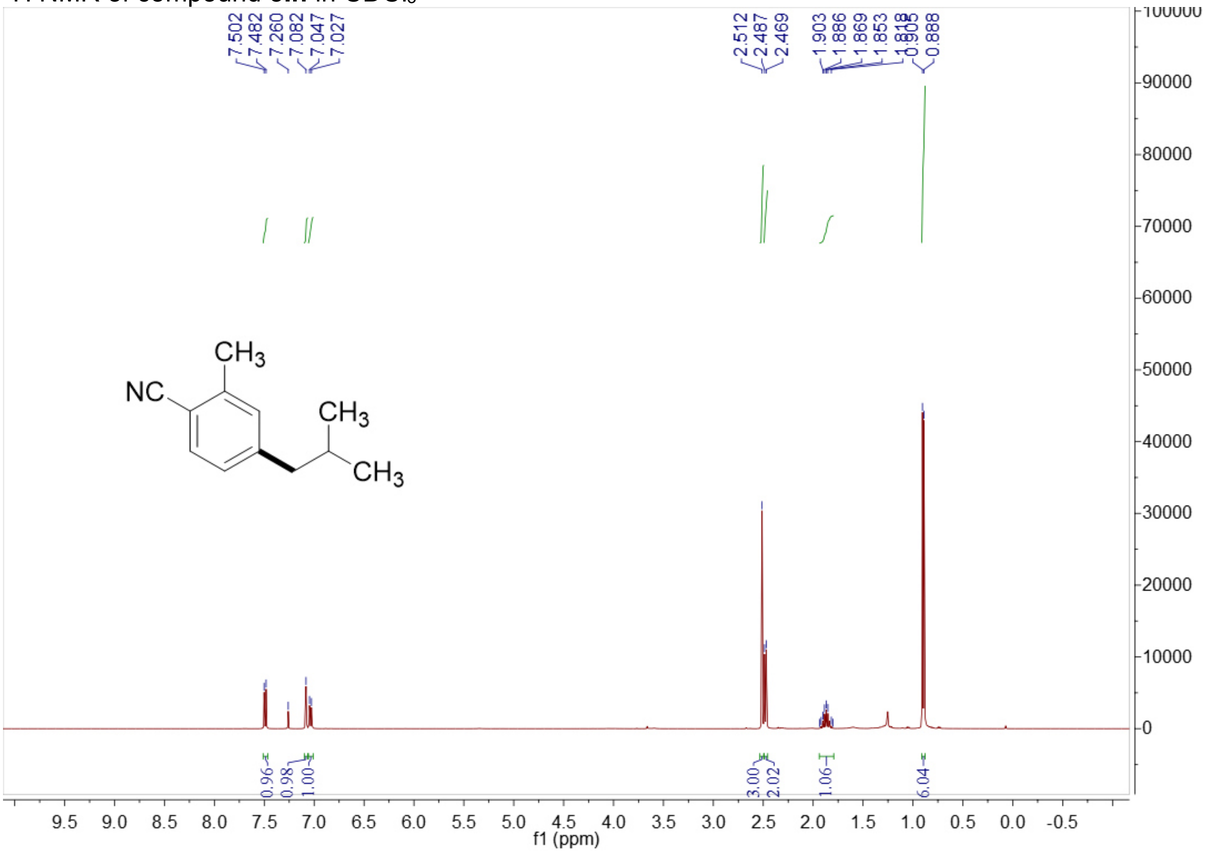

^{13}C NMR of compound **3ln** in $CDCl_3$

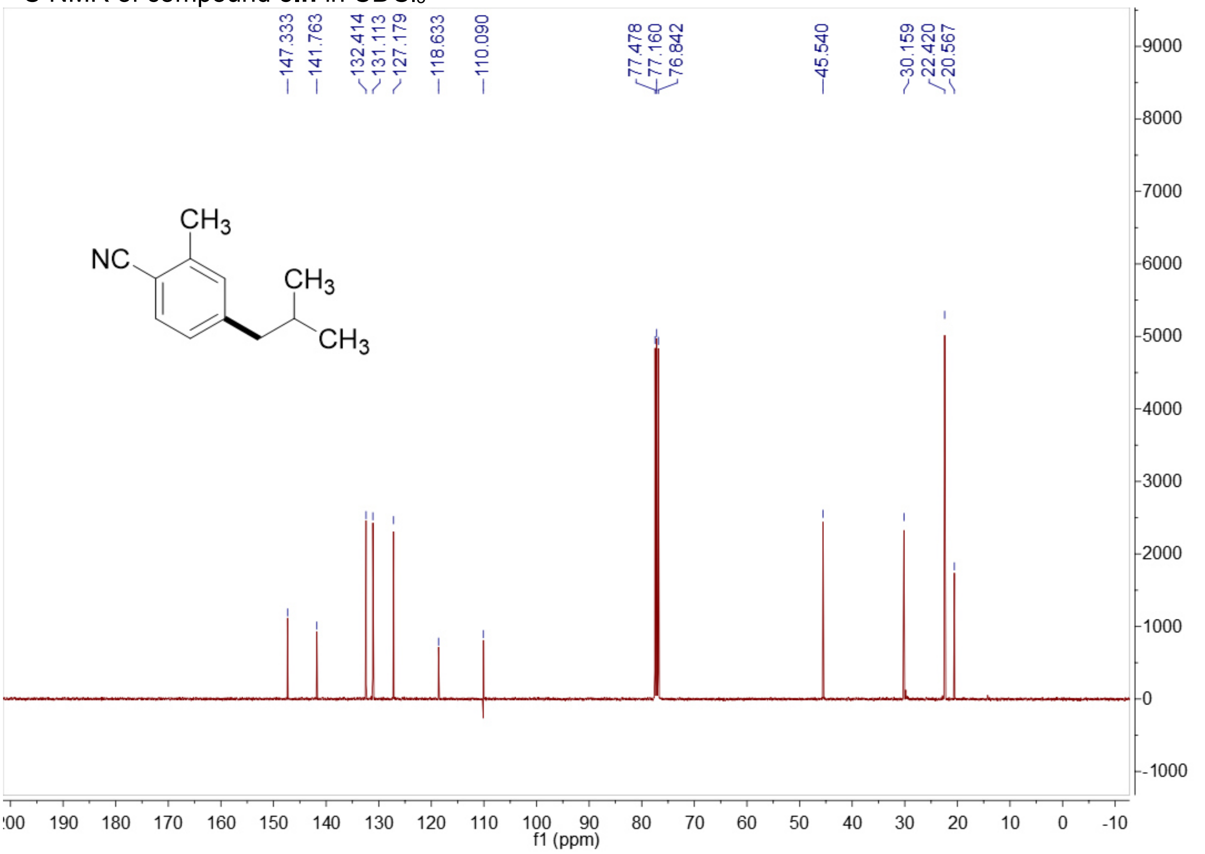

^{1}H NMR of compound **3qo** in $CDCl_3$

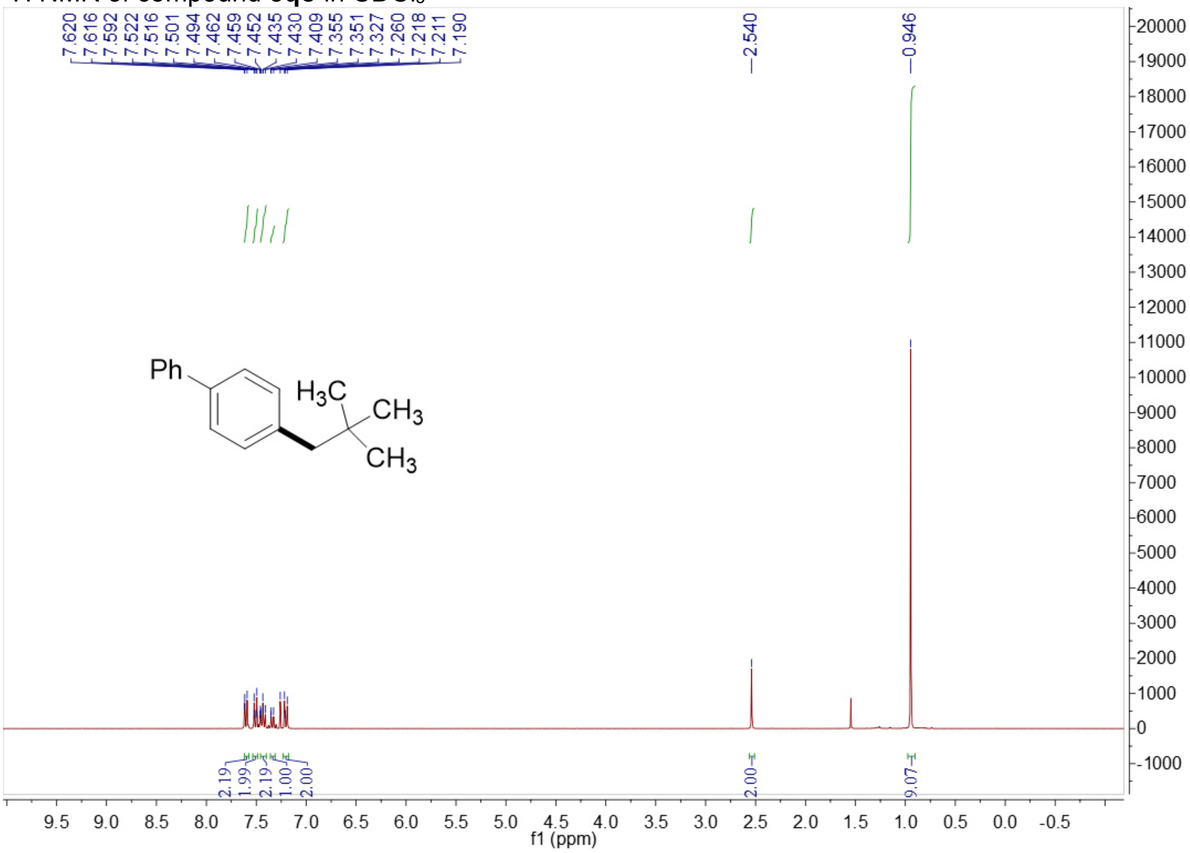

^{13}C NMR of compound **3qo** in $CDCl_3$

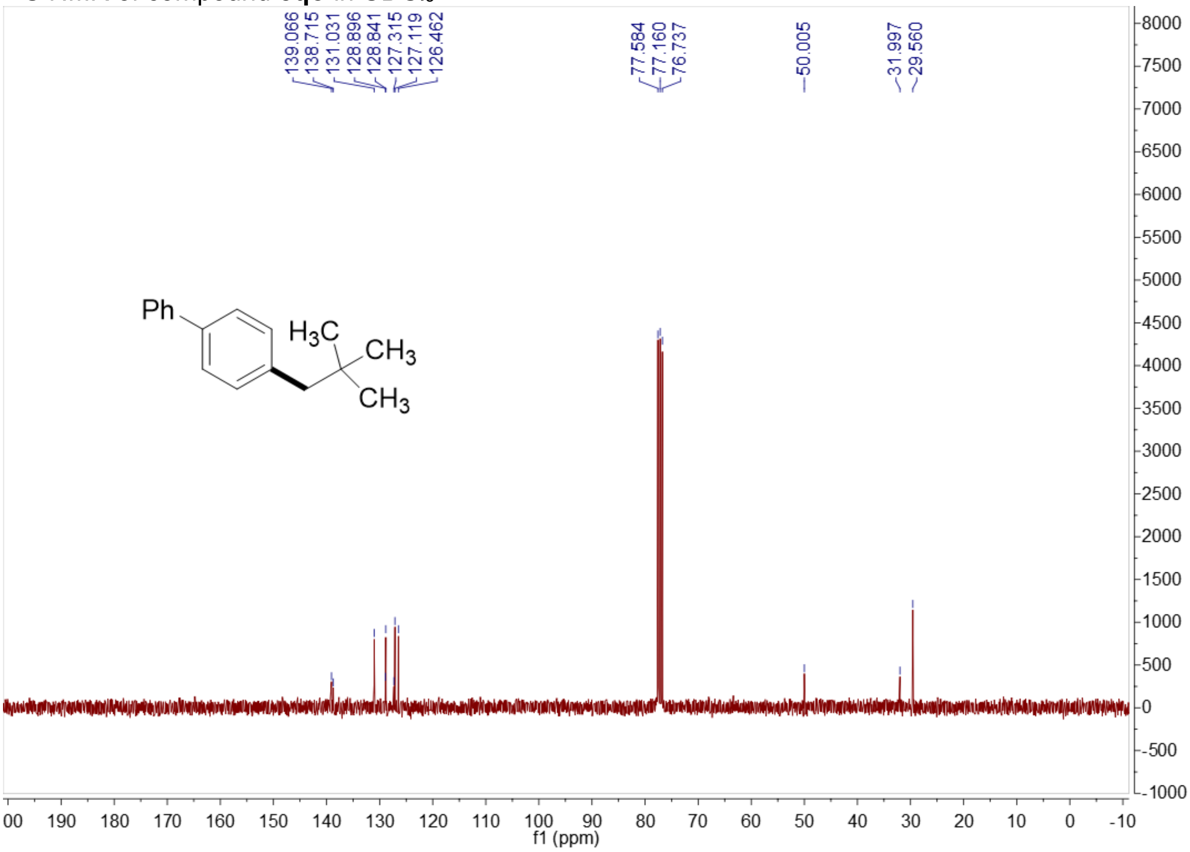

^{1}H NMR of compound **3lp** in $CDCl_3$

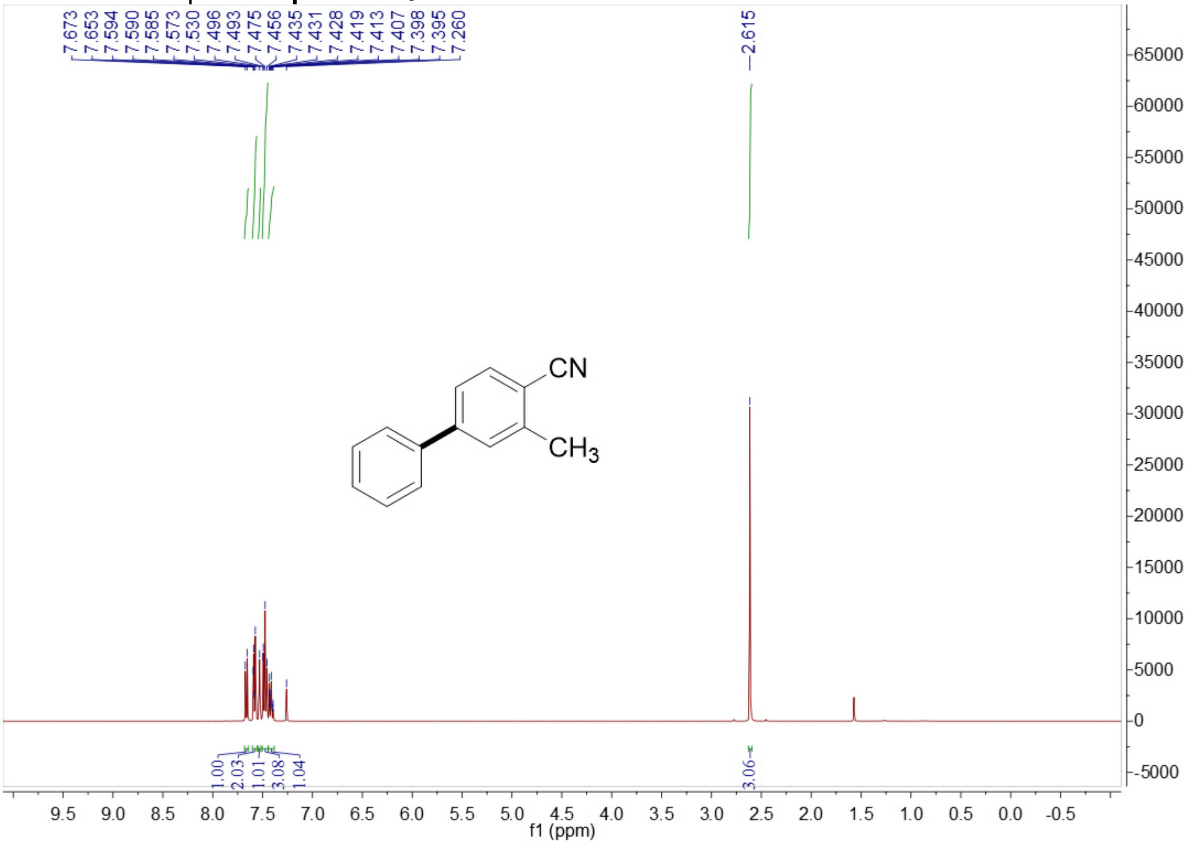

^{13}C NMR of compound **3lp** in $CDCl_3$

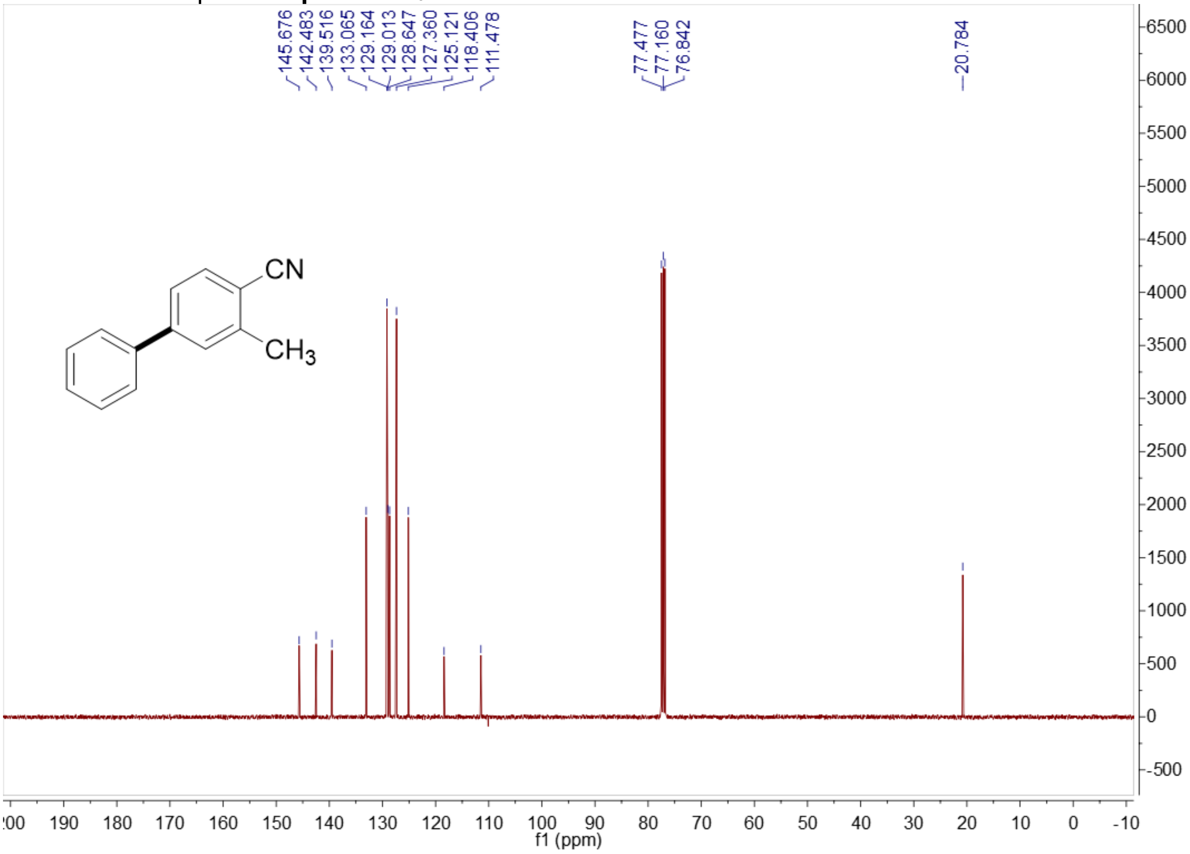

^1H NMR of compound **3jq** in $CDCl_3$

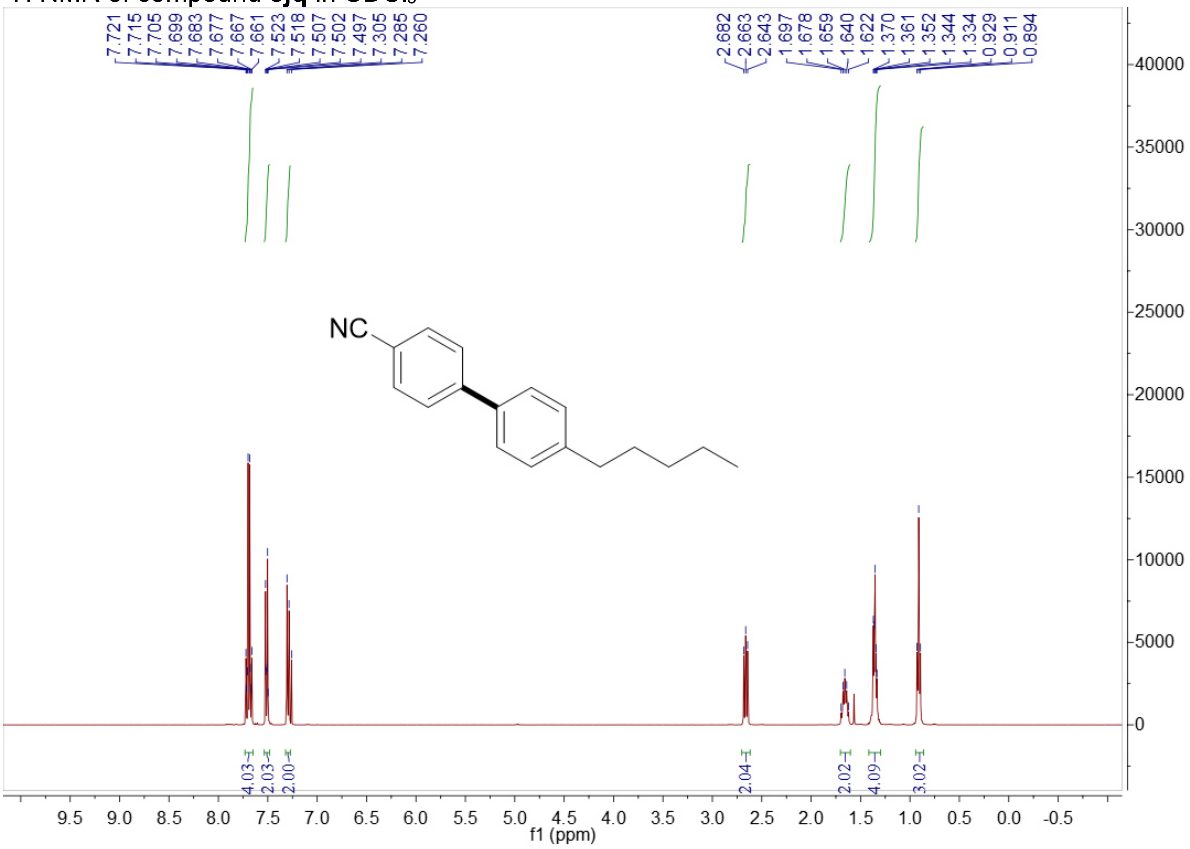

^{13}C NMR of compound **3jq** in $CDCl_3$

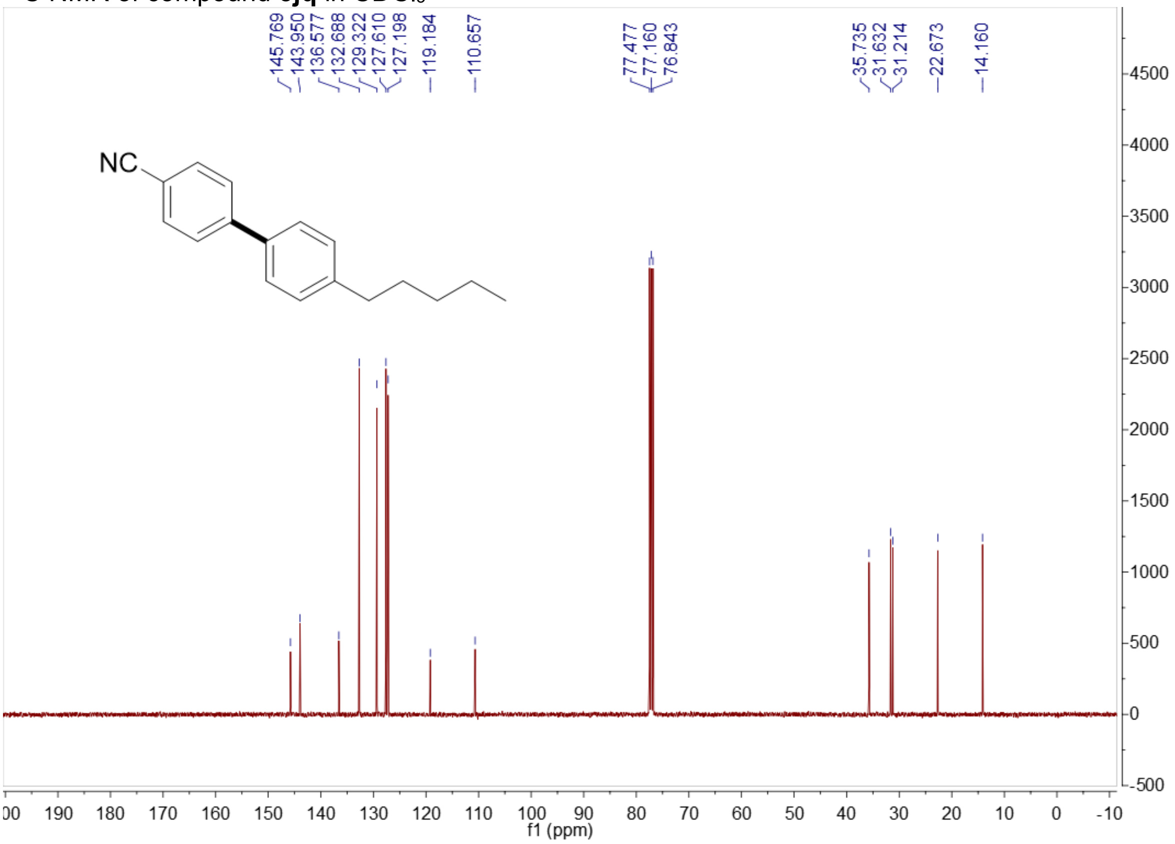

^{1}H NMR of compound **3wp** in $CDCl_3$

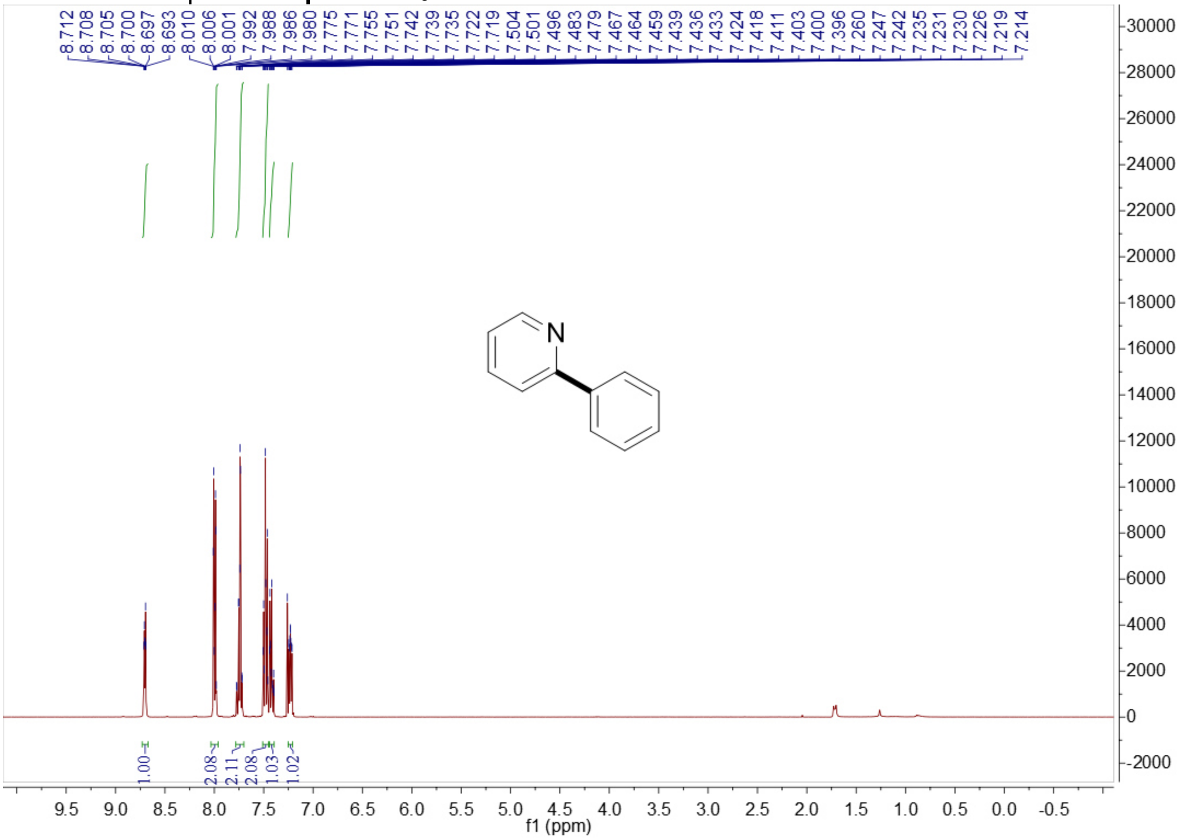

^{13}C NMR of compound **3wp** in $CDCl_3$

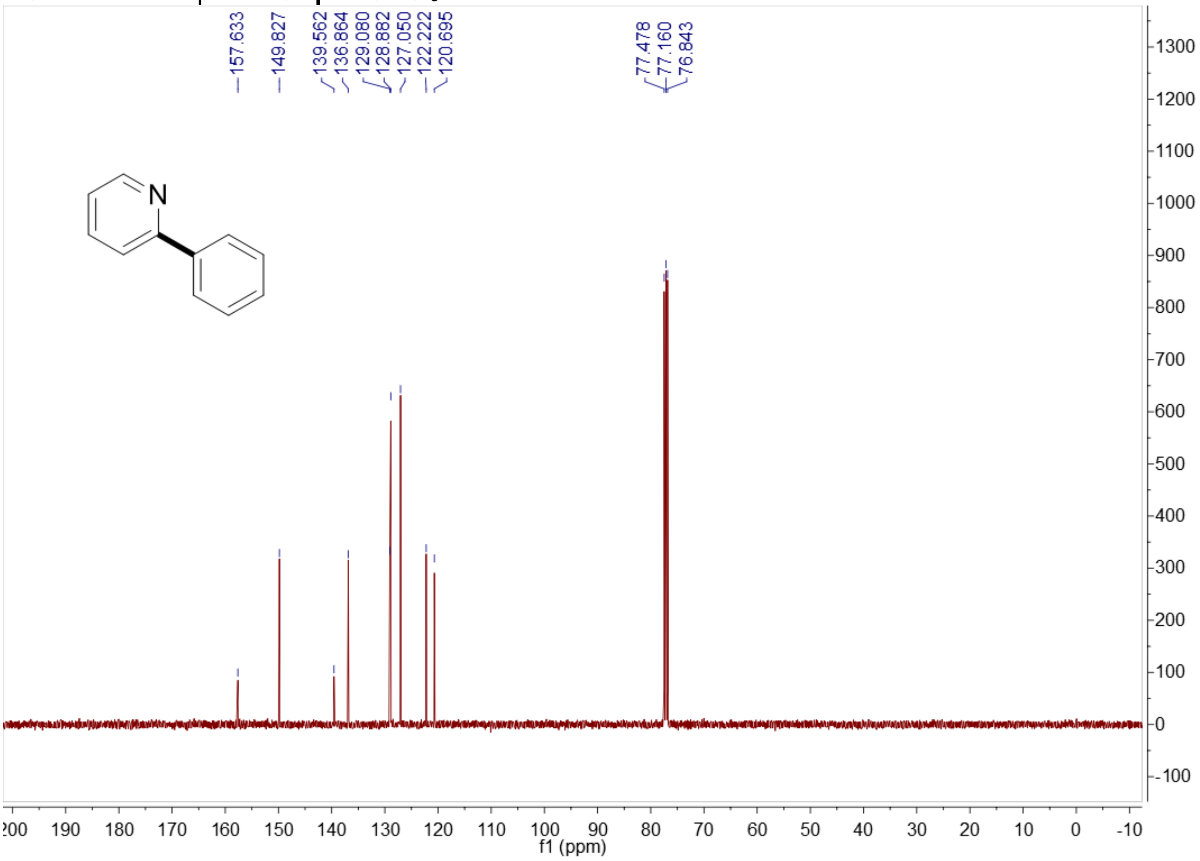

^{1}H NMR of compound **3lr** in $CDCl_3$

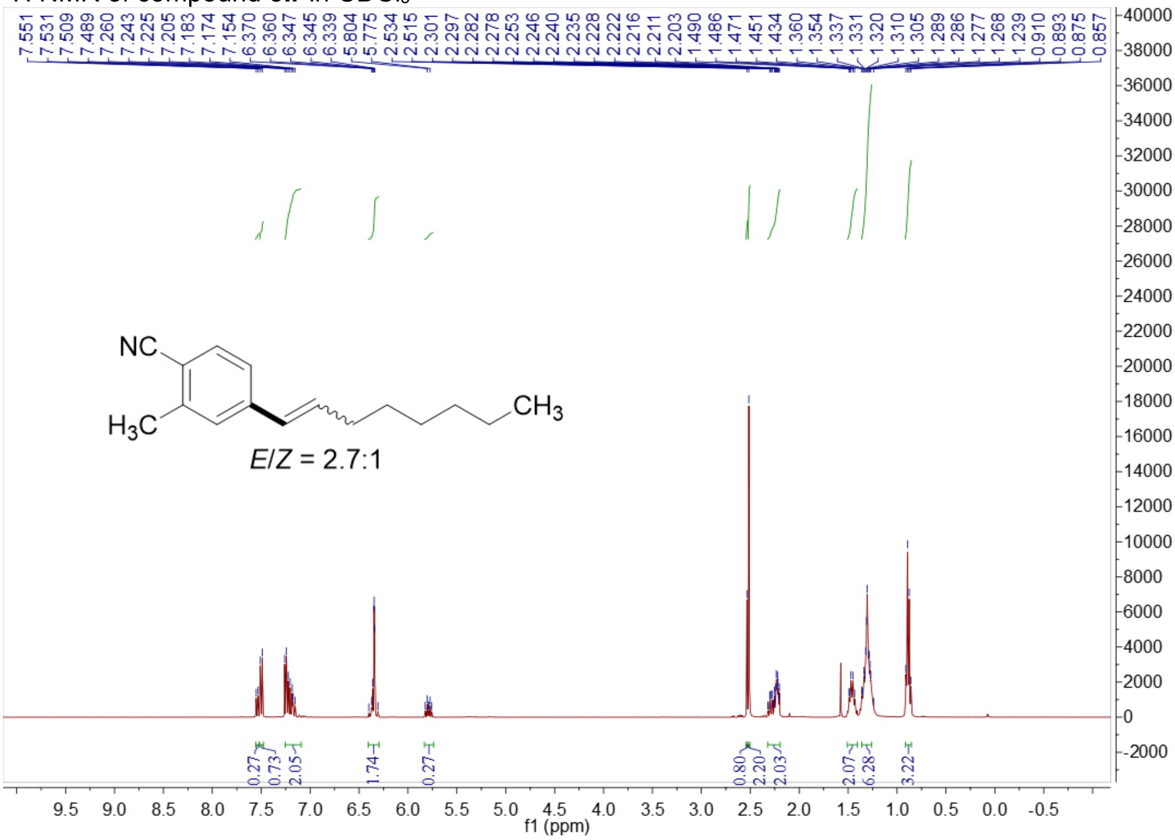

^{13}C NMR of compound **3lr** in $CDCl_3$

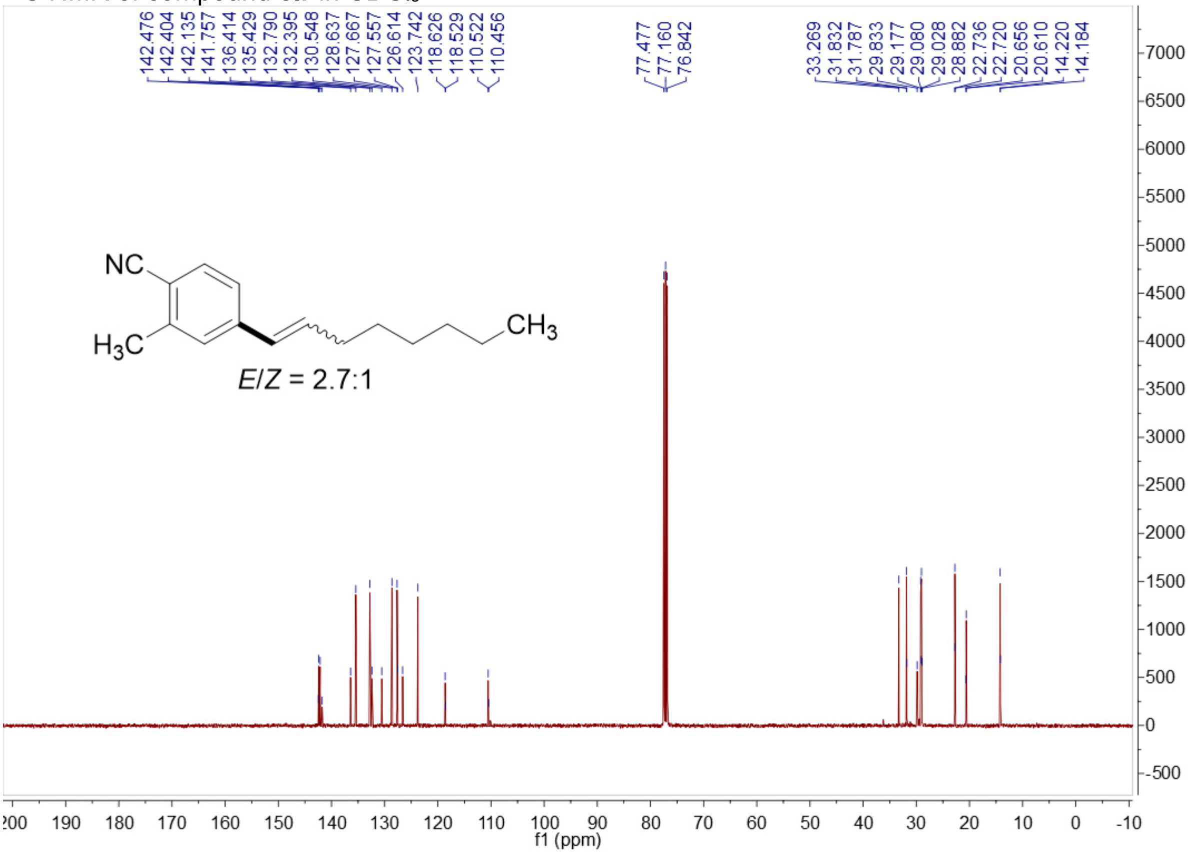

^{1}H NMR of compound **3ls** in $CDCl_3$

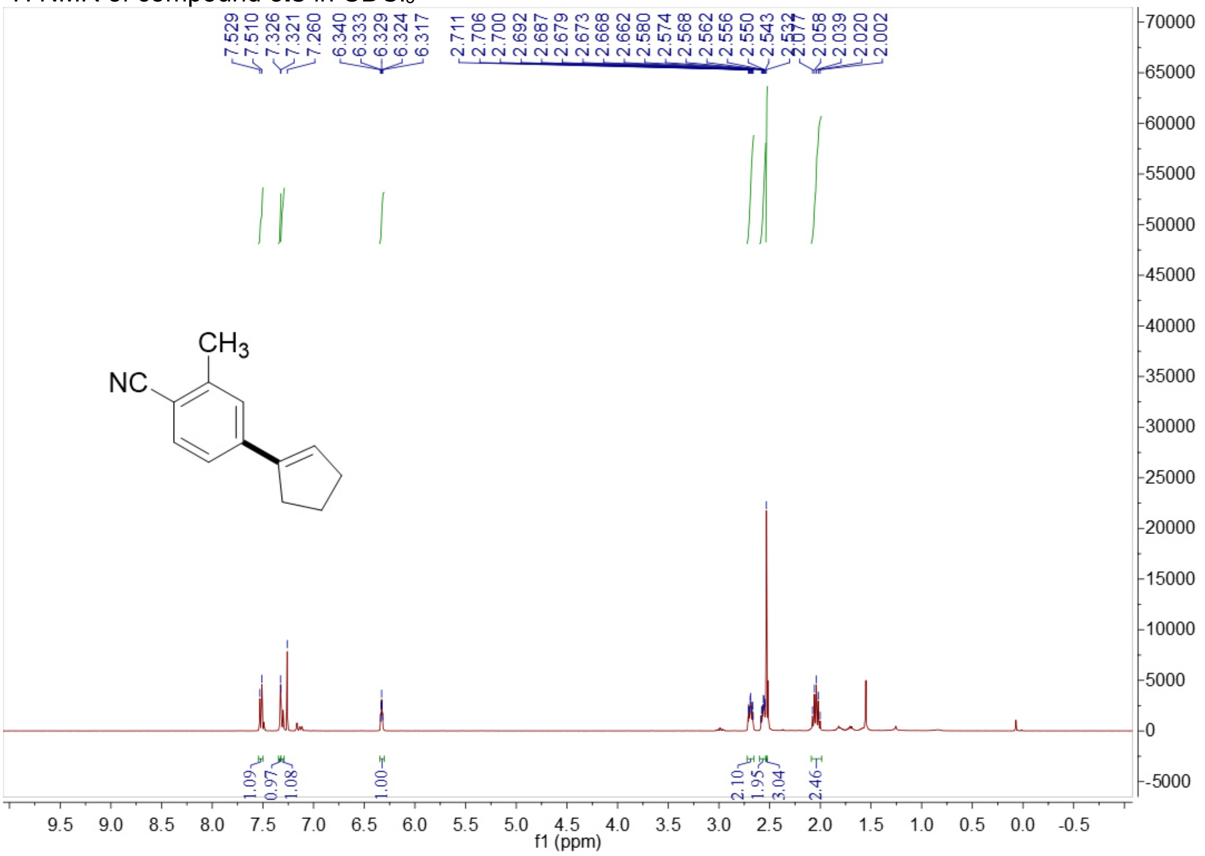

^{13}C NMR of compound **3ls** in $CDCl_3$

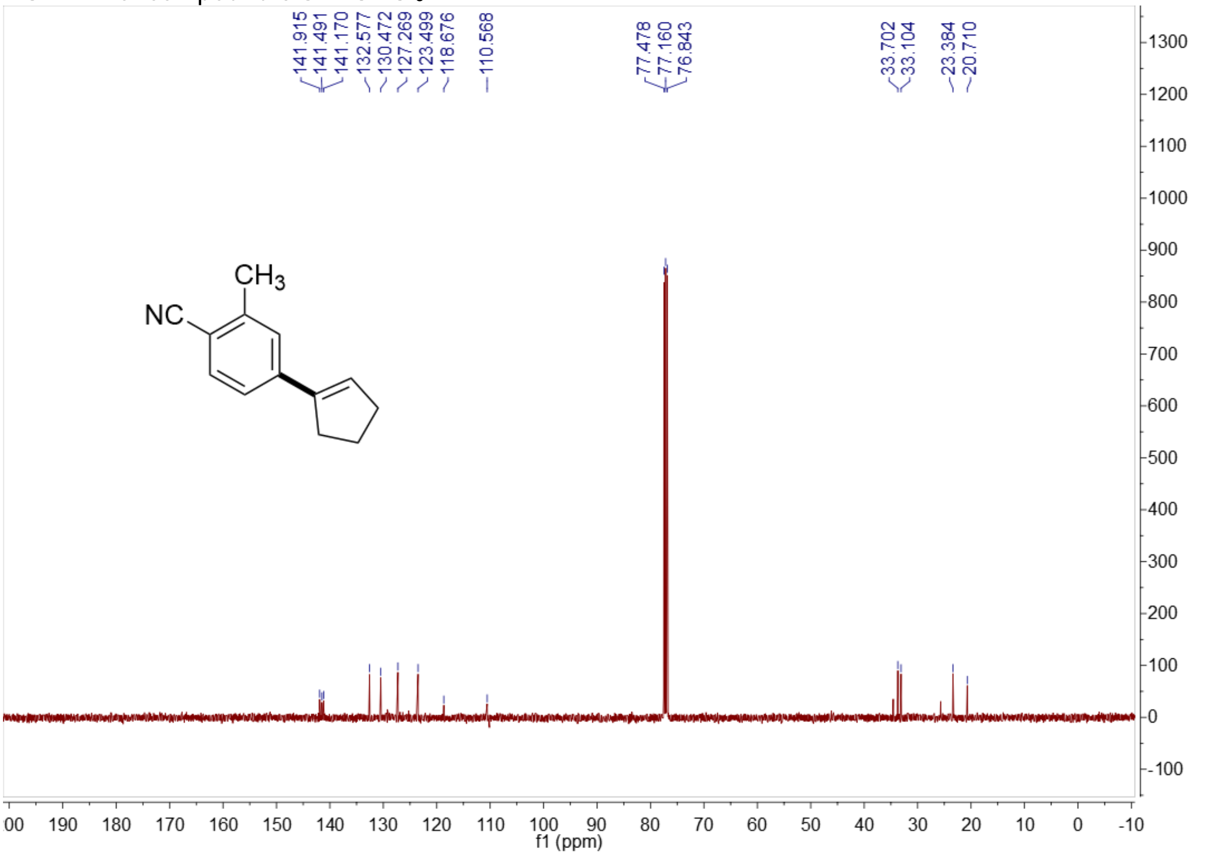

^{1}H NMR of compound **3lt** in $CDCl_3$

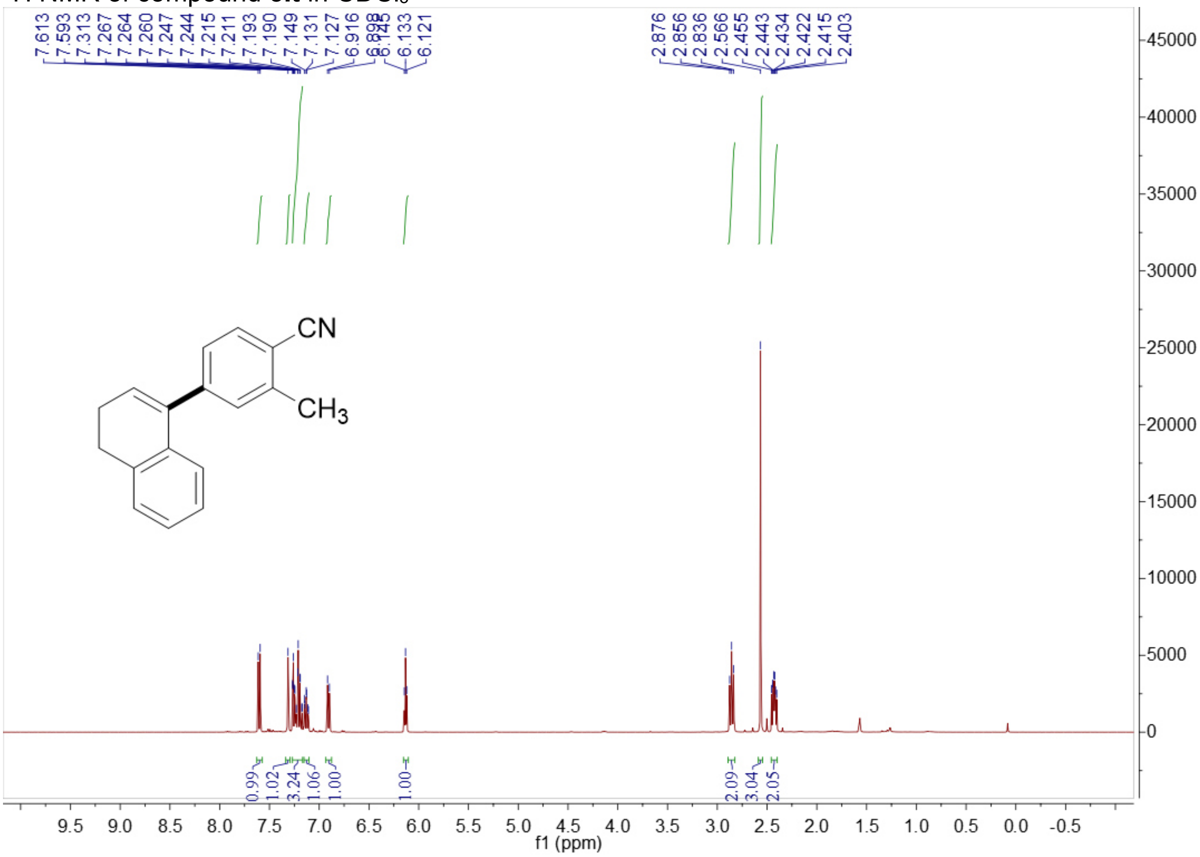

^{13}C NMR of compound **3lt** in $CDCl_3$

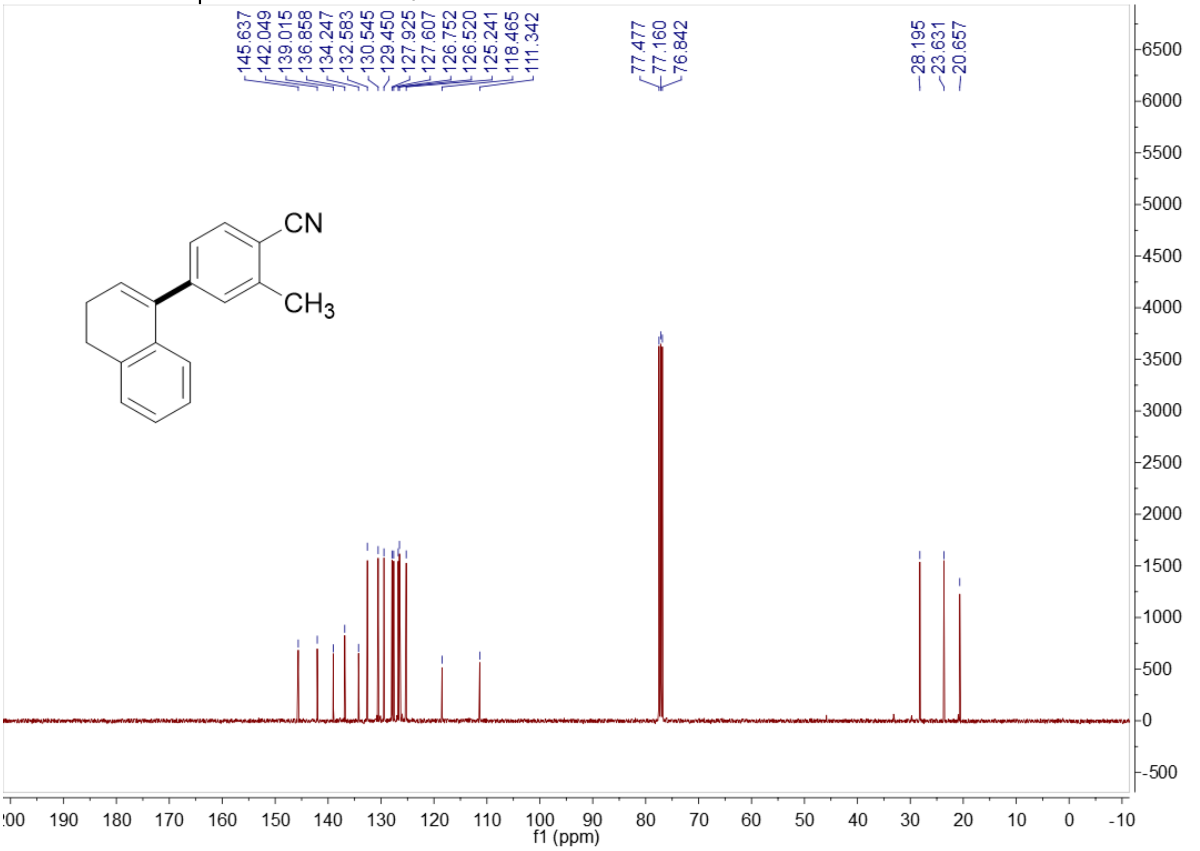

^{1}H NMR of compound **3du** in $CDCl_3$

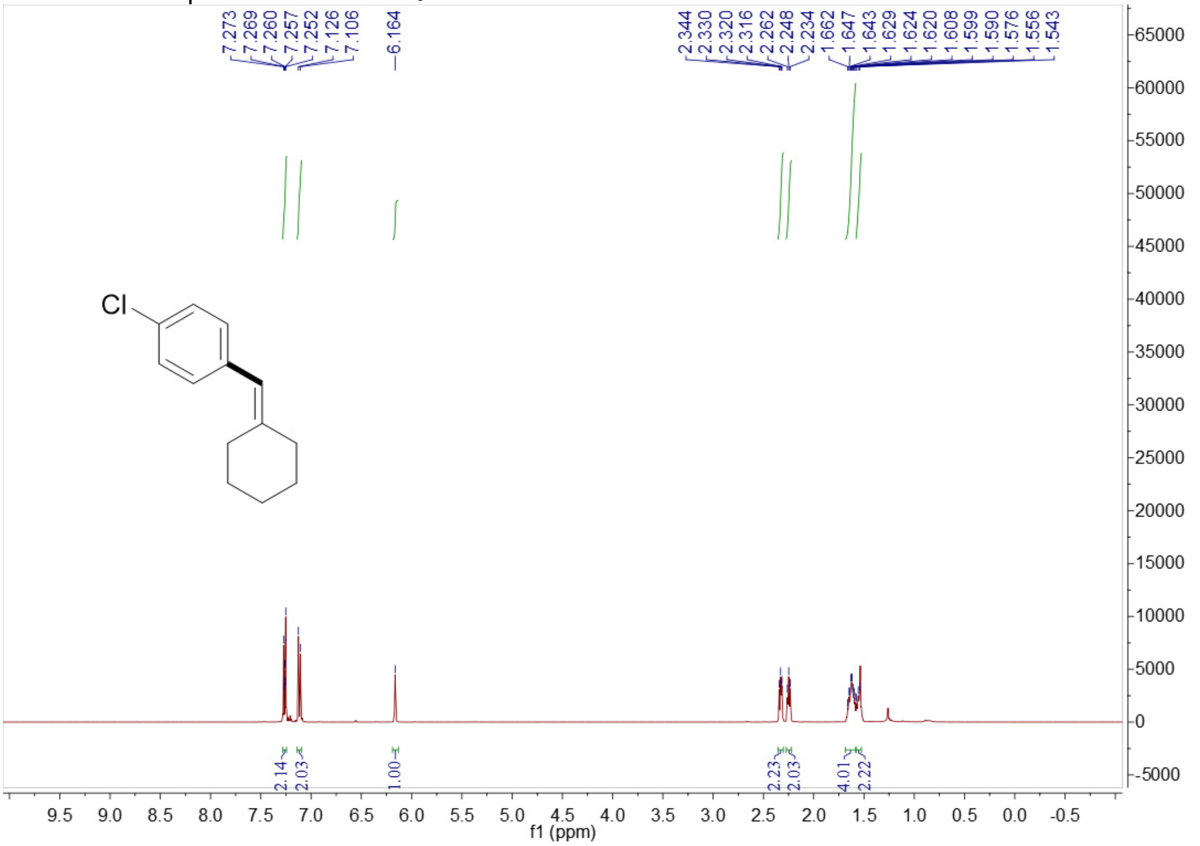

^{13}C NMR of compound **3du** in $CDCl_3$

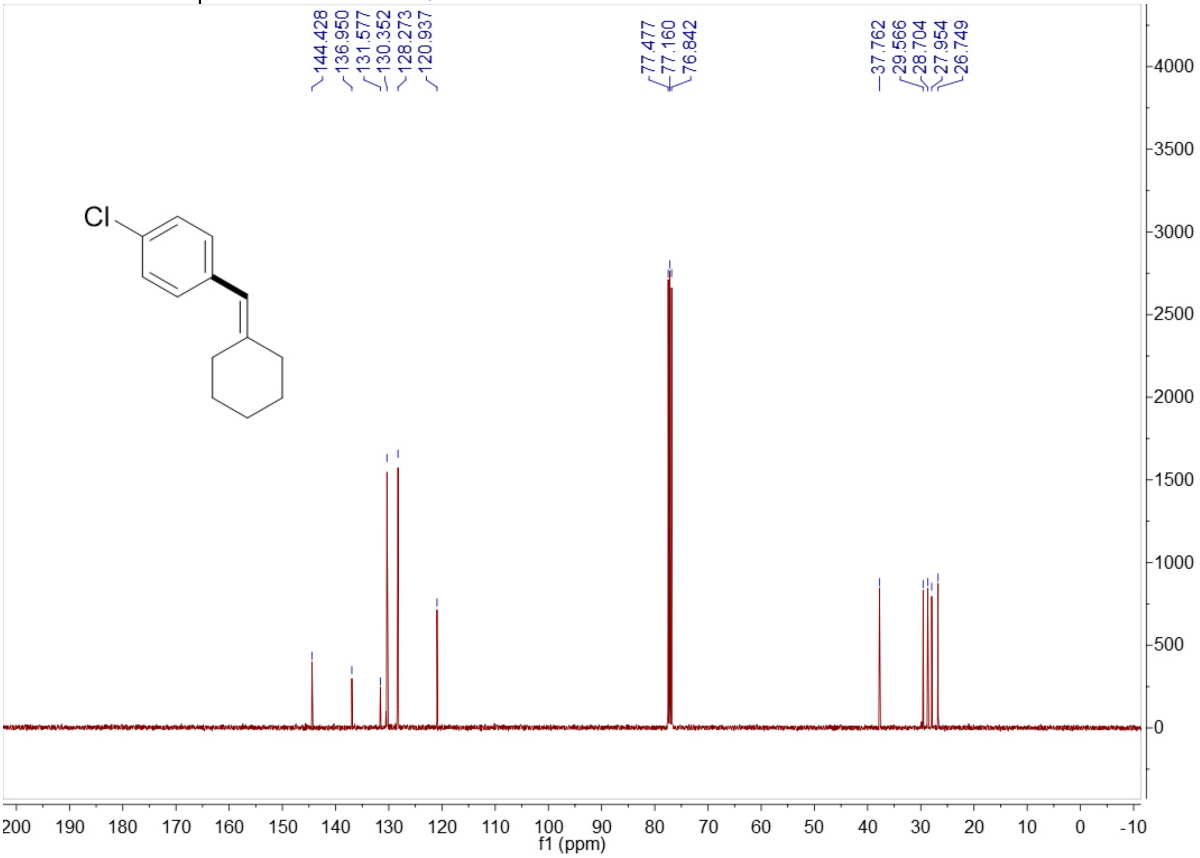

^{1}H NMR of compound **3uv** in $CDCl_3$

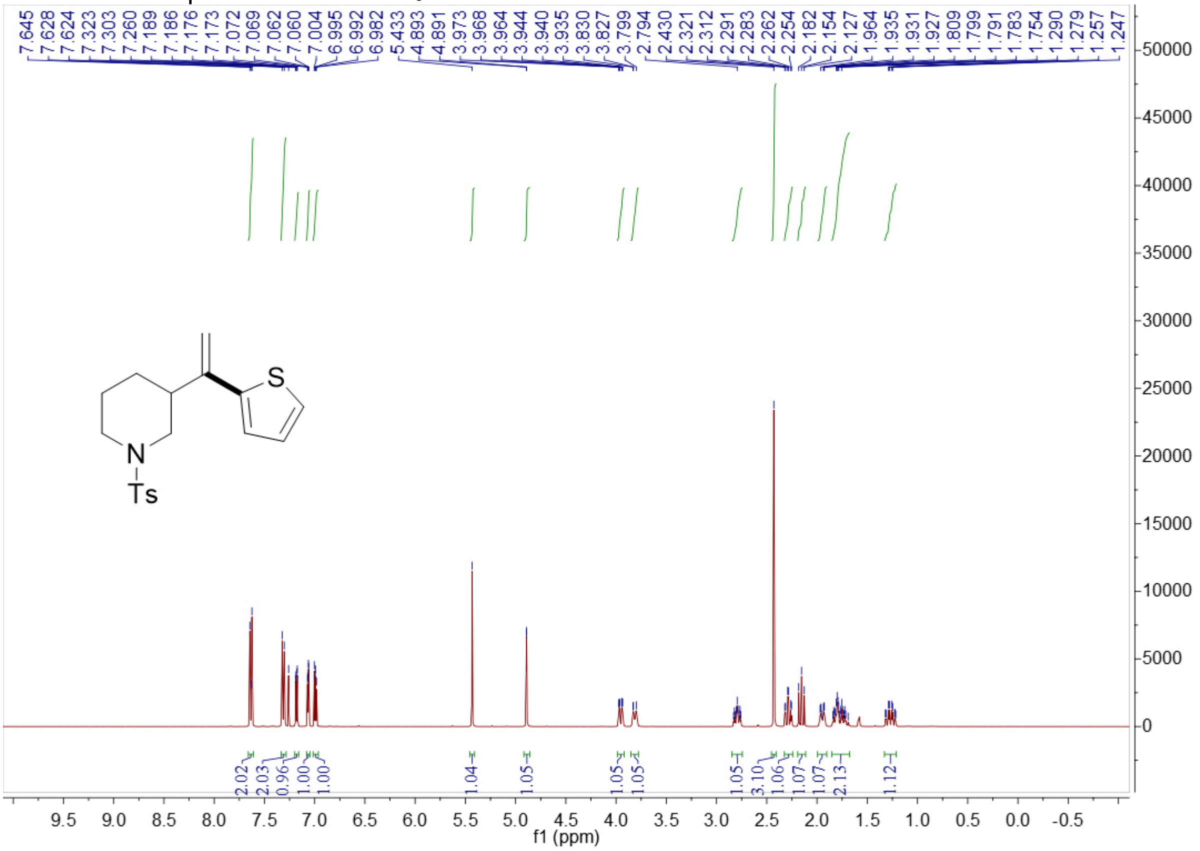

^{13}C NMR of compound **3uv** in $CDCl_3$

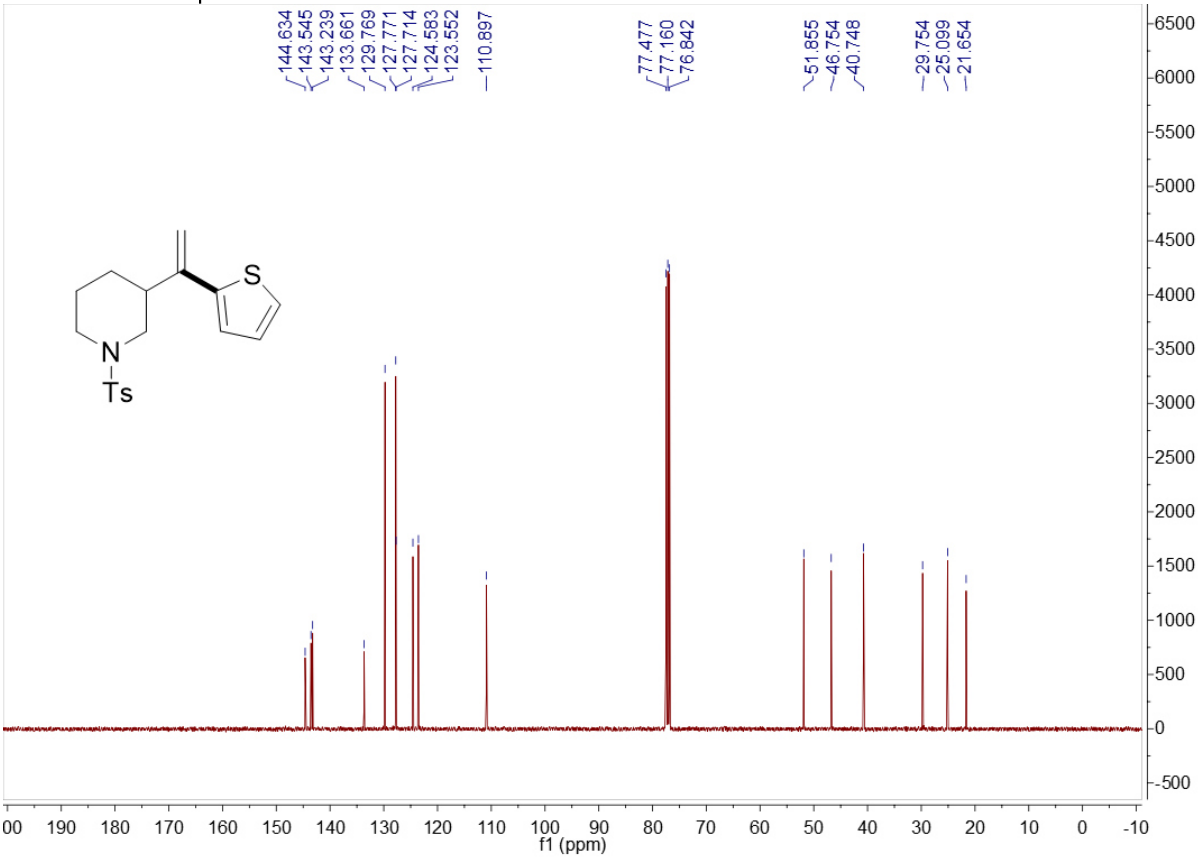

^1H NMR of compound **3lw** in $CDCl_3$

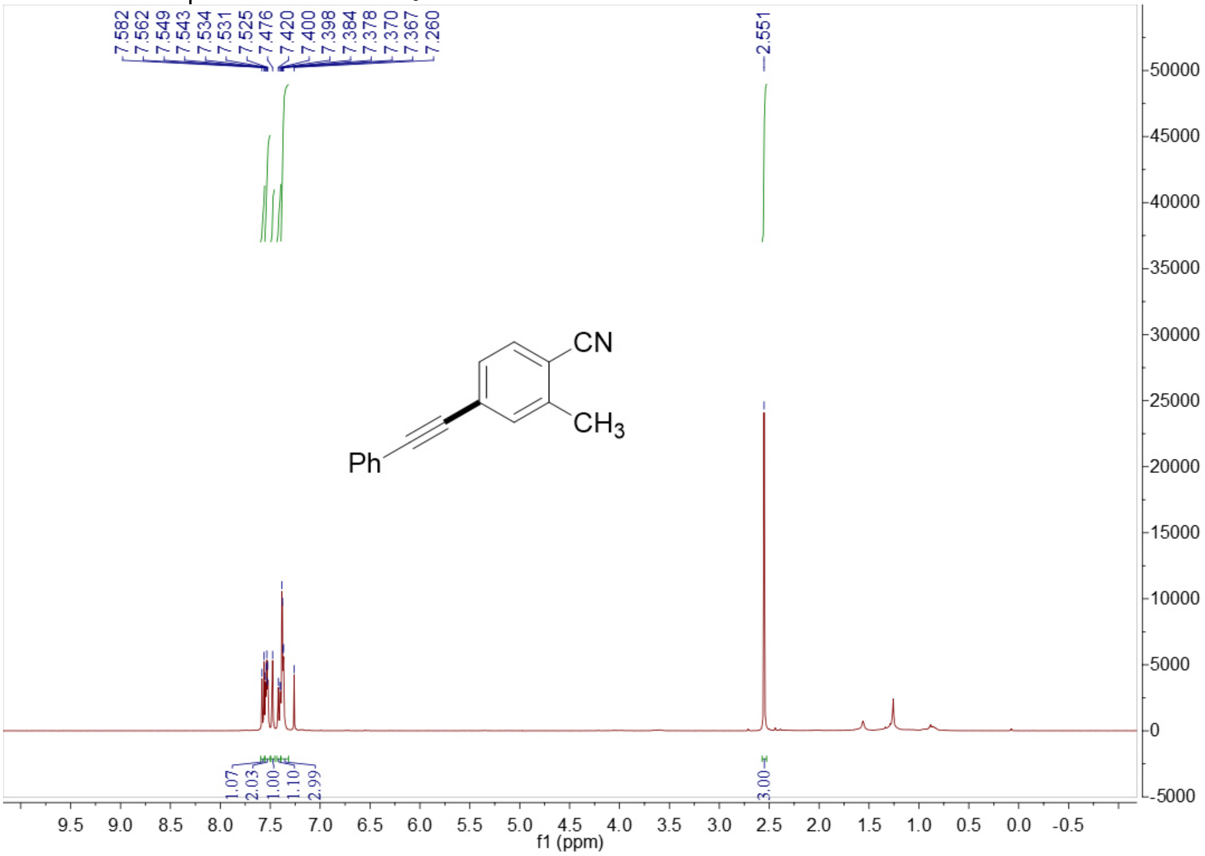

^{13}C NMR of compound **3lw** in $CDCl_3$

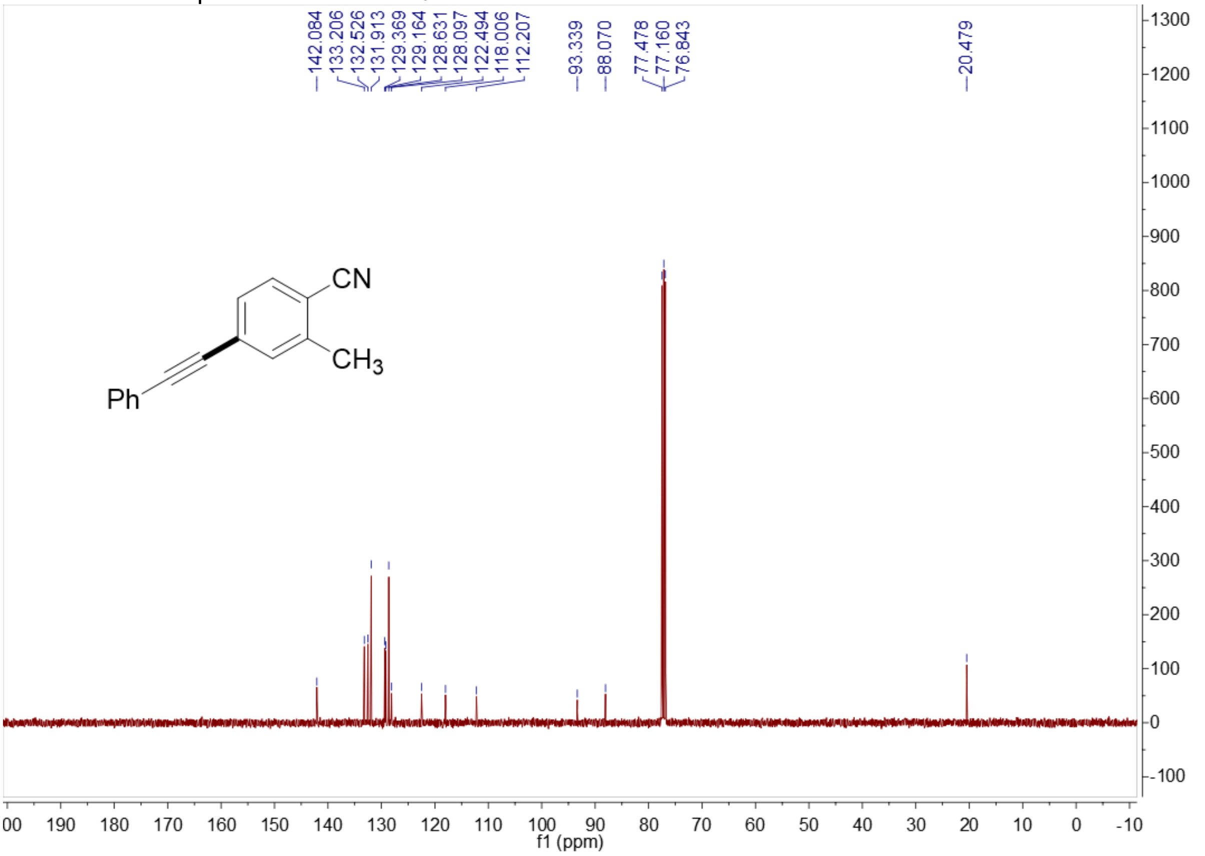

^{1}H NMR of compound **3fw** in $CDCl_3$

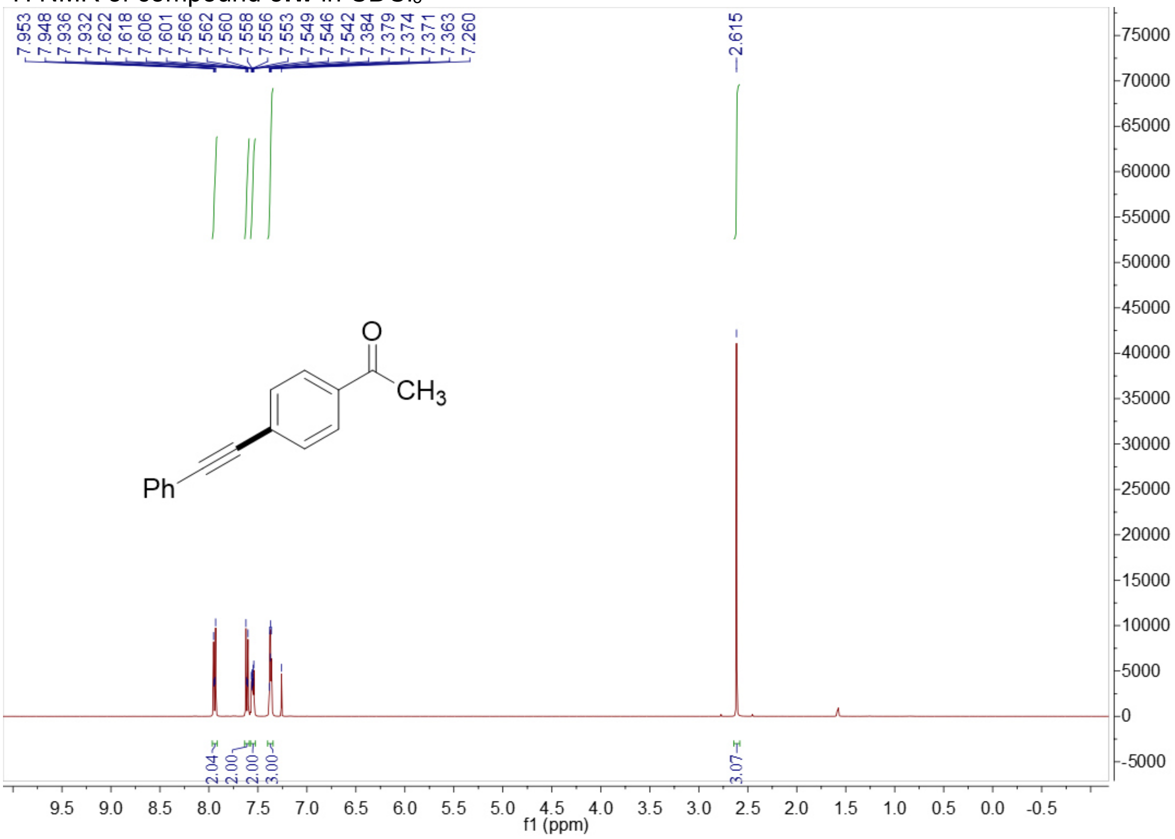

^{13}C NMR of compound **3fw** in $CDCl_3$

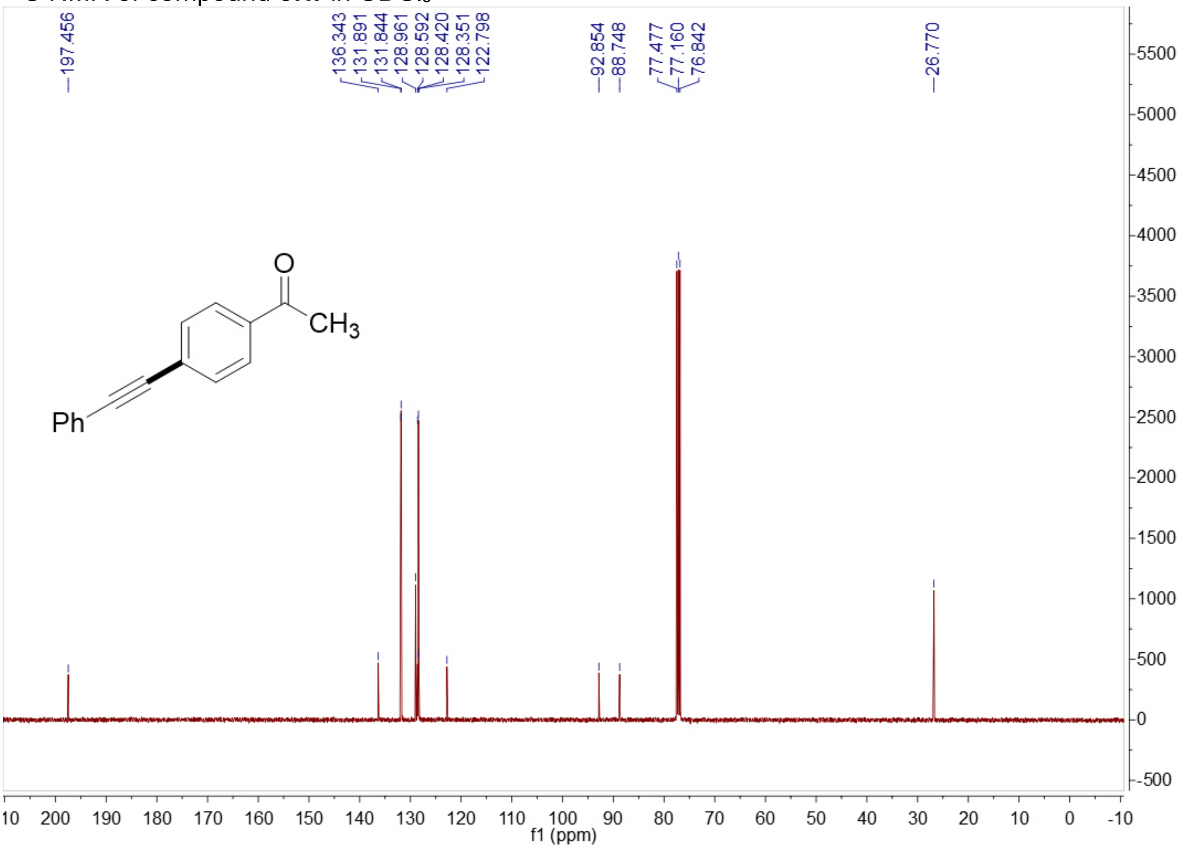

^{1}H NMR of compound **3dw** in $CDCl_3$

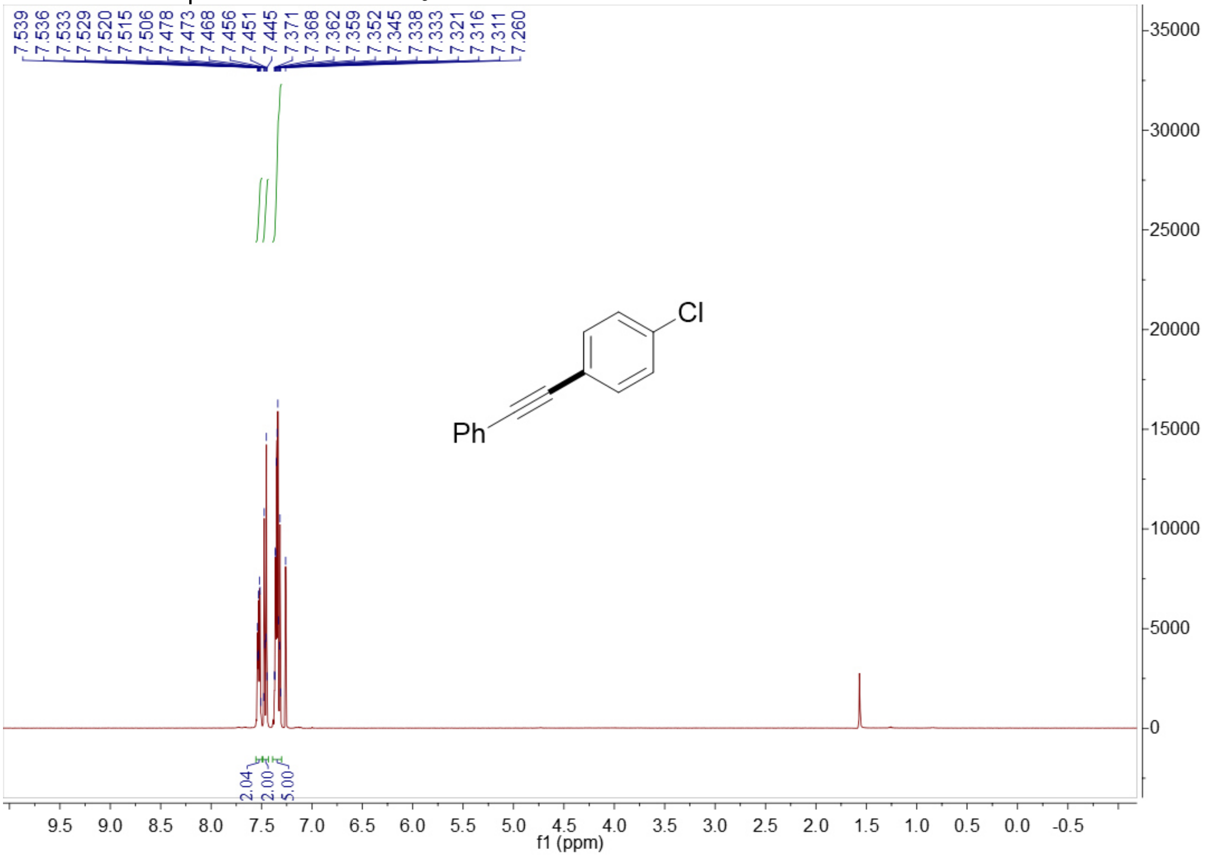

^{13}C NMR of compound **3dw** in $CDCl_3$

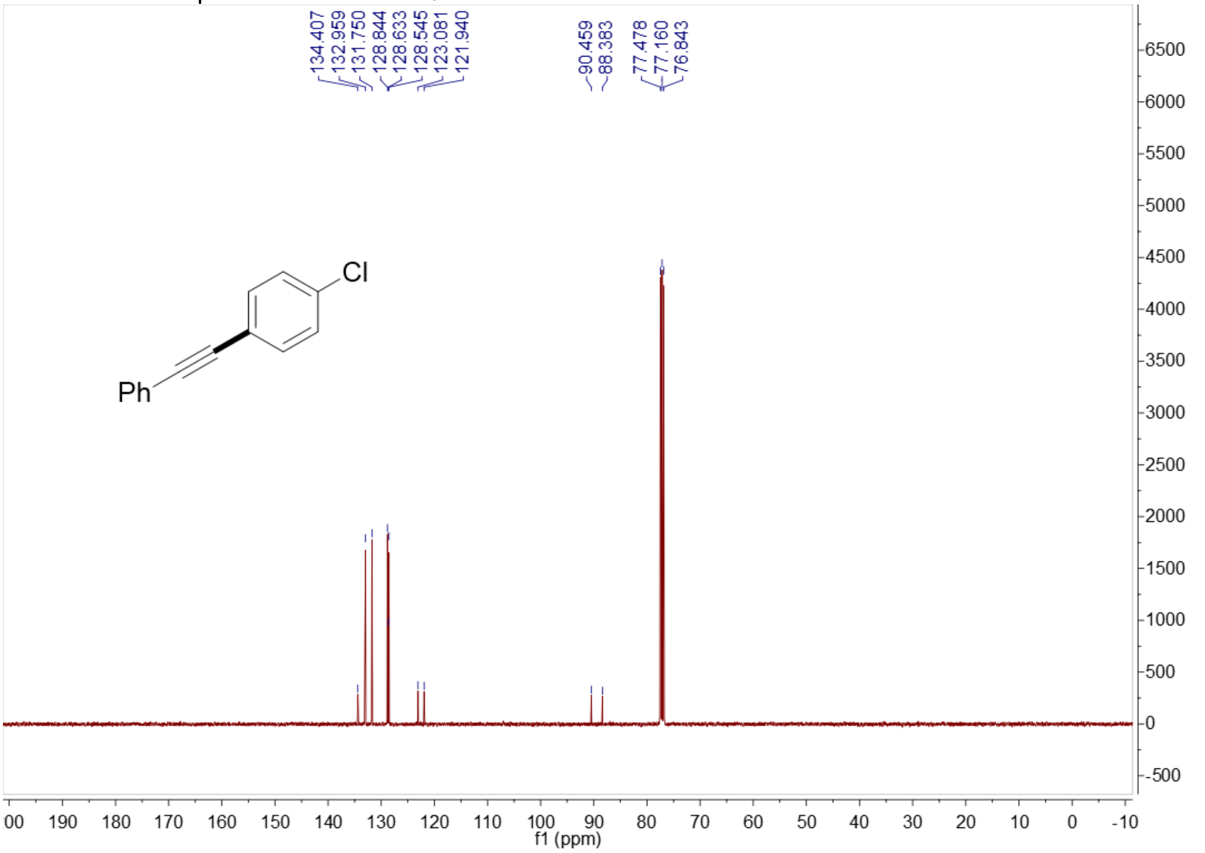

^{1}H NMR of compound **3aew** in $CDCl_3$

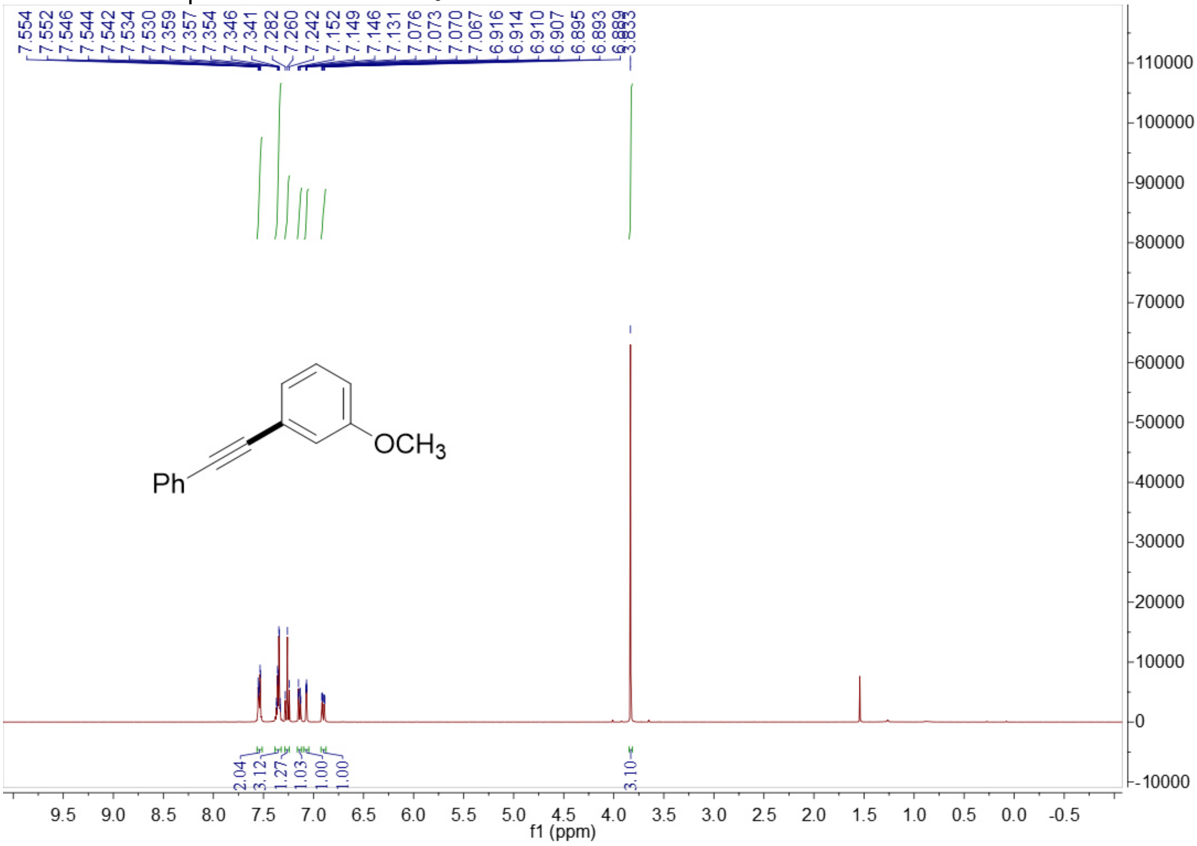

^{13}C NMR of compound **3aew** in $CDCl_3$

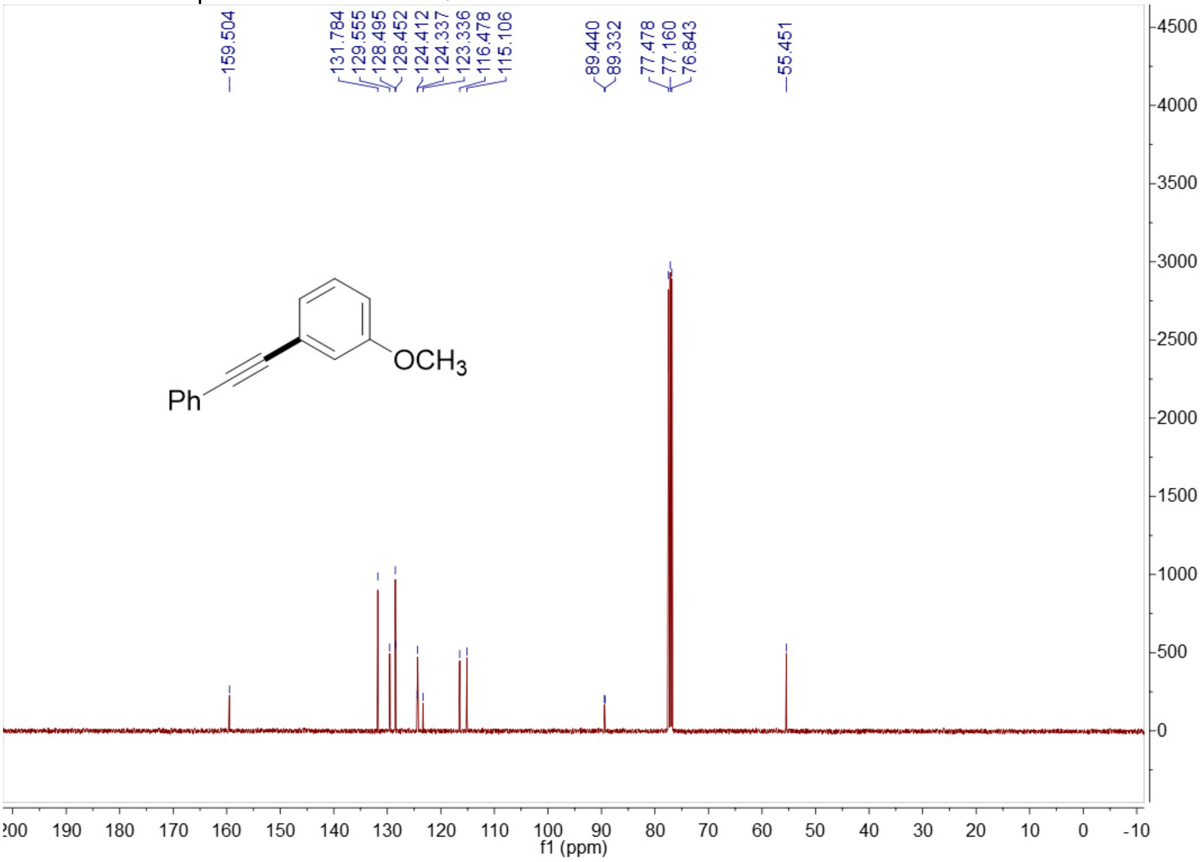

^{1}H NMR of compound **3fx** in $CDCl_3$

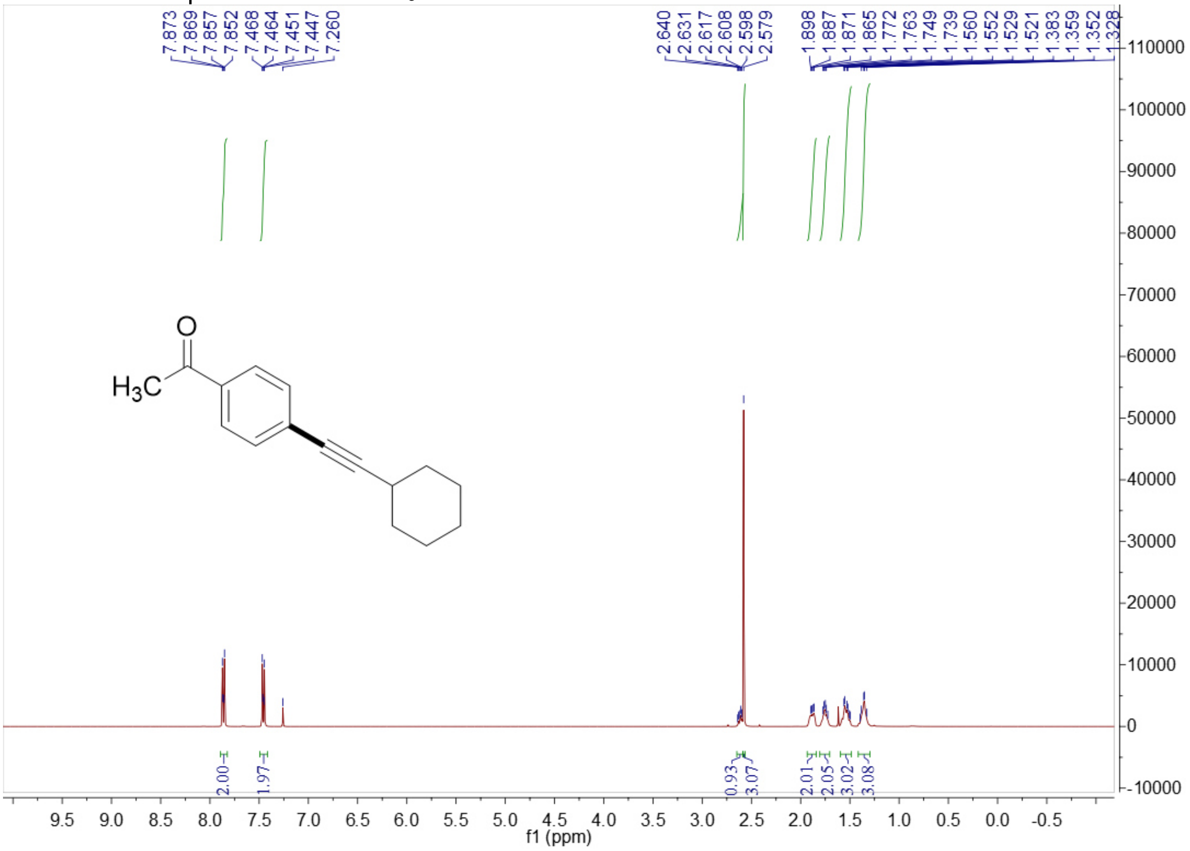

^{13}C NMR of compound **3fx** in $CDCl_3$

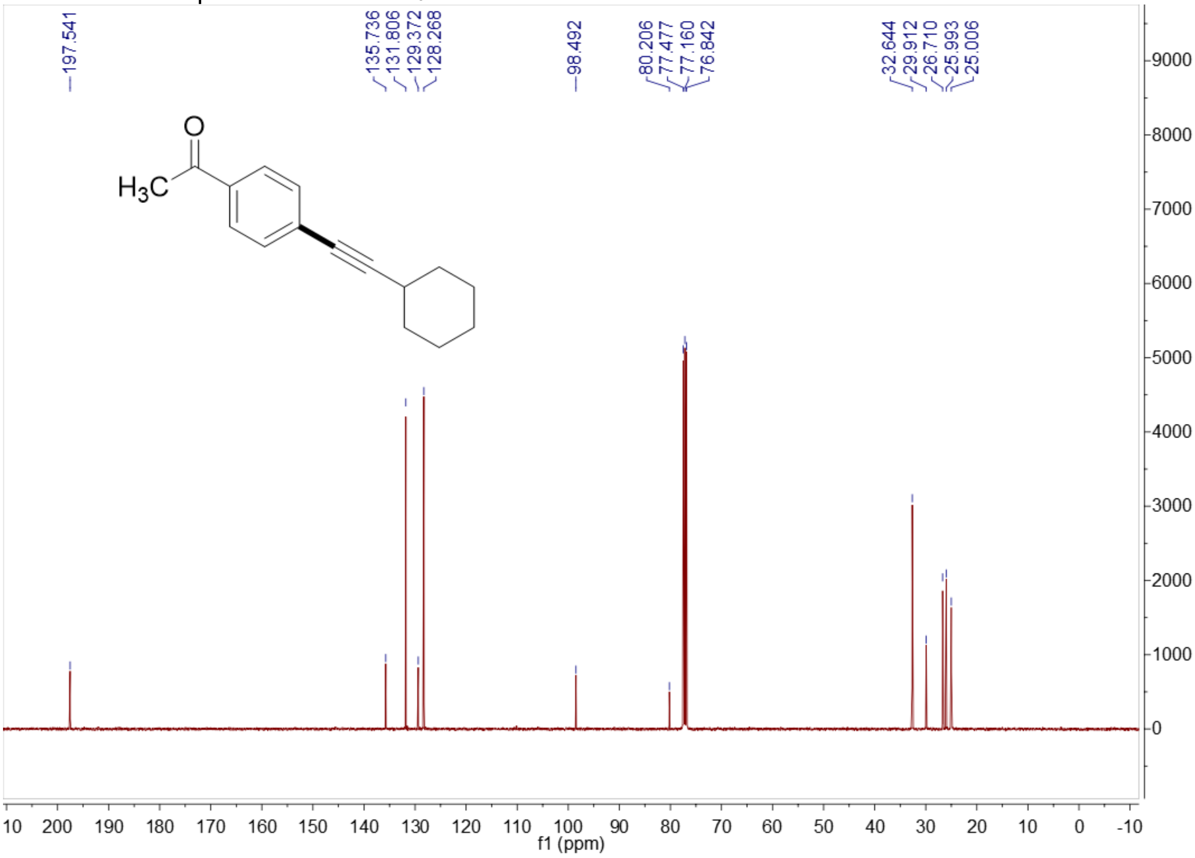

^{1}H NMR of compound **3ly** in $CDCl_3$

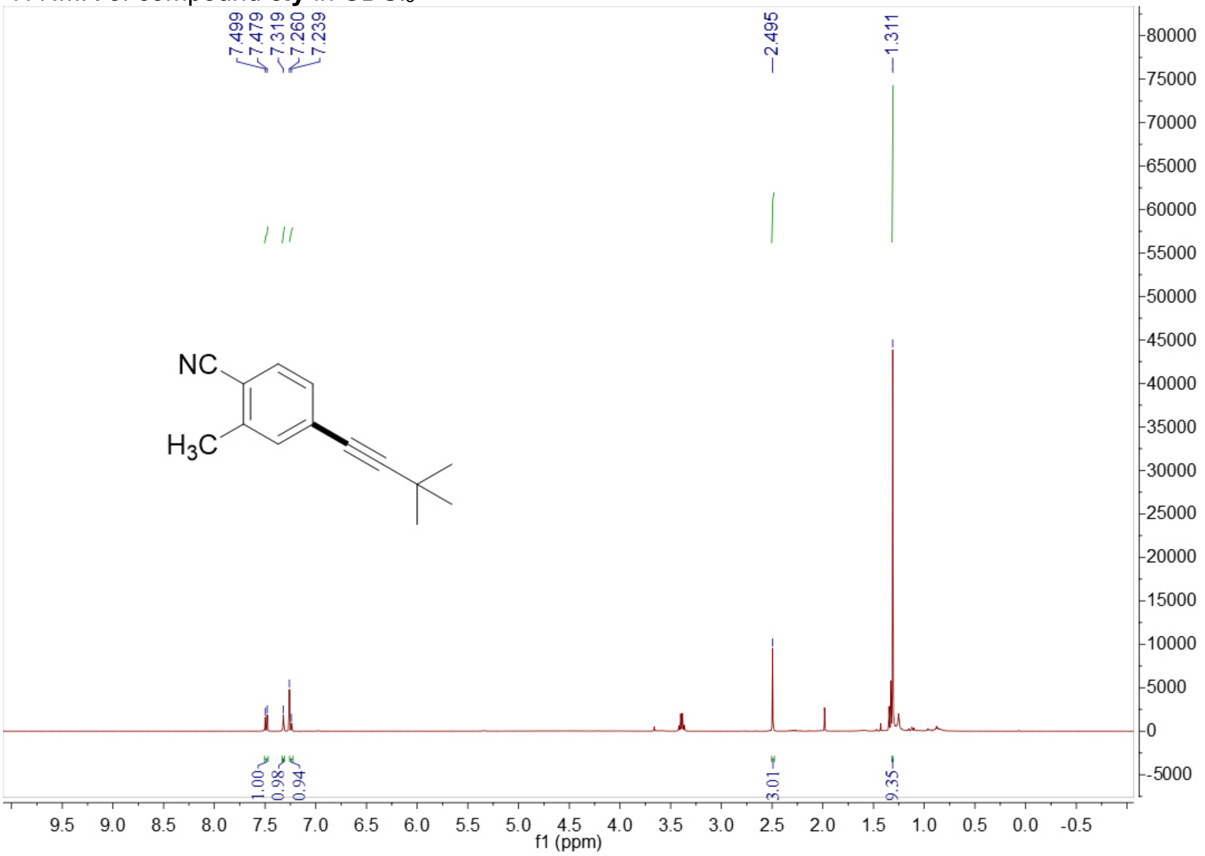

^{13}C NMR of compound **3ly** in $CDCl_3$

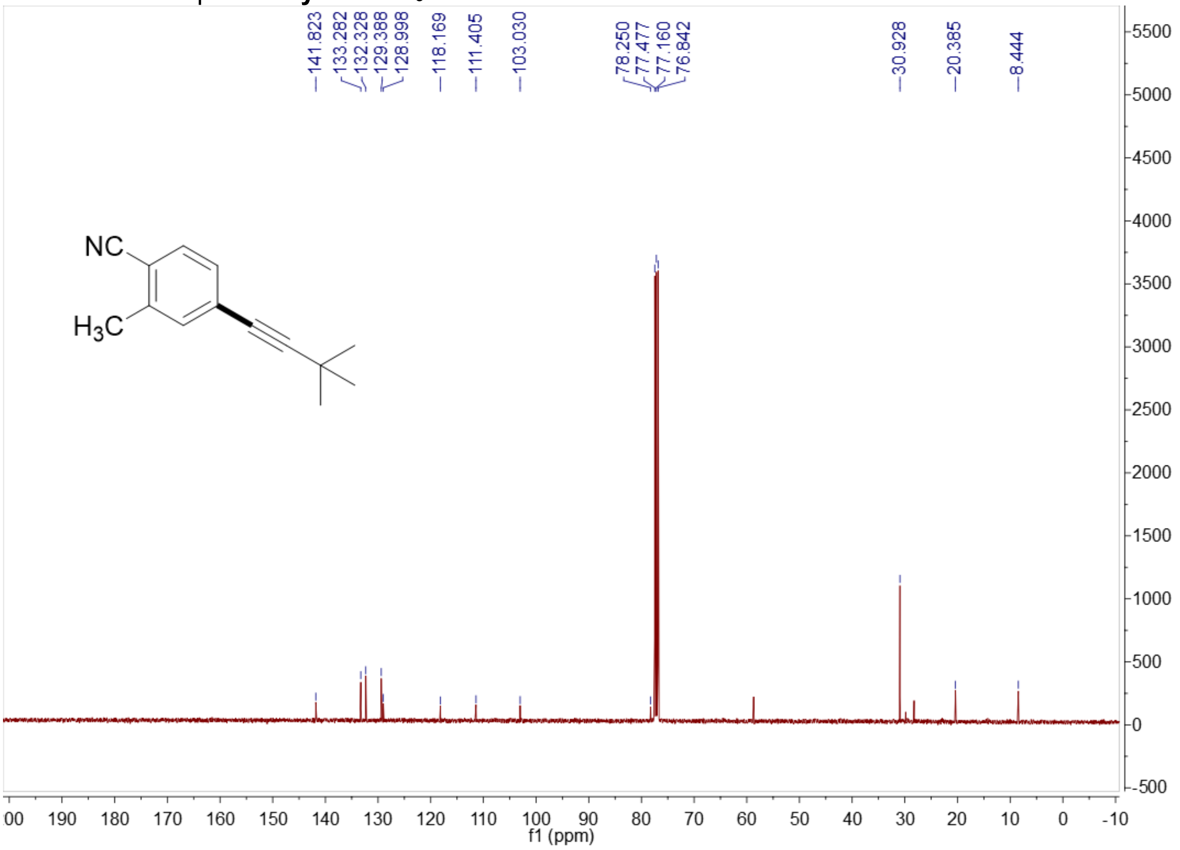

^{1}H NMR of compound **3afa** in $CDCl_3$

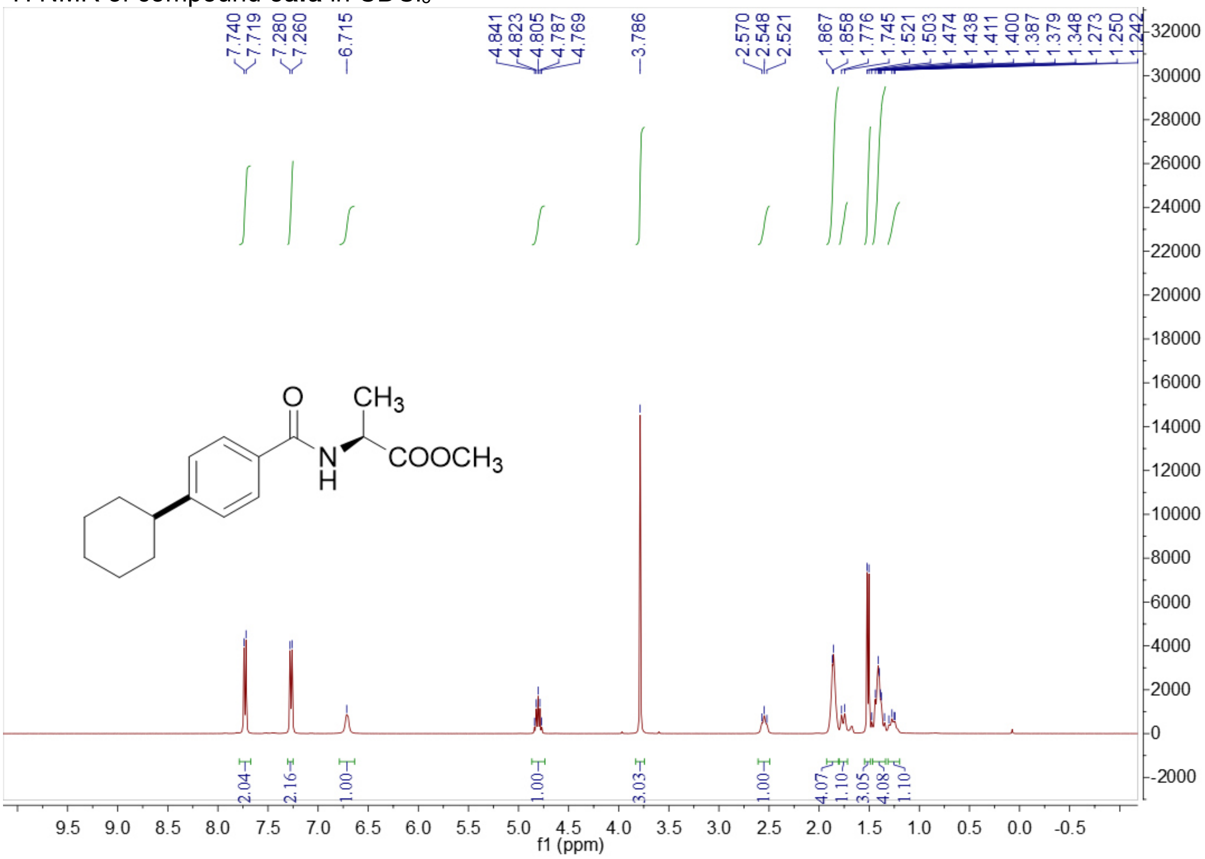

^{13}C NMR of compound **3afa** in $CDCl_3$

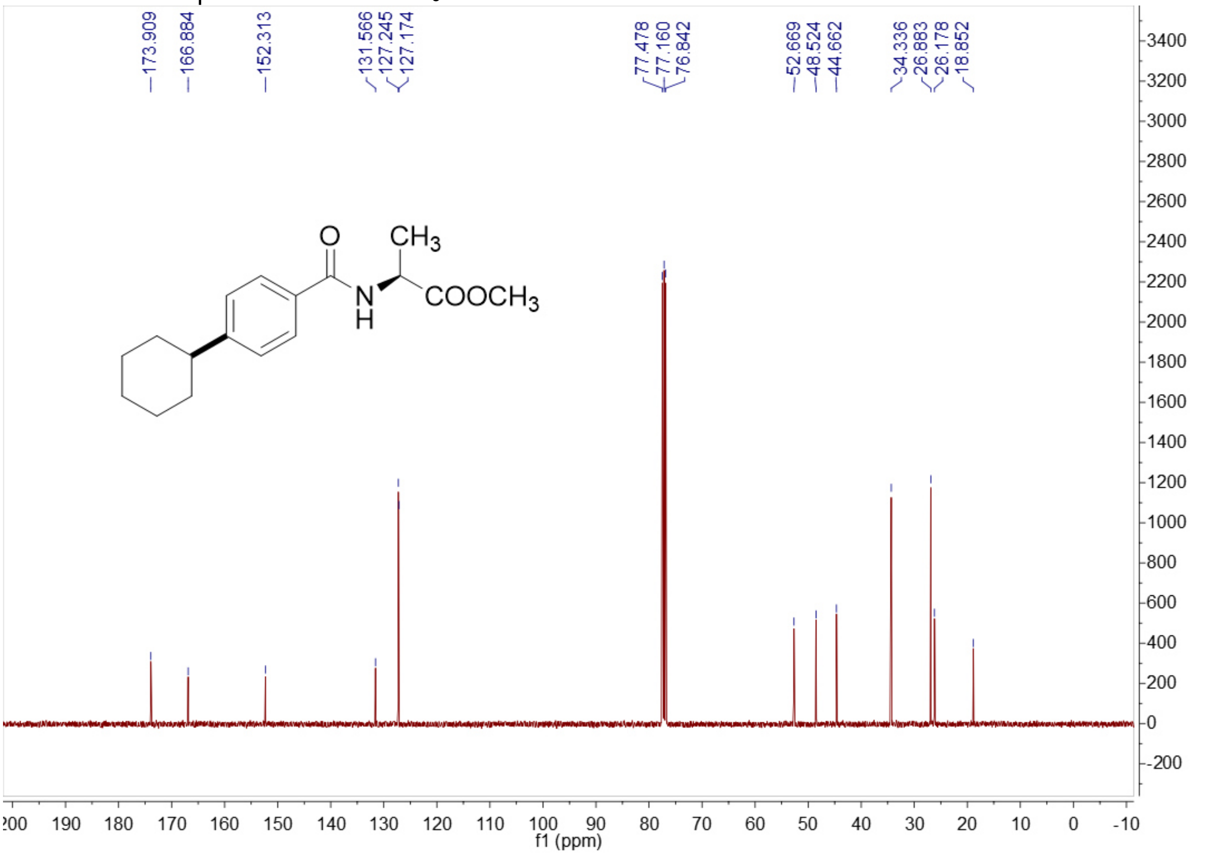

^{1}H NMR of compound **3aga** in $CDCl_3$

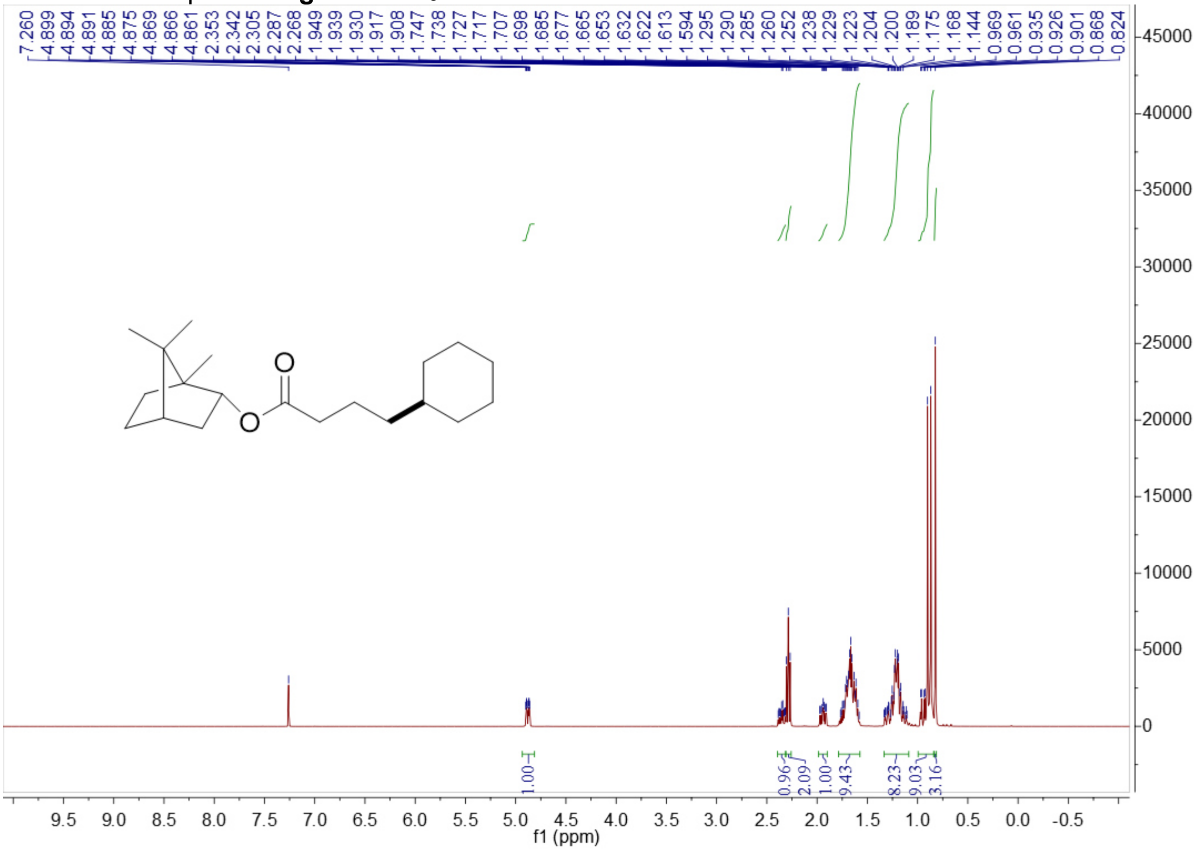

^{13}C NMR of compound **3aga** in $CDCl_3$

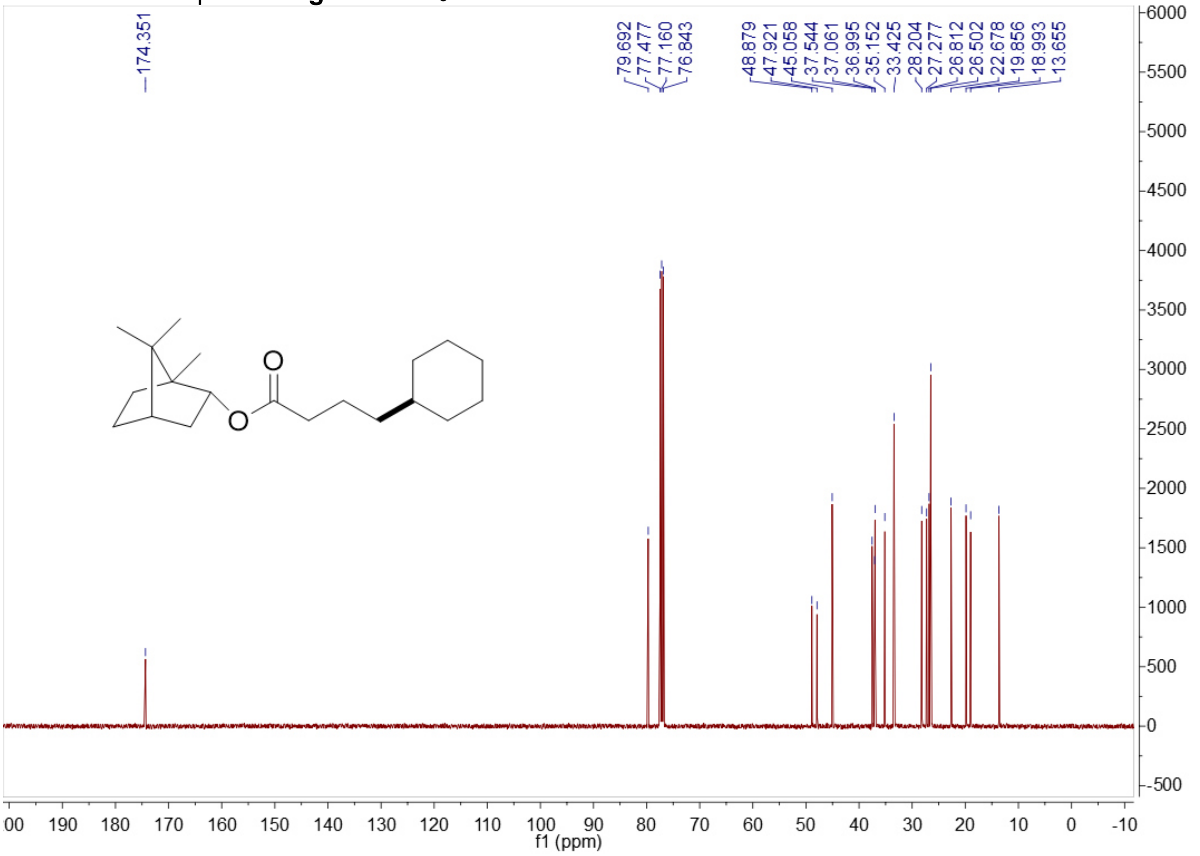

^{1}H NMR of compound **3aha** in $CDCl_3$

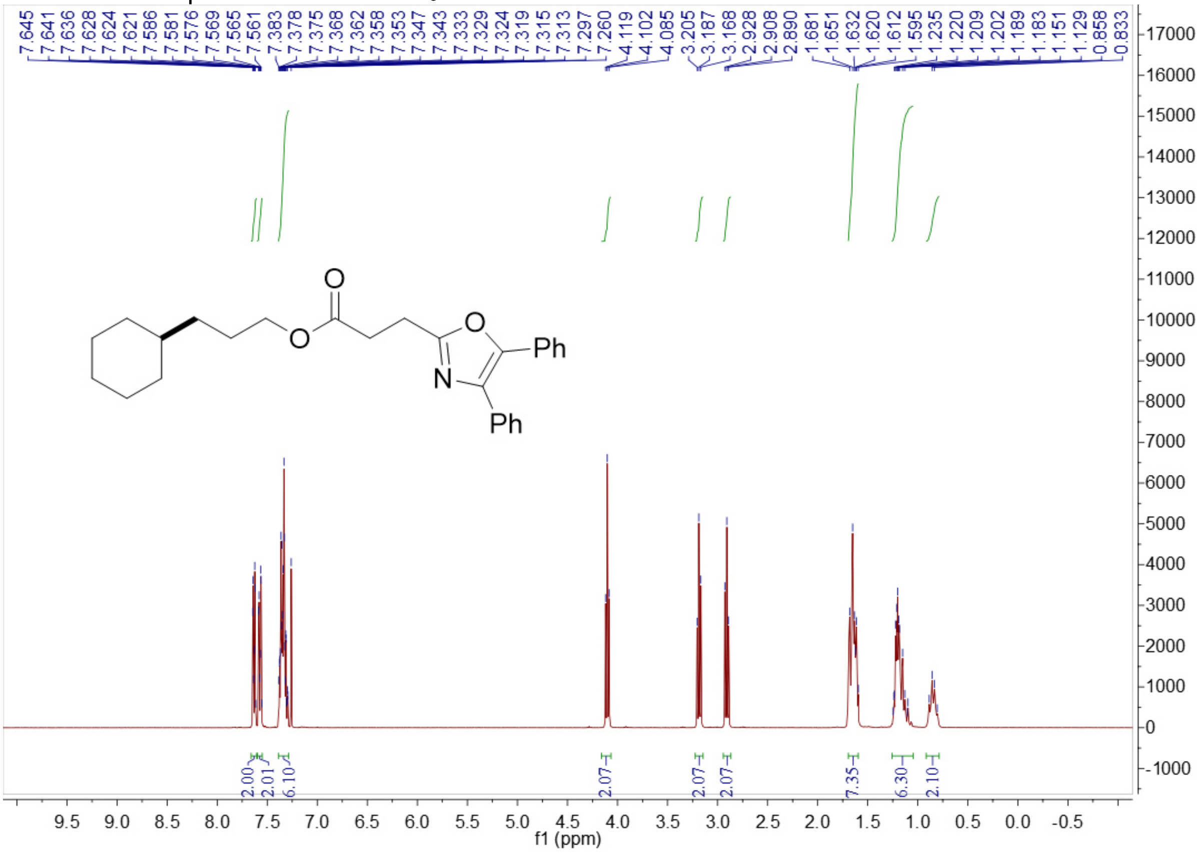

^{13}C NMR of compound **3aha** in $CDCl_3$

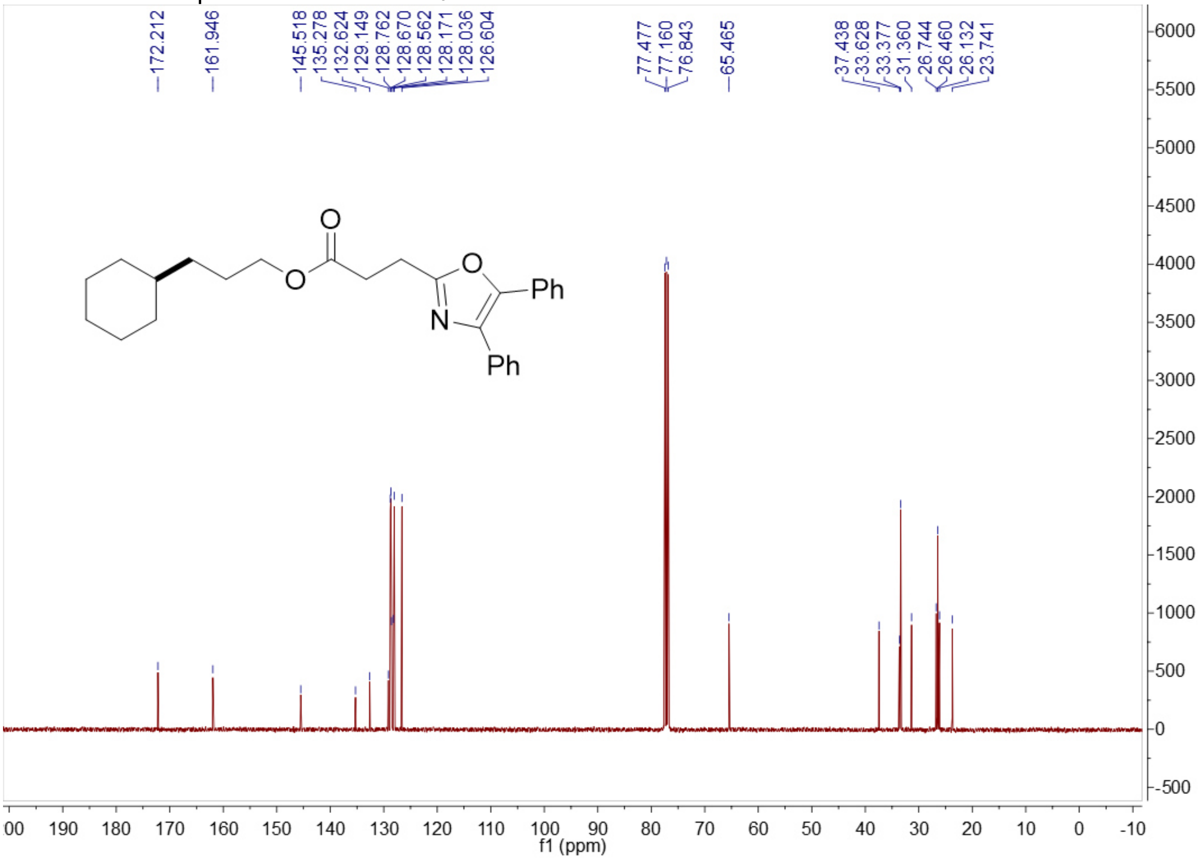

^{1}H NMR of compound **3aia** in $CDCl_3$

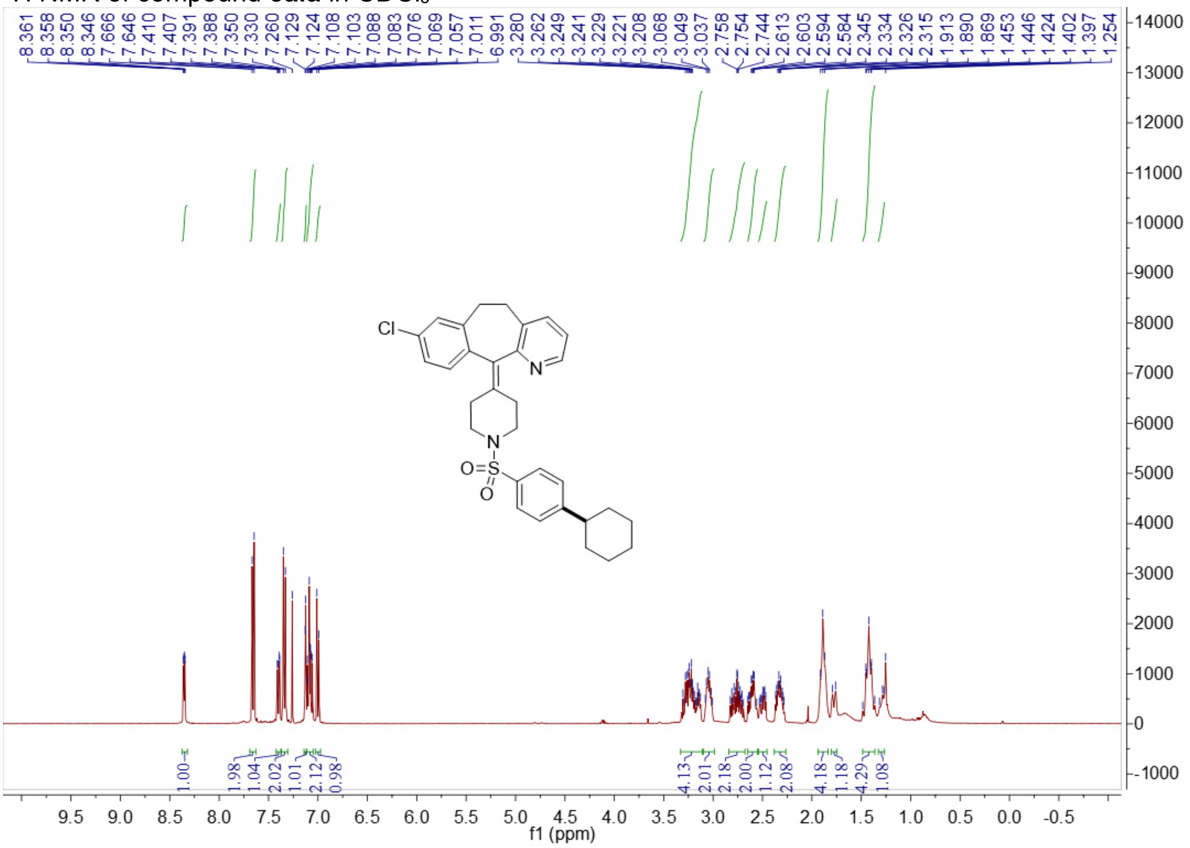

^{13}C NMR of compound **3aia** in $CDCl_3$

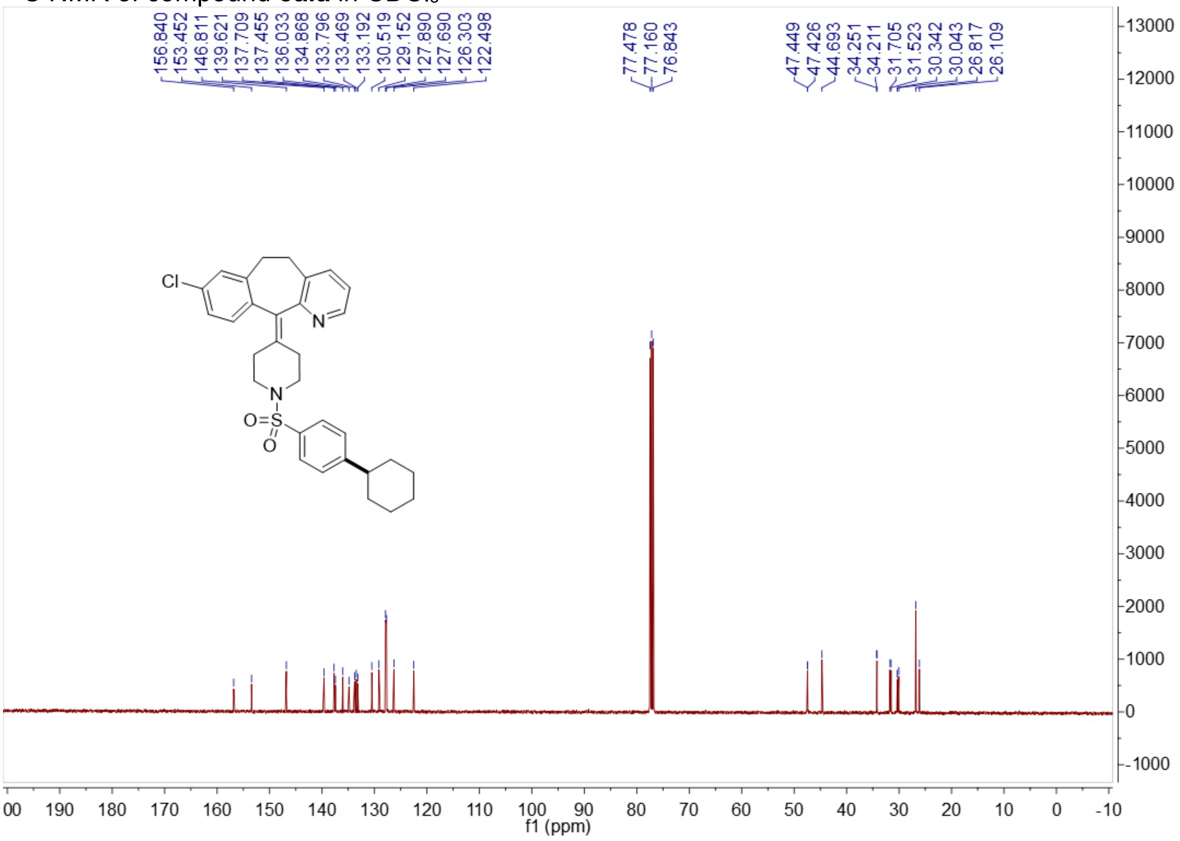

^{1}H NMR of compound **3lz** in $CDCl_3$

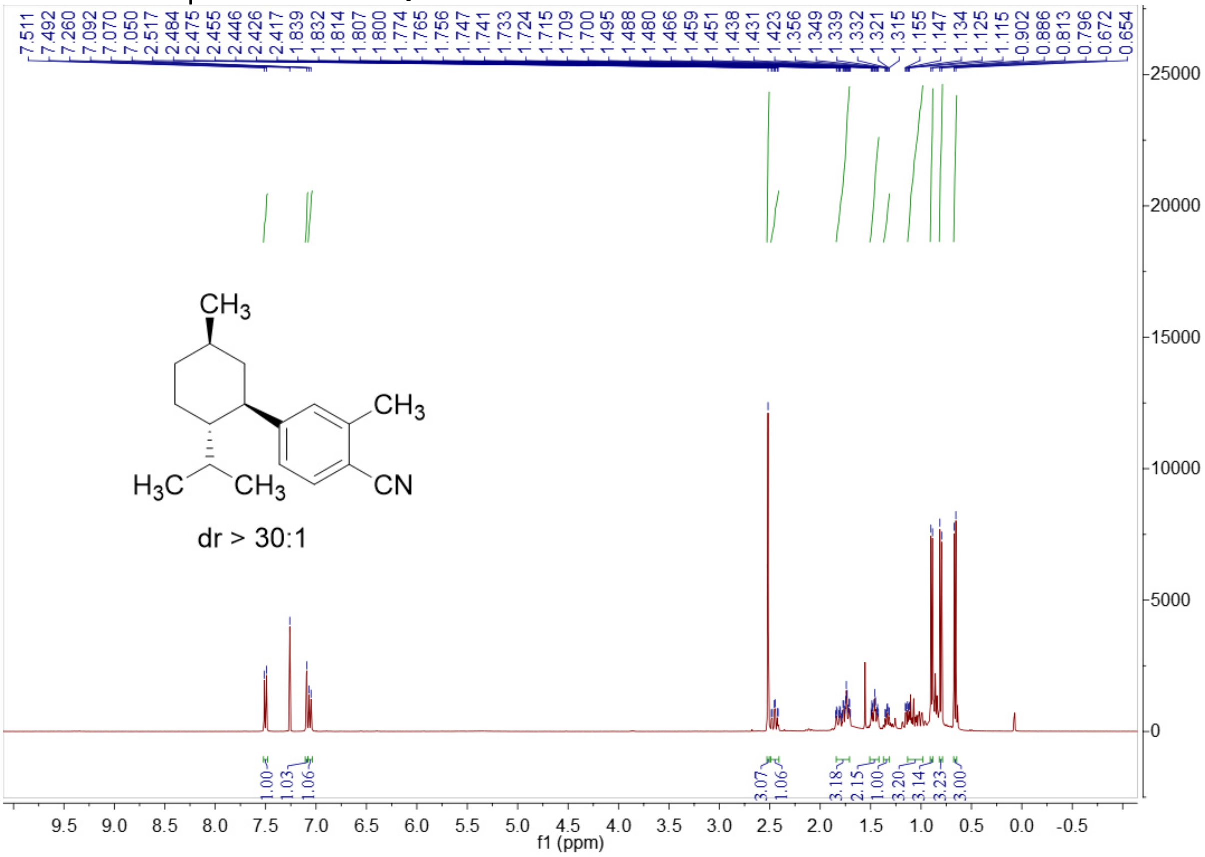

^{13}C NMR of compound **3lz** in $CDCl_3$

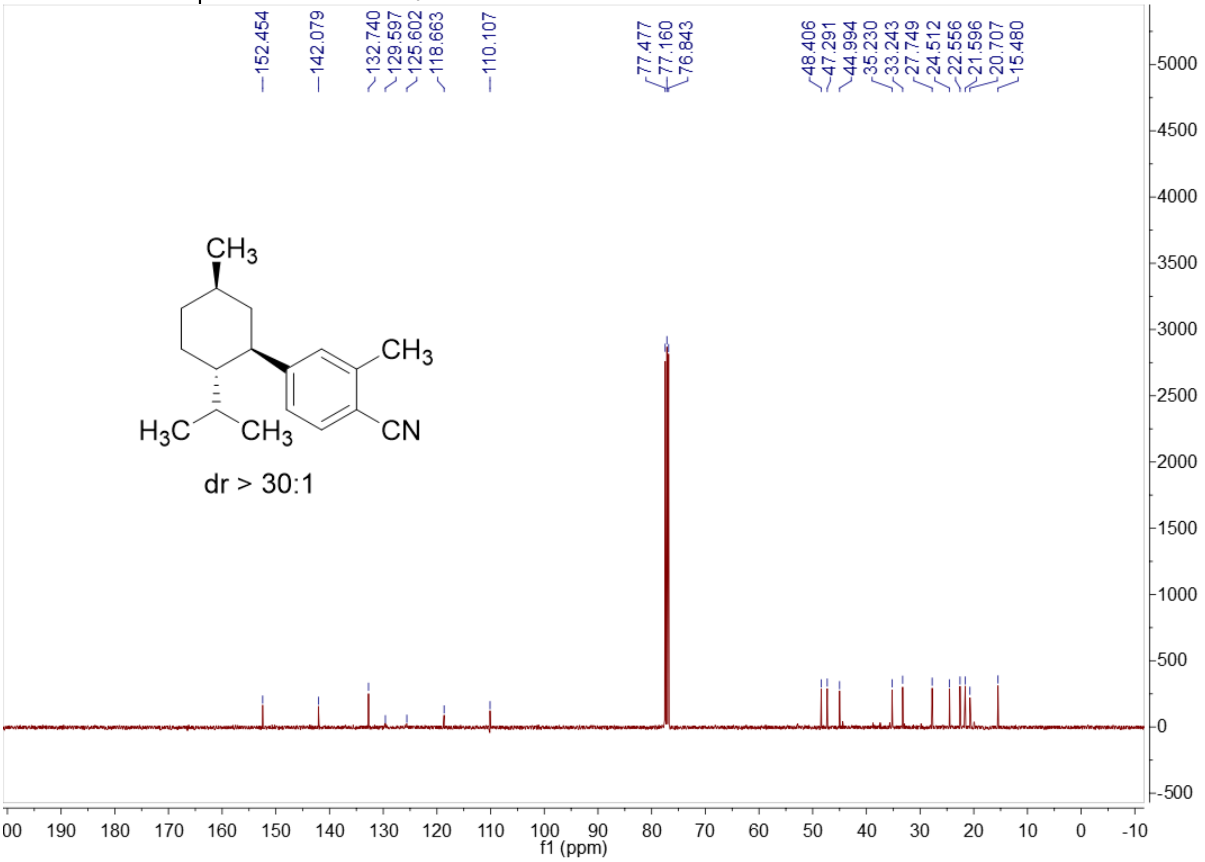

^{1}H NMR of compound **3aaz** in $CDCl_3$

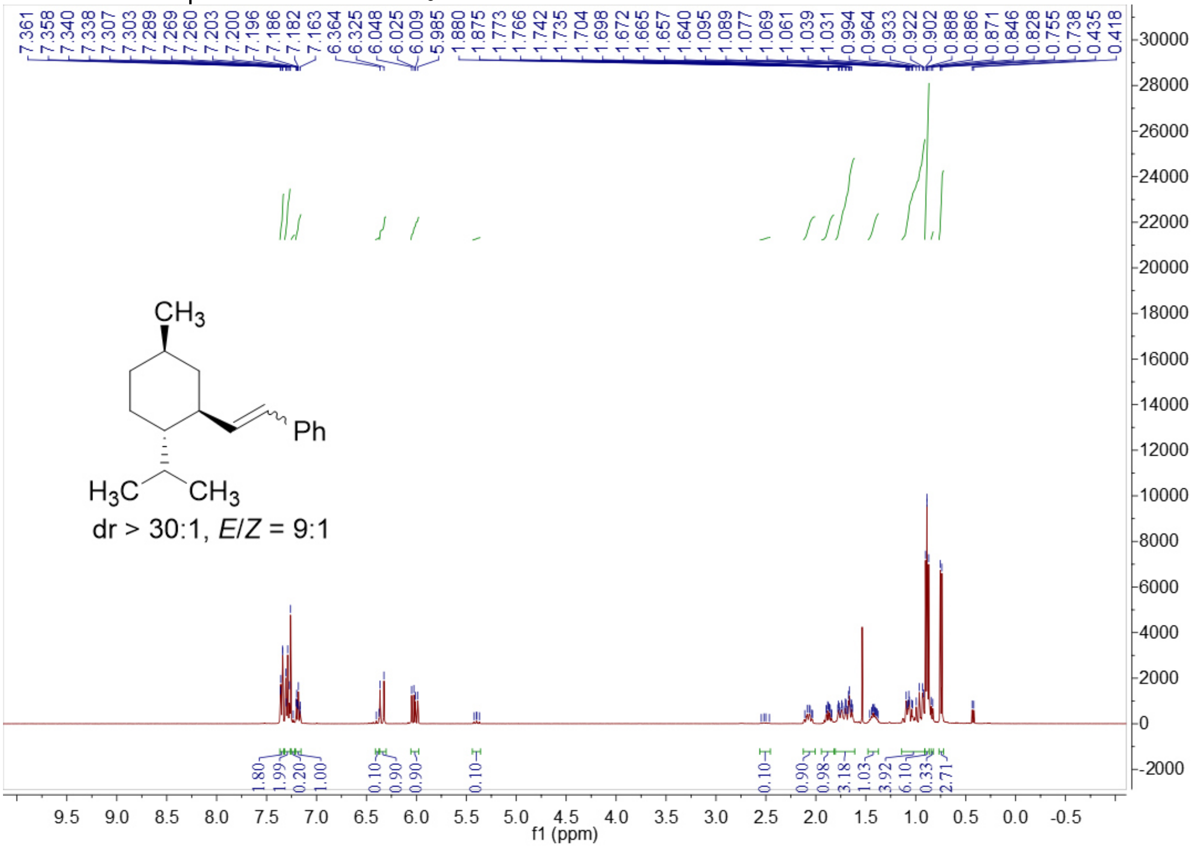

^{13}C NMR of compound **3aaz** in $CDCl_3$

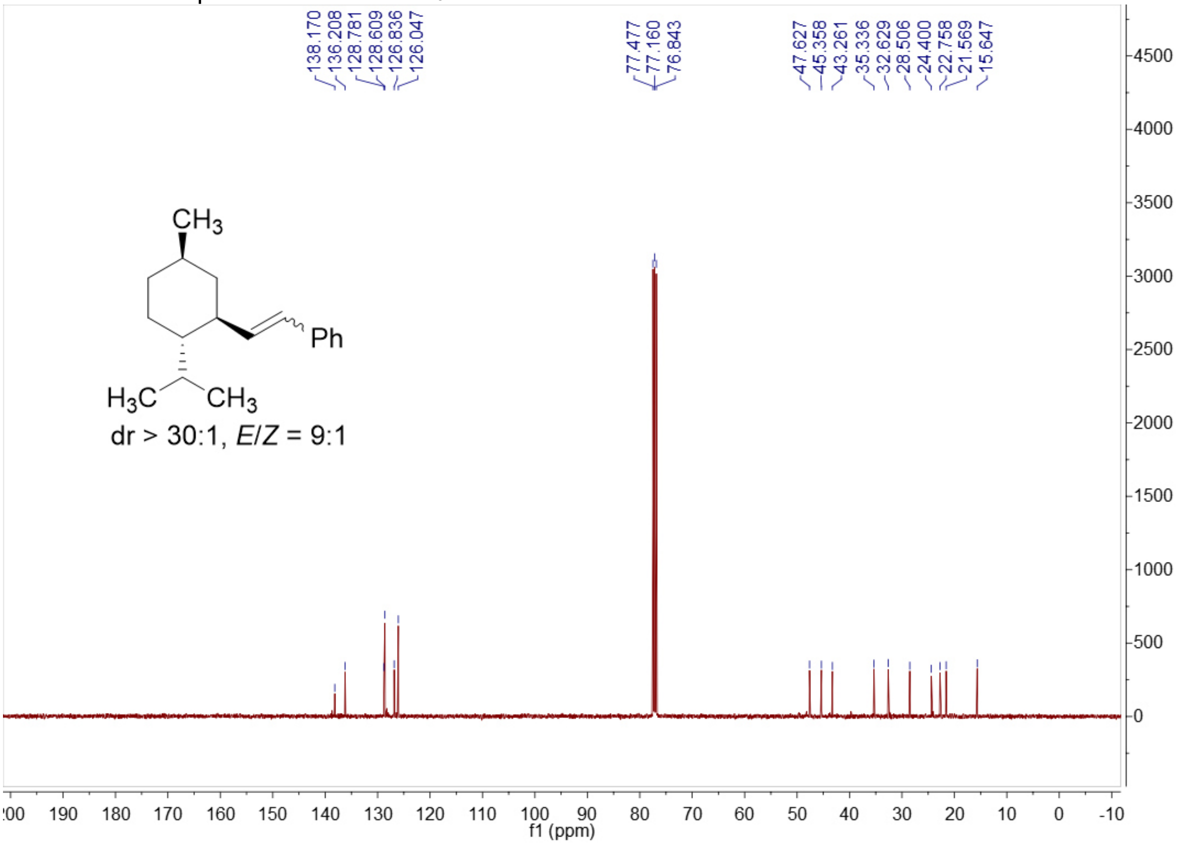

^{1}H NMR of compound **3aaaa** in $CDCl_3$

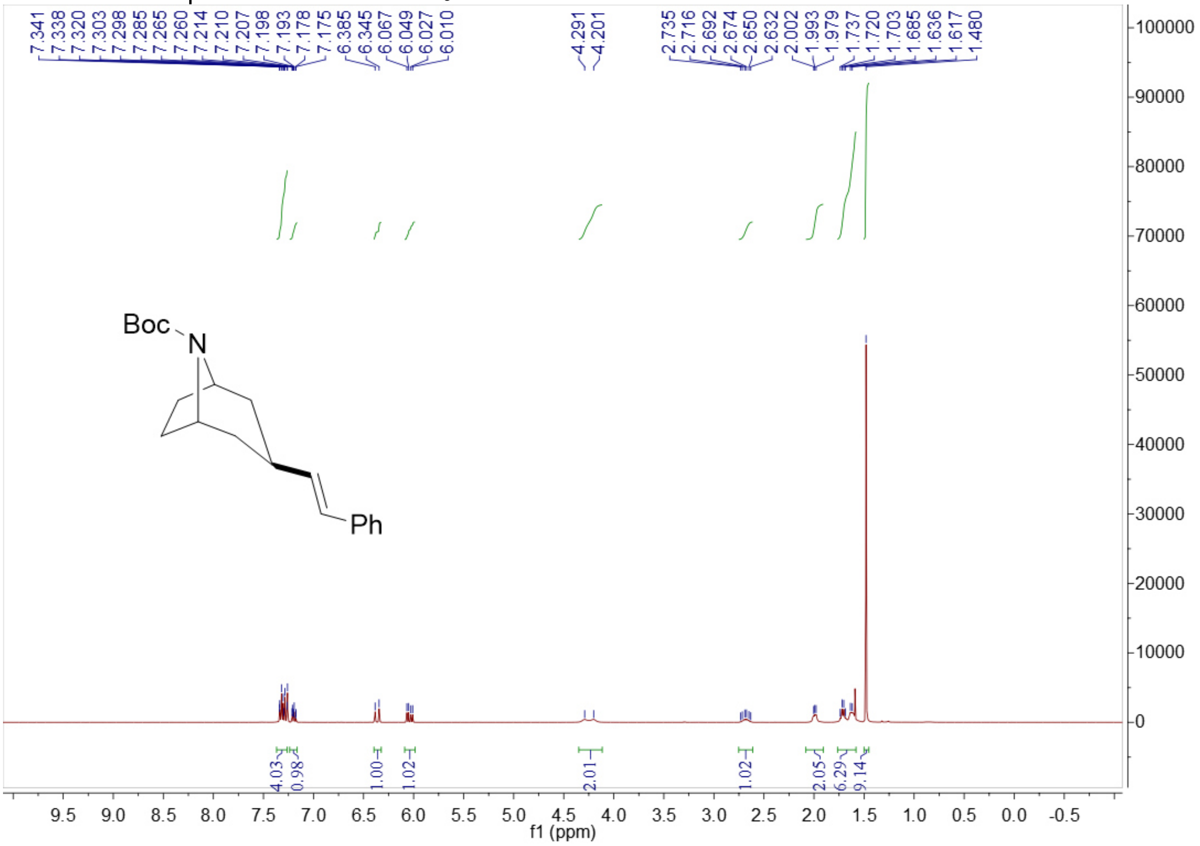

^{13}C NMR of compound **3aaaa** in $CDCl_3$

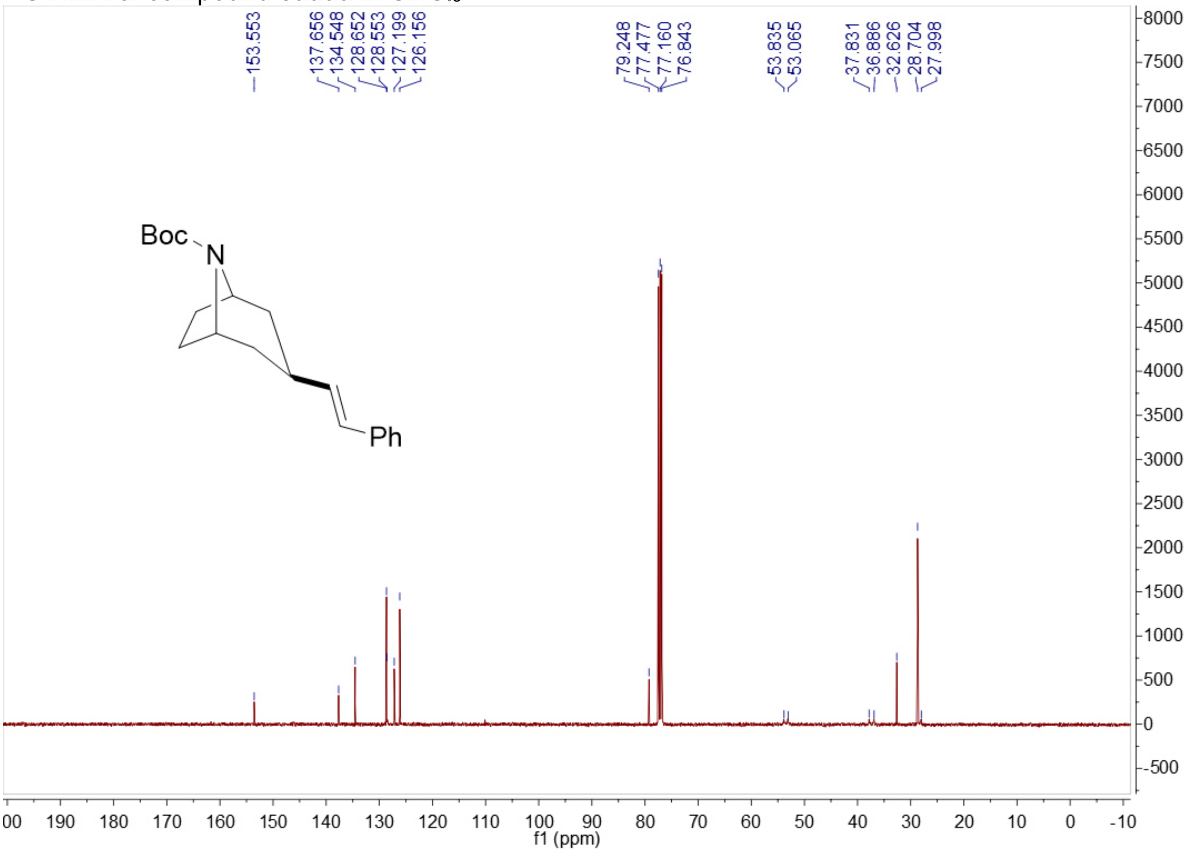

^{1}H NMR of compound **3lab** in $CDCl_3$

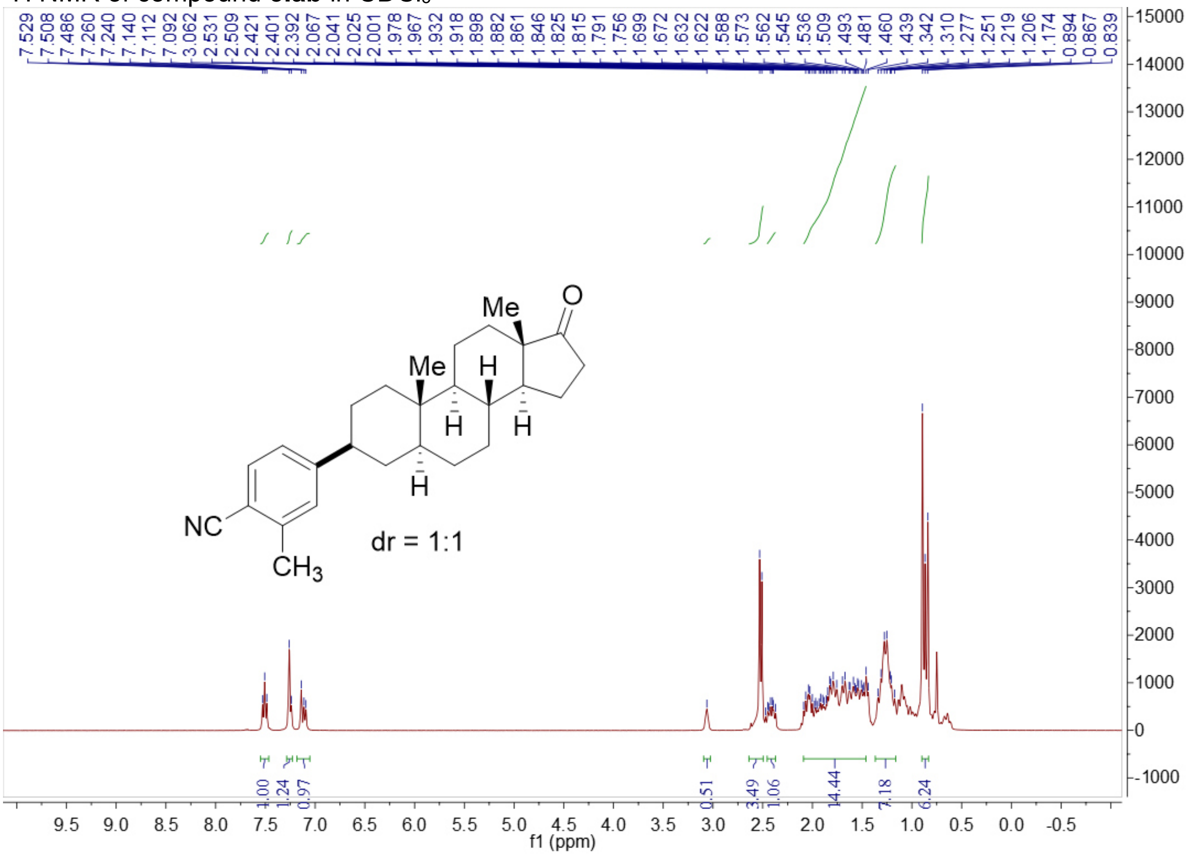

^{13}C NMR of compound **3lab** in $CDCl_3$

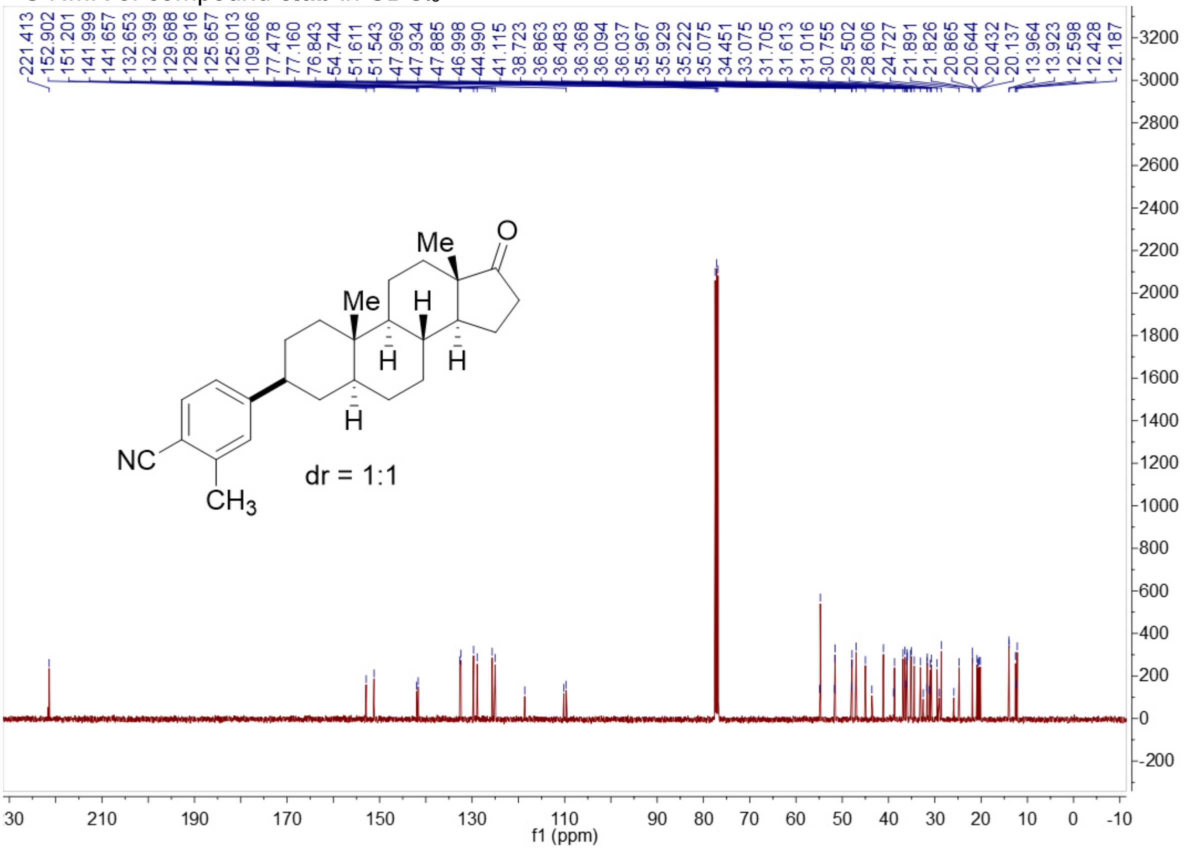

^{1}H NMR of compound **3jac** in $CDCl_3$

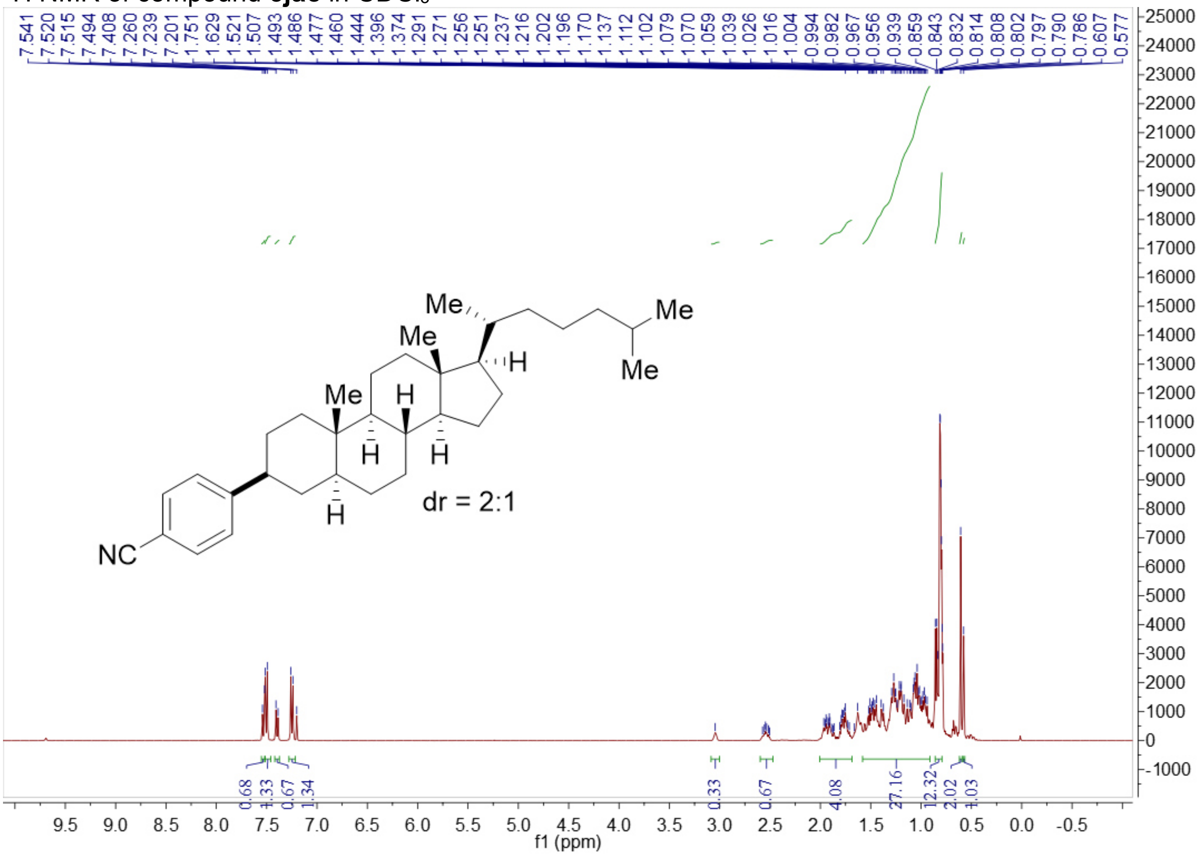

^{13}C NMR of compound **3jac** in $CDCl_3$

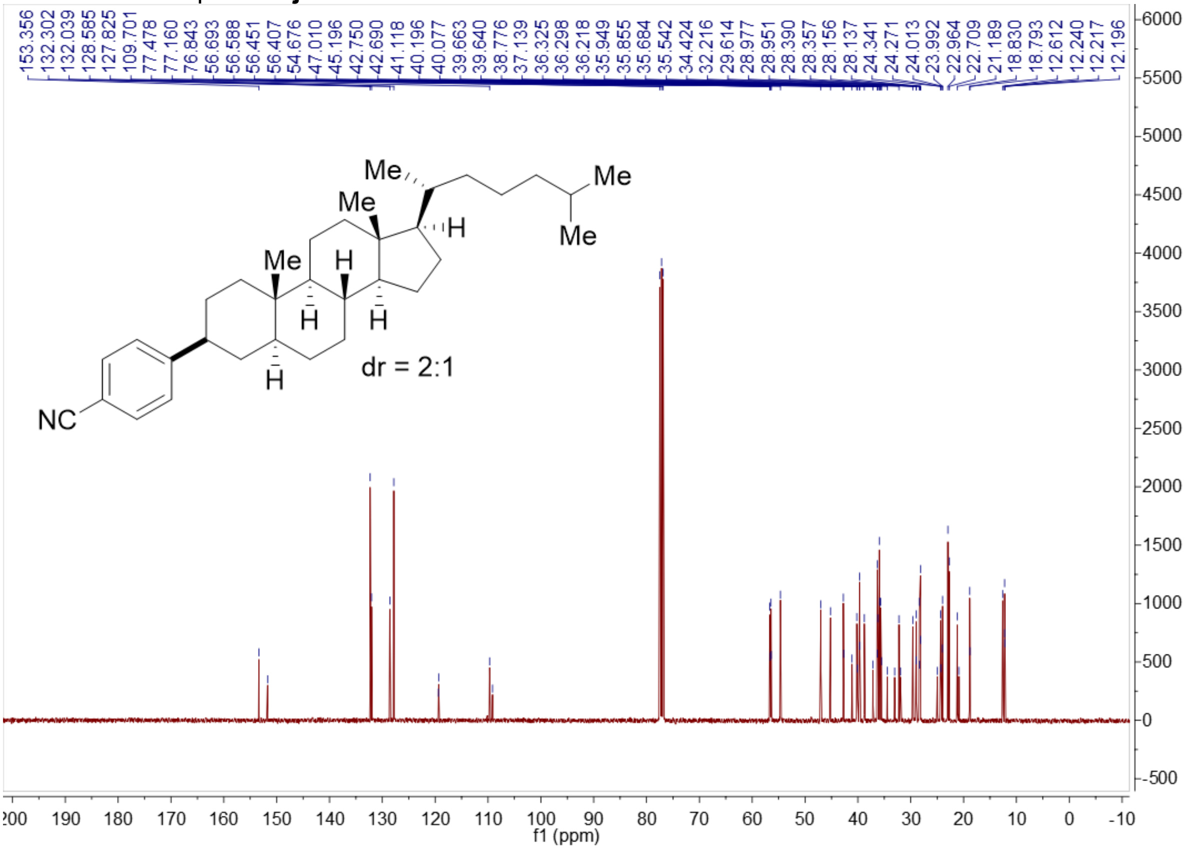

^{1}H NMR of compound **3lac** in $CDCl_3$

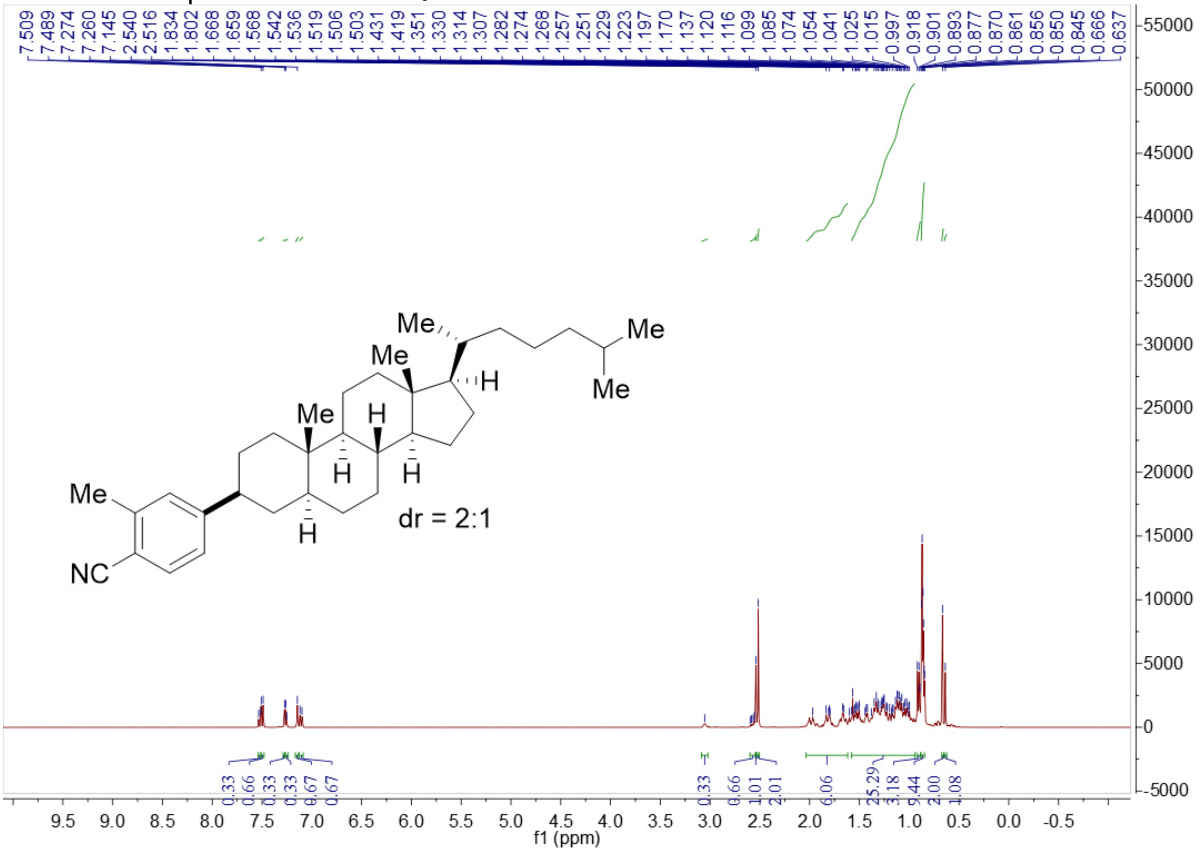

^{13}C NMR of compound **3lac** in $CDCl_3$

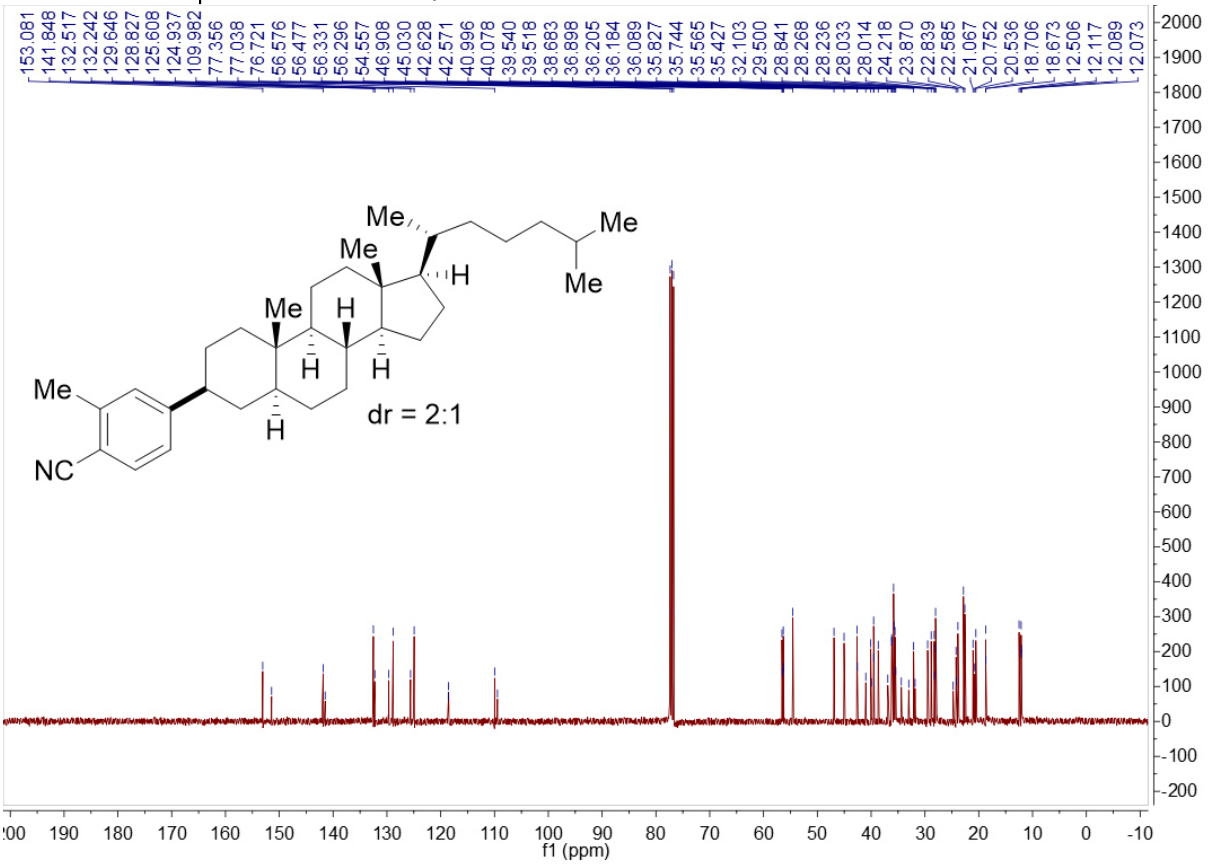

^{1}H NMR of compound **3lad** in $CDCl_3$

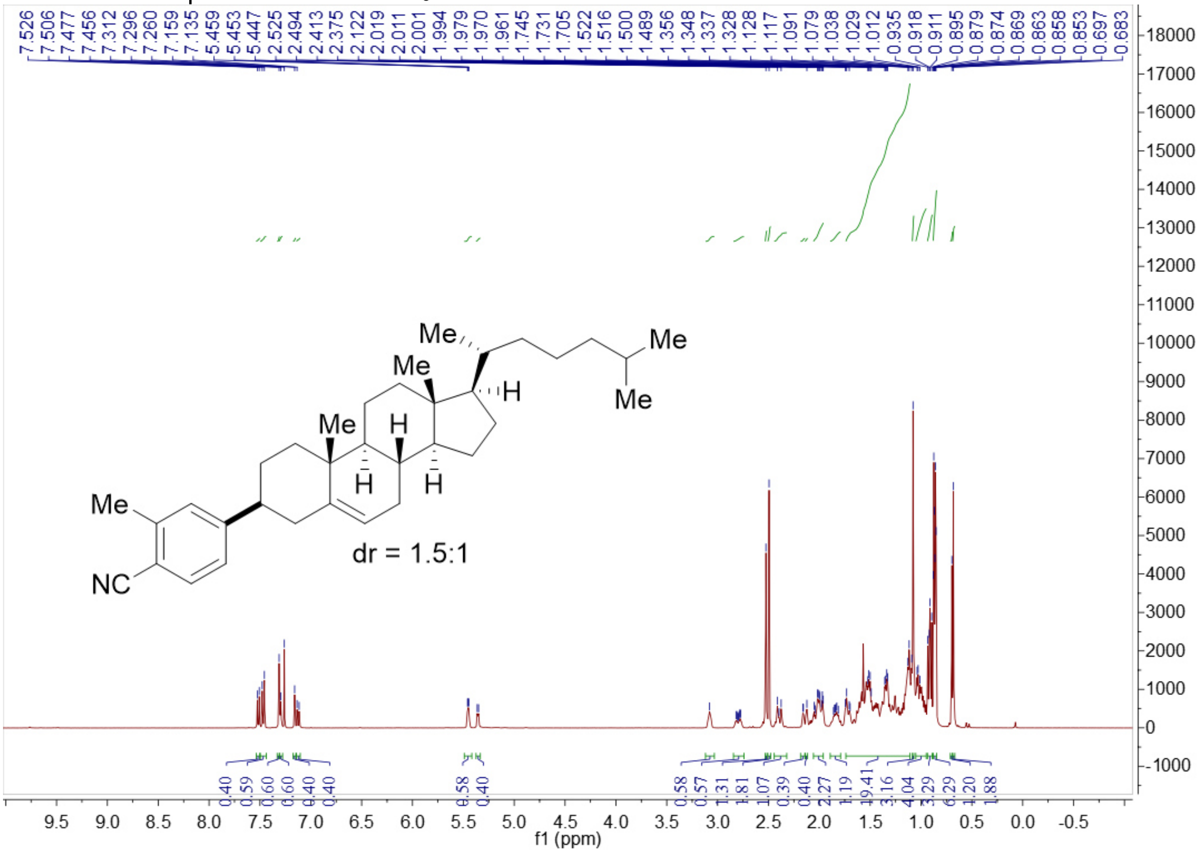

^{13}C NMR of compound **3lad** in $CDCl_3$

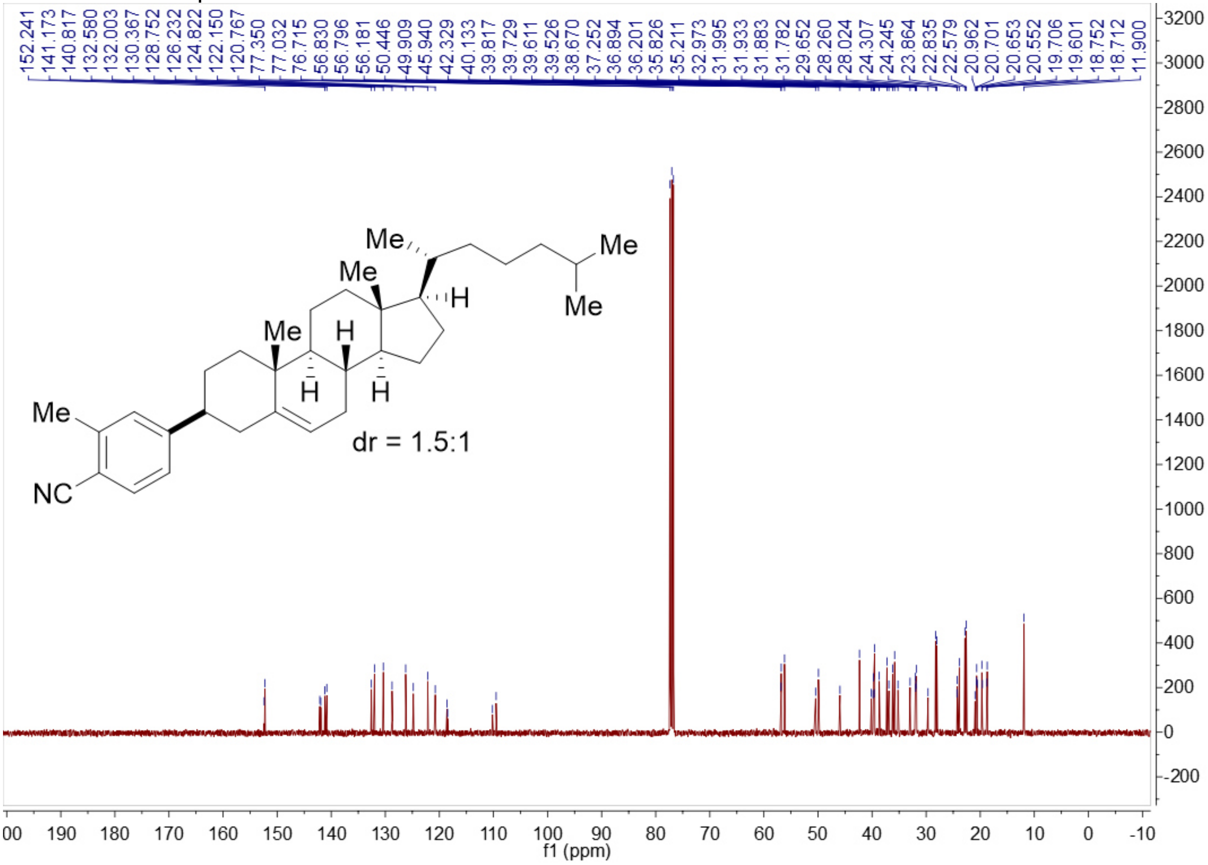

^{1}H NMR of compound **3lae** in $CDCl_3$

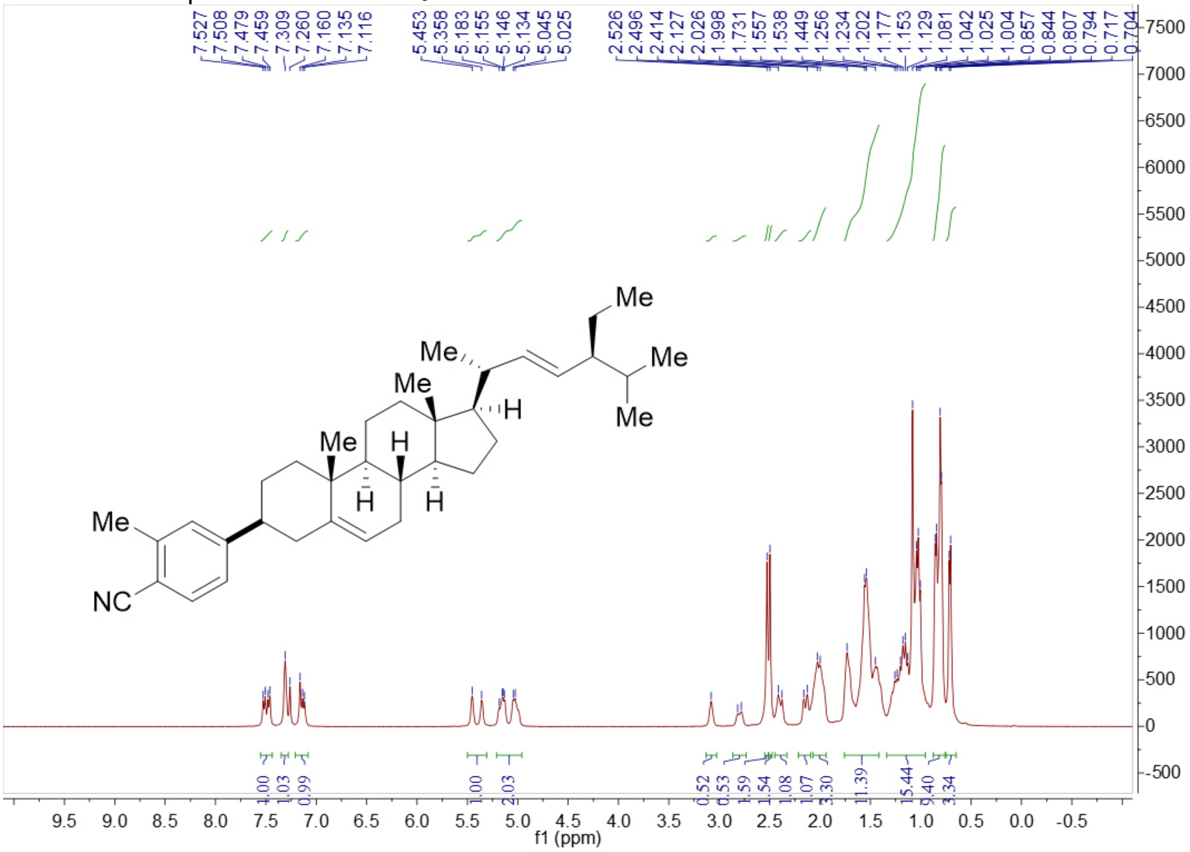

^{13}C NMR of compound **3lae** in $CDCl_3$

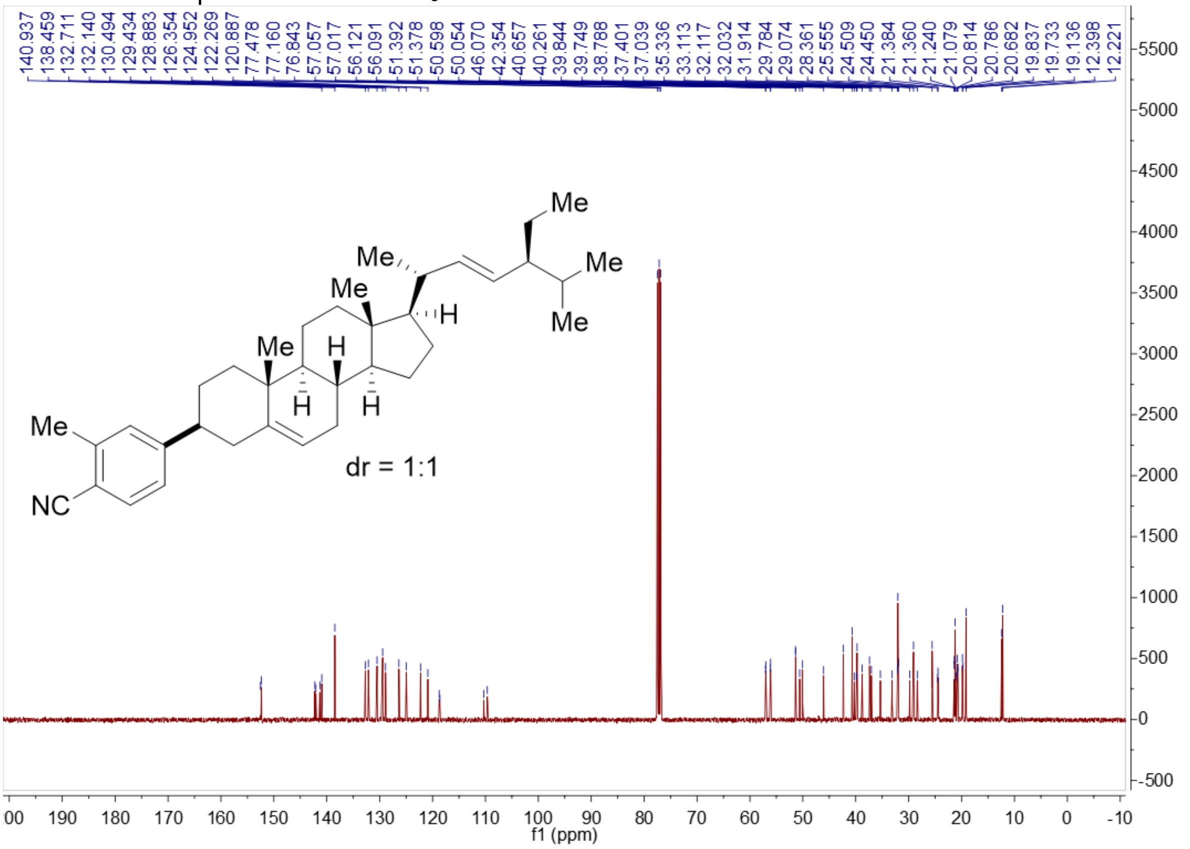

^{1}H NMR of compound **3laf** in $CDCl_3$

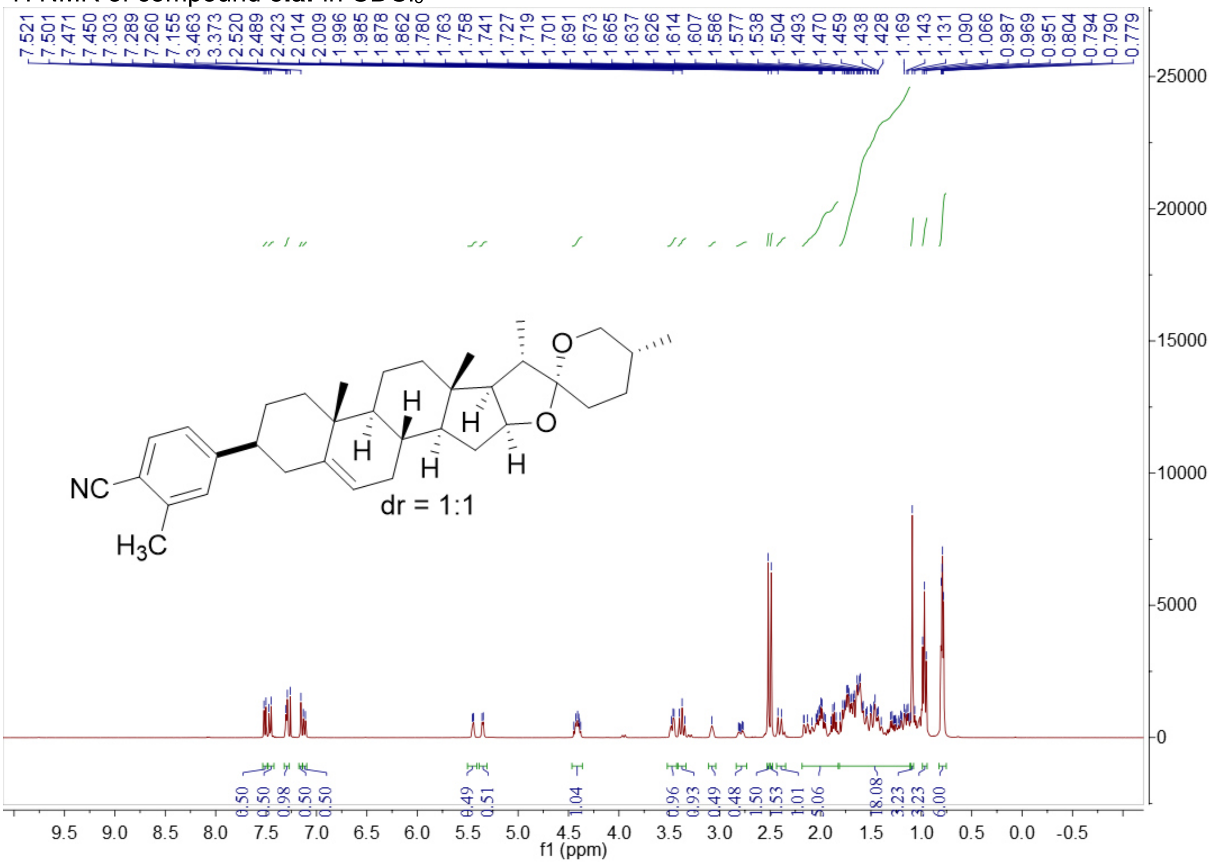

^{13}C NMR of compound **3laf** in $CDCl_3$

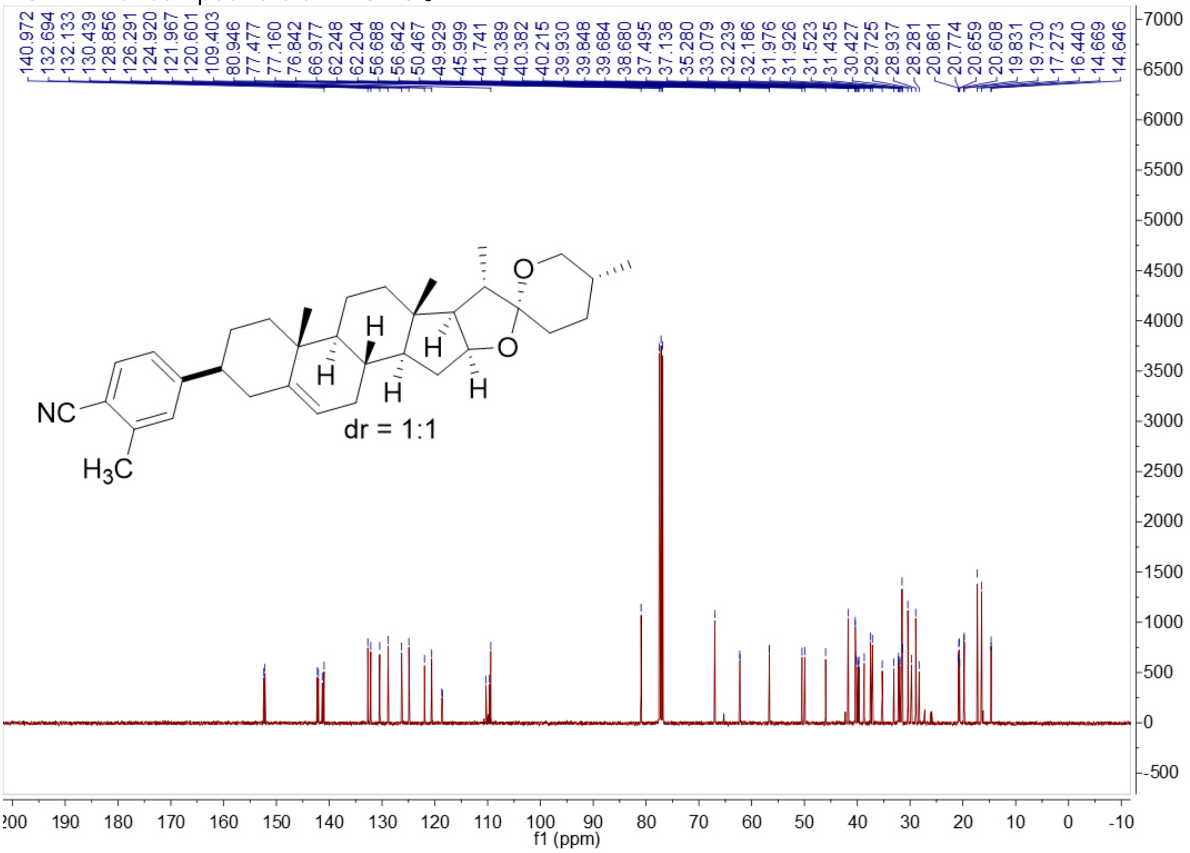

^{1}H NMR of compound **3afn** in DMSO-d_6

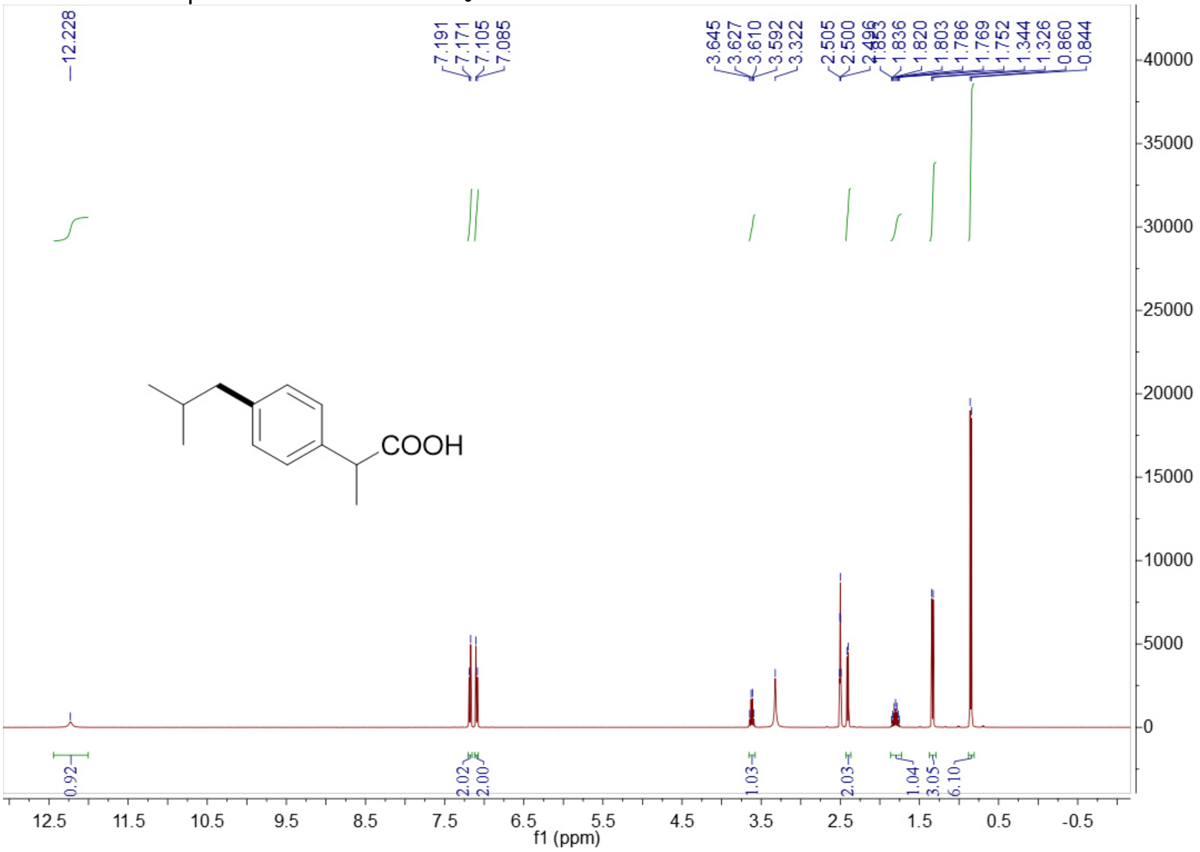

^{13}C NMR of compound **3afn** in DMSO-d_6

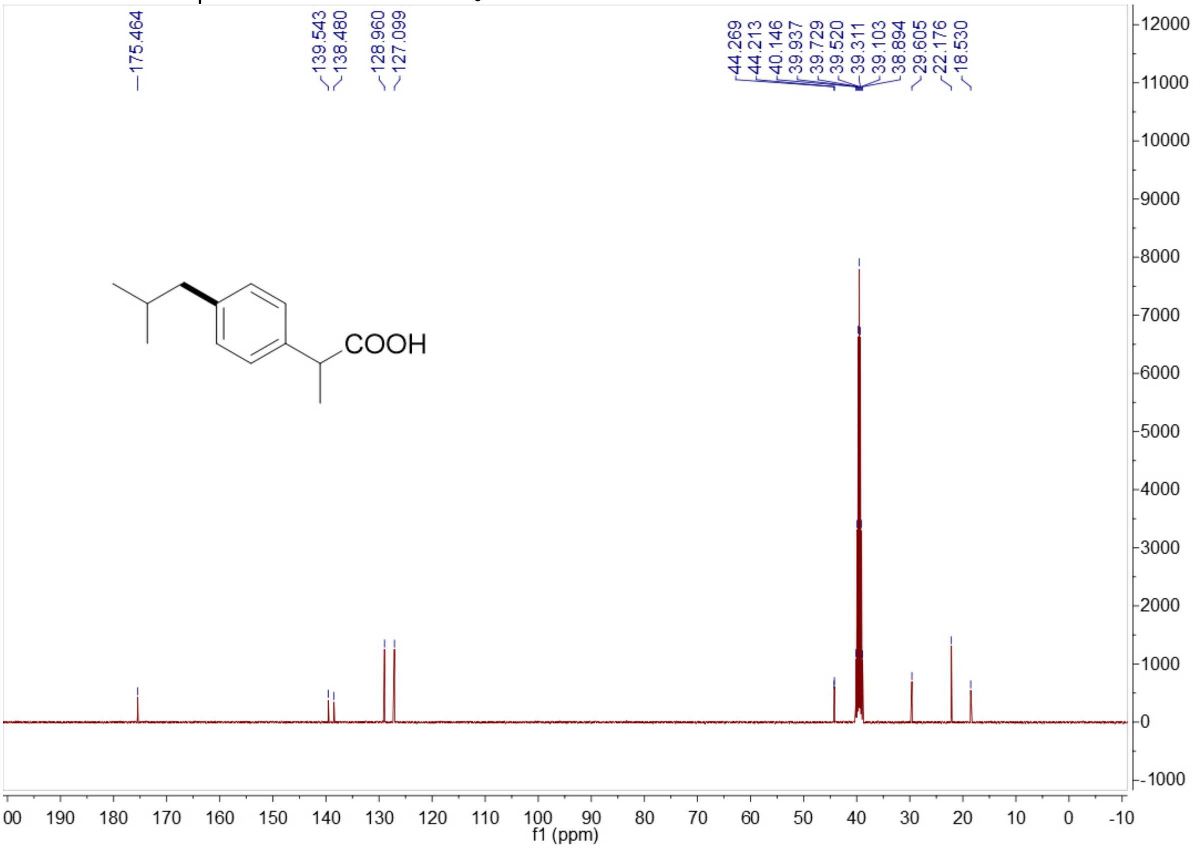

^{1}H NMR of compound **5** in $CDCl_3$

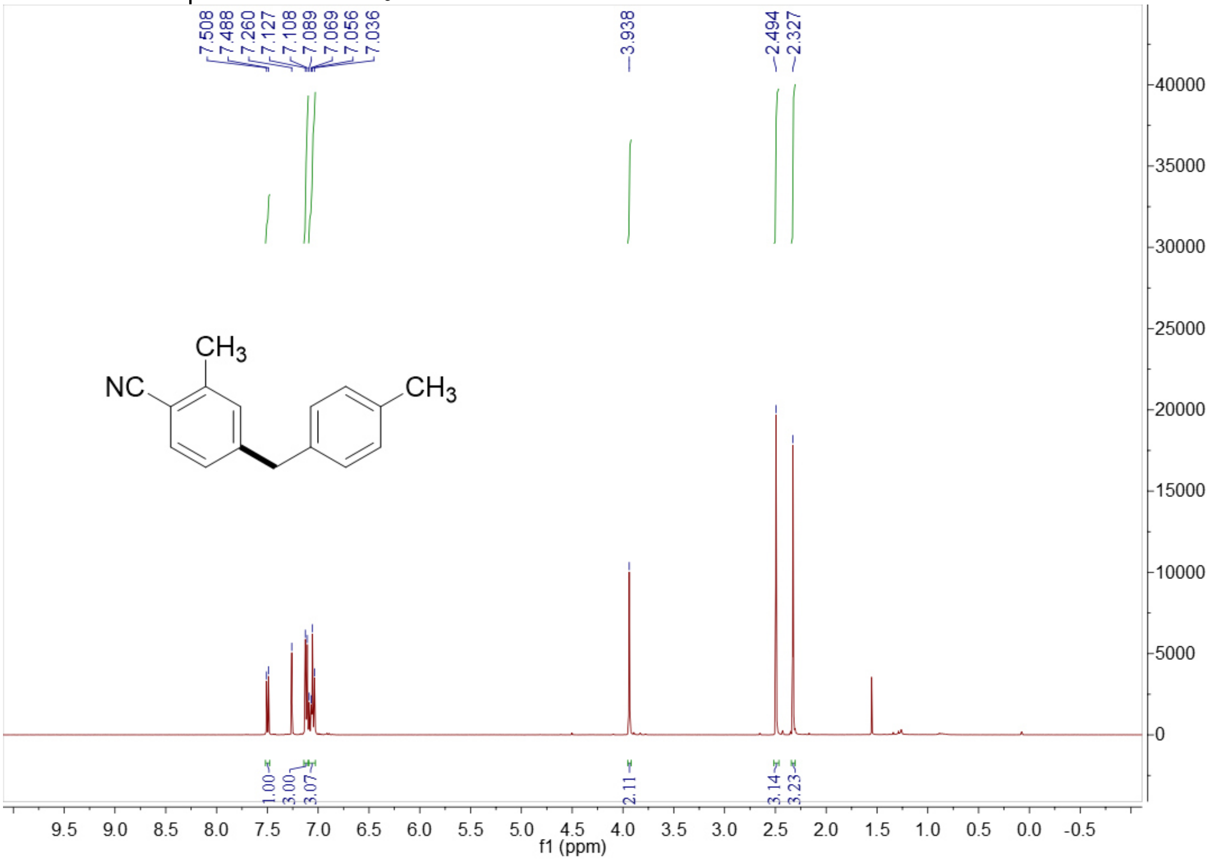

^{13}C NMR of compound **5** in $CDCl_3$

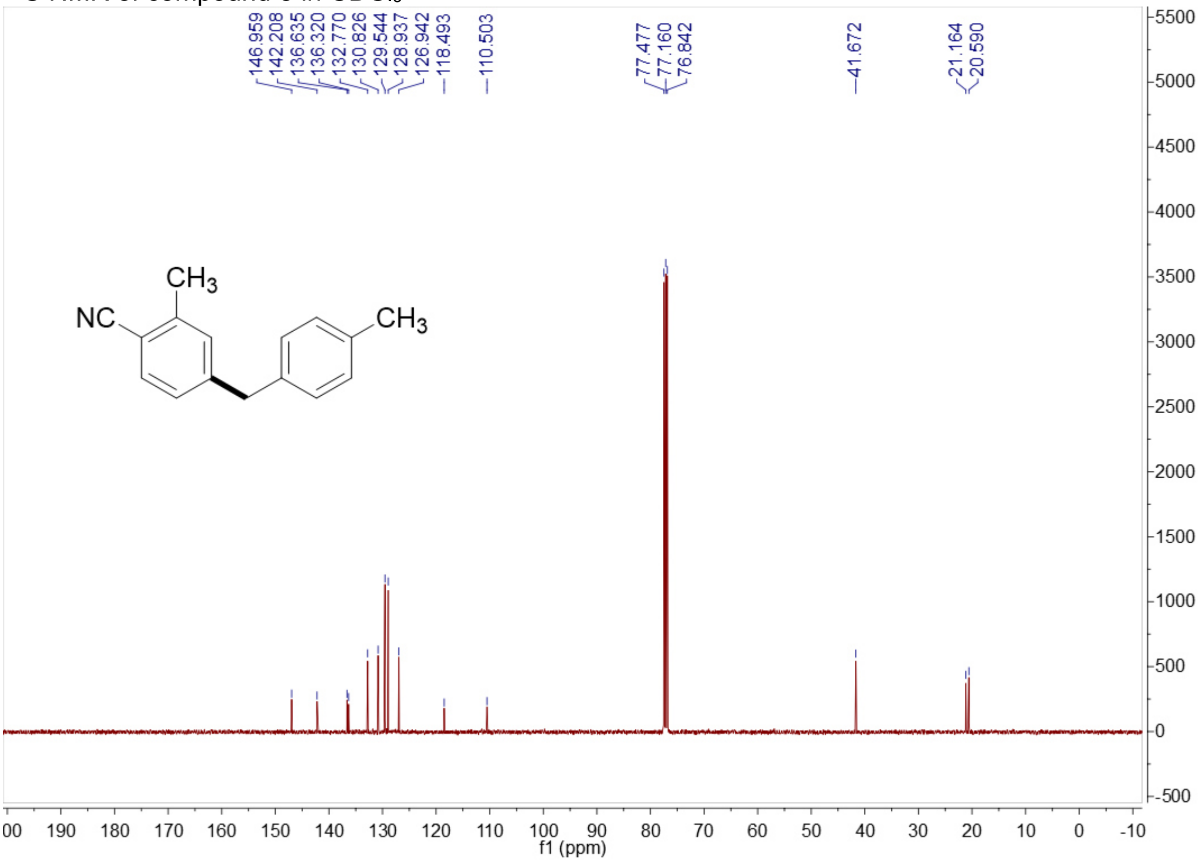

7. REFERENCES

1. K. Lee, D. E. Wiemer, *Tetrahedron Lett.* **1982**, *34*, 2433-2436.
2. J. Chandra, R. M. Manne, S. Mondal, B. Mandal, *ACS Omega* **2018**, *3*, 6120-6133.
3. M. Namavari, N. Satyamurthy, J. R. Barrio, *J. Fluorine Chem.* **1995**, *72*, 89-93.
4. E. G. Corley, K. Conrad, J. A. Murry, C. Savarin, J. Holko, G. Boice, *J. Org. Chem.* **2004**, *69*, 5120-5123.
5. S. Rezazadeh, V. Devannah, D. A. Watson, *J. Am. Chem. Soc.* **2017**, *139*, 8110-8113.
6. A. M. DeBerardinis, M. Turlington, L. Pu, *Angew. Chem. Int. Ed.* **2011**, *50*, 2368-2370.
7. L. Caiger, C. Sinton, T. Constantin, J. J. Douglas, N. S. Sheikh, F. Juliá, D. Leonori, *Chem. Sci.* **2021**, *12*, 10448-10454.
8. J. Nugent, C. Arroniz, B. R. Shire, A. J. Sterling, H. D. Pickford, M. L. J. Wong, S. J. Mansfield, D. F. J. Caputo, B. Owen, J. J. Mousseau, F. Duarte, E. A. Anderson, *ACS Catal.* **2019**, *9*, 9568-9574.
9. A. W. McDonagh, M. F. Mahon, P. V. Murphy, *Org. Lett.* **2016**, *18*, 552-555.
10. J. P. Moerdyk, C. W. Bielawski, *Chem. Eur. J.* **2014**, *20*, 13487-13490.
11. H. Yue, C. Zhu, L. Shen, Q. Geng, K. J. Hock, T. Yuan, L. Cavallo, M. Rueping, *Chem. Sci.* **2019**, *10*, 4430-4435.
12. X. J. Wei, I. Abdiaj, C. Sambiagio, C. Li, E. Zysman-Colman, J. Alcázar, T. Noël, *Angew. Chem., Int. Ed.* **2019**, *58*, 13030-13034.
13. I. B. Perry, T. F. Brewer, P. J. Sarver, D. M. Schultz, D. A. DiRocco, D. W. C. MacMillan, *Nature*, **2018**, *560*, 70-75.
14. X. Zhang, C. Yang, *Adv. Synth. Catal.* **2015**, *357*, 2721-2727.
15. R. Martin-Montero, V. R. Yatham, H. Yin, J. Davies, R. Martin, *Org. Lett.* **2019**, *21*, 2947-2951.
16. G. Antonacci, A. Ahlburg, P. Fristrup, P. O. Norrby, R. Madsen, *Eur. J. Org. Chem.* **2017**, *32*, 4758-4764.
17. H. Shoji, D. Kitagawa, S. Kobatake, *New J. Chem.* **2014**, *38*, 933-941.
18. Y. Zhu, Y. Wei, *Chem. Sci.* **2014**, *5*, 2379-2382.
19. M. Y. Jin, N. Yoshikai, *J. Org. Chem.* **2011**, *76*, 1972-1978.
20. J. Y. Lee, G. C. Fu, *J. Am. Chem. Soc.* **2003**, *125*, 5616-5617.
21. M. Koy, F. Sandfort, A. Tlahuext-Aca, L. Quach, C. G. Daniliuc, F. Glorius, *Chem. Eur. J.* **2018**, *24*, 4552-4555.
22. S. Han, X. Ren, Q. Wu, A. Liang, J. Li, D. Zou, Y. Wu, Y. Wu, *Adv. Synth. Catal.* **2018**, *360*, 2308-2312.
23. R. Kancherla, K. Muralirajan, B. Maity, C. Zhu, P. E. Krach, L. Cavallo, M. Rueping, *Angew. Chem. Int. Ed.* **2019**, *58*, 3412-3416.
24. Y. Kim, T. Iwai, S. Fujii, K. Ueno, M. Sawamura, *Chem. Eur. J.* **2021**, *27*, 2289-2293.
25. J. Zhao, T. Shen, Z. Sun, N. Wang, L. Yang, J. Wu, H. You, Z. Liu, *Org. Lett.* **2021**, *23*, 4057-4061.
26. C.-B. Kim, H. Jo, B.-K. Ahn, C. K. Kim, K. Park, *J. Org. Chem.* **2009**, *74*, 9566-9569.
27. C. Liu, X. Rao, Y. Zhang, X. Li, J. Qiu, Z. Jin, *Eur. J. Org. Chem.* **2013**, *20*, 4345-4350.
28. Y. M. A. Yamada, T. Watanabe, T. Beppu, N. Fukuyama, K. Torii, Y. Uozumi, *Chem. Eur. J.* **2010**, *16*, 11311-11319.
29. N. Yoshikai, H. Matsuda, E. Nakamura, *J. Am. Chem. Soc.* **2009**, *131*, 9590-9599.
30. N. Jeedimalla, C. Jacquet, D. Bahneva, J.-J. Y. Tendoung, S. P. Roche, *J. Org. Chem.* **2018**, *83*, 12357-12373.
31. T. Saetan, C. Lertvachirapaiboon, S. Ekgasit, M. Sukwattanasinitt, S. Wacharasindhu, *Chem. Asian J.* **2017**, *12*, 2221.
32. C. Najerá, J. Gil-Moltó, S. Karlström, L. R. Falvello, *Org. Lett.* **2003**, *5*, 1451-1454.
33. P. Klumphu, B. H. Lipshutz, *J. Org. Chem.* **2014**, *79*, 888-900.
34. M. L. N. Rao, D. N. Jadhav, P. Dasgupta, *Org. Lett.* **2010**, *12*, 9, 2048-2051.
35. A. R. Bogdan, S. L. Poe, D. C. Kubis, S. J. Broadwater, D. T. McQuade, *Angew. Chem. Int. Ed.* **2009**, *48*, 8547-8550.
